# Enhancing control systems of higher plant culture chambers via multilevel structural mechanistic modelling

**DOI:** 10.3389/fpls.2022.970410

**Published:** 2022-10-20

**Authors:** Carles Ciurans, Josep M. Guerrero, Ivan Martínez-Mongue, Claude G. Dussap, Igor Marin de Mas, Francesc Gòdia

**Affiliations:** ^1^ Micro-Ecological Life Support System Alternative (MELiSSA) Pilot Plant-Claude Chipaux Laboratory, Universitat Autònoma de Barcelona, Barcelona, Spain; ^2^ Centre for Research on Microgrids (CROM), Aalborg University, Aalborg, Denmark; ^3^ AAU Energy, Novo Nordisk Foundation Center for Sustainability, Lyngby, Denmark; ^4^ Institut Pascal, Université Clermont Auvergne, Clermont-Ferrand, France; ^5^ Centre for Space Studies and Research - Universitat Autònoma de Barcelona (CERES-UAB), Institut d’Estudis Espacials de Catalunya, Universitat Autònoma de Barcelona, Barcelona, Spain

**Keywords:** closed ecological life support systems, higher plant chambers, functional-structural modelling, model predictive control, FBA, Melissa

## Abstract

Modelling higher plant growth is of strategic interest for modern agriculture as well as for the development of bioregenerative life support systems for space applications, where crop growth is expected to play an essential role. The capability of constraint-based metabolic models to cope the diel dynamics of plants growth is integrated into a multilevel modelling approach including mass and energy transfer and enzyme kinetics. Lactuca sativa is used as an exemplary crop to validate, with experimental data, the approach presented as well as to design a novel model-based predictive control strategy embedding metabolic information. The proposed modelling strategy predicts with high accuracy the dynamics of gas exchange and the distribution of fluxes in the metabolic network whereas the control architecture presented can be useful to manage higher plants chambers and open new ways of merging metabolome and control algorithms.

## 1 Introduction

Modelling crop growth has been a topic of research since the mid-twentieth century given the relevance that agronomic-related activities have in the global economy, but the focus on plant modelling research has evolved in the last decades moving towards new applications (Louarn and Song, 2020). A lot of attention has been placed on developing full-canopy models to assess climate change from different perspectives, such as its effect on crop physiology, the higher plant adaptive strategies, or the contribution of forestry and agricultural systems on carbon dioxide capture, to mention a few (Soussana et al., 2010; Peng et al., 2020). Besides responding to the current global climate and demographic challenges, the need for more efficient forms of horticulture to increase productivities, improve yield, and optimize crop growth has also contributed to the generation of mathematical models to support agronomic activities. Still, much progress is required in the field of biological systems modelling and this is especially relevant in the case of higher plants due to the complexity of their underlying growth mechanisms. Modelling complex systems like higher plants may be an objective by itself as before-mentioned, but they may also lead to other interesting applications like the development of model-based control methodologies. Particularly, space research and the development of bio-regenerative life support systems (BLSSs), which are the set of technologies designed to guarantee life in long-term crewed missions (Eckart, 1995), have exploited the use of controllers based on first principles in opposition to surrogated and reduced order models, to improve both the management of missions under operation as well as to improve the design of future missions (Fulget et al., 1999). One of the main actors on BLSS research is the Micro-Ecological Life Support System Alternative (MELiSSA), a European Space agency (ESA) BLSS program, which is devoted to developing technologies accompanied by a modelling framework to support their research and development activities. One remaining task, which is in turn one of the most relevant consensuses of the development of BLSSs shared by the major space agencies, is the importance of developing technologies and mathematical models to grow plants on space, which are expected to be the major source of edible biomass in BLSSs (Gitelson and Lisovsky, 2002; Poughon et al., 2009; Dong et al., 2017).

Even though current models can cope with the evolution of biomass, the compounds involved in photosynthesis and respiration (O_2_ and CO_2_), and the nutrient uptake by the roots, most of the phenomena involved in plant growth are not addressed given their complexity and lack of knowledge. This complexity is associated with several factors; higher plants are multicellular, compartmentalized organisms undergoing strong metabolic changes associated with the cyclic switch between light and dark phases of the day. They are also characterised by having a complex substrate partitioning strategy with different parts being coordinated to uptake and distribute specific compounds. This has contributed to the preference of empirical at the expense of mechanistic models due to the usually satisfactory information provided by the former, especially in nominal conditions (Boscheri et al., 2012; Amitrano et al., 2020). Empirical models cover a limited range of operating conditions though; thus, the scope of their use is narrow and cannot contemplate all scenarios that plant culture may undergo. As an alternative, mechanistic approaches have also been deployed to understand the first principles behind higher plant growth, surpassing the Farquhar model (Farquhar et al., 1980) to calculate photosynthesis rates based on the enzyme kinetics of principal metabolic pathways. However, out of the photosynthesis process, many key mechanisms like respiration, substrate accumulation and management, tissue morphology, or multi-tissue interactions have not completely been mathematically characterised yet. To treat such a complex system, the use of metabolome information has attracted the attention of plant modellers as an alternative to gather multiple biological reactions formalized as a constraint-based metabolic model (Gomes et al., 2015). It should be highlighted that constraint-based metabolic models have been demonstrated to successfully address the plant diel cycle with a light phase with resource accumulation and a dark phase with resource depletion (Cheung et al., 2014), a critical phenomenon in higher plant metabolism very difficult to deal through first-principle approaches thus far. Several efforts have been recently placed on integrating available mechanistic information and *omics* data in a common multiscale modelling framework that could potentially be used by the plant computational biology research community to feed data in a single converging platform (Marshall-Colon et al., 2017; Xiao et al., 2017).

In this study, the modelling of higher plants is approached through the design of a multilevel organization of the mechanistic processes that take place during crop growth. To present the results, *L. sativa* has been used as an exemplary higher plant. The higher level in the hierarchy copes with the mechanistic phenomena corresponding to a higher characteristic length (i.e., crop chamber scale), whereas the lower the level the smaller the characteristic length of the modelled phenomena (i.e., enzyme rate). Information follows a top-bottom flow, and it is eventually used to calculate the metabolic flux distribution by applying a flux balance analysis (FBA). This multilevel modelling approach is firstly validated with experimental data and secondly integrated in a model-based predictive control, representing, to the best of the authors, the first attempt to incorporate cell metabolism in an advanced control strategy.

Overall, the modelling and control methodology presented in this study may pave the way for a more efficient and sustainable agriculture either for intensive cultivation systems or as a part of BLSSs in space exploration.

## 2 Model proposal

### 2.1 Multilevel mechanistic model

**Figure 1:**
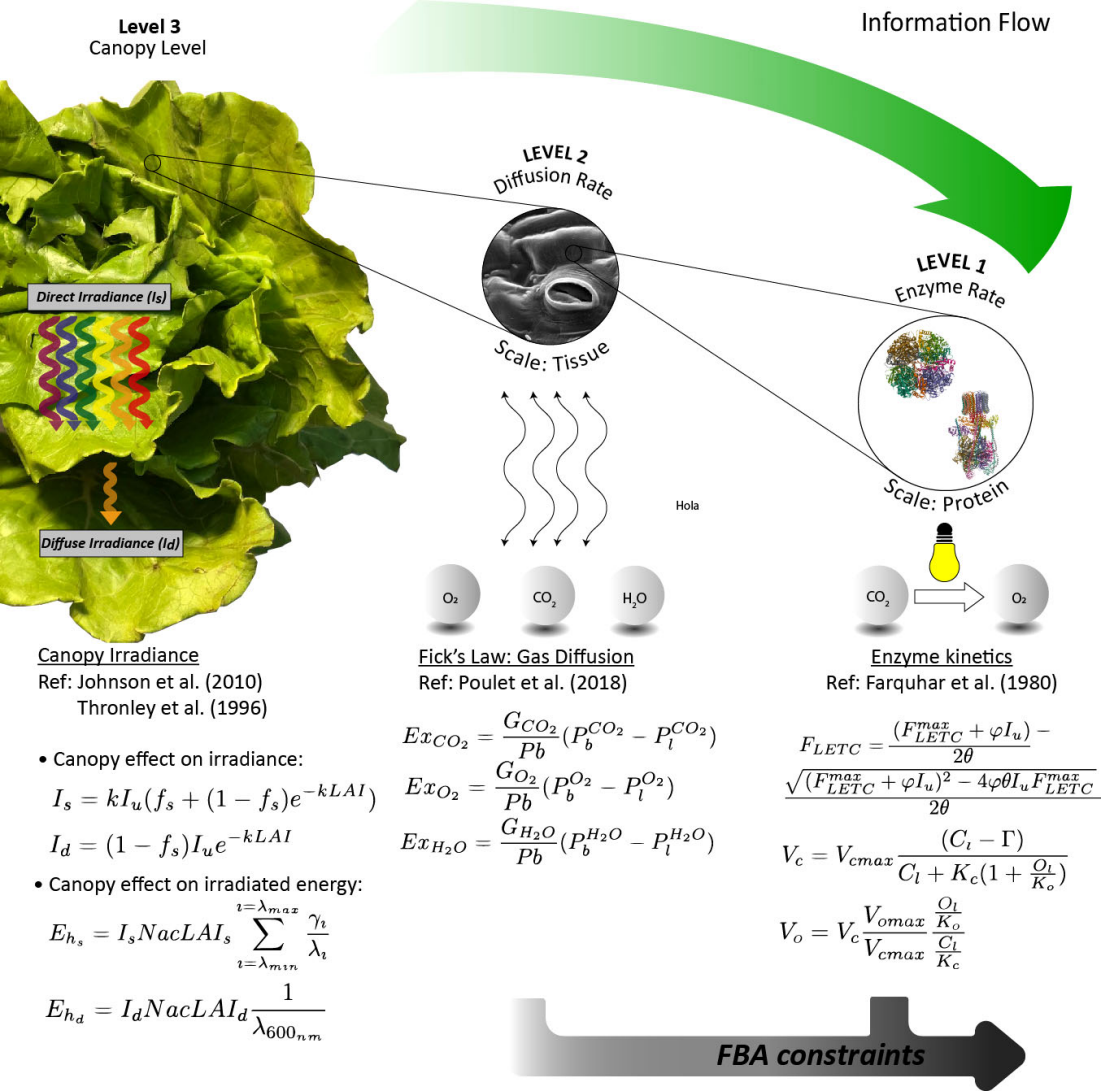
Graphical scheme of the multilevel mechanistic model approach used for *L. sativa* prediction growth.

The model developed here is organized following a multilevel approach, considering the different levels of the plant, from canopy to metabolic level, and uses the output of the higher levels as the input to the lower levels. In this section, the models used in the different levels are explained. A graphical description of the model organization is presented in **Figure 1** and detailed in the following paragraphs. For the sake of clarity, model parameters are described in **Table 1** and constant parameters in **Table 2**.

**Table 1:** Model parameters.

Symbol	Description	Units
***Morphological module***		
*LA*	Leaf area	m^2^ leaves
*L*	Leaf length	m leaves
*M_x_*	Dry biomass	g
**Irradiance module**		
*I*	Irradiance	moles m^-2^ ground s^-1^
*I_u_*	Irradiance at LAI = 0	moles m^-2^ ground s^-1^
*I_s,g_*	Direct irradiance over ground surface	moles m^-2^ ground s^-1^
*I_d,g_*	Diffuse irradiance over ground surface	moles m^-2^ ground s^-1^
*I_s_*	Direct irradiance over leaf surface	moles m^-2^ leaves s^-1^
*I_d_*	Diffuse irradiance over leaf surface	moles m^-2^ leaves s^-1^
*LAI*	Leaf area index	m^2^ leaves m^-2^ ground
*LAI_s_*	LAI exposed to direct irradiation	m^2^ leaves m^-2^ ground
*LAI_d_*	LAI exposed to diffuse irradiation	m^2^ leaves m^-2^ ground
***Energy balance***		
*T_l_*	Leaf temperature	K
*k_t_*	Heat transfer coefficient	m s^-1^
*E_hs_*	Direct irradiance energy	J s^-1^
*E_hd_*	Diffuse irradiance energy	J s^-1^
*E_r_*	Radiation energy	J s^-1^
*E_conv_*	Convection energy	J s^-1^
*E_tr_*	Transpiration energy	J s^-1^
**Gas exchange**		
*Ex*_*CO*_2__	CO_2_ exchange rate	moles m^-2^ leaves s^-1^
*Ex*_*O*_2__	O_2_ exchange rate	moles m^-2^ leaves s^-1^
*Ex*_*H*_2_ *O*_	H_2_O exchange (transpiration) rate	moles m^-2^ leaves s^-1^
*G_z_*	Conductance compound z	moles m^-2^ leaves s^-1^
$P_{b}^{z}$	Bulk partial pressure compound z	Pa
$P_{l}^{z}$	Leaf partial pressure compound z	Pa
**Biochemical module**		
*F_LETC_*	Light electron transport chain rate	moles m^-2^ leaves s^-1^
$F_{LETC}^{max}$	Maximum light electron transport chain rate	moles m^-2^ leaves s^-1^
*J*	Ribulose 1,5-biphosphate regeneration	moles m^-2^ leaves s^-1^
Г	Carbon dioxide compensation point	moles m^-3^
*V_c_*	Carboxylation rate	moles m^-2^ leaves s^-1^
*V_cmax_*	Maximum carboxylation rate	moles m^-2^ leaves s^-1^
*V_o_*	Oxygenation rate	moles m^-2^ leaves s^-1^
*C_i_*	Carbon dioxide leaf concentration	moles m^-3^
*O_i_*	Oxygen leaf concentration	moles m^-3^
*P_g_*	Gross photosynthesis rate	moles m^-2^ leaves s^-1^
*P_n_*	Net photosynthesis rate	moles m^-2^ leaves s^-1^
**Boundary layer**		
$g_{BL}^{z}$	Boundary layer conductance of compound z	moles m^-2^ leavess^-1^
$g_{s}^{z}$	Stomatal conductance of compound z	moles m^-2^ leaves s^-1^
*δ*	Boundary layer thickness	m
*T_bl_*	Average leaf-bulk temperature	K
*D^z^*	Diffusion coefficient of compound z	m^2^ s^-1^
*v*_*bulk*_	Bulk velocity	m s^-1^
*v*_*free*_	Free velocity	m s^-1^
*ρ_l_*	Leaf air density	kg m^-3^

**Table 2:** Model constants.

Symbol	Description	Value	Units	Reference
	**Irradiance module**			
*K*	Extinction coefficient	0.5		MELiSSA Pilot Plant
*f_s_*	Direct irradiance fraction	0.7		Thornley and Johnson (1980)
*BCmol*	C-mole molecular weight	27	g mol-C^-1^	MELiSSA Pilot Plant
	***Energy balance***			
Na	Avogadro number	6.02·10^23^	Pa	
c	Light velocity	3·10^9^	Pa	
*h*	Planck constant	6.63·10^-34^	m^2^ kg s^-1^	
*ϵ*	Leaf emissivity	0.97		Poulet et al. (2020)
σ	Stefan–Boltzmann constant	5.67·10^-8^	J s^-1^ K^-4^	Poulet et al. (2020)
*R*	Ideal gas constant	8.314	m^3^ Pa K^-^1 mol^-1^	
*C_p_*	Molar air-specific heat capacity	29.3	J mol^-1^ K^-1^	Poulet et al. (2020)
*λ_mol_*	Water latent heat of vaporization	4.0788·10^4^	J mol^-1^	Poulet et al. (2020)
	**Biochemical module**			
*θ*	Convexity coefficient	0.8		Farquhar et al. (1980)
*f*	Energy loss for LETC	0.045		Nikolov et al. (1995)
*K_c_*	Carboxylation half-saturation constant	460	μbar	Farquhar et al. (1980)
*K_o_*	Oxygenation half-saturation constant	330	mbar	Farquhar et al. (1980)
$F_{LETC}^{max25}$	$F_{LETC}^{max}$ at 25°C	100	μmol m^-2^ s^-1^	Nikolov et al. (1995)
*V*_*cmax*25_	*V*_*cmax*_ at 25°C	31.31	μmol m^-2^ s^-1^	Nikolov et al. (1995)
*E*	Activation energy of reaction	81,993	J mol^-1^ K^-1^	Nikolov et al. (1995)
*S*	Entropy	711.36	J mol^-1^ K^-1^	Nikolov et al. (1995)
*H’*	Energy of deactivation	219,814	J mol^-1^ K^-1^	Nikolov et al. (1995)
*M_c_*	C-molar molecular weight	27	g mol-C^-1^	MELiSSA Pilot Plant
*DM*	Dry biomass fraction	0.045	g/g	MELiSSA Pilot Plant
	**Boundary layer**			
*η*	Air kinematic viscosity	1.8·10^-5^	m^2^ s^-1^	Poulet et al. (2020)
*α*	Leaf angle in relation to the vertical axis	0.1	°	Poulet et al. (2020)
*g*	Gravity force	9.8	m s^-2^	
*ρ_b_*	Bulk air density	1.186	kg m^-3^	

#### 2.1.1 Level 3: Modelling canopy growth

When dealing with whole-leaf or canopy modelling, it is necessary to consider the effect of shading among leaves, which is not accounted in single-leaf models (Poulet, 2018). The common practice is to use the leaf area index (*LAI*) as an indicative parameter of the leaf area density over ground surface. The photon flux density inside the canopy *I* declines along the canopy exponentially and is a function of the leaf area index:


(1) $$I=I_{u}e^{\left(-kLAI\right)}$$

Parameter *I_u_* represents the photon flux density at the top of the canopy, and *k* represents the extinction coefficient. Extending (1), leaves can receive direct photon flux density (*I_s_*) or diffuse photon flux density (*I_d_*) as stated by Thornley (2002), which are expressed in terms of μmol m^-2^ leaf s^-1^ by using the extinction coefficient *k:*


(2) $$I_{s}=k\cdot f_{s}\cdot I_{u}+k\cdot (1-f_{s})\cdot I_{u}\cdot e^{\left(-k\cdot LAI\right)}$$

(3) $$I_{d}=k\cdot (1-f_{s})\cdot I_{u}e^{\left(-k\cdot LAI\right)}$$

Notice in (2) that the parts of the canopy under direct irradiance also receive diffuse irradiance. The *LAI* term should be differentiated between the fractions exposed to direct and diffuse light sources (*LAI_s_* and *LAI_d_* respectively), as suggested by Thornley (2002):


(4) $$LAI_{s}=(1-e^{-kLAI})/k$$

(5) $$LAI_{d}=LAI-LAI_{s}$$

The derivative of *LAI_s_* and *LAI_d_* should be obtained and used to integrate *I_s_* and *I_d_* to calculate the overall irradiance received by the canopy:


(6) $$dLAI_{s}=e^{-kLAI}\;dLAI$$

(7) $$dLAI_{d}=\;(\;1-e^{-kLAI})\;\;dLAI$$

(8) $$I_{s,l}=\int_{0}^{LAI}I_{s}dLAI_{s}$$

(9) $$I_{d,l}=\int_{0}^{LAI}I_{d}dLAI_{d}$$

The way light irradiates exposed and shadowed leaves strongly affects the energy balance in the leaf surface, with the shadowed leaves irradiated by diffused light mainly of a wavelength of 600 nm corresponding to the green-colour spectrum of transmitted light. Energy received by irradiance contains the direct (*E_hs_*) and diffuse (*E_hd_*) terms, which are expressed as follows:


(10) $$E_{h_{s}}=I_{s}\cdot Na\cdot c\cdot h\cdot LAI_{s}\sum_{i=\lambda_{min}}^{i=\lambda_{max}}\frac{\gamma_{i}}{\lambda_{i}}$$

(11) $$E_{h_{d}}=I_{d}\cdot Na\cdot c\cdot h\cdot LAI_{d}\frac{1}{\lambda_{600nm}}$$

*Na* represents the Avogadro number, *c* represents the velocity of light, and *γ* is the fraction of wavelength *λ* that compose the light directly irradiating the canopy. The total energy irradiated to the leaves is the summation of both Equations (10) and (11). The radiation energy emitted by the plants (*E_r_*), the energy lost by convection (*E_conv_*), and the energy lost by transpiration (*E_tr_*) are determined by the following equations:


(12) $$E_{r}=\epsilon \sigma \left(T_{leaf}^{4}-T_{b}^{4}\right)$$

(13) $$E_{conv}=C_{p}k_{t}\frac{P_{b}}{RT_{b}}(T_{leaf}-T_{b})$$

(14) $$E_{tr}=\lambda_{mol}Ex_{H_{2}O}$$

In (12), *ϵ* and *σ* represent the leaf emissivity and the Stefan–Boltzmann constant, respectively. In (13), *C_p_* and *k_t_* represent the molar specific heat capacity at constant pressure and 298.15 K and the heat transfer coefficient, respectively, the latter being a function of the diffusion coefficient and the boundary layer thickness, as follows (see **Supplementary S1** for details on *D^t^* calculation and for an extended description of the boundary layer model):


(15) $$k_{t}=\frac{{\text{D}}^{t}}{\delta }$$

In (14), *λ_mol_* is the water latent heat of vaporization and *Ex*_*H*_2_ *O*_ the transpiration rate defined in the following section. Finally, *T_leaf_* is the leaf surface temperature, *T_b_* is the bulk temperature, and *R* is the ideal gas constant.

#### 2.1.2 Level 2: Modelling gas exchange rates

This level is dedicated to calculating the uptake and release rates between the atmosphere and the leaves concerning the exchange gases (*Ex*_*CO*_2__ , *Ex*_*O*_2__ , and *Ex*_*H*_2_ *O*_ ). The approach to modelling gas exchanges between the leaves and the atmosphere follows Fick’s law, with the concentration gradient being the driver of the molecular transport:


(16) $$Ex_{CO_{2}}=\frac{{\text{G}}^{CO_{2}}}{P_{b}}(P_{b}^{CO_{2}}-P_{l}^{CO_{2}})$$

(17) $$Ex_{O_{2}}=\frac{{\text{G}}^{O_{2}}}{P_{b}}(P_{b}^{O_{2}}-P_{l}^{O_{2}})$$

(18) $$Ex_{H_{2}O}=\frac{{\text{G}}^{H_{2}O}}{P_{b}}(P_{b}^{H_{2}O}-P_{l}^{H_{2}O})$$

In (16)–(18), the atmospheric partial pressure is calculated assuming gases behave following the general gas equation, whereas the conductance for the different gases (G^*CO*_2_^ , G^*O*_2_^ , and G^*H*_2_ *O*^ ) and the internal (i.e., leaf) partial pressure ( $P_{l}^{CO_{2}}$ , $P_{l}^{O_{2}}$ , $P_{l}^{H_{2}O}$ ) as well as the leaf area (*LA*) are calculated according to Poulet et al. (2018) and explained in **Supplementary S1**.

#### 2.1.3 Level 1: Modelling enzyme kinetics

The gross photosynthesis rate (*P_g_*) is calculated using the Farquhar model (Farquhar et al., 1980), which has been widely used to model photosynthesis phenomena (Harley et al., 1992; Morgan and Robles, 2002; Arnold and Nikoloski, 2011). In this model, *P_g_* is determined through finding the limiting rate of the photosynthesis, which is caused by either the regeneration of ribulose 1,5-biphosphate (*J*), substrate of the ribulose-1,5-biphosphate carboxylase (RuBisCo), or the RuBisCO carboxylation rate itself (*V_c_*).

On the one hand, the regeneration of ribulose 1,5-biphosphate depends on the potential rate of the light electron transport chain (*F_LETC_*) and its capacity to generate reducing power. Thus, it is necessary to define an expression for *F_LETC_*, which can be approximated by a quadratic equation (Nikolov et al., 1995):


(19) $$F_{LETC}=\frac{(F_{LETC}^{max}+\varphi I_{u})-\sqrt{{\left(F_{LETC}^{max}+\varphi I_{u}\right)}^{2}-4\varphi \theta I_{u}F_{LETC}^{max}}}{2\theta }$$

(20) $$\text{φ}=\frac{\left(1-\text{f}\right)}{2}$$

In (19), the maximum *F_LETC_* is represented by $F_{LETC}^{max}$ (see **Supplementary S1** for calculation details). The photosynthetic photon flux density used is represented by *I_u_* and *θ* is a convexity coefficient. The efficiency of energy conversion is represented by φ, which is a function of the fraction of absorbed photon flux unavailable for photosynthesis (*f*). As previously demonstrated, it is necessary to consider the direct and diffuse irradiances when considering the whole canopy. Therefore, combining with (4) and (5):


(21) $$F_{LETC(I_{s},I_{d})}=\int_{0}^{LAI}\frac{F_{LETC}(I_{s})\;dLAI_{s}}{2}+\int_{0}^{LAI}\frac{F_{LETC}(I_{d})\;dLAI_{d}}{2}$$

Considering that two electrons are necessary per molecule of NADPH generated, the light electron transport chain rate resulting from (21) is divided by 2. This is necessary to provide information to the FBA with consistent units considering the stoichiometry matrix used (see S2 for a full list of reactions and stoichiometry matrix).

As mentioned before, in Farquhar et al.’s (1980) approach, it is necessary to convert *F_LETC_* defined in (21) to a flow of ribulose 1,5-biphosphate (Ru5P) regeneration (*J*), through the following expression derived from the stoichiometry of the light electron transport chain and the Calvin cycle (Farquhar et al., 1980):


(22) $$J=\frac{F_{LETC}}{2(2+2\emptyset )}$$

The RuBisCo carboxylation *(V_c_*) kinetics is of Michaelis–Menten type and is a function of the leaf internal oxygen (*O_i_*) and carbon dioxide (*C_i_*) concentrations:


(23) $$V_{c}=V_{cmax}\frac{\left(C_{i}-\Gamma \right)}{C_{i}+K_{c}(1+\frac{O_{i}}{K_{o}})}$$

In (23), *V_cmax_* represents the maximum carboxylation velocity of RuBisCo, *K_c_* and *K_o_* are the Michaelis–Menten half-saturation constants for the carboxylation and oxygenation activities of RuBisCo, respectively, and *Г* represents the carbon dioxide compensation point. Details about the calculation of the internal carbon and oxygen concentrations are found in **Supplementary S1**, based on the boundary layer approach defined by Poulet et al. (2018; 2020). *V_c_* can be directly used to feed the FBA as an upper bound (see **Figure 1**).

Finally, the gross photosynthesis rate (*P_g_*) is determined by finding the minimum between the Ru5P regeneration rate (*J*), the RuBisCo carboxylation rate (*V_c_*), and the gas exchange rate (*Ex*_*CO*_2__ ) and the net photosynthesis rate (*P_n_*) is determined by retrieving the RuBisCo oxygenation (*V_o_*) to *P_g_* as follows:


(24) $$P_{g}=min(V_{c},Ex_{CO_{2}},J)\cdot LA$$

(25) $$V_{o}=V_{c}\cdot \frac{V_{omax}}{V_{cmax}}\cdot \frac{\frac{O_{l}}{K_{o}}}{\frac{C_{l}}{K_{c}}}$$

(26) $$P_{n}=P_{g}-V_{o}\cdot LA$$

Notice that Equation (24) can be formalized due to the conversion applied in Equation (22) considering that *J* represents the regeneration rate of Ru5P and *V_c_* represents its carboxylation rate. Parameter *A* indicates the surface of the crop growing area. In this study, the use of a metabolic matrix makes the conversion from light electron transport flux (*F_LETC_*) to RuBP regeneration (J) unnecessary, because this information is already included in the stoichiometric matrix. Similarly, the discontinuity introduced by Farquhar et al. (1980) in (24) can be prevented with an FBA formulation as addressed in the following section.

#### 2.1.4 Level 0: Stoichiometry matrix

**Figure 2:**
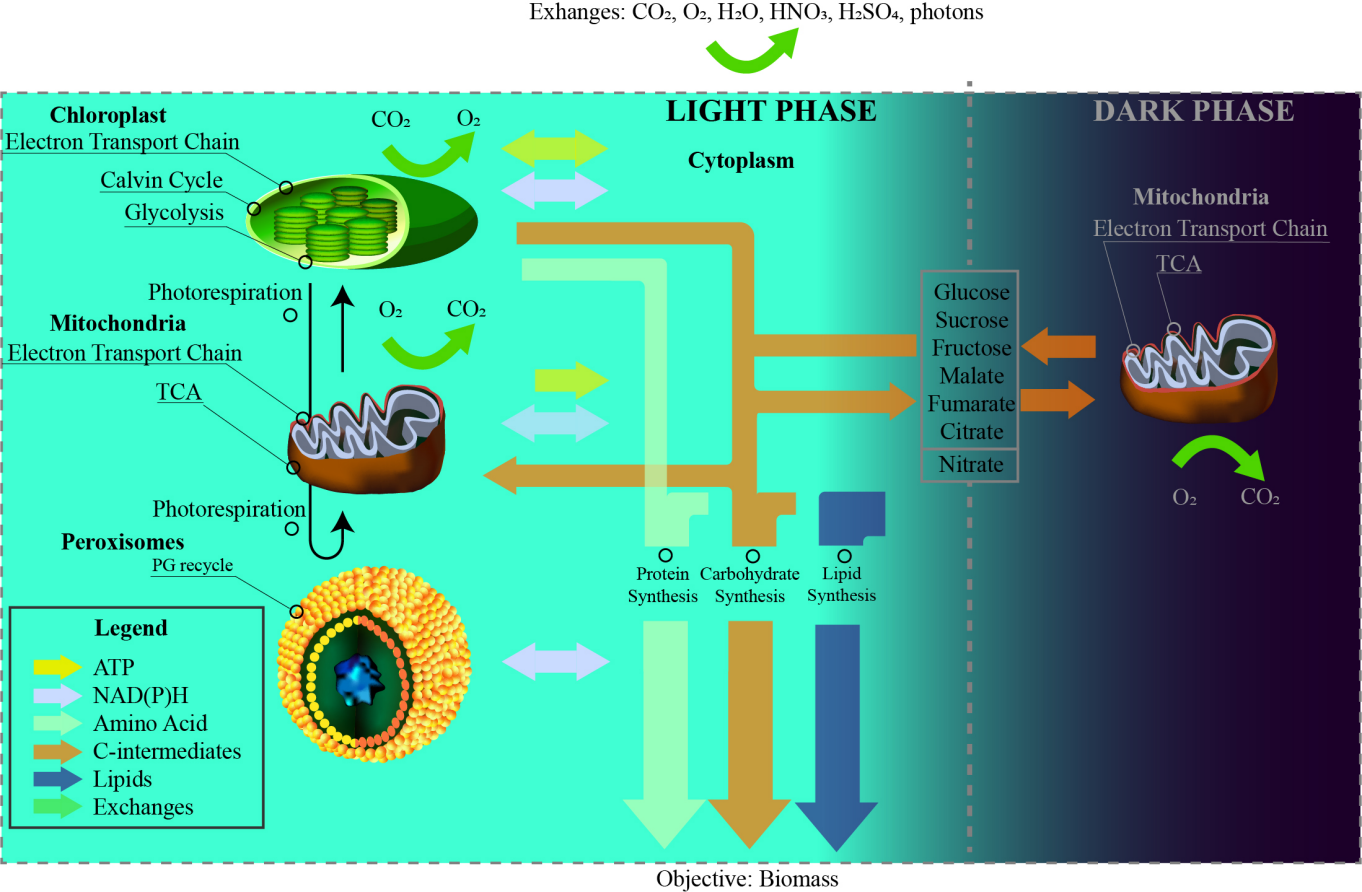
Structure of the diel model presented. Four compartments (chloroplast, mitochondria, peroxisomes, and cytoplasm) and the two phases of the day (light and dark) are considered with exchange reactions including metabolites diffused through leaves (CO_2_, O_2_, H_2_O, light photons) and through the roots (HNO_3_ and H_2_SO_4_). PG represents 2-phospho-glycolate.

In this level, a simplified network model of the photosynthetic leaves’ metabolism of *L. sativa* is described. The stoichiometry matrix is based on the work of Sasidharan (2012), which contains the distinguishing characteristics of *L. sativa*, such as the reduced starch content to store carbon and the definition of the elemental composition that makes up the macromolecules of the biomass. This model though has been extended to include relevant reactions like the pentose phosphate pathway or the photorespiration cycle, originally missing. In the model used, the cellular organelles are described as different compartments. The model also describes dark and light phases of the day by duplicating each one of the reactions. Hence, a diel model is achieved where both light and dark phases of the day account for separate pools of metabolites and organelle compartments. Here, only those metabolites that have been reported to be accumulated in one phase and consumed in the other are connected by exchange reactions that simulate the transference of nutrients between phases. For example, sugars that are synthesised in the light phase can be used in the dark phase due to the addition of exchange reactions among day phases. In **Figure 2**, the metabolic model is presented in a simplified way including the cellular compartmentalization, the pathways involved, and the connections between them as well as the exchange reactions with the atmosphere and between day periods.

The diel model is composed by different organelles including chloroplasts, mitochondria, peroxisomes, and cytoplasm. The proposed model also features plants’ simultaneity of metabolic pathways (i.e., glycolysis in chloroplast and cytoplasm or the folate metabolism in chloroplasts, cytoplasm, and mitochondria) as well as the aforementioned coordination between day phases. A summary of the metabolic model is found in **Supplementary S3**, and the model in SBML format is accessed in **Supplementary S4**.

The metabolic network model is mathematically formalized as a constraint-based model, and the fluxes are calculated by applying a flux balance analysis (Cheung et al., 2014):


(27) $$\begin{array}{ll} \begin{array}{c} max\\ v \end{array} & \left(v_{biomass}\left[d\right]\right)\\ subject: & \begin{array}{l} lb\leq Sv\leq ub\\ Cv=F \end{array} \end{array}$$

In the FBA formulation in (27), the objective function refers only to the daily biomass production. It is well known for other crop species like *Arabidopsis thaliana* that biomass growth also takes place during the dark phases of the day (Gomes et al., 2015). However, night metabolism of non-starchy crops like *lettuce* is still not clear so in the current approach maintenance-associated reactions are limited to night metabolism whereas metabolism associated with light periods of the day concentrates, on top of maintenance, also biosynthetic reactions. Letter *υ* represents the array of fluxes for each of the reactions of the model. The lower and upper bounds (*lb* and *ub*) are fixed only for those fluxes indicated in **Table 3**. Matrix *C* contains information regarding the flux ratios specified in **Table 3**, and matrix *F* represents the resulting flux. The inequality constraints represented in **Table 3** are generated in levels 3 and 2 of the model and are used to feed the FBA. ATP_maintenance_ and NAD(P)H_maintenance_ includes the reactions that contribute to the consumption of ATP and the reducing agent for respiration purposes. As suggested in Cheung et al. (2013), this can be achieved including generic ATPase and NAD(P)H oxidase reactions. Finally, the enzyme rates included are those related to constrain the reducing power supply in the cytoplasm. At night, plastidic NADP-malate dehydrogenase and glyceraldehyde 3-phosphate dehydrogenase are downregulated (Miginiac-Maslow and Lancelin, 2002; Mekhalfi et al., 2014). Finally, minimums and maximums for a set of reactions are defined given the presence of thermodynamically infeasible loops when no restrictions are applied. These reactions include the following: PYK, pyruvate kinase; PGM: phosphoglycerate mutase; ENO: enolase; EB1: inorganic pyrophosphatase; EB2: inorganic pyrophosphatase; ACS: acetyl-CoA synthetase; Ser_bio_cl: phosphorylated serine pathway; GOGAT: glutamate synthase; Prot32: 3-mercaptopyruvate sulfurtransferase/cytoplasmic aspartate aminotransferase; OASTL: cysteine synthase; GS: glutamine synthetase. The FBA presented is implemented both in Matlab ® 2021 using the Cobra Toolbox (Becker et al., 2007) and in python 3.0 using Cobrapy (Ebrahim et al., 2013) and can be found in **Supplementary S5**.

**Table 3:** Flux balance analysis equality, inequality, and flow ratio restrictions. [d] and [n] indicate day and night phase period respectively.

Inequality constraints	Type	Description
*ν*_*Ex*_*CO*_2___	lb	Gas exchange
*ν*_*Ex*_*O*_2___	ub	Gas exchange
*ν*_*Ex*_*H*_2*O*___	lb	Gas exchange
*ν*_*F*_*LETC*__	ub	Light ETC
*ν*_*V*_*c*__	ub	Carboxylation
*ν*_*V*_*o*__	lb	Oxygenation
**Flux ratios**	**Value**	
*ν*_*Ex*_*O*_2___[*d*] : *ν*_*Ex*_*CO*_2___[*d*]	1.22:-1	Photosynthesis rate (MELiSSA Pilot Plant)
*ν*_*ATPmaintenance*_[*d*/*n*]: *ν*_*NADPHmaintenance*_[*d*/*n*]	3:1	Maintenance (Cheung et al., 2014)
*ν*_*Ex*_*CO*_2___[*d*] : *ν*_*Ex*_*CO*_2___[*n*]	-1:0.25	Respiration (MELiSSA Pilot Plant)
*ν*_*ATPmaintenance*_[*d*]:*ν*_*ATPmaintenance*_[*n*]	1	Respiration (MELiSSA Pilot Plant); (Liu and van Iersel, 2021)
*ν*_*NADPHmaintenance*_[*d*]:*ν*_*NADPHmaintenance*_[*d*]	1	Respiration (MELiSSA Pilot Plant); (Liu and van Iersel, 2021)
*ν*_*OPPP*_[*c*,*m*,*cl*]__[*d*/*n*]+*ν*_*ICDH*_[*c*,*m*]__[*d*/*n*]+*ν*_*ME*_[*c*,*m*]__[*d*/*n*]:*ν*_*NADPHmaintenance*_[*d*/*n*]	1:1	(Corpas and Barroso, 2014)
*ν*_*SGT*_[*p*]__[*d*/*n*] : *ν*_*GT*_[*p*]__[*d*/*n*]	1:1	(Yu et al., 1984)
**Enzyme rates**	**Value**	
*ν*_*GAPN*_[*c*]__	ub: 0.33	(Shameer et al., 2019)
*ν*_*GAPDH*_[*c*]__	lb: -93	(Shameer et al., 2019)
*ν*_*MDH*_[*c*]__	lb: -0.75	(Shameer et al., 2019)

^*^NADPH oxidation and ATP hydrolysis associated with maintenance reactions include plastidic, cytoplasmic, and mitochondrial locations.

Subscripts *c*, *m*, *cl*, and *p* indicate cytoplasmic, mitochondrial, plastidic, and peroxisomal location. lb and ub represent lower and upper bounds, respectively. List of enzyme abbreviations: GAPN, cytosolic non-phosphorylating NADP-glyceraldehyde-3-phosphate dehydrogenase; GAPDH, cytosolic glyceraldehyde 3-phosphate dehydrogenase; MDH, cytosolic malate dehydrogenase; OPPP, glucose-6-phosphate dehydrogenase and 6-phosphogluconate dehydrogenase; ICDH, isocitrate dehydrogenase; MDH, malate dehydrogenase; SGT, serine-glyoxylate aminotransferase; GT, glutamate-glyoxylate aminotransferase.

#### 2.1.5 Dynamic model

The evolution of the different states of interest through time for either dry biomass, leaf temperature, or gas compositions can be obtained integrating their rates of generation or consumption over time. For dry biomass, this can be done straightforward from *Pn*, considering that all carbon molecules captured by the plant are fixed into structural biomass:


(28) $$M_{x}=\int_{t=t0}^{t=\;t_{f}}Pn\cdot BCmol\cdot dt$$

For the evolution of leaf temperature, it is necessary to solve an energy balance between the leaf temperature and the environment and to convert energy units to temperature degrees:


(29) $$T_{l}=\int_{t=t0}^{t=\;t_{f}}\frac{(E_{hs}+E_{hd})A-(E_{r}+E_{conv}+E_{tr})LA}{C_{p}\frac{M_{x}}{DM}}\cdot dt$$

Finally, the oxygen concentration can be obtained by solving a mass balance within the growing crop chamber:


(30) $$O_{2}=\int_{t=t0}^{t=\;t_{f}}\frac{u\cdot (O_{2}^{in}-O_{2})+v_{Ex_{O_{2}}}}{\text{V}}\cdot dt$$

In (30), gas flow is represented by *u*, oxygen concentration in the input flow by $O_{2}^{in}$ , and the chamber volume is *V*.

### 2.2 Integration of the multilevel mechanistic model with advanced control architectures

**Figure 3:**
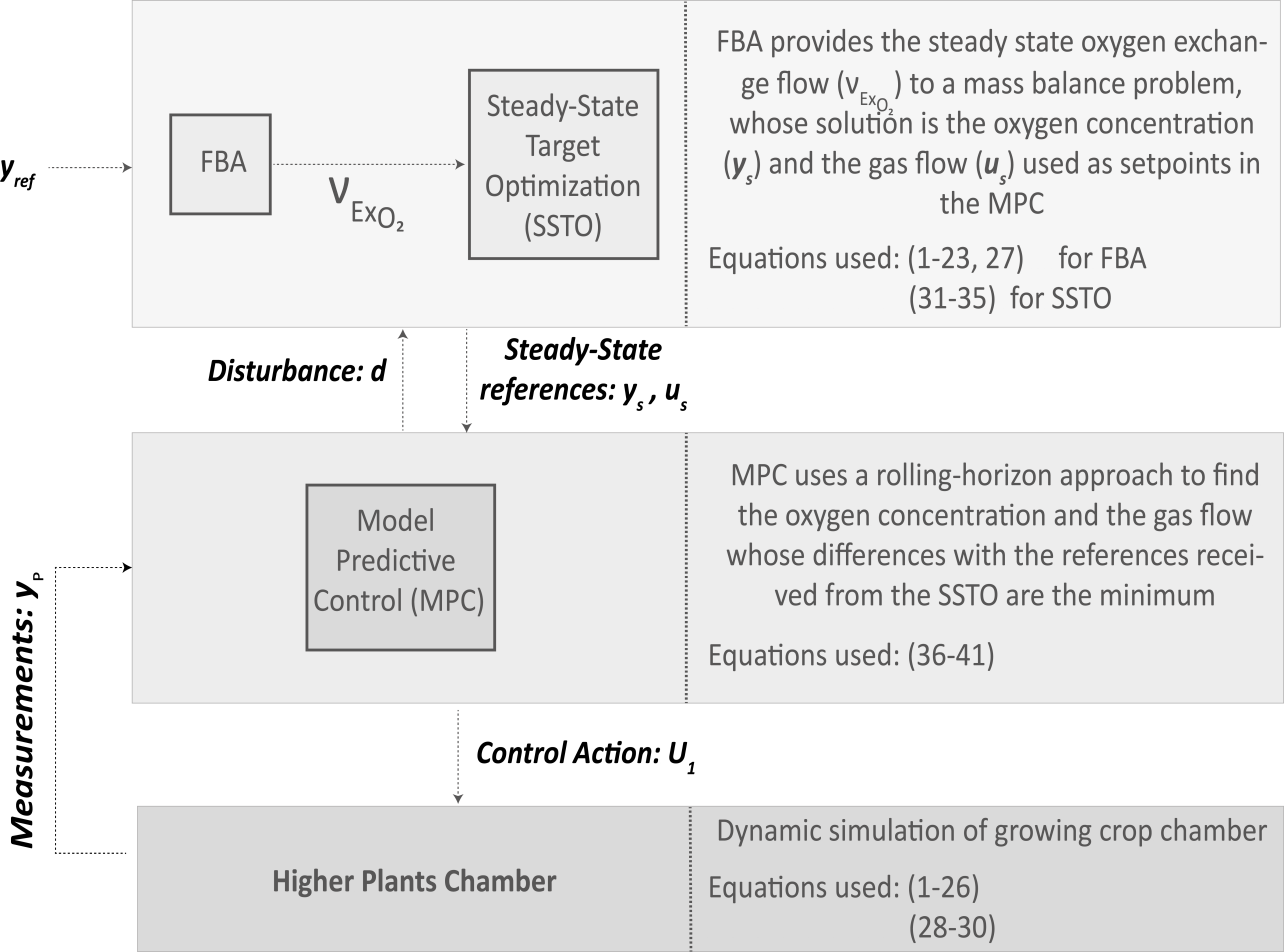
Scheme of the advanced control architecture proposed to integrate metabolic models.

Once having defined the modelling strategy for higher crops, the second scope of this study is to integrate the use of metabolic models into an advanced control strategy. In different complex systems ranging from microgrids (Vasquez et al., 2010), life support (Ciurans et al., 2021), or water distribution systems (Ocampo-Martinez et al., 2012) to chemical plants (Scattolini, 2009; Marchetti et al., 2014), the use of advanced control architectures has been proven to be efficient in terms of optimal management and control. Advanced control architectures are characterized by hierarchically distributing management and control functions in different levels. In this study, an adaptation of a common control architecture used in process plants is adapted to control oxygen in a crop-growing chamber. The top layer called steady-state target optimization (SSTO) aims at finding reference values for the controlled and manipulated variables given a specific setpoint through solving a mass balance problem at steady state. The output of SSTO are the controlled and manipulated variables that give the closest estimation of the controlled variables to the setpoint at steady state. This output becomes the input of the following control step in the hierarchy, which is a model-based predictive control (MPC). MPC uses a discretized model of the process to be controlled and aims at finding the sequence of control commands along a prediction horizon that brings the predicted controlled variables the closest to the reference given a set of hard and soft constraints. MPC works based on a rolling-horizon approach, which essentially solves the minimization problem along the defined prediction horizon but sends solely the control command corresponding to the first step. This process is repeated every time the controller is executed (Pannocchia and Bemporad, 2007). In **Figure 3**, a schematic representation of the advanced control architecture with details on the communication among its levels is presented. In the presented study, the controlled variable is the oxygen concentration in the chamber whereas the manipulated variable is the gas flow.

The SSTO solves the following system of equations using the output of FBA, considering oxygen exchange rate (*v*_*Ex*_*O*_2___ ) as the generation rate in a steady-state mass balance:


(31) $$u_{s}(O_{2}^{in}-x_{s})+v_{Ex_{O_{2}}}=0$$

(32) $$x_{s}+d=y_{s}$$

(33) $$y_{s}=y_{ref}$$

*s.t.*


(34) $$y_{L}\leq y_{s}\leq y_{U}$$

(35) $$u_{L}\leq u_{s}\leq u_{U}$$

As indicated in **Figure 3**, the output of the SSTO is provided as a reference to the MPC, which in this case is the concentration of oxygen in the chamber (*x_s_*) and the external gas flow (*u_s_*) both at steady state. The internal model at steady state is defined in (3), with the generation term (*v*_*Ex*_*O*_2___ ) being the output of the FBA, $O_{2}^{in}$ the input oxygen concentration, and the internal model prediction defined by *x_s_*. A disturbance (*d*) is incorporated in (32) to take into account any possible plant-model mismatch or a measured perturbation and must be taken into account for the new reference generation. Hence, the final prediction value (*y_s_*) at steady state considering the presence of any given disturbance will match the process measurement guaranteeing offset-free control (Pannocchia and Bomperad, 2007). The expression to obtain the disturbance is defined in the MPC development hereafter. The technical upper and lower bounds of controlled and manipulated variables are summarised in (34)–(35) and thus do not need to be defined in the MPC. It is then possible to violate constraints on controlled and measured variables during transition states but not at steady state (Marchetti et al., 2014).

The MPC solves a rolling-horizon non-linear optimization problem, taking the output of the SSTO as the reference to track:


(36) $$J=\underset{U}{min\;}\lambda_{1}\sum_{i=k}^{i=N_{p}}\frac{{\left|\hat{x}\left(i|k\right)-x_{s}\right|}^{2}}{\Delta x}+\lambda_{2}\sum_{i=k}^{i=N_{c}}\frac{{\left|u(i|k)-u_{s}\right|}^{2}}{\Delta u}+\lambda_{3}\sum_{i=k}^{i=N_{c}}\frac{{\left|u(i|k)-u(i-1|k)\right|}^{2}}{\Delta u}$$

*s.t.*


(37) $$\hat{x}(i+1|k)=\hat{x}(i|k)+U(i|k)\left(O_{2}^{in}-\hat{x}(i|k)\right)\frac{T_{s}}{V}+v_{Ex_{O_{2}}}\frac{T_{s}}{V}$$

(38) $$\hat{y}(i|k)=\hat{x}(i|k)+\text{d(}i|k)$$

(39) $$\text{d(}i|k)=y_{p}-\hat{x}(2|k-1)$$

(40) d(i+1|k)=d(i|k)

(41) $$U=\left[\begin{array}{c} U(i|k)\\ \vdots \\ \text{U}(N_{c}|\text{k}) \end{array}\right]$$

The cost function in (36) includes penalization terms to the deviation of the internal prediction ( $\hat{x}$ ) and the control command (*u*) from the concentration (*x_s_*) and the gas flow (*u_s_*) references generated in the SSTO. A third penalization term to the rate of change of the manipulated variable is also included in (36) aimed to adjust the speed of the controller response. All three penalization terms are normalized using the range of possible maximum and minimum values for both controlled and manipulated variables (defined by Δ*x* and Δ*u*) and are subject to a scaling factor (*λ*). Prediction and control horizons are represented by *N_p_* and *N_c_*, respectively. Constraints in (37) reflect the dynamics of the growing crop chamber with *T_s_* as the sample time of the MPC. The internal model is initialized with the current process measurement (*y_p_*). In (39), the disturbance is integrated to the internal model prediction as similarly done in the SSTO in (32). This disturbance is estimated at each sampling time and is defined as the difference between the process measurement (*y_p_*) and the first step prediction of the previous MPC execution ( $\hat{y}\left(2|k-1\right)$ ) as stated in Tatjewski (2017). It is assumed in (40) the disturbance to be constant through the whole prediction horizon. The output matrix of gas flows (*U*) is expressed in (41), and only the control action for the first step of the control horizon is sent to the control actuators until the next SSTO and MPC execution. This control strategy is implemented in Matlab® 2021 using the Optimization Toolbox for solving non-linear programming problems and Cobra Toolbox 2022 for the FBA resolution (Becker et al., 2007).

### 2.3 Simulation scenarios

Two simulation packages are presented: first, the results of the multilevel model presented in Section 2.1 and their validation with experimental data, and second, a dynamic simulation presenting the response of the control architecture presented in Section 2.2 under different perturbations.

#### 2.3.1 Simulation conditions for multilevel model validation

CO_2_-response curves were generated using the fixed light intensity indicated in **Table 4** and the following range of internal CO_2_ values in μmol CO_2_/mol: 100, 225, 300, 450, 600, 850, 1,000, 1,100. Light-response curves were generated using the fixed internal carbon dioxide concentration indicated in **Table 4** and the following range of light intensity values in μmol/m^2^/s: 100, 200, 350, 500, 600, 800, 1,000, 1,200. Considering the conditions listed in **Table 4** and the model Equations (1)–(26), it is possible to retrieve the inequality constraints described in **Table 3** and thus the FBA can be resolved. Results are represented in Section 3.1. The distribution of fluxes is analysed for both light and dark metabolism using atmospheric conditions for CO_2_ which are 400 ppm and a light intensity of 400 µmole/m^2^/s. Results are graphically represented in Section 3.2.

**Table 4:** List of operating parameters used in FBA simulation.

Parameter	Value	Units
Leaf area (LA)	25	m^2^ leaf
Leaf area index (LAI)	5	m^2^ leaf/m^2^ ground
Growing area (A)	5	m^2^ ground
Chamber height (H)	1	m
Bulk temperature (T_b_)	25	°C
Bulk pressure (P_b_)	101,300	Pa
Relative humidity (RH)	70	%
Light intensity (I_u_)	800	μmol/m^2^/s
Internal CO_2_ concentration (C_i_)	400	μmol/mol
Forced velocity (*v_forced_*)	0.3	m s^-1^

#### 2.3.2 Advanced control architecture configuration

In **Table 5**, the controller specifications and parameter values are indicated. On top of the control objective defined in Section 2.2, atmospheric CO_2_ is controlled at 800 ppm with external addition of pure CO_2_.

**Table 5:** Controller specifications including SSTO and MPC algorithms.

Parameter	Value	Units
Input oxygen concentration ( ${\text{O}}_{2}^{\text{in}}$ )	0	%
Oxygen setpoint (y_ref_)	21	%
Carbon dioxide setpoint	800	ppm
Lower and upper bounds oxygen concentration (y_L_ – y_U_)	18-24	%
Lower and upper bound flow ((u_L_ – u_U_))	0-1	m^3^/h
Scaling factor (λ_1_,λ_2_,λ_3_)	10,1,1	
Prediction horizon (N_p_)	4	
Control horizon (N_c_)	3	
Sample time (T_s_)		s

Worthy of note is that the scaling factors are used to promote the control of the system close to the setpoint but at the expense of having a more aggressive control. Prediction and control horizons are important tuning parameters of the controller increasing its sensitivity but also the computational cost of the calculation. Finally, the sampling time also affects the control performance. For slow systems like a plant cultivar, sample times should not be too short because the prediction would not have enough perspective to take correct decisions. Tuning model-based predictive controllers is critical to achieving a good process operation and represents a trade-off between the expected performance and the controller and system capabilities. The metabolic-based control architecture represented in **Figure 3** is tested in a 24-h dynamic simulation using the operating conditions listed in **Table 4**.

## 3 Results

This section is divided into two parts: the first dedicated to present the output of the multilevel model and its validation with experimental data and the second related to the integration of that modelling approach containing metabolic information into an advanced control architecture.

### 3.1 Validating photosynthesis rates

**Figure 4:**
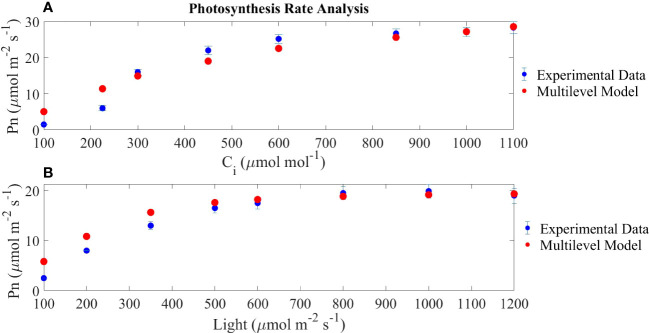
Model validation through the comparison of the photosynthesis rate expressed as net carbon assimilation (Pn) as a function of internal carbon concentration and light irradiance. In **(A)**, a fixed light intensity of 800 μmol/m^2^/s is set; in **(B)**, a fixed internal carbon concentration of 400 μmol/m^2^/s is set. Experimental data are based on Zhou et al. (2020).

The results of the model introduced in Section 2.1 are represented in **Figure 4**, where modelled and experimental results for mature leaves (28 days after transplanting) are compared.

**Figure 4A** represents the RuBisCo saturation curve showing a fast rate of change in the smaller range of internal carbon dioxide concentrations, a pattern which is reproduced in **Figure 4B** which shows the light electron transport chain saturation curve. The maximum net photosynthesis rate achieved in the light-response curve is lower than the maximum achieved in the CO_2_-response curve because in the former, the internal CO_2_ concentration used for the simulation is 400 μmol CO_2_/mol reaching the expected photosynthesis rates if compared to **Figure 4A**. Finally, the output of the model is comparable to the reported experimental results by Zhou et al. (2020) under the same operating conditions. Overall, the error observed in the modelled results in relation to the experimental values is higher at low internal carbon concentrations and at low light intensities.

### 3.2 Distribution of metabolic fluxes using a metabolic diel model

In this section, the flux distribution of day and night metabolites obtained after the resolution of the FBA introduced in (27) are presented. The parameters used for the simulation are those provided in **Table 3**.

#### 3.2.1 Day flux distribution

**Figure 5:**
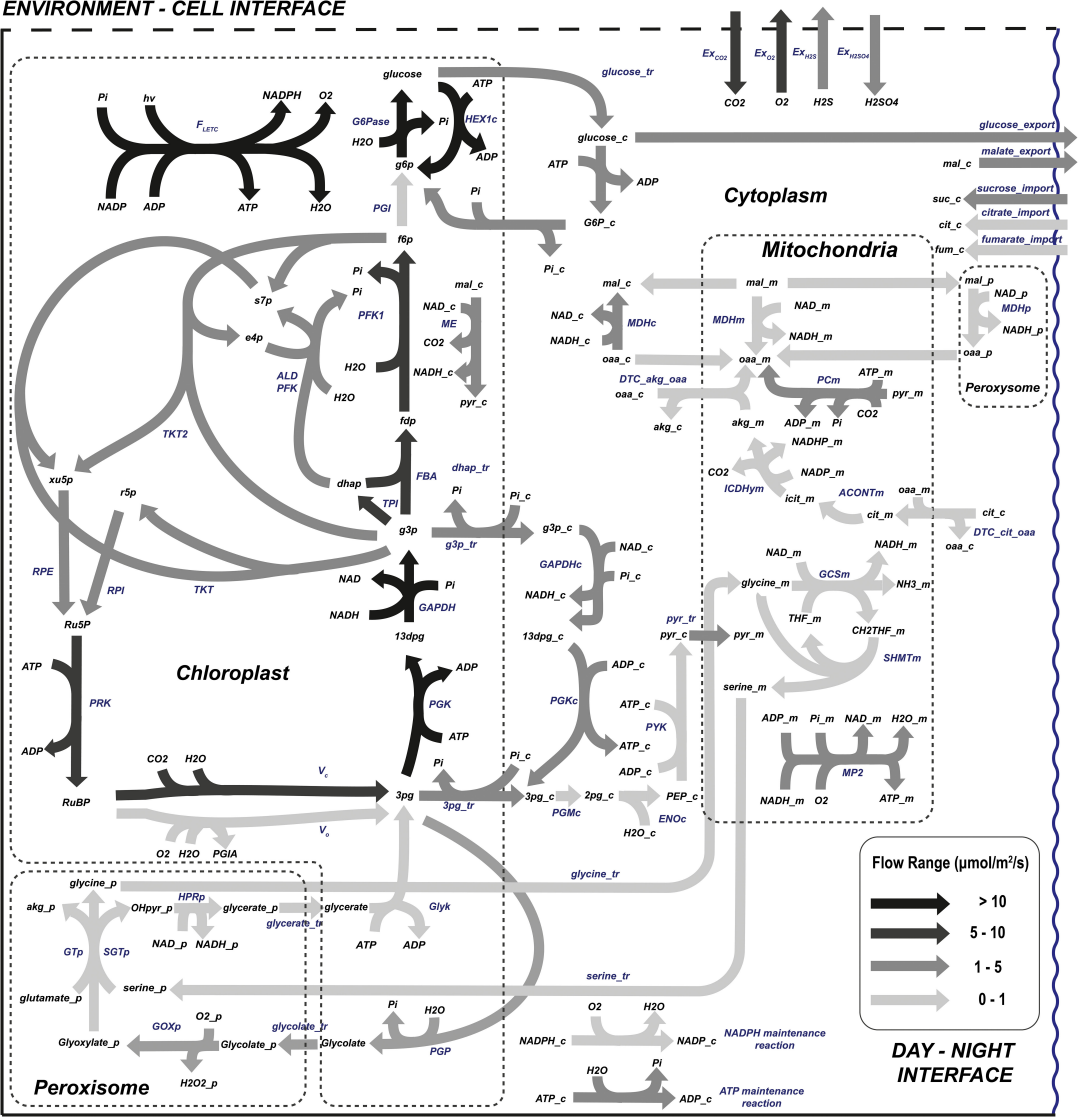
Flow distribution of the central carbon metabolism during *L. sativa* grown during the light photoperiod. List of abbreviated enzymes or enzyme reactions. In chloroplast: F_LETC_, flow of light electron transport chain; G6Pase, glucose-6-phosphatase; PGI, glucose-6-phosphate isomerase; MDH, malate dehydrogenase; FBPase, fructose-1,6-biphosphatase; PFK, phosphofruktokinase-1; ALD, aldolase; TKT, transketolase; TKT2, transketolase 2; FBA, fructose-1,6-biphosphate aldolase; TPI, triose-phosphate isomerase; RPI, ribose-5-phosphate isomerase; RPE, ribulose-phosphate 3-epimerase; GAPDHy, NADP-glyceraldehyde-3-phosphate dehydrogenase; PGK, phosphoglycerate kinase; PRK, phosphoribulokinase; V_c_, RuBisCo carboxylation; V_o_, RuBisCo oxgygenation; Glyk, D-glycerate 3-kinase; PGP, phosphoglycolate phosphatase. In peroxisome: GOXp, glycolate oxidase; SGTp, serine-glyoxylate transaminase; GTp, glycine transaminase; HPRp, hydroxypyruvate reductase; MDHp, malate dehydrogenase. In cytoplasm: PGMc, phosphoglycerate mutase; ENOc, enolase; PYKc, pyruvate kinase; PGKc, phosphoglycerate kinase; GAPDHc, NAD-glycerate-3-phosphate dehydrogenase; MDHc, NADH malate dehydrogenase; ICDHyc, NADP-based isocitrate dehydrogenase; ALDc, aldolase; PFK, phosphofructokinase; TALc, transaldolase. In mitochondria: ACONTm, aconitase; MDHm, malate dehydrogenase; GCSm, glycine cleavage system; SHMTm, serine hydroxymethyltransferase; CSm, citrate synthase; PCm, pyruvate carboxylase; MP2, mitochondrial phosphorylation 2. Carbohydrate reactions represent a set of lumped reactions related to carbohydrate metabolism.

The day flux distribution is presented in **Figure 5**. The central carbon metabolism of plants in light conditions is well represented in this model with the main fluxes located in the Calvin cycle-associated reactions. The results indicate that, as has been extensively studied and published (Michelet et al., 2013; Tan and Cheung, 2020), the flow through the Calvin cycle generates triose phosphate (g3p) from ribulose 1,5-biphosphate (RuBP), consuming part of the reducing power and ATP molecules synthesized during the light electron transport chain. Triose phosphate is used to feed the rest of the Calvin cycle machinery aimed at restoring the ribulose 1,5-biphosphate while it is also partially used to generate photosynthetic end products (McClain and Sharkey, 2019).

As previously stated, higher plants store carbon during the light phase of the day, to be used during night respiration and fuel maintenance processes. For non-starchy crops, even though they can still generate starch, most of the carbon fixed during the light phase is stored as soluble sugars or organic acids. In this study, the sugar molecules stored and mobilized between light and dark periods of the day have not been restricted and, as **Figure 5** suggests, sucrose, fructose, citrate, malate, and fumarate are the metabolites used for carbon exchange. This modelled result fits well with the reported experimental concentrations of sugars in lettuce at harvest, with glucose, sucrose, and fructose being the main carbohydrates found for carbon exchange between phases of the day (Chen et al., 2019) and also predicting with accuracy the role of malate accumulation during the light phase of the day in vacuoles for its use in the dark (Lee et al., 2021). Not all carbon compounds mobilized from light to night metabolism are consumed during the latter. Therefore, some carbon intermediates need to be exported from night to light metabolism too. Specifically, citrate imported from dark periods is used in the light phase of the day to generate oxoglutarate which is important for the nitrogen assimilation mechanism and for the synthesis of nitrogen-rich amino acids. The citrate cycle and its interactions with amino acid biosynthesis are well covered by the presented model, both suffering a flux reduction when nitrate uptake is limited (Morcuende et al., 1998; Weiwei Zhou et al., 2021).

One of the critical phenomena of plant photosynthetic cell metabolism is the coordination of photosynthesis and respiration, which essentially determines how and where energy carrier molecules (ATP and NAD(P)H) are produced. Most of the ATP and NAD(P)H used for catabolic reactions are produced in the light electron transport chain in chloroplasts for amino acid and lipid production. Part of the ATP synthesized in chloroplasts is exported to the cytosol through the 3PG-G3P shuttle, satisfying the ATP demand together with ATP exported from mitochondria (Gakière et al., 2018; Shameer et al., 2019). The resulting metabolic network shows the mechanisms of redox power balancing in the different organelles of photosynthetic leaves enabled by the metabolite shuttles represented by the malate/oxalacetate and the triose phosphate/3-phosphoglycerate shuttle in **Figure 5** (and the glutamate/2-oxoglutarate and the malate/aspartate shuttle, not represented) (Taniguchi and Miyake, 2012). Around half the NAD(P)H generated in mitochondria comes from the glycine decarboxylation, which in turn generates the serine used in the serine-glyoxylate aminotransferase (SGT) in the peroxisome. It has been reported that malate dehydrogenase seems to regulate the reducing power in mitochondria based on the reduction state of the cells, removing and restoring NAD(P)H at low- and high-light conditions responding to changes in the photosynthesis rate (Bykova et al., 2014; Schertl and Braun, 2014). This is validated in the presented fluxome, where mitochondrial malate dehydrogenase (MDHm) removes excess NAD(P)H produced through the glycine cleavage system (GCS, also known as glycine decarboxylase system), the main contributor of redox power in the mitochondria. The metabolic flux distribution represented in **Figure 5** also shows that the TCA cycle is not complete during light photoperiods. This is mainly because most TCA intermediates are dedicated to anabolic reactions and pyruvate dehydrogenase (PDH) is photo-inhibited (Schertl and Braun, 2014).

#### 3.2.2 Night flux distribution

**Figure 6:**
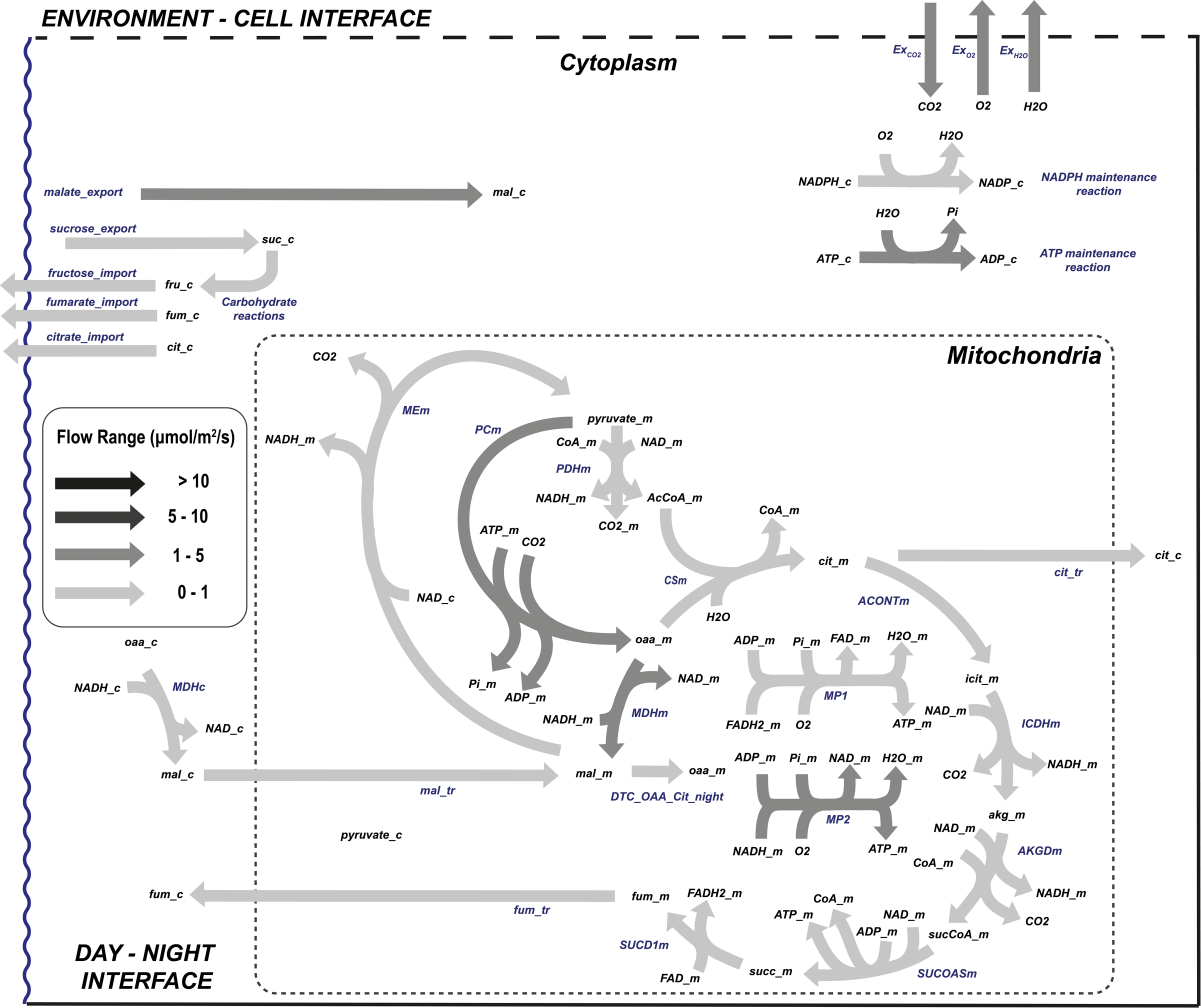
Flow distribution of the central carbon metabolism during *L. sativa* dark phase. List of abbreviated enzymes or enzyme reactions. In cytoplasm: MDHc, malate dehydrogenase; ICDHyc, NADPH isocitrate dehydrogenase; ACONT, aconitase; MEc, malic enzyme; CP1, glucose isomerase; CP2, sucrose-6-phosphate synthase. In mitochondria: ACONTm, aconitase; PDHm, pyruvate dehydrogenase; PCm, pyruvate carboxylase; CSm, citrate synthase; ICDHm, isocitrate dehydrogenase; AKGDm, α-oxoglutarate dehydrogenase; SUCOASm, succinyl-CoA synthetase; SUCD1m, succinate dehydrogenase complex; MDHm, malate dehydrogenase; MP1/2, mitochondrial phosphorylation 1/2.

During the dark phase of the day in **Figure 6**, ATP can only be produced in mitochondria with NAD(P)H being the electron donor and thus completely modifying the flow distribution within the cell. Autotrophic organisms like plants use the sugars generated and stored during the day to feed TCA, which is cyclic in dark conditions. In lettuce and other non-starchy vegetables, instead of mobilizing starch, glucose, sucrose, or fructose is broken down to pyruvate at very similar proportions (Chen et al., 2019) even though in this study the proportion of soluble sugar utilization has not been constrained. The pyruvate produced in the glycolysis is then transferred to the mitochondria to regenerate the reducing power needed to fuel the mitochondrial respiration. In dark conditions, the exchange rates are completely opposite to those observed in light conditions, with carbon dioxide and water being released and oxygen consumed. In a way, respiration and photosynthesis are opposite processes, but complementary as demonstrated during crop growth. The governing rule of night metabolism is represented in **Table 3** in the ratio of consumed CO_2_ during the day phase over produced CO_2_ due to night respiration (Ex_CO2_[d]:Ex_CO2_[n]) which sets the night carbon conversion.

#### 3.2.3 Sensitivity analysis

FBA is a powerful tool but strongly affected by its mathematical formalization and particularly by the flux boundary choice (Raposo et al., 2020; Nobile et al., 2021). A sensitivity analysis on the selected boundaries is very useful for detecting those constraints (either hard or soft constraints) that generate the highest impact on the flux distribution and thus requires a thorough parameter identification. However, sensitivity analysis can also be used to detect those fluxes with the highest variability in relation to a given boundary, metabolic model shortcomings, or fluxes that are invariable to the boundaries. A local sensitivity analysis is presented, with a focus on the flux ratios defined in **Table 3**, which are either defined empirically or based on literature and may be prone to uncertainty. The sensitivity analysis has also been explored for the light irradiance. Details on the ranges of flux ratios explored for this analysis are presented in **Table 6**. The sensitivity analysis has been based on the variability of the fluxes along the range of flux ratios allowed. Such variability is expressed as the normalized summation of the slopes in each of the steps of the range of flux ratios explored:


(42) $$FV_{j,z}=\;\sum_{i=1}^{i=6}\frac{\frac{\nu_{i+1,j,z}-\nu_{i,j,z}}{\text{Δ}r_{z}}}{\text{Δ}v_{j,z}}$$

(43) $$\text{Δ}v_{j,z}=\;\max(\nu_{j,z})-\min(\nu_{j,z})$$

**Table 6:** Range of values explored in sensitivity analysis.

Flux ratios	Nominal ratio	Range	Description
*Ex_O2_[d]: Ex_CO2_[d]*	1.22:1	[-30%, +30%]	Photosynthesis rate
*ATP_maintenance_[n]:NADPH_maintenance_[n]*	3:1	[-30%, +30%]	Maintenance
*ATP_maintenance_[d] :NADPH_maintenance_[d]*	3:1	[-30%, +30%]	Maintenance
*Ex_CO2_[n]:Ex_CO2_[d]*	0.25:1	[-30%, +30%]	Respiration
*I_u_*	400 μmol m^-2^ s^-1^	[-30%, +30%]	Irradiance

The percentage is applied to the nominal ratio (i.e., for the photosynthesis rate ratio, the range explored is from 0.8:1 to 1.59:1). The whole range is split into six points.

**Figure 7:**
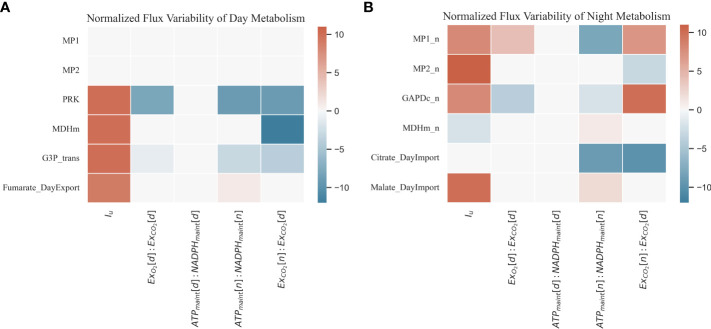
Normalized sensitivity analysis result of a selection of day **(A)** and night **(B)** metabolic fluxes **(B)** according to a given range of ratios and constraints explored.

In (42), the *i* index refers to the discrete steps in which the range of flux ratios has been divided (a total of six steps for each flux ratio) whereas index *j* and *z* refer to the reaction and the flux ratio, respectively. The parameter Δ*r_z_* is the increment in the flux ratio expressed as a fraction and equivalent to 0.1 for all cases. The denominator Δ*v_j,z_* is used to normalize the summation of slopes, and it represents the range of values for a given reaction *j*. The sensitivity analysis has been carried for light and night metabolism and is represented in **Figure 7** considering an atmospheric concentration of CO_2_ of 1,000 ppm in order not to limit by carbon substrate and a nominal light intensity of 400 µmol/m^2^/s.

Considering the light metabolism sensitivity analysis (**Figure 7A**), the light phase of day-time mitochondrial respiration is never active. In the predicted fluxome for all ranges of flux ratios applied, the ATP production comes mainly from chloroplast activity. The main mitochondrial NADH producer in light conditions is the GDC system, being consumed mainly by the malate dehydrogenase activity and not by the electron transport chain (Bykova et al., 2014). The lack of the electron transport chain in day metabolism is due to the lack of maintenance reactions during the light period. When light is increased, the reactions associated with the regeneration of RuBP are activated. This is the case of phosphoribulokinase (PRK), which restores the ribulose 1,5-biphosphate. An increase in the triose phosphate export activity (G3P_trans) is also detected responding to the increase in the carbon fixation. The mitochondrial malate dehydrogenase (MDHm) is also augmented in the first place to respond to the increased anabolic demands and in the second place given the increased flux through photorespiration that triggers the GCS and the consequent increased consumption of NADH through MDHm to keep the redox balance in mitochondria (Schertl and Braun, 2014). The sensitivity analysis of the day metabolism also highlights that, when the night respiration flux in relation to the daily carbon fixation is increased relative to the nominal value of 0.25, the associated photosynthetic reactions (PRK and G3P_trans) are reduced.

Regarding night metabolism (**Figure 7B**), the variability of the represented reactions is higher than in day metabolism. When light intensity (I_u_) is increased, night mitochondrial activity (MP1_n and MP2_n) is also increased to respond to the maintenance reaction demands (Frantz and Bugbee, 2005). The activity of glyceraldehyde 3-phosphate dehydrogenase (GAPDc_n) responds to the increased flux towards glycolytic pathways to process the carbon compounds converted during night metabolic activities (Schneider et al., 2018; Gaude et al., 2018). In the dark phase, with the cyclic TCA cycle re-stored, the excess TCA intermediates and soluble sugars not used to fuel dark metabolism are stored and reused in light metabolism for nitrogen assimilation and biosynthesis reactions (Popova and Pinheiro De Carvalho, 1998; Igamberdiev and Eprintsev, 2016). In this way, the pool of organic carbon compounds and reducing equivalents is managed by the plants as a response to variations in the day carbon fixation efficiency and night respiration activities. GAPDc_n is also positively affected when the ratio of respiratory produced over photosynthetically consumed CO_2_ is increased (Ex_CO2_[n]:Ex_CO2_[d]) with the availability of citrate and the usage of other TCA intermediates in light photoperiods reduced given the night metabolic increased activities as suggested by the citrate import flux. Therefore, an increased night CO_2_ release is corresponded by an increased glycolytic activity represented by GAPDC_n, also impacting the TCA intermediates and soluble sugar accumulation at night.

### 3.3 Testing the integration of multilevel model approach to advanced control architectures in dynamic simulations

The control strategy presented based on a new approach combining the prediction capacity of MPC and the integration of constraint-based metabolic modelling has been tested in a 24-h dynamic simulation. To do that, different perturbations have been included at different points of the simulation to test the resiliency of the proposed control approach its capacity to overcome common control challenges and to analyse its versatility:

- At 5 h of simulation time, a plant-model mismatch has been introduced by adding a perturbation in the form of a multiplication factor to the gas flows in (31):
(44) $$2u_{s}(O_{2}^{in}-x_{s})+v_{Ex_{O_{2}}}=0$$
(45) $$\hat{x}(i+1|k)=\hat{x}(i|k)+2U(i|k)\left(O_{2}^{in}-\hat{x}(i|k)\right)\frac{T_{s}}{V}+v_{Ex_{O_{2}}}\frac{T_{s}}{V}$$
• At 10 h of simulation time, a perturbation in the process output has been introduced by adding a sudden decrease in the oxygen concentration from the measurement to 20.8%.
• At 16 h of simulation time, a change in the oxygen setpoint from 21% to 21.2% has been included.

**Figure 8:**
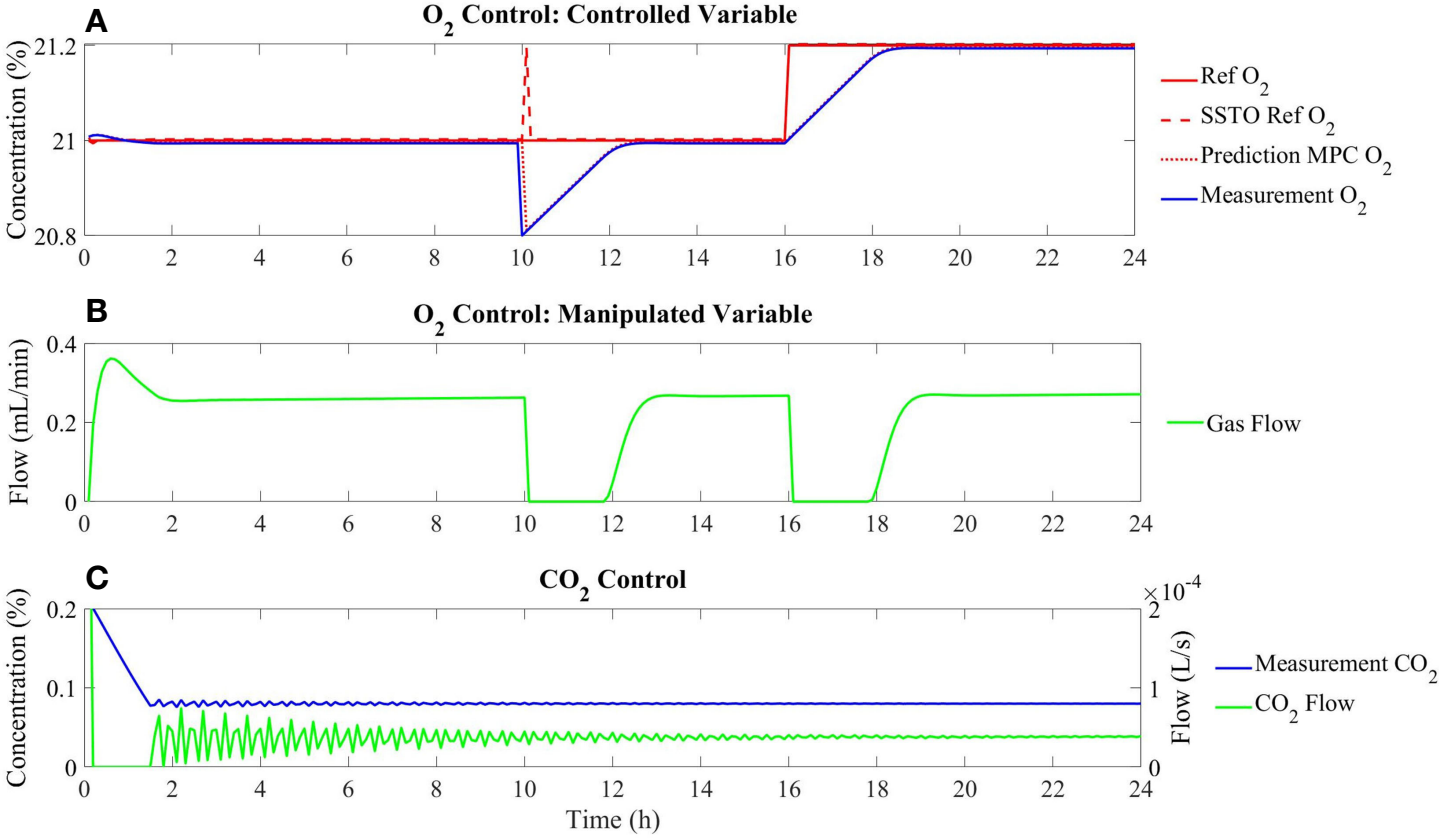
Dynamic control performance under introducing different perturbations at 5, 10, and 16 hours as described in the text. **(A)** Tracking of the Oxygen concentration; **(B)** Evolution of the gas flow as a manipulated variable; **(C)** Dynamics of concentration and external addition of Carbon dioxide.

The results of this simulation schedule are presented in **Figure 8**. It is observed that when a plant-model mismatch is deliberately included, the controller can keep minimising the offset of the O_2_ measurement in relation to the reference (**Figure 8A**). Similarly, when an abrupt perturbation is added at 10 h of simulation, the gas flow is stopped to restore the oxygen concentration rapidly. Finally, when the reference is modified from 21% to 21.2%, the gas flow is also reduced in order to accumulate oxygen in the growing chamber and reduce the tracking error (**Figure 8B**). The control of CO_2_ is achieved by externally injection of pure CO_2_ (**Figure 8C**).

**Figure 9:**
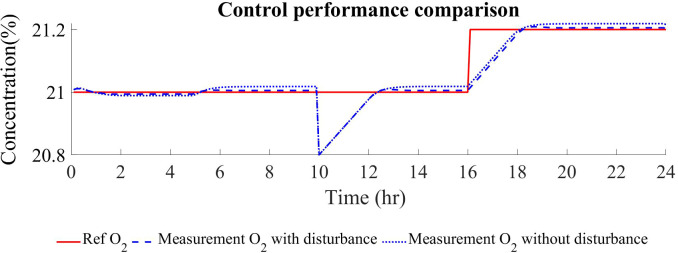
Comparison of the controller performance in terms of reference tracking between controllers including or not disturbance rejection in the internal SSTO and MPC models.

The controller is thus demonstrated to be resilient and smooth to overcome any of the perturbations applied as well as on the nominal operation. This performance is due to the reliable metabolic-based model introduced in the MPC and largely due to the integration of a disturbance specially to achieve offset-free control. In **Figure 9**, the comparison of the controller performance with and without disturbance integration is represented. It can be observed that under different perturbations, when disturbance is integrated in the internal model of both the SSTO and the MPC, offset is reduced and thus the reference tracking is improved. Nevertheless, not all disturbances can be rejected using a constant state disturbance prediction as defined in (32), especially in scenarios where plant-model mismatches are bigger, that is, when models are less reliable than the one presented in this study. In the case of higher model disagreement, disturbance integration approaches should be considered like integrating the error in the multistep model prediction (Tian et al., 2007) or integrating a moving horizon estimation (MHE) and MPC to estimate uncertainty parameters and to include them in the MPC algorithm (Huang et al., 2010). Disturbance rejection especially in the situation of plant-model mismatch has been highly analysed within MPC development, and certainly, the future metabolic-based controllers will need to deal with a wide diversity of model typologies from simplified and surrogated models to complex genome-scale metabolic models.

## 4 Conclusions

Modelling higher plants has major challenges to deal with. These challenges include huge metabolic changes associated with the light and night photoperiods, substrate partitioning given the heterogenic requirements of the different tissues present in higher plants, organelle coordination, complex morphologies that condition the interaction with the environment, and many other phenomena still not fully understood. In this study, a multilevel model has been designed with the main mechanistic phenomena that drive crop growth distributed into different levels in decreasing order of scale length. The output of the mechanistic multilevel model has been connected to a constraint-based metabolic model providing information of high interest about cell metabolism. The presented multilevel model offers the advantage to merge all the available information related to plant growth in a structured way ensuring the solution found to be feasible for all the phenomena described in the different layers in the model hierarchy, from light reception and biochemical conversion down to specific metabolic pathways, a feasibility that cannot be granted in only mechanistic or metabolic-based models using stand-alone modelling strategies. This method has been validated with experimental data, integrated, and tested in a novel advanced control strategy with promising results. Computational capabilities are no longer a major constraint, making it possible to contemplate the design of control strategies that can integrate more information about the system under operation than currently done. In this study, the focus has been placed in the use of metabolic information embedded in a model-predictive control providing promising results in a dynamic simulation of a growing crop chamber with a range of applications going from agriculture to life support systems. It is in the framework of the MELiSSA project and by extension to the field of fully regenerative life support systems where structural multilevel models, integrating from physical to metabolic information, emerges as an opportunity to design model-based predictive controller techniques. These advanced control strategies should be further explored with different levels of metabolic complexity, in different control formulations, and applied to different biobased processes as they may contribute to improving the overall performance through exploiting the increasingly available metabolic information.

## Data availability statement

The datasets presented in this study can be found in online repositories. The names of the repository/repositories and accession number(s) can be found in the article/**Supplementary Material**.

## Author contributions

CC is the main writer of the manuscript as well as the one responsible for script development, simulation, and main analysis and interpretation of the results. IM and IMdM are the main contributors to the constraint-based metabolic modelling. IMdM contributed to the design, validation, and interpretation of the results. JM is the main contributor and is responsible for the design of a control architecture adapted to metabolic networks. CG and FG have contributed to the design of the mechanistic multilevel model, specially concerning the physical and enzymatic equations, and they have supervised the overall text. CG is the responsible of the original lettuce metabolic model development. All authors contributed to the article and approved the submitted version.

## Funding

MELiSSA is an international consortium of 15 partners led by the European Space Agency. Its activities are governed by a Memorandum of Understanding (ESA 4000100293/10/NL/PA). The MELiSSA Pilot Plant is funded from ESA contributions from Spain (main contributor), Belgium, France, Italy, and Norway, under Frame Contract C4000109802/13/NL/CP. Cofunding from Ministerio de Ciencia, Innovación y Universidades, Generalitat de Catalunya, and Universitat Autònoma de Barcelona is also acknowledged. Carles Ciurans is a PhD fellow from the POMP Program (MELiSSA Foundation) of ESA. This work was supported by VILLUM FONDEN under the VILLUM Investigator Grant (no. 25920): Center for Research on Microgrids (CROM). Igor Marin de Mas and Ivan Martínez-Mongue would like to thank The Novo Nordisk Foundation (NNF Grant numbers: NNF10CC1016517 and NNF14OC0009473).

## Acknowledgments

The authors wish to thank the partners of the MELiSSA Consortium for carefully reviewing this publication.

## Conflict of interest

Author IMdM and IM are employed by Novo Nordisk Foundation Center for Sustainability.

The remaining authors declare that the research was conducted in the absence of any commercial or financial relationships that could be construed as a potential conflict of interest.

## Publisher’s note

All claims expressed in this article are solely those of the authors and do not necessarily represent those of their affiliated organizations, or those of the publisher, the editors and the reviewers. Any product that may be evaluated in this article, or claim that may be made by its manufacturer, is not guaranteed or endorsed by the publisher.
