# Supplementary material for: Enhancing control systems of higher plant culture chambers via multilevel structural mechanistic modelling
Source: Front Plant Sci. 2022 Oct 20;13:970410. doi: 10.3389/fpls.2022.970410 (PMC9632494; doi:10.3389/fpls.2022.970410)
Supplement: Supplementary file 1 [file Table_1.docx]

Supplementary Material 1: Boundary Layer Model

# Supplementary Data: Boundary Layer Model (Equation indexes are a continuation of the manuscript indexes)

# *Morphological module*

Leaf area ($LA$) is expressed as a function of biomass accumulated following a logistic approximation:

| $LA=3(1+e^{(220-\frac{0.016M_{x}}{DM})})$ | (46) |
| --- | --- |
|  |  |

Being $DM$ the dry mass ratio fraction and $M_{x}$ the accumulated dry biomass. Leaf length ($L$) is approximated considering a circular shape:

| $L=2\sqrt{\frac{LA}{\pi}}$ | (47) |
| --- | --- |

*Biochemical module*

According to Harley et al. (1992), the temperature dependence of $F_{LETC}^{max}$ and $V_{cmax}$is described by the following expressions:

| $F_{LETC}^{max}=F_{LETC}^{max25}\frac{exp[\left( 3.3621\cdot{10}^{-3}T_{l}-1 \right)E/(RT_{l})]}{1+exp[(ST_{l}-H')/(RT_{l})]}$ | (48) |
| --- | --- |
| $V_{cmax}=V_{cmax25}\frac{exp[46.9411-116300/(RT_{l})]}{1+exp[(650T_{l}-202900)/(RT_{l})]}$ | (49) |
| $V_{omax}=V_{cmax}\frac{k_{o}}{k_{c}}$ | (50) |

In (49) $k_{c}$ is the turnover number of carboxylase sites, which in this model a value of 2.5 s^-1^ has been used as suggested by Farquhar et al. (1980). At 25ºC the value of $k_{o}$, the equivalent of $k_{c}$ for oxygenase sites is 0.21 times that of $k_{c}$.

*Gas exchange module*

In (18)-(20) the conductance of the different gases through the leaves is introduced. Two layers are considered to calculate the leaf conductance for a given gas *z (*$G^{z}$*)*, the boundary layer ($g_{BL}^{z}$) and the stomatal conductance ($g_{s}^{z}$). Considering an electrical resistance analogy for each of these layers, the following expression for calculating the overall leaf conductance can be used:

| $G^{z}=\frac{g_{BL}^{z}{\cdot g}_{s}^{z}}{g_{BL}^{z}{+g}_{s}^{z}}$ | (51) |
| --- | --- |

The conductance through the boundary layer depends on the diffusion coefficient ($D^{z}$), which is species-specific:

| $g_{BL}^{z}=\frac{D^{z}P_{b}}{\delta\cdot R\cdot T_{bl}}$ | (52) |
| --- | --- |

In (52), $P_{b}$ represents the bulk pressure, $T_{bl}$ is the average temperature between the bulk air and the leaves, R is the ideal gas constant and $\delta$ the boundary layer thickness.

The diffusion coefficient for the different gases uses depend on the environmental conditions in the following way:

| $D^{H_{2}O}=0.242\frac{{Tb/293}^{3/2}}{0.1P_{b}}$ | (53) |
| --- | --- |
| $D^{{CO}_{2}}=0.177\frac{{Tb/317}^{3/2}}{0.1P_{b}}$ | (54) |
| $D^{O_{2}}=0.176\frac{{Tb/298}^{3/2}}{0.1P_{b}}$ | (55) |
| $D^{t}=0.2207\frac{{Tb/300}^{1.81}}{0.1P_{b}}$ | (56) |

In (51), the conductance through the stomata for each of the gas species is proportional to the conductance of water:

| $g_{s}^{{CO}_{2}}=g_{s}^{H_{2}O}\frac{D^{{CO}_{2}}}{D^{H_{2}O}}$ | (57) |
| --- | --- |
| $g_{s}^{O_{2}}=g_{s}^{H_{2}O}\frac{D^{O_{2}}}{D^{H_{2}O}}$ | (58) |

The boundary layer thickness $\delta$ also depends on the conditions of the bulk air, more specifically on the air density and the bulk velocity:

| $\delta=2\sqrt{\frac{L\cdot\eta}{v_{bulk}}}$ | (59) |
| --- | --- |
| $v_{bulk}=\sin\left( \alpha\right)\cdot v_{forced}+\cos\left( \alpha\right)\cdot v_{free}$ | (60) |
| $v_{free}=\sqrt{2\cdot g\cdot H\cdot\Delta_{\rho}}$ | (61) |
| $\Delta_{\rho}=\vert\frac{\rho_{b}-\rho_{l}}{\rho_{b}}\vert$ | (62) |

In (59)-(62), $\eta$ is the air kinematic viscosity, $v_{bulk}$, $v_{forced}$ and $v_{free}$ are the resulting bulk velocity parallel to the leaf surface, the free convection velocity and the forced convection velocity respectively, $g$ is the gravity force, $H$ is the chamber height and $\alpha$ is the leaf inclination in relation to the vertical direction. The leaves air density ($\rho_{l}$) depends on gas species concentrations in the following way:

| $\rho_{l}=\frac{P_{l}^{{CO}_{2}}\cdot{MW}_{{CO}_{2}}+P_{l}^{O_{2}}\cdot{MW}_{O_{2}}+P_{l}^{H_{2}O}\cdot{MW}_{H_{2}O}+P_{l}^{N_{2}}\cdot{MW}_{N_{2}}}{R\cdot T_{l}}$ | (63) |
| --- | --- |

Finally, the internal concentration of the different species can be derived from (18)-(20):

| $P_{l}^{{CO}_{2}}=P_{b}^{{CO}_{2}}-\frac{P_{b}}{LA\cdot G_{CO_{2}}}Pn$ | (64) |
| --- | --- |
| $P_{l}^{O_{2}}=P_{b}^{O_{2}}+\frac{P_{b}}{LA{\cdot G}_{O_{2}}}Pn$ | (65) |
| $P_{l}^{H_{2}O}=P_{b}^{H_{2}O}+\frac{P_{b}}{LA{\cdot G}_{H_{2}O}}{Ex}_{H_{2}O}$ | (66) |

For the sake of clarity, the main difference between the classical way of solving plant photosynthesis model and the methodology presented in this study is on the way to obtain the net photosynthesis ($P_{n}$). As stated in (26)-(27) the net photosynthesis is usually obtained by finding the phenomena that limits the photosynthesis and can either be due to a biochemical machinery limitation ($J,V_{c}$) or by a mass transfer limitation (${Ex}_{{CO}_{2}}$):

| $P_{g}=min\left( V_{c}\cdot LA,{Ex}_{{CO}_{2}}\cdot LA,J\cdot A \right)$ | (67) |
| --- | --- |
| $P_{n}=P_{g}\left( 1-R_{d} \right)$ | (68) |

The multilevel modelling approach presented provides the net photosynthesis directly from the resolution of the FBA:

| $P_{n}=\upsilon_{{Ex}_{O_{2}}}$ | (69) |
| --- | --- |

# Supplementary Data: Model nomenclature

| **Symbol** | **Description** | **Units** |
| --- | --- | --- |
| ***Morphological module*** | | |
| $LA$ | Leaf area | m^2^ leaves |
| $L$ | Leaf length | m leaves |
| **Irradiance module** | | |
| $I$ | Irradiance | moles m^-2^ ground s^-1^ |
| $I_{u}$ | Irradiance at LAI = 0 | moles m^-2^ ground s^-1^ |
| $I_{s,g}$ | Direct irradiance over ground surface | moles m^-2^ ground s^-1^ |
| $I_{d,g}$ | Diffuse irradiance over ground surface | moles m^-2^ ground s^-1^ |
| $I_{s}$ | Direct irradiance over leaf surface | moles m^-2^ leaves s^-1^ |
| $I_{d}$ | Diffuse irradiance over leaf surface | moles m^-2^ leaves s^-1^ |
| $LAI$ | Leaf area index | m^2^ leaves m^-2^ ground |
| ${LAI}_{s}$ | LAI exposed to direct irradiation | m^2^ leaves m^-2^ ground |
| ${LAI}_{d}$ | LAI exposed to diffuse irradiation | m^2^ leaves m^-2^ ground |
| ***Energy Balance*** | | |
| $T_{l}$ | Leaf temperature | K |
| $k_{t}$ | Heat transfer coefficient | m s^-1^ |
| $E_{hs}$ | Direct irradiance energy | J s^-1^ |
| $E_{hd}$ | Diffuse irradiance energy | J s^-1^ |
| $E_{r}$ | Radiation energy | J s^-1^ |
| $E_{conv}$ | Convection energy | J s^-1^ |
| $E_{tr}$ | Transpiration energy | J s^-1^ |
| **Gas Exchange** | | |
| ${Ex}_{{CO}_{2}}$ | CO_2_ exchange rate | moles m^-2^ leaves s^-1^ |
| ${Ex}_{O_{2}}$ | O_2_ exchange rate | moles m^-2^ leaves s^-1^ |
| ${Ex}_{H_{2}O}$ | H_2_O exchange (transpiration) rate | moles m^-2^ leaves s^-1^ |
| $G_{z}$ | Conductance compound z | moles m^-2^ leaves s^-1^ |
| $P_{b}^{z}$ | Bulk partial pressure compound z | Pa |
| $P_{l}^{z}$ | Leaf partial pressure compound z | Pa |
| **Biochemical module** | | |
| $F_{LETC}$ | Light electron transport chain rate | moles m^-2^ leaves s^-1^ |
| $F_{LETC}^{max}$ | Maximum light electron transport chain rate | moles m^-2^ leaves s^-1^ |
| $J$ | Ribulose 1,5-biphosphate regeneration | moles m^-2^ leaves s^-1^ |
| $\Gamma$ | Carbon dioxide compensation point | moles m^-3^ |
| $V_{c}$ | Carboxylation rate | moles m^-2^ leaves s^-1^ |
| $V_{cmax}$ | Maximum carboxylation rate | moles m^-2^ leaves s^-1^ |
| $V_{o}$ | Oxygenation rate | moles m^-2^ leaves s^-1^ |
| $C_{l}$ | Carbon dioxide leaf concentration | moles m^-3^ |
| $O_{l}$ | Oxygen leaf concentration | moles m^-3^ |
| $P_{g}$ | Gross photosynthesis rate | moles m^-2^ leaves s^-1^ |
| $P_{n}$ | Net photosynthesis rate | moles m^-2^ leaves s^-1^ |
| **Boundary layer** | | |
| $g_{BL}^{z}$ | Boundary layer conductance of compound z | moles m^-2^ leavess^-1^ |
| $g_{s}^{z}$ | Stomatal conductance of compound z | moles m^-2^ leaves s^-1^ |
| $\delta$ | Boundary layer thickness | m |
| $T_{bl}$ | Average leaf-bulk temperature | K |
| $D^{z}$ | Diffusion coefficient of compound z | m^2^ s^-1^ |
| $v_{bulk}$ | Bulk velocity | m s^-1^ |
| $v_{free}$ | Free velocity | m s^-1^ |
| $\rho_{l}$ | Leaf air density | kg m^-3^ |

# Supplementary Data: Constant Parameters

| **Symbol** | **Description** | **Units** |
| --- | --- | --- |
| **Irradiance module** | | |
| $k$ | Extinction coefficient (0.5) |  |
| $f_{s}$ | Direct irradiance fraction (0.7) |  |
| $BCmol$ | C-mole molecular weight (27) | g mol-C^-1^ |
| ***Energy Balance*** | | |
| Na | Avogadro number (6.02·10^23^) | Pa |
| c | Light velocity (3·10^9^) | Pa |
| $h$ | Planck constant (6.63·10^-34^) | m^2^ kg s^-1^ |
| $\gamma$ | Wavelength fraction |  |
| $\lambda$ | Wavelength | nm |
| $\varepsilon$ | Leaf emissivity (0.97) |  |
| σ | Stefan-Boltzman constant (5.67·10^-8^) | J s^-1^ K^-4^ |
| $T_{b}$ | Bulk temperature (293 - 299) | K |
| $P_{b}$ | Bulk pressure (101300) | Pa |
| $R$ | Ideal gas constant (8.314) | m^3^ Pa K^-^1 mol^-1^ |
| $C_{p}$ | Molar air specific heat capacity (29.3) | J mol^-1^ K^-1^ |
| $\lambda_{mol}$ | Water latent heat of vaporization (4.0788·10^4^) | J mol^-1^ |
| **Biochemical module** | | |
| $\theta$ | Convexity coefficient (0.8) |  |
| $\varphi$ | Efficiency of energy conversion for LETC |  |
| $K_{c}$ | Carboxylation half-saturation constant | moles m^-3^ |
| $K_{o}$ | Oxygenation half-saturation constant | moles m^-3^ |
| ${K_{c}}_{25}$ | $K_{c}$ at 25ºC (0.66) | moles m^-3^ |
| ${K_{o}}_{25}$ | $K_{o}$ at 25ºC (1.0113·10^3^) | moles m^-3^ |
| $F_{LETC}^{max25}$ | $F_{LETC}^{max}$ at 25ºC | μmol m^-2^ s^-1^ |
| ${V_{cmax}}_{25}$ | $V_{cmax}$ at 25ºC | moles m^-2^ leaves s^-1^ |
| $E$ | Activation energy of reaction (81993) | J mol^-1^ K^-1^ |
| $S$ | Entropy (711.36) | J mol^-1^ K^-1^ |
| $H'$ | Energy of deactivation (219814) | J mol^-1^ K^-1^ |
| $M_{c}$ | C-molar molecular weight (27) | g mol-C^-1^ |
| $DM$ | Dry Biomass fraction (0.045) | g/g |
| **Boundary layer** | | |
| $\eta$ | Air kinematic viscosity (1.8·10^-5^) | m^2^ s^-1^ |
| $v_{forced}$ | Forced velocity (0.3) | m s^-1^ |
| $\alpha$ | Leaf angle in relation to the vertical axis (0.1) | º |
| $g$ | Gravity force (9.8) | m s^-2^ |
| $H$ | Chamber height (1) | m |
| $\rho_{b}$ | Bulk air density (1.1866) | kg m^-3^ |
