# Supplementary material for: Enhancing control systems of higher plant culture chambers via multilevel structural mechanistic modelling
Source: Front Plant Sci. 2022 Oct 20;13:970410. doi: 10.3389/fpls.2022.970410 (PMC9632494; doi:10.3389/fpls.2022.970410)
Supplement: Supplementary file 3 [file Table_3.docx]

Supplementary Material 2: Summary of Metabolic Reactions

| - **Compartment** | - **Pathway** | - **Number of Reactions** |
| --- | --- | --- |
| - *Chloroplast* | - Amino Acid Synthesis | - 38 |
|  | - Calvin Cycle | - 17 |
|  | - Connecting Reactions | - 4 |
|  | - Exchange | - 1 |
|  | - Folate Cycle | - 1 |
|  | - Glutathione Cycle | - 2 |
|  | - Glycolysis | - 5 |
|  | - Light ETC | - 1 |
|  | - Lipid Synthesis | - 3 |
|  | - Malic Enzyme | - 2 |
|  | - Nitrogen Assimilation | - 5 |
|  | - Pentose Phosphate Pathway | - 2 |
|  | - Photorespiration | - 2 |
|  | - Sulphate Cycle | - 3 |
|  | - Transport | - 38 |
| - *Mitochondria* | - Amino Acid Synthesis | - 3 |
|  | - Connecting Reaction | - 6 |
|  | - Folate Cycle | - 6 |
|  | - Lipid Synthesis | - 2 |
|  | - Malic Enzyme | - 1 |
|  | - Nitrogen Assimilation | - 2 |
|  | - Respiratory ETC | - 2 |
|  | - Transport | - 43 |
| - *Cytoplasm* | - Amino Acid Synthesis | - 8 |
|  | - Calvin Cycle | - 5 |
|  | - Carbohydrate Synthesis | - 7 |
|  | - Connecting Reaction | - 10 |
|  | - Exchange | - 9 |
|  | - Folate Cycle | - 6 |
|  | - Glycolysis | - 11 |
|  | - Lipid Synthesis | - 17 |
|  | - Malic Enzyme | - 2 |
|  | - Nitrogen Assimilation | - 3 |
|  | - Pentose Phosphate Pathway | - 2 |
|  | - Photoperiod Exchange | - 7 |
| - *Peroxisome* | - Photorespiration | - 5 |
|  | - Transport | - 4 |
