# Supplementary material for: Enhancing control systems of higher plant culture chambers via multilevel structural mechanistic modelling
Source: Front Plant Sci. 2022 Oct 20;13:970410. doi: 10.3389/fpls.2022.970410 (PMC9632494; doi:10.3389/fpls.2022.970410)
Supplement: Supplementary file 4 [file Table_4.docx]

<?xml version="1.0" encoding="UTF-8"?>

<sbml xmlns="http://www.sbml.org/sbml/level3/version1/core" xmlns:fbc="http://www.sbml.org/sbml/level3/version1/fbc/version2" xmlns:groups="http://www.sbml.org/sbml/level3/version1/groups/version1" level="3" version="1" fbc:required="false" groups:required="false">

<model id="COBRAModel" name="Model Exported from COBRA Toolbox" fbc:strict="true">

<listOfUnitDefinitions>

<unitDefinition id="mmol_per_gDW_per_hr">

<listOfUnits>

<unit kind="mole" exponent="1" scale="-3" multiplier="1"/>

<unit kind="gram" exponent="-1" scale="0" multiplier="1"/>

<unit kind="second" exponent="-1" scale="0" multiplier="3600"/>

</listOfUnits>

</unitDefinition>

</listOfUnitDefinitions>

<listOfCompartments>

<compartment metaid="c" id="c" name="Cytoplasm" constant="false"/>

<compartment metaid="d" id="d" name="unknownCompartment2" constant="false"/>

<compartment metaid="m" id="m" name="Mitochondrion" constant="false"/>

<compartment metaid="n" id="n" name="Nucleus" constant="false"/>

<compartment metaid="p" id="p" name="Periplasm" constant="false"/>

</listOfCompartments>

<listOfSpecies>

<species metaid="M_10_FormylTHF__91__d__93__" id="M_10_FormylTHF__91__d__93__" name="10_FormylTHF[d]" compartment="d" hasOnlySubstanceUnits="false" boundaryCondition="false" constant="false"/>

<species metaid="M_10_FormylTHF__91__n__93__" id="M_10_FormylTHF__91__n__93__" name="10_FormylTHF[n]" compartment="n" hasOnlySubstanceUnits="false" boundaryCondition="false" constant="false"/>

<species metaid="M_1_2Oxoisovalerate__91__d__93____91__c__93__" id="M_1_2Oxoisovalerate__91__d__93____91__c__93__" name="1_2Oxoisovalerate[d][c]" compartment="c" hasOnlySubstanceUnits="false" boundaryCondition="false" constant="false"/>

<species metaid="M_1_2Oxoisovalerate__91__n__93____91__c__93__" id="M_1_2Oxoisovalerate__91__n__93____91__c__93__" name="1_2Oxoisovalerate[n][c]" compartment="c" hasOnlySubstanceUnits="false" boundaryCondition="false" constant="false"/>

<species metaid="M_1_3BPGA__91__d__93__" id="M_1_3BPGA__91__d__93__" name="1_3BPGA[d]" compartment="d" hasOnlySubstanceUnits="false" boundaryCondition="false" constant="false"/>

<species metaid="M_1_3BPGA__91__d__93____91__c__93__" id="M_1_3BPGA__91__d__93____91__c__93__" name="1_3BPGA[d][c]" compartment="c" hasOnlySubstanceUnits="false" boundaryCondition="false" constant="false"/>

<species metaid="M_1_3BPGA__91__n__93__" id="M_1_3BPGA__91__n__93__" name="1_3BPGA[n]" compartment="n" hasOnlySubstanceUnits="false" boundaryCondition="false" constant="false"/>

<species metaid="M_1_3BPGA__91__n__93____91__c__93__" id="M_1_3BPGA__91__n__93____91__c__93__" name="1_3BPGA[n][c]" compartment="c" hasOnlySubstanceUnits="false" boundaryCondition="false" constant="false"/>

<species metaid="M_1_pyrroline_5_Carboxylate__91__d__93__" id="M_1_pyrroline_5_Carboxylate__91__d__93__" name="1_pyrroline_5_Carboxylate[d]" compartment="d" hasOnlySubstanceUnits="false" boundaryCondition="false" constant="false"/>

<species metaid="M_1_pyrroline_5_Carboxylate__91__d__93____91__c__93__" id="M_1_pyrroline_5_Carboxylate__91__d__93____91__c__93__" name="1_pyrroline_5_Carboxylate[d][c]" compartment="c" hasOnlySubstanceUnits="false" boundaryCondition="false" constant="false"/>

<species metaid="M_1_pyrroline_5_Carboxylate__91__n__93__" id="M_1_pyrroline_5_Carboxylate__91__n__93__" name="1_pyrroline_5_Carboxylate[n]" compartment="n" hasOnlySubstanceUnits="false" boundaryCondition="false" constant="false"/>

<species metaid="M_1_pyrroline_5_Carboxylate__91__n__93____91__c__93__" id="M_1_pyrroline_5_Carboxylate__91__n__93____91__c__93__" name="1_pyrroline_5_Carboxylate[n][c]" compartment="c" hasOnlySubstanceUnits="false" boundaryCondition="false" constant="false"/>

<species metaid="M_2Oxobutanoate__91__d__93____91__c__93__" id="M_2Oxobutanoate__91__d__93____91__c__93__" name="2Oxobutanoate[d][c]" compartment="c" hasOnlySubstanceUnits="false" boundaryCondition="false" constant="false"/>

<species metaid="M_2Oxobutanoate__91__n__93____91__c__93__" id="M_2Oxobutanoate__91__n__93____91__c__93__" name="2Oxobutanoate[n][c]" compartment="c" hasOnlySubstanceUnits="false" boundaryCondition="false" constant="false"/>

<species metaid="M_2PGA__91__d__93__" id="M_2PGA__91__d__93__" name="2PGA[d]" compartment="d" hasOnlySubstanceUnits="false" boundaryCondition="false" constant="false"/>

<species metaid="M_2PGA__91__d__93____91__c__93__" id="M_2PGA__91__d__93____91__c__93__" name="2PGA[d][c]" compartment="c" hasOnlySubstanceUnits="false" boundaryCondition="false" constant="false"/>

<species metaid="M_2PGA__91__n__93__" id="M_2PGA__91__n__93__" name="2PGA[n]" compartment="n" hasOnlySubstanceUnits="false" boundaryCondition="false" constant="false"/>

<species metaid="M_2PGA__91__n__93____91__c__93__" id="M_2PGA__91__n__93____91__c__93__" name="2PGA[n][c]" compartment="c" hasOnlySubstanceUnits="false" boundaryCondition="false" constant="false"/>

<species metaid="M_3PGA__91__d__93__" id="M_3PGA__91__d__93__" name="3PGA[d]" compartment="d" hasOnlySubstanceUnits="false" boundaryCondition="false" constant="false"/>

<species metaid="M_3PGA__91__d__93____91__c__93__" id="M_3PGA__91__d__93____91__c__93__" name="3PGA[d][c]" compartment="c" hasOnlySubstanceUnits="false" boundaryCondition="false" constant="false"/>

<species metaid="M_3PGA__91__n__93__" id="M_3PGA__91__n__93__" name="3PGA[n]" compartment="n" hasOnlySubstanceUnits="false" boundaryCondition="false" constant="false"/>

<species metaid="M_3PGA__91__n__93____91__c__93__" id="M_3PGA__91__n__93____91__c__93__" name="3PGA[n][c]" compartment="c" hasOnlySubstanceUnits="false" boundaryCondition="false" constant="false"/>

<species metaid="M_4_HydroxyPhenylpyruvate__91__d__93____91__c__93__" id="M_4_HydroxyPhenylpyruvate__91__d__93____91__c__93__" name="4_HydroxyPhenylpyruvate[d][c]" compartment="c" hasOnlySubstanceUnits="false" boundaryCondition="false" constant="false"/>

<species metaid="M_4_HydroxyPhenylpyruvate__91__n__93____91__c__93__" id="M_4_HydroxyPhenylpyruvate__91__n__93____91__c__93__" name="4_HydroxyPhenylpyruvate[n][c]" compartment="c" hasOnlySubstanceUnits="false" boundaryCondition="false" constant="false"/>

<species metaid="M_5p_Ribosyl_1_pp__91__d__93____91__c__93__" id="M_5p_Ribosyl_1_pp__91__d__93____91__c__93__" name="5p_Ribosyl_1_pp[d][c]" compartment="c" hasOnlySubstanceUnits="false" boundaryCondition="false" constant="false"/>

<species metaid="M_5p_Ribosyl_1_pp__91__n__93____91__c__93__" id="M_5p_Ribosyl_1_pp__91__n__93____91__c__93__" name="5p_Ribosyl_1_pp[n][c]" compartment="c" hasOnlySubstanceUnits="false" boundaryCondition="false" constant="false"/>

<species metaid="M_AA_exp__91__d__93__" id="M_AA_exp__91__d__93__" name="AA_exp[d]" compartment="d" hasOnlySubstanceUnits="false" boundaryCondition="false" constant="false"/>

<species metaid="M_AA_exp__91__n__93__" id="M_AA_exp__91__n__93__" name="AA_exp[n]" compartment="n" hasOnlySubstanceUnits="false" boundaryCondition="false" constant="false"/>

<species metaid="M_ADP__91__d__93__" id="M_ADP__91__d__93__" name="ADP[d]" compartment="d" hasOnlySubstanceUnits="false" boundaryCondition="false" constant="false"/>

<species metaid="M_ADP__91__d__93____91__c__93__" id="M_ADP__91__d__93____91__c__93__" name="ADP[d][c]" compartment="c" hasOnlySubstanceUnits="false" boundaryCondition="false" constant="false"/>

<species metaid="M_ADP__91__d__93____91__m__93__" id="M_ADP__91__d__93____91__m__93__" name="ADP[d][m]" compartment="m" hasOnlySubstanceUnits="false" boundaryCondition="false" constant="false"/>

<species metaid="M_ADP__91__n__93__" id="M_ADP__91__n__93__" name="ADP[n]" compartment="n" hasOnlySubstanceUnits="false" boundaryCondition="false" constant="false"/>

<species metaid="M_ADP__91__n__93____91__c__93__" id="M_ADP__91__n__93____91__c__93__" name="ADP[n][c]" compartment="c" hasOnlySubstanceUnits="false" boundaryCondition="false" constant="false"/>

<species metaid="M_ADP__91__n__93____91__m__93__" id="M_ADP__91__n__93____91__m__93__" name="ADP[n][m]" compartment="m" hasOnlySubstanceUnits="false" boundaryCondition="false" constant="false"/>

<species metaid="M_AMP__91__d__93__" id="M_AMP__91__d__93__" name="AMP[d]" compartment="d" hasOnlySubstanceUnits="false" boundaryCondition="false" constant="false"/>

<species metaid="M_AMP__91__d__93____91__c__93__" id="M_AMP__91__d__93____91__c__93__" name="AMP[d][c]" compartment="c" hasOnlySubstanceUnits="false" boundaryCondition="false" constant="false"/>

<species metaid="M_AMP__91__d__93____91__m__93__" id="M_AMP__91__d__93____91__m__93__" name="AMP[d][m]" compartment="m" hasOnlySubstanceUnits="false" boundaryCondition="false" constant="false"/>

<species metaid="M_AMP__91__n__93__" id="M_AMP__91__n__93__" name="AMP[n]" compartment="n" hasOnlySubstanceUnits="false" boundaryCondition="false" constant="false"/>

<species metaid="M_AMP__91__n__93____91__c__93__" id="M_AMP__91__n__93____91__c__93__" name="AMP[n][c]" compartment="c" hasOnlySubstanceUnits="false" boundaryCondition="false" constant="false"/>

<species metaid="M_AMP__91__n__93____91__m__93__" id="M_AMP__91__n__93____91__m__93__" name="AMP[n][m]" compartment="m" hasOnlySubstanceUnits="false" boundaryCondition="false" constant="false"/>

<species metaid="M_APS__91__d__93____91__c__93__" id="M_APS__91__d__93____91__c__93__" name="APS[d][c]" compartment="c" hasOnlySubstanceUnits="false" boundaryCondition="false" constant="false"/>

<species metaid="M_APS__91__n__93____91__c__93__" id="M_APS__91__n__93____91__c__93__" name="APS[n][c]" compartment="c" hasOnlySubstanceUnits="false" boundaryCondition="false" constant="false"/>

<species metaid="M_ATP__91__d__93__" id="M_ATP__91__d__93__" name="ATP[d]" compartment="d" hasOnlySubstanceUnits="false" boundaryCondition="false" constant="false"/>

<species metaid="M_ATP__91__d__93____91__c__93__" id="M_ATP__91__d__93____91__c__93__" name="ATP[d][c]" compartment="c" hasOnlySubstanceUnits="false" boundaryCondition="false" constant="false"/>

<species metaid="M_ATP__91__d__93____91__m__93__" id="M_ATP__91__d__93____91__m__93__" name="ATP[d][m]" compartment="m" hasOnlySubstanceUnits="false" boundaryCondition="false" constant="false"/>

<species metaid="M_ATP__91__n__93__" id="M_ATP__91__n__93__" name="ATP[n]" compartment="n" hasOnlySubstanceUnits="false" boundaryCondition="false" constant="false"/>

<species metaid="M_ATP__91__n__93____91__c__93__" id="M_ATP__91__n__93____91__c__93__" name="ATP[n][c]" compartment="c" hasOnlySubstanceUnits="false" boundaryCondition="false" constant="false"/>

<species metaid="M_ATP__91__n__93____91__m__93__" id="M_ATP__91__n__93____91__m__93__" name="ATP[n][m]" compartment="m" hasOnlySubstanceUnits="false" boundaryCondition="false" constant="false"/>

<species metaid="M_Acetate__91__d__93__" id="M_Acetate__91__d__93__" name="Acetate[d]" compartment="d" hasOnlySubstanceUnits="false" boundaryCondition="false" constant="false"/>

<species metaid="M_Acetate__91__d__93____91__c__93__" id="M_Acetate__91__d__93____91__c__93__" name="Acetate[d][c]" compartment="c" hasOnlySubstanceUnits="false" boundaryCondition="false" constant="false"/>

<species metaid="M_Acetate__91__d__93____91__m__93__" id="M_Acetate__91__d__93____91__m__93__" name="Acetate[d][m]" compartment="m" hasOnlySubstanceUnits="false" boundaryCondition="false" constant="false"/>

<species metaid="M_Acetate__91__n__93__" id="M_Acetate__91__n__93__" name="Acetate[n]" compartment="n" hasOnlySubstanceUnits="false" boundaryCondition="false" constant="false"/>

<species metaid="M_Acetate__91__n__93____91__c__93__" id="M_Acetate__91__n__93____91__c__93__" name="Acetate[n][c]" compartment="c" hasOnlySubstanceUnits="false" boundaryCondition="false" constant="false"/>

<species metaid="M_Acetate__91__n__93____91__m__93__" id="M_Acetate__91__n__93____91__m__93__" name="Acetate[n][m]" compartment="m" hasOnlySubstanceUnits="false" boundaryCondition="false" constant="false"/>

<species metaid="M_AcetylCoA__91__d__93__" id="M_AcetylCoA__91__d__93__" name="AcetylCoA[d]" compartment="d" hasOnlySubstanceUnits="false" boundaryCondition="false" constant="false"/>

<species metaid="M_AcetylCoA__91__d__93____91__c__93__" id="M_AcetylCoA__91__d__93____91__c__93__" name="AcetylCoA[d][c]" compartment="c" hasOnlySubstanceUnits="false" boundaryCondition="false" constant="false"/>

<species metaid="M_AcetylCoA__91__d__93____91__m__93__" id="M_AcetylCoA__91__d__93____91__m__93__" name="AcetylCoA[d][m]" compartment="m" hasOnlySubstanceUnits="false" boundaryCondition="false" constant="false"/>

<species metaid="M_AcetylCoA__91__n__93__" id="M_AcetylCoA__91__n__93__" name="AcetylCoA[n]" compartment="n" hasOnlySubstanceUnits="false" boundaryCondition="false" constant="false"/>

<species metaid="M_AcetylCoA__91__n__93____91__c__93__" id="M_AcetylCoA__91__n__93____91__c__93__" name="AcetylCoA[n][c]" compartment="c" hasOnlySubstanceUnits="false" boundaryCondition="false" constant="false"/>

<species metaid="M_AcetylCoA__91__n__93____91__m__93__" id="M_AcetylCoA__91__n__93____91__m__93__" name="AcetylCoA[n][m]" compartment="m" hasOnlySubstanceUnits="false" boundaryCondition="false" constant="false"/>

<species metaid="M_AcetylHomoserine__91__d__93____91__c__93__" id="M_AcetylHomoserine__91__d__93____91__c__93__" name="AcetylHomoserine[d][c]" compartment="c" hasOnlySubstanceUnits="false" boundaryCondition="false" constant="false"/>

<species metaid="M_AcetylHomoserine__91__n__93____91__c__93__" id="M_AcetylHomoserine__91__n__93____91__c__93__" name="AcetylHomoserine[n][c]" compartment="c" hasOnlySubstanceUnits="false" boundaryCondition="false" constant="false"/>

<species metaid="M_Alanine__91__d__93__" id="M_Alanine__91__d__93__" name="Alanine[d]" compartment="d" hasOnlySubstanceUnits="false" boundaryCondition="false" constant="false"/>

<species metaid="M_Alanine__91__d__93____91__c__93__" id="M_Alanine__91__d__93____91__c__93__" name="Alanine[d][c]" compartment="c" hasOnlySubstanceUnits="false" boundaryCondition="false" constant="false"/>

<species metaid="M_Alanine__91__n__93__" id="M_Alanine__91__n__93__" name="Alanine[n]" compartment="n" hasOnlySubstanceUnits="false" boundaryCondition="false" constant="false"/>

<species metaid="M_Alanine__91__n__93____91__c__93__" id="M_Alanine__91__n__93____91__c__93__" name="Alanine[n][c]" compartment="c" hasOnlySubstanceUnits="false" boundaryCondition="false" constant="false"/>

<species metaid="M_Alanine_exp__91__d__93__" id="M_Alanine_exp__91__d__93__" name="Alanine_exp[d]" compartment="d" hasOnlySubstanceUnits="false" boundaryCondition="false" constant="false"/>

<species metaid="M_Alanine_exp__91__n__93__" id="M_Alanine_exp__91__n__93__" name="Alanine_exp[n]" compartment="n" hasOnlySubstanceUnits="false" boundaryCondition="false" constant="false"/>

<species metaid="M_Arginine__91__d__93__" id="M_Arginine__91__d__93__" name="Arginine[d]" compartment="d" hasOnlySubstanceUnits="false" boundaryCondition="false" constant="false"/>

<species metaid="M_Arginine__91__d__93____91__c__93__" id="M_Arginine__91__d__93____91__c__93__" name="Arginine[d][c]" compartment="c" hasOnlySubstanceUnits="false" boundaryCondition="false" constant="false"/>

<species metaid="M_Arginine__91__n__93__" id="M_Arginine__91__n__93__" name="Arginine[n]" compartment="n" hasOnlySubstanceUnits="false" boundaryCondition="false" constant="false"/>

<species metaid="M_Arginine__91__n__93____91__c__93__" id="M_Arginine__91__n__93____91__c__93__" name="Arginine[n][c]" compartment="c" hasOnlySubstanceUnits="false" boundaryCondition="false" constant="false"/>

<species metaid="M_Arginine_exp__91__d__93__" id="M_Arginine_exp__91__d__93__" name="Arginine_exp[d]" compartment="d" hasOnlySubstanceUnits="false" boundaryCondition="false" constant="false"/>

<species metaid="M_Arginine_exp__91__n__93__" id="M_Arginine_exp__91__n__93__" name="Arginine_exp[n]" compartment="n" hasOnlySubstanceUnits="false" boundaryCondition="false" constant="false"/>

<species metaid="M_Asparagine__91__d__93__" id="M_Asparagine__91__d__93__" name="Asparagine[d]" compartment="d" hasOnlySubstanceUnits="false" boundaryCondition="false" constant="false"/>

<species metaid="M_Asparagine__91__n__93__" id="M_Asparagine__91__n__93__" name="Asparagine[n]" compartment="n" hasOnlySubstanceUnits="false" boundaryCondition="false" constant="false"/>

<species metaid="M_Asparagine_exp__91__d__93__" id="M_Asparagine_exp__91__d__93__" name="Asparagine_exp[d]" compartment="d" hasOnlySubstanceUnits="false" boundaryCondition="false" constant="false"/>

<species metaid="M_Asparagine_exp__91__n__93__" id="M_Asparagine_exp__91__n__93__" name="Asparagine_exp[n]" compartment="n" hasOnlySubstanceUnits="false" boundaryCondition="false" constant="false"/>

<species metaid="M_Aspartate__91__d__93__" id="M_Aspartate__91__d__93__" name="Aspartate[d]" compartment="d" hasOnlySubstanceUnits="false" boundaryCondition="false" constant="false"/>

<species metaid="M_Aspartate__91__d__93____91__c__93__" id="M_Aspartate__91__d__93____91__c__93__" name="Aspartate[d][c]" compartment="c" hasOnlySubstanceUnits="false" boundaryCondition="false" constant="false"/>

<species metaid="M_Aspartate__91__d__93____91__m__93__" id="M_Aspartate__91__d__93____91__m__93__" name="Aspartate[d][m]" compartment="m" hasOnlySubstanceUnits="false" boundaryCondition="false" constant="false"/>

<species metaid="M_Aspartate__91__n__93__" id="M_Aspartate__91__n__93__" name="Aspartate[n]" compartment="n" hasOnlySubstanceUnits="false" boundaryCondition="false" constant="false"/>

<species metaid="M_Aspartate__91__n__93____91__c__93__" id="M_Aspartate__91__n__93____91__c__93__" name="Aspartate[n][c]" compartment="c" hasOnlySubstanceUnits="false" boundaryCondition="false" constant="false"/>

<species metaid="M_Aspartate__91__n__93____91__m__93__" id="M_Aspartate__91__n__93____91__m__93__" name="Aspartate[n][m]" compartment="m" hasOnlySubstanceUnits="false" boundaryCondition="false" constant="false"/>

<species metaid="M_Aspartate_exp__91__d__93__" id="M_Aspartate_exp__91__d__93__" name="Aspartate_exp[d]" compartment="d" hasOnlySubstanceUnits="false" boundaryCondition="false" constant="false"/>

<species metaid="M_Aspartate_exp__91__n__93__" id="M_Aspartate_exp__91__n__93__" name="Aspartate_exp[n]" compartment="n" hasOnlySubstanceUnits="false" boundaryCondition="false" constant="false"/>

<species metaid="M_Aspartate_semialdehyde__91__d__93____91__c__93__" id="M_Aspartate_semialdehyde__91__d__93____91__c__93__" name="Aspartate_semialdehyde[d][c]" compartment="c" hasOnlySubstanceUnits="false" boundaryCondition="false" constant="false"/>

<species metaid="M_Aspartate_semialdehyde__91__n__93____91__c__93__" id="M_Aspartate_semialdehyde__91__n__93____91__c__93__" name="Aspartate_semialdehyde[n][c]" compartment="c" hasOnlySubstanceUnits="false" boundaryCondition="false" constant="false"/>

<species metaid="M_Biomass__91__d__93__" id="M_Biomass__91__d__93__" name="Biomass[d]" compartment="d" hasOnlySubstanceUnits="false" boundaryCondition="false" constant="false"/>

<species metaid="M_Biomass__91__n__93__" id="M_Biomass__91__n__93__" name="Biomass[n]" compartment="n" hasOnlySubstanceUnits="false" boundaryCondition="false" constant="false"/>

<species metaid="M_Brassicasterol__91__d__93__" id="M_Brassicasterol__91__d__93__" name="Brassicasterol[d]" compartment="d" hasOnlySubstanceUnits="false" boundaryCondition="false" constant="false"/>

<species metaid="M_Brassicasterol__91__n__93__" id="M_Brassicasterol__91__n__93__" name="Brassicasterol[n]" compartment="n" hasOnlySubstanceUnits="false" boundaryCondition="false" constant="false"/>

<species metaid="M_CH2THF__91__d__93__" id="M_CH2THF__91__d__93__" name="CH2THF[d]" compartment="d" hasOnlySubstanceUnits="false" boundaryCondition="false" constant="false"/>

<species metaid="M_CH2THF__91__d__93____91__m__93__" id="M_CH2THF__91__d__93____91__m__93__" name="CH2THF[d][m]" compartment="m" hasOnlySubstanceUnits="false" boundaryCondition="false" constant="false"/>

<species metaid="M_CH2THF__91__n__93__" id="M_CH2THF__91__n__93__" name="CH2THF[n]" compartment="n" hasOnlySubstanceUnits="false" boundaryCondition="false" constant="false"/>

<species metaid="M_CH2THF__91__n__93____91__m__93__" id="M_CH2THF__91__n__93____91__m__93__" name="CH2THF[n][m]" compartment="m" hasOnlySubstanceUnits="false" boundaryCondition="false" constant="false"/>

<species metaid="M_CH3THF__91__d__93__" id="M_CH3THF__91__d__93__" name="CH3THF[d]" compartment="d" hasOnlySubstanceUnits="false" boundaryCondition="false" constant="false"/>

<species metaid="M_CH3THF__91__n__93__" id="M_CH3THF__91__n__93__" name="CH3THF[n]" compartment="n" hasOnlySubstanceUnits="false" boundaryCondition="false" constant="false"/>

<species metaid="M_CO2__91__d__93__" id="M_CO2__91__d__93__" name="CO2[d]" compartment="d" hasOnlySubstanceUnits="false" boundaryCondition="false" constant="false"/>

<species metaid="M_CO2__91__n__93__" id="M_CO2__91__n__93__" name="CO2[n]" compartment="n" hasOnlySubstanceUnits="false" boundaryCondition="false" constant="false"/>

<species metaid="M_Chorismate__91__d__93____91__c__93__" id="M_Chorismate__91__d__93____91__c__93__" name="Chorismate[d][c]" compartment="c" hasOnlySubstanceUnits="false" boundaryCondition="false" constant="false"/>

<species metaid="M_Chorismate__91__n__93____91__c__93__" id="M_Chorismate__91__n__93____91__c__93__" name="Chorismate[n][c]" compartment="c" hasOnlySubstanceUnits="false" boundaryCondition="false" constant="false"/>

<species metaid="M_Cisaconitate__91__d__93__" id="M_Cisaconitate__91__d__93__" name="Cisaconitate[d]" compartment="d" hasOnlySubstanceUnits="false" boundaryCondition="false" constant="false"/>

<species metaid="M_Cisaconitate__91__d__93____91__m__93__" id="M_Cisaconitate__91__d__93____91__m__93__" name="Cisaconitate[d][m]" compartment="m" hasOnlySubstanceUnits="false" boundaryCondition="false" constant="false"/>

<species metaid="M_Cisaconitate__91__n__93__" id="M_Cisaconitate__91__n__93__" name="Cisaconitate[n]" compartment="n" hasOnlySubstanceUnits="false" boundaryCondition="false" constant="false"/>

<species metaid="M_Cisaconitate__91__n__93____91__m__93__" id="M_Cisaconitate__91__n__93____91__m__93__" name="Cisaconitate[n][m]" compartment="m" hasOnlySubstanceUnits="false" boundaryCondition="false" constant="false"/>

<species metaid="M_Citrate__91__d__93__" id="M_Citrate__91__d__93__" name="Citrate[d]" compartment="d" hasOnlySubstanceUnits="false" boundaryCondition="false" constant="false"/>

<species metaid="M_Citrate__91__d__93____91__m__93__" id="M_Citrate__91__d__93____91__m__93__" name="Citrate[d][m]" compartment="m" hasOnlySubstanceUnits="false" boundaryCondition="false" constant="false"/>

<species metaid="M_Citrate__91__n__93__" id="M_Citrate__91__n__93__" name="Citrate[n]" compartment="n" hasOnlySubstanceUnits="false" boundaryCondition="false" constant="false"/>

<species metaid="M_Citrate__91__n__93____91__m__93__" id="M_Citrate__91__n__93____91__m__93__" name="Citrate[n][m]" compartment="m" hasOnlySubstanceUnits="false" boundaryCondition="false" constant="false"/>

<species metaid="M_Citrulline__91__d__93____91__c__93__" id="M_Citrulline__91__d__93____91__c__93__" name="Citrulline[d][c]" compartment="c" hasOnlySubstanceUnits="false" boundaryCondition="false" constant="false"/>

<species metaid="M_Citrulline__91__n__93____91__c__93__" id="M_Citrulline__91__n__93____91__c__93__" name="Citrulline[n][c]" compartment="c" hasOnlySubstanceUnits="false" boundaryCondition="false" constant="false"/>

<species metaid="M_CoenzymeA__91__d__93__" id="M_CoenzymeA__91__d__93__" name="CoenzymeA[d]" compartment="d" hasOnlySubstanceUnits="false" boundaryCondition="false" constant="false"/>

<species metaid="M_CoenzymeA__91__d__93____91__c__93__" id="M_CoenzymeA__91__d__93____91__c__93__" name="CoenzymeA[d][c]" compartment="c" hasOnlySubstanceUnits="false" boundaryCondition="false" constant="false"/>

<species metaid="M_CoenzymeA__91__d__93____91__m__93__" id="M_CoenzymeA__91__d__93____91__m__93__" name="CoenzymeA[d][m]" compartment="m" hasOnlySubstanceUnits="false" boundaryCondition="false" constant="false"/>

<species metaid="M_CoenzymeA__91__n__93__" id="M_CoenzymeA__91__n__93__" name="CoenzymeA[n]" compartment="n" hasOnlySubstanceUnits="false" boundaryCondition="false" constant="false"/>

<species metaid="M_CoenzymeA__91__n__93____91__c__93__" id="M_CoenzymeA__91__n__93____91__c__93__" name="CoenzymeA[n][c]" compartment="c" hasOnlySubstanceUnits="false" boundaryCondition="false" constant="false"/>

<species metaid="M_CoenzymeA__91__n__93____91__m__93__" id="M_CoenzymeA__91__n__93____91__m__93__" name="CoenzymeA[n][m]" compartment="m" hasOnlySubstanceUnits="false" boundaryCondition="false" constant="false"/>

<species metaid="M_Cystathionine__91__d__93____91__c__93__" id="M_Cystathionine__91__d__93____91__c__93__" name="Cystathionine[d][c]" compartment="c" hasOnlySubstanceUnits="false" boundaryCondition="false" constant="false"/>

<species metaid="M_Cystathionine__91__n__93____91__c__93__" id="M_Cystathionine__91__n__93____91__c__93__" name="Cystathionine[n][c]" compartment="c" hasOnlySubstanceUnits="false" boundaryCondition="false" constant="false"/>

<species metaid="M_Cysteine__91__d__93__" id="M_Cysteine__91__d__93__" name="Cysteine[d]" compartment="d" hasOnlySubstanceUnits="false" boundaryCondition="false" constant="false"/>

<species metaid="M_Cysteine__91__d__93____91__c__93__" id="M_Cysteine__91__d__93____91__c__93__" name="Cysteine[d][c]" compartment="c" hasOnlySubstanceUnits="false" boundaryCondition="false" constant="false"/>

<species metaid="M_Cysteine__91__d__93____91__m__93__" id="M_Cysteine__91__d__93____91__m__93__" name="Cysteine[d][m]" compartment="m" hasOnlySubstanceUnits="false" boundaryCondition="false" constant="false"/>

<species metaid="M_Cysteine__91__n__93__" id="M_Cysteine__91__n__93__" name="Cysteine[n]" compartment="n" hasOnlySubstanceUnits="false" boundaryCondition="false" constant="false"/>

<species metaid="M_Cysteine__91__n__93____91__c__93__" id="M_Cysteine__91__n__93____91__c__93__" name="Cysteine[n][c]" compartment="c" hasOnlySubstanceUnits="false" boundaryCondition="false" constant="false"/>

<species metaid="M_Cysteine__91__n__93____91__m__93__" id="M_Cysteine__91__n__93____91__m__93__" name="Cysteine[n][m]" compartment="m" hasOnlySubstanceUnits="false" boundaryCondition="false" constant="false"/>

<species metaid="M_Cystine__91__d__93__" id="M_Cystine__91__d__93__" name="Cystine[d]" compartment="d" hasOnlySubstanceUnits="false" boundaryCondition="false" constant="false"/>

<species metaid="M_Cystine__91__d__93____91__c__93__" id="M_Cystine__91__d__93____91__c__93__" name="Cystine[d][c]" compartment="c" hasOnlySubstanceUnits="false" boundaryCondition="false" constant="false"/>

<species metaid="M_Cystine__91__n__93__" id="M_Cystine__91__n__93__" name="Cystine[n]" compartment="n" hasOnlySubstanceUnits="false" boundaryCondition="false" constant="false"/>

<species metaid="M_Cystine__91__n__93____91__c__93__" id="M_Cystine__91__n__93____91__c__93__" name="Cystine[n][c]" compartment="c" hasOnlySubstanceUnits="false" boundaryCondition="false" constant="false"/>

<species metaid="M_DHAP__91__d__93__" id="M_DHAP__91__d__93__" name="DHAP[d]" compartment="d" hasOnlySubstanceUnits="false" boundaryCondition="false" constant="false"/>

<species metaid="M_DHAP__91__d__93____91__c__93__" id="M_DHAP__91__d__93____91__c__93__" name="DHAP[d][c]" compartment="c" hasOnlySubstanceUnits="false" boundaryCondition="false" constant="false"/>

<species metaid="M_DHAP__91__d__93____91__m__93__" id="M_DHAP__91__d__93____91__m__93__" name="DHAP[d][m]" compartment="m" hasOnlySubstanceUnits="false" boundaryCondition="false" constant="false"/>

<species metaid="M_DHAP__91__n__93__" id="M_DHAP__91__n__93__" name="DHAP[n]" compartment="n" hasOnlySubstanceUnits="false" boundaryCondition="false" constant="false"/>

<species metaid="M_DHAP__91__n__93____91__c__93__" id="M_DHAP__91__n__93____91__c__93__" name="DHAP[n][c]" compartment="c" hasOnlySubstanceUnits="false" boundaryCondition="false" constant="false"/>

<species metaid="M_DHAP__91__n__93____91__m__93__" id="M_DHAP__91__n__93____91__m__93__" name="DHAP[n][m]" compartment="m" hasOnlySubstanceUnits="false" boundaryCondition="false" constant="false"/>

<species metaid="M_E4P__91__d__93__" id="M_E4P__91__d__93__" name="E4P[d]" compartment="d" hasOnlySubstanceUnits="false" boundaryCondition="false" constant="false"/>

<species metaid="M_E4P__91__d__93____91__c__93__" id="M_E4P__91__d__93____91__c__93__" name="E4P[d][c]" compartment="c" hasOnlySubstanceUnits="false" boundaryCondition="false" constant="false"/>

<species metaid="M_E4P__91__n__93__" id="M_E4P__91__n__93__" name="E4P[n]" compartment="n" hasOnlySubstanceUnits="false" boundaryCondition="false" constant="false"/>

<species metaid="M_E4P__91__n__93____91__c__93__" id="M_E4P__91__n__93____91__c__93__" name="E4P[n][c]" compartment="c" hasOnlySubstanceUnits="false" boundaryCondition="false" constant="false"/>

<species metaid="M_F6P__91__d__93__" id="M_F6P__91__d__93__" name="F6P[d]" compartment="d" hasOnlySubstanceUnits="false" boundaryCondition="false" constant="false"/>

<species metaid="M_F6P__91__d__93____91__c__93__" id="M_F6P__91__d__93____91__c__93__" name="F6P[d][c]" compartment="c" hasOnlySubstanceUnits="false" boundaryCondition="false" constant="false"/>

<species metaid="M_F6P__91__n__93__" id="M_F6P__91__n__93__" name="F6P[n]" compartment="n" hasOnlySubstanceUnits="false" boundaryCondition="false" constant="false"/>

<species metaid="M_F6P__91__n__93____91__c__93__" id="M_F6P__91__n__93____91__c__93__" name="F6P[n][c]" compartment="c" hasOnlySubstanceUnits="false" boundaryCondition="false" constant="false"/>

<species metaid="M_FADH2__91__d__93____91__m__93__" id="M_FADH2__91__d__93____91__m__93__" name="FADH2[d][m]" compartment="m" hasOnlySubstanceUnits="false" boundaryCondition="false" constant="false"/>

<species metaid="M_FADH2__91__n__93____91__m__93__" id="M_FADH2__91__n__93____91__m__93__" name="FADH2[n][m]" compartment="m" hasOnlySubstanceUnits="false" boundaryCondition="false" constant="false"/>

<species metaid="M_FAD__91__d__93____91__m__93__" id="M_FAD__91__d__93____91__m__93__" name="FAD[d][m]" compartment="m" hasOnlySubstanceUnits="false" boundaryCondition="false" constant="false"/>

<species metaid="M_FAD__91__n__93____91__m__93__" id="M_FAD__91__n__93____91__m__93__" name="FAD[n][m]" compartment="m" hasOnlySubstanceUnits="false" boundaryCondition="false" constant="false"/>

<species metaid="M_FBP__91__d__93__" id="M_FBP__91__d__93__" name="FBP[d]" compartment="d" hasOnlySubstanceUnits="false" boundaryCondition="false" constant="false"/>

<species metaid="M_FBP__91__d__93____91__c__93__" id="M_FBP__91__d__93____91__c__93__" name="FBP[d][c]" compartment="c" hasOnlySubstanceUnits="false" boundaryCondition="false" constant="false"/>

<species metaid="M_FBP__91__n__93__" id="M_FBP__91__n__93__" name="FBP[n]" compartment="n" hasOnlySubstanceUnits="false" boundaryCondition="false" constant="false"/>

<species metaid="M_FBP__91__n__93____91__c__93__" id="M_FBP__91__n__93____91__c__93__" name="FBP[n][c]" compartment="c" hasOnlySubstanceUnits="false" boundaryCondition="false" constant="false"/>

<species metaid="M_Formate__91__d__93__" id="M_Formate__91__d__93__" name="Formate[d]" compartment="d" hasOnlySubstanceUnits="false" boundaryCondition="false" constant="false"/>

<species metaid="M_Formate__91__d__93____91__c__93__" id="M_Formate__91__d__93____91__c__93__" name="Formate[d][c]" compartment="c" hasOnlySubstanceUnits="false" boundaryCondition="false" constant="false"/>

<species metaid="M_Formate__91__d__93____91__m__93__" id="M_Formate__91__d__93____91__m__93__" name="Formate[d][m]" compartment="m" hasOnlySubstanceUnits="false" boundaryCondition="false" constant="false"/>

<species metaid="M_Formate__91__n__93__" id="M_Formate__91__n__93__" name="Formate[n]" compartment="n" hasOnlySubstanceUnits="false" boundaryCondition="false" constant="false"/>

<species metaid="M_Formate__91__n__93____91__c__93__" id="M_Formate__91__n__93____91__c__93__" name="Formate[n][c]" compartment="c" hasOnlySubstanceUnits="false" boundaryCondition="false" constant="false"/>

<species metaid="M_Formate__91__n__93____91__m__93__" id="M_Formate__91__n__93____91__m__93__" name="Formate[n][m]" compartment="m" hasOnlySubstanceUnits="false" boundaryCondition="false" constant="false"/>

<species metaid="M_Fumarate__91__d__93__" id="M_Fumarate__91__d__93__" name="Fumarate[d]" compartment="d" hasOnlySubstanceUnits="false" boundaryCondition="false" constant="false"/>

<species metaid="M_Fumarate__91__d__93____91__m__93__" id="M_Fumarate__91__d__93____91__m__93__" name="Fumarate[d][m]" compartment="m" hasOnlySubstanceUnits="false" boundaryCondition="false" constant="false"/>

<species metaid="M_Fumarate__91__n__93__" id="M_Fumarate__91__n__93__" name="Fumarate[n]" compartment="n" hasOnlySubstanceUnits="false" boundaryCondition="false" constant="false"/>

<species metaid="M_Fumarate__91__n__93____91__m__93__" id="M_Fumarate__91__n__93____91__m__93__" name="Fumarate[n][m]" compartment="m" hasOnlySubstanceUnits="false" boundaryCondition="false" constant="false"/>

<species metaid="M_G1P__91__d__93__" id="M_G1P__91__d__93__" name="G1P[d]" compartment="d" hasOnlySubstanceUnits="false" boundaryCondition="false" constant="false"/>

<species metaid="M_G1P__91__n__93__" id="M_G1P__91__n__93__" name="G1P[n]" compartment="n" hasOnlySubstanceUnits="false" boundaryCondition="false" constant="false"/>

<species metaid="M_G3P__91__d__93__" id="M_G3P__91__d__93__" name="G3P[d]" compartment="d" hasOnlySubstanceUnits="false" boundaryCondition="false" constant="false"/>

<species metaid="M_G3P__91__d__93____91__c__93__" id="M_G3P__91__d__93____91__c__93__" name="G3P[d][c]" compartment="c" hasOnlySubstanceUnits="false" boundaryCondition="false" constant="false"/>

<species metaid="M_G3P__91__n__93__" id="M_G3P__91__n__93__" name="G3P[n]" compartment="n" hasOnlySubstanceUnits="false" boundaryCondition="false" constant="false"/>

<species metaid="M_G3P__91__n__93____91__c__93__" id="M_G3P__91__n__93____91__c__93__" name="G3P[n][c]" compartment="c" hasOnlySubstanceUnits="false" boundaryCondition="false" constant="false"/>

<species metaid="M_G6P__91__d__93__" id="M_G6P__91__d__93__" name="G6P[d]" compartment="d" hasOnlySubstanceUnits="false" boundaryCondition="false" constant="false"/>

<species metaid="M_G6P__91__d__93____91__c__93__" id="M_G6P__91__d__93____91__c__93__" name="G6P[d][c]" compartment="c" hasOnlySubstanceUnits="false" boundaryCondition="false" constant="false"/>

<species metaid="M_G6P__91__n__93__" id="M_G6P__91__n__93__" name="G6P[n]" compartment="n" hasOnlySubstanceUnits="false" boundaryCondition="false" constant="false"/>

<species metaid="M_G6P__91__n__93____91__c__93__" id="M_G6P__91__n__93____91__c__93__" name="G6P[n][c]" compartment="c" hasOnlySubstanceUnits="false" boundaryCondition="false" constant="false"/>

<species metaid="M_GSH__91__d__93____91__c__93__" id="M_GSH__91__d__93____91__c__93__" name="GSH[d][c]" compartment="c" hasOnlySubstanceUnits="false" boundaryCondition="false" constant="false"/>

<species metaid="M_GSH__91__n__93____91__c__93__" id="M_GSH__91__n__93____91__c__93__" name="GSH[n][c]" compartment="c" hasOnlySubstanceUnits="false" boundaryCondition="false" constant="false"/>

<species metaid="M_GSSG__91__d__93____91__c__93__" id="M_GSSG__91__d__93____91__c__93__" name="GSSG[d][c]" compartment="c" hasOnlySubstanceUnits="false" boundaryCondition="false" constant="false"/>

<species metaid="M_GSSG__91__n__93____91__c__93__" id="M_GSSG__91__n__93____91__c__93__" name="GSSG[n][c]" compartment="c" hasOnlySubstanceUnits="false" boundaryCondition="false" constant="false"/>

<species metaid="M_Glutamate__91__d__93__" id="M_Glutamate__91__d__93__" name="Glutamate[d]" compartment="d" hasOnlySubstanceUnits="false" boundaryCondition="false" constant="false"/>

<species metaid="M_Glutamate__91__d__93____91__c__93__" id="M_Glutamate__91__d__93____91__c__93__" name="Glutamate[d][c]" compartment="c" hasOnlySubstanceUnits="false" boundaryCondition="false" constant="false"/>

<species metaid="M_Glutamate__91__d__93____91__m__93__" id="M_Glutamate__91__d__93____91__m__93__" name="Glutamate[d][m]" compartment="m" hasOnlySubstanceUnits="false" boundaryCondition="false" constant="false"/>

<species metaid="M_Glutamate__91__d__93____91__p__93__" id="M_Glutamate__91__d__93____91__p__93__" name="Glutamate[d][p]" compartment="p" hasOnlySubstanceUnits="false" boundaryCondition="false" constant="false"/>

<species metaid="M_Glutamate__91__n__93__" id="M_Glutamate__91__n__93__" name="Glutamate[n]" compartment="n" hasOnlySubstanceUnits="false" boundaryCondition="false" constant="false"/>

<species metaid="M_Glutamate__91__n__93____91__c__93__" id="M_Glutamate__91__n__93____91__c__93__" name="Glutamate[n][c]" compartment="c" hasOnlySubstanceUnits="false" boundaryCondition="false" constant="false"/>

<species metaid="M_Glutamate__91__n__93____91__m__93__" id="M_Glutamate__91__n__93____91__m__93__" name="Glutamate[n][m]" compartment="m" hasOnlySubstanceUnits="false" boundaryCondition="false" constant="false"/>

<species metaid="M_Glutamate__91__n__93____91__p__93__" id="M_Glutamate__91__n__93____91__p__93__" name="Glutamate[n][p]" compartment="p" hasOnlySubstanceUnits="false" boundaryCondition="false" constant="false"/>

<species metaid="M_Glutamate_exp__91__d__93__" id="M_Glutamate_exp__91__d__93__" name="Glutamate_exp[d]" compartment="d" hasOnlySubstanceUnits="false" boundaryCondition="false" constant="false"/>

<species metaid="M_Glutamate_exp__91__n__93__" id="M_Glutamate_exp__91__n__93__" name="Glutamate_exp[n]" compartment="n" hasOnlySubstanceUnits="false" boundaryCondition="false" constant="false"/>

<species metaid="M_Glutamate_y_Semialdehyde__91__d__93__" id="M_Glutamate_y_Semialdehyde__91__d__93__" name="Glutamate_y_Semialdehyde[d]" compartment="d" hasOnlySubstanceUnits="false" boundaryCondition="false" constant="false"/>

<species metaid="M_Glutamate_y_Semialdehyde__91__d__93____91__c__93__" id="M_Glutamate_y_Semialdehyde__91__d__93____91__c__93__" name="Glutamate_y_Semialdehyde[d][c]" compartment="c" hasOnlySubstanceUnits="false" boundaryCondition="false" constant="false"/>

<species metaid="M_Glutamate_y_Semialdehyde__91__n__93__" id="M_Glutamate_y_Semialdehyde__91__n__93__" name="Glutamate_y_Semialdehyde[n]" compartment="n" hasOnlySubstanceUnits="false" boundaryCondition="false" constant="false"/>

<species metaid="M_Glutamate_y_Semialdehyde__91__n__93____91__c__93__" id="M_Glutamate_y_Semialdehyde__91__n__93____91__c__93__" name="Glutamate_y_Semialdehyde[n][c]" compartment="c" hasOnlySubstanceUnits="false" boundaryCondition="false" constant="false"/>

<species metaid="M_Glutamine__91__d__93__" id="M_Glutamine__91__d__93__" name="Glutamine[d]" compartment="d" hasOnlySubstanceUnits="false" boundaryCondition="false" constant="false"/>

<species metaid="M_Glutamine__91__d__93____91__c__93__" id="M_Glutamine__91__d__93____91__c__93__" name="Glutamine[d][c]" compartment="c" hasOnlySubstanceUnits="false" boundaryCondition="false" constant="false"/>

<species metaid="M_Glutamine__91__d__93____91__m__93__" id="M_Glutamine__91__d__93____91__m__93__" name="Glutamine[d][m]" compartment="m" hasOnlySubstanceUnits="false" boundaryCondition="false" constant="false"/>

<species metaid="M_Glutamine__91__n__93__" id="M_Glutamine__91__n__93__" name="Glutamine[n]" compartment="n" hasOnlySubstanceUnits="false" boundaryCondition="false" constant="false"/>

<species metaid="M_Glutamine__91__n__93____91__c__93__" id="M_Glutamine__91__n__93____91__c__93__" name="Glutamine[n][c]" compartment="c" hasOnlySubstanceUnits="false" boundaryCondition="false" constant="false"/>

<species metaid="M_Glutamine__91__n__93____91__m__93__" id="M_Glutamine__91__n__93____91__m__93__" name="Glutamine[n][m]" compartment="m" hasOnlySubstanceUnits="false" boundaryCondition="false" constant="false"/>

<species metaid="M_Glutamine_exp__91__d__93__" id="M_Glutamine_exp__91__d__93__" name="Glutamine_exp[d]" compartment="d" hasOnlySubstanceUnits="false" boundaryCondition="false" constant="false"/>

<species metaid="M_Glutamine_exp__91__n__93__" id="M_Glutamine_exp__91__n__93__" name="Glutamine_exp[n]" compartment="n" hasOnlySubstanceUnits="false" boundaryCondition="false" constant="false"/>

<species metaid="M_Glycerate__91__d__93____91__c__93__" id="M_Glycerate__91__d__93____91__c__93__" name="Glycerate[d][c]" compartment="c" hasOnlySubstanceUnits="false" boundaryCondition="false" constant="false"/>

<species metaid="M_Glycerate__91__d__93____91__p__93__" id="M_Glycerate__91__d__93____91__p__93__" name="Glycerate[d][p]" compartment="p" hasOnlySubstanceUnits="false" boundaryCondition="false" constant="false"/>

<species metaid="M_Glycerate__91__n__93____91__c__93__" id="M_Glycerate__91__n__93____91__c__93__" name="Glycerate[n][c]" compartment="c" hasOnlySubstanceUnits="false" boundaryCondition="false" constant="false"/>

<species metaid="M_Glycerate__91__n__93____91__p__93__" id="M_Glycerate__91__n__93____91__p__93__" name="Glycerate[n][p]" compartment="p" hasOnlySubstanceUnits="false" boundaryCondition="false" constant="false"/>

<species metaid="M_Glycerol3P__91__d__93__" id="M_Glycerol3P__91__d__93__" name="Glycerol3P[d]" compartment="d" hasOnlySubstanceUnits="false" boundaryCondition="false" constant="false"/>

<species metaid="M_Glycerol3P__91__d__93____91__m__93__" id="M_Glycerol3P__91__d__93____91__m__93__" name="Glycerol3P[d][m]" compartment="m" hasOnlySubstanceUnits="false" boundaryCondition="false" constant="false"/>

<species metaid="M_Glycerol3P__91__n__93__" id="M_Glycerol3P__91__n__93__" name="Glycerol3P[n]" compartment="n" hasOnlySubstanceUnits="false" boundaryCondition="false" constant="false"/>

<species metaid="M_Glycerol3P__91__n__93____91__m__93__" id="M_Glycerol3P__91__n__93____91__m__93__" name="Glycerol3P[n][m]" compartment="m" hasOnlySubstanceUnits="false" boundaryCondition="false" constant="false"/>

<species metaid="M_Glycerol__91__d__93__" id="M_Glycerol__91__d__93__" name="Glycerol[d]" compartment="d" hasOnlySubstanceUnits="false" boundaryCondition="false" constant="false"/>

<species metaid="M_Glycerol__91__n__93__" id="M_Glycerol__91__n__93__" name="Glycerol[n]" compartment="n" hasOnlySubstanceUnits="false" boundaryCondition="false" constant="false"/>

<species metaid="M_Glycine__91__d__93__" id="M_Glycine__91__d__93__" name="Glycine[d]" compartment="d" hasOnlySubstanceUnits="false" boundaryCondition="false" constant="false"/>

<species metaid="M_Glycine__91__d__93____91__c__93__" id="M_Glycine__91__d__93____91__c__93__" name="Glycine[d][c]" compartment="c" hasOnlySubstanceUnits="false" boundaryCondition="false" constant="false"/>

<species metaid="M_Glycine__91__d__93____91__m__93__" id="M_Glycine__91__d__93____91__m__93__" name="Glycine[d][m]" compartment="m" hasOnlySubstanceUnits="false" boundaryCondition="false" constant="false"/>

<species metaid="M_Glycine__91__d__93____91__p__93__" id="M_Glycine__91__d__93____91__p__93__" name="Glycine[d][p]" compartment="p" hasOnlySubstanceUnits="false" boundaryCondition="false" constant="false"/>

<species metaid="M_Glycine__91__n__93__" id="M_Glycine__91__n__93__" name="Glycine[n]" compartment="n" hasOnlySubstanceUnits="false" boundaryCondition="false" constant="false"/>

<species metaid="M_Glycine__91__n__93____91__c__93__" id="M_Glycine__91__n__93____91__c__93__" name="Glycine[n][c]" compartment="c" hasOnlySubstanceUnits="false" boundaryCondition="false" constant="false"/>

<species metaid="M_Glycine__91__n__93____91__m__93__" id="M_Glycine__91__n__93____91__m__93__" name="Glycine[n][m]" compartment="m" hasOnlySubstanceUnits="false" boundaryCondition="false" constant="false"/>

<species metaid="M_Glycine__91__n__93____91__p__93__" id="M_Glycine__91__n__93____91__p__93__" name="Glycine[n][p]" compartment="p" hasOnlySubstanceUnits="false" boundaryCondition="false" constant="false"/>

<species metaid="M_Glycine_exp__91__d__93__" id="M_Glycine_exp__91__d__93__" name="Glycine_exp[d]" compartment="d" hasOnlySubstanceUnits="false" boundaryCondition="false" constant="false"/>

<species metaid="M_Glycine_exp__91__n__93__" id="M_Glycine_exp__91__n__93__" name="Glycine_exp[n]" compartment="n" hasOnlySubstanceUnits="false" boundaryCondition="false" constant="false"/>

<species metaid="M_Glycolate__91__d__93____91__c__93__" id="M_Glycolate__91__d__93____91__c__93__" name="Glycolate[d][c]" compartment="c" hasOnlySubstanceUnits="false" boundaryCondition="false" constant="false"/>

<species metaid="M_Glycolate__91__d__93____91__p__93__" id="M_Glycolate__91__d__93____91__p__93__" name="Glycolate[d][p]" compartment="p" hasOnlySubstanceUnits="false" boundaryCondition="false" constant="false"/>

<species metaid="M_Glycolate__91__n__93____91__c__93__" id="M_Glycolate__91__n__93____91__c__93__" name="Glycolate[n][c]" compartment="c" hasOnlySubstanceUnits="false" boundaryCondition="false" constant="false"/>

<species metaid="M_Glycolate__91__n__93____91__p__93__" id="M_Glycolate__91__n__93____91__p__93__" name="Glycolate[n][p]" compartment="p" hasOnlySubstanceUnits="false" boundaryCondition="false" constant="false"/>

<species metaid="M_Glyoxylate__91__d__93____91__p__93__" id="M_Glyoxylate__91__d__93____91__p__93__" name="Glyoxylate[d][p]" compartment="p" hasOnlySubstanceUnits="false" boundaryCondition="false" constant="false"/>

<species metaid="M_Glyoxylate__91__n__93____91__p__93__" id="M_Glyoxylate__91__n__93____91__p__93__" name="Glyoxylate[n][p]" compartment="p" hasOnlySubstanceUnits="false" boundaryCondition="false" constant="false"/>

<species metaid="M_H2O2__91__d__93__" id="M_H2O2__91__d__93__" name="H2O2[d]" compartment="d" hasOnlySubstanceUnits="false" boundaryCondition="false" constant="false"/>

<species metaid="M_H2O2__91__d__93____91__c__93__" id="M_H2O2__91__d__93____91__c__93__" name="H2O2[d][c]" compartment="c" hasOnlySubstanceUnits="false" boundaryCondition="false" constant="false"/>

<species metaid="M_H2O2__91__n__93__" id="M_H2O2__91__n__93__" name="H2O2[n]" compartment="n" hasOnlySubstanceUnits="false" boundaryCondition="false" constant="false"/>

<species metaid="M_H2O2__91__n__93____91__c__93__" id="M_H2O2__91__n__93____91__c__93__" name="H2O2[n][c]" compartment="c" hasOnlySubstanceUnits="false" boundaryCondition="false" constant="false"/>

<species metaid="M_H2O__91__d__93__" id="M_H2O__91__d__93__" name="H2O[d]" compartment="d" hasOnlySubstanceUnits="false" boundaryCondition="false" constant="false"/>

<species metaid="M_H2O__91__n__93__" id="M_H2O__91__n__93__" name="H2O[n]" compartment="n" hasOnlySubstanceUnits="false" boundaryCondition="false" constant="false"/>

<species metaid="M_H2S__91__d__93__" id="M_H2S__91__d__93__" name="H2S[d]" compartment="d" hasOnlySubstanceUnits="false" boundaryCondition="false" constant="false"/>

<species metaid="M_H2S__91__d__93____91__c__93__" id="M_H2S__91__d__93____91__c__93__" name="H2S[d][c]" compartment="c" hasOnlySubstanceUnits="false" boundaryCondition="false" constant="false"/>

<species metaid="M_H2S__91__d__93____91__m__93__" id="M_H2S__91__d__93____91__m__93__" name="H2S[d][m]" compartment="m" hasOnlySubstanceUnits="false" boundaryCondition="false" constant="false"/>

<species metaid="M_H2S__91__n__93__" id="M_H2S__91__n__93__" name="H2S[n]" compartment="n" hasOnlySubstanceUnits="false" boundaryCondition="false" constant="false"/>

<species metaid="M_H2S__91__n__93____91__c__93__" id="M_H2S__91__n__93____91__c__93__" name="H2S[n][c]" compartment="c" hasOnlySubstanceUnits="false" boundaryCondition="false" constant="false"/>

<species metaid="M_H2S__91__n__93____91__m__93__" id="M_H2S__91__n__93____91__m__93__" name="H2S[n][m]" compartment="m" hasOnlySubstanceUnits="false" boundaryCondition="false" constant="false"/>

<species metaid="M_HNO3__91__d__93__" id="M_HNO3__91__d__93__" name="HNO3[d]" compartment="d" hasOnlySubstanceUnits="false" boundaryCondition="false" constant="false"/>

<species metaid="M_HNO3__91__n__93__" id="M_HNO3__91__n__93__" name="HNO3[n]" compartment="n" hasOnlySubstanceUnits="false" boundaryCondition="false" constant="false"/>

<species metaid="M_Histidine__91__d__93__" id="M_Histidine__91__d__93__" name="Histidine[d]" compartment="d" hasOnlySubstanceUnits="false" boundaryCondition="false" constant="false"/>

<species metaid="M_Histidine__91__d__93____91__c__93__" id="M_Histidine__91__d__93____91__c__93__" name="Histidine[d][c]" compartment="c" hasOnlySubstanceUnits="false" boundaryCondition="false" constant="false"/>

<species metaid="M_Histidine__91__n__93__" id="M_Histidine__91__n__93__" name="Histidine[n]" compartment="n" hasOnlySubstanceUnits="false" boundaryCondition="false" constant="false"/>

<species metaid="M_Histidine__91__n__93____91__c__93__" id="M_Histidine__91__n__93____91__c__93__" name="Histidine[n][c]" compartment="c" hasOnlySubstanceUnits="false" boundaryCondition="false" constant="false"/>

<species metaid="M_Histidine_exp__91__d__93__" id="M_Histidine_exp__91__d__93__" name="Histidine_exp[d]" compartment="d" hasOnlySubstanceUnits="false" boundaryCondition="false" constant="false"/>

<species metaid="M_Histidine_exp__91__n__93__" id="M_Histidine_exp__91__n__93__" name="Histidine_exp[n]" compartment="n" hasOnlySubstanceUnits="false" boundaryCondition="false" constant="false"/>

<species metaid="M_Homocysteine__91__d__93__" id="M_Homocysteine__91__d__93__" name="Homocysteine[d]" compartment="d" hasOnlySubstanceUnits="false" boundaryCondition="false" constant="false"/>

<species metaid="M_Homocysteine__91__d__93____91__c__93__" id="M_Homocysteine__91__d__93____91__c__93__" name="Homocysteine[d][c]" compartment="c" hasOnlySubstanceUnits="false" boundaryCondition="false" constant="false"/>

<species metaid="M_Homocysteine__91__n__93__" id="M_Homocysteine__91__n__93__" name="Homocysteine[n]" compartment="n" hasOnlySubstanceUnits="false" boundaryCondition="false" constant="false"/>

<species metaid="M_Homocysteine__91__n__93____91__c__93__" id="M_Homocysteine__91__n__93____91__c__93__" name="Homocysteine[n][c]" compartment="c" hasOnlySubstanceUnits="false" boundaryCondition="false" constant="false"/>

<species metaid="M_Homoserine__91__d__93____91__c__93__" id="M_Homoserine__91__d__93____91__c__93__" name="Homoserine[d][c]" compartment="c" hasOnlySubstanceUnits="false" boundaryCondition="false" constant="false"/>

<species metaid="M_Homoserine__91__n__93____91__c__93__" id="M_Homoserine__91__n__93____91__c__93__" name="Homoserine[n][c]" compartment="c" hasOnlySubstanceUnits="false" boundaryCondition="false" constant="false"/>

<species metaid="M_Isocitrate__91__d__93__" id="M_Isocitrate__91__d__93__" name="Isocitrate[d]" compartment="d" hasOnlySubstanceUnits="false" boundaryCondition="false" constant="false"/>

<species metaid="M_Isocitrate__91__d__93____91__m__93__" id="M_Isocitrate__91__d__93____91__m__93__" name="Isocitrate[d][m]" compartment="m" hasOnlySubstanceUnits="false" boundaryCondition="false" constant="false"/>

<species metaid="M_Isocitrate__91__n__93__" id="M_Isocitrate__91__n__93__" name="Isocitrate[n]" compartment="n" hasOnlySubstanceUnits="false" boundaryCondition="false" constant="false"/>

<species metaid="M_Isocitrate__91__n__93____91__m__93__" id="M_Isocitrate__91__n__93____91__m__93__" name="Isocitrate[n][m]" compartment="m" hasOnlySubstanceUnits="false" boundaryCondition="false" constant="false"/>

<species metaid="M_Isoleucine__91__d__93__" id="M_Isoleucine__91__d__93__" name="Isoleucine[d]" compartment="d" hasOnlySubstanceUnits="false" boundaryCondition="false" constant="false"/>

<species metaid="M_Isoleucine__91__d__93____91__c__93__" id="M_Isoleucine__91__d__93____91__c__93__" name="Isoleucine[d][c]" compartment="c" hasOnlySubstanceUnits="false" boundaryCondition="false" constant="false"/>

<species metaid="M_Isoleucine__91__n__93__" id="M_Isoleucine__91__n__93__" name="Isoleucine[n]" compartment="n" hasOnlySubstanceUnits="false" boundaryCondition="false" constant="false"/>

<species metaid="M_Isoleucine__91__n__93____91__c__93__" id="M_Isoleucine__91__n__93____91__c__93__" name="Isoleucine[n][c]" compartment="c" hasOnlySubstanceUnits="false" boundaryCondition="false" constant="false"/>

<species metaid="M_Isoleucine_exp__91__d__93__" id="M_Isoleucine_exp__91__d__93__" name="Isoleucine_exp[d]" compartment="d" hasOnlySubstanceUnits="false" boundaryCondition="false" constant="false"/>

<species metaid="M_Isoleucine_exp__91__n__93__" id="M_Isoleucine_exp__91__n__93__" name="Isoleucine_exp[n]" compartment="n" hasOnlySubstanceUnits="false" boundaryCondition="false" constant="false"/>

<species metaid="M_Leucine__91__d__93__" id="M_Leucine__91__d__93__" name="Leucine[d]" compartment="d" hasOnlySubstanceUnits="false" boundaryCondition="false" constant="false"/>

<species metaid="M_Leucine__91__d__93____91__c__93__" id="M_Leucine__91__d__93____91__c__93__" name="Leucine[d][c]" compartment="c" hasOnlySubstanceUnits="false" boundaryCondition="false" constant="false"/>

<species metaid="M_Leucine__91__n__93__" id="M_Leucine__91__n__93__" name="Leucine[n]" compartment="n" hasOnlySubstanceUnits="false" boundaryCondition="false" constant="false"/>

<species metaid="M_Leucine__91__n__93____91__c__93__" id="M_Leucine__91__n__93____91__c__93__" name="Leucine[n][c]" compartment="c" hasOnlySubstanceUnits="false" boundaryCondition="false" constant="false"/>

<species metaid="M_Leucine_exp__91__d__93__" id="M_Leucine_exp__91__d__93__" name="Leucine_exp[d]" compartment="d" hasOnlySubstanceUnits="false" boundaryCondition="false" constant="false"/>

<species metaid="M_Leucine_exp__91__n__93__" id="M_Leucine_exp__91__n__93__" name="Leucine_exp[n]" compartment="n" hasOnlySubstanceUnits="false" boundaryCondition="false" constant="false"/>

<species metaid="M_Linoleate__91__d__93__" id="M_Linoleate__91__d__93__" name="Linoleate[d]" compartment="d" hasOnlySubstanceUnits="false" boundaryCondition="false" constant="false"/>

<species metaid="M_Linoleate__91__n__93__" id="M_Linoleate__91__n__93__" name="Linoleate[n]" compartment="n" hasOnlySubstanceUnits="false" boundaryCondition="false" constant="false"/>

<species metaid="M_Lysine__91__d__93__" id="M_Lysine__91__d__93__" name="Lysine[d]" compartment="d" hasOnlySubstanceUnits="false" boundaryCondition="false" constant="false"/>

<species metaid="M_Lysine__91__d__93____91__c__93__" id="M_Lysine__91__d__93____91__c__93__" name="Lysine[d][c]" compartment="c" hasOnlySubstanceUnits="false" boundaryCondition="false" constant="false"/>

<species metaid="M_Lysine__91__n__93__" id="M_Lysine__91__n__93__" name="Lysine[n]" compartment="n" hasOnlySubstanceUnits="false" boundaryCondition="false" constant="false"/>

<species metaid="M_Lysine__91__n__93____91__c__93__" id="M_Lysine__91__n__93____91__c__93__" name="Lysine[n][c]" compartment="c" hasOnlySubstanceUnits="false" boundaryCondition="false" constant="false"/>

<species metaid="M_Lysine_exp__91__d__93__" id="M_Lysine_exp__91__d__93__" name="Lysine_exp[d]" compartment="d" hasOnlySubstanceUnits="false" boundaryCondition="false" constant="false"/>

<species metaid="M_Lysine_exp__91__n__93__" id="M_Lysine_exp__91__n__93__" name="Lysine_exp[n]" compartment="n" hasOnlySubstanceUnits="false" boundaryCondition="false" constant="false"/>

<species metaid="M_Malate__91__d__93__" id="M_Malate__91__d__93__" name="Malate[d]" compartment="d" hasOnlySubstanceUnits="false" boundaryCondition="false" constant="false"/>

<species metaid="M_Malate__91__d__93____91__c__93__" id="M_Malate__91__d__93____91__c__93__" name="Malate[d][c]" compartment="c" hasOnlySubstanceUnits="false" boundaryCondition="false" constant="false"/>

<species metaid="M_Malate__91__d__93____91__m__93__" id="M_Malate__91__d__93____91__m__93__" name="Malate[d][m]" compartment="m" hasOnlySubstanceUnits="false" boundaryCondition="false" constant="false"/>

<species metaid="M_Malate__91__d__93____91__p__93__" id="M_Malate__91__d__93____91__p__93__" name="Malate[d][p]" compartment="p" hasOnlySubstanceUnits="false" boundaryCondition="false" constant="false"/>

<species metaid="M_Malate__91__n__93__" id="M_Malate__91__n__93__" name="Malate[n]" compartment="n" hasOnlySubstanceUnits="false" boundaryCondition="false" constant="false"/>

<species metaid="M_Malate__91__n__93____91__c__93__" id="M_Malate__91__n__93____91__c__93__" name="Malate[n][c]" compartment="c" hasOnlySubstanceUnits="false" boundaryCondition="false" constant="false"/>

<species metaid="M_Malate__91__n__93____91__m__93__" id="M_Malate__91__n__93____91__m__93__" name="Malate[n][m]" compartment="m" hasOnlySubstanceUnits="false" boundaryCondition="false" constant="false"/>

<species metaid="M_Malate__91__n__93____91__p__93__" id="M_Malate__91__n__93____91__p__93__" name="Malate[n][p]" compartment="p" hasOnlySubstanceUnits="false" boundaryCondition="false" constant="false"/>

<species metaid="M_MalonylCoA__91__d__93__" id="M_MalonylCoA__91__d__93__" name="MalonylCoA[d]" compartment="d" hasOnlySubstanceUnits="false" boundaryCondition="false" constant="false"/>

<species metaid="M_MalonylCoA__91__n__93__" id="M_MalonylCoA__91__n__93__" name="MalonylCoA[n]" compartment="n" hasOnlySubstanceUnits="false" boundaryCondition="false" constant="false"/>

<species metaid="M_MethenylTHF__91__d__93__" id="M_MethenylTHF__91__d__93__" name="MethenylTHF[d]" compartment="d" hasOnlySubstanceUnits="false" boundaryCondition="false" constant="false"/>

<species metaid="M_MethenylTHF__91__n__93__" id="M_MethenylTHF__91__n__93__" name="MethenylTHF[n]" compartment="n" hasOnlySubstanceUnits="false" boundaryCondition="false" constant="false"/>

<species metaid="M_Methionine__91__d__93__" id="M_Methionine__91__d__93__" name="Methionine[d]" compartment="d" hasOnlySubstanceUnits="false" boundaryCondition="false" constant="false"/>

<species metaid="M_Methionine__91__n__93__" id="M_Methionine__91__n__93__" name="Methionine[n]" compartment="n" hasOnlySubstanceUnits="false" boundaryCondition="false" constant="false"/>

<species metaid="M_Methionine_exp__91__d__93__" id="M_Methionine_exp__91__d__93__" name="Methionine_exp[d]" compartment="d" hasOnlySubstanceUnits="false" boundaryCondition="false" constant="false"/>

<species metaid="M_Methionine_exp__91__n__93__" id="M_Methionine_exp__91__n__93__" name="Methionine_exp[n]" compartment="n" hasOnlySubstanceUnits="false" boundaryCondition="false" constant="false"/>

<species metaid="M_NADH__91__d__93__" id="M_NADH__91__d__93__" name="NADH[d]" compartment="d" hasOnlySubstanceUnits="false" boundaryCondition="false" constant="false"/>

<species metaid="M_NADH__91__d__93____91__c__93__" id="M_NADH__91__d__93____91__c__93__" name="NADH[d][c]" compartment="c" hasOnlySubstanceUnits="false" boundaryCondition="false" constant="false"/>

<species metaid="M_NADH__91__d__93____91__m__93__" id="M_NADH__91__d__93____91__m__93__" name="NADH[d][m]" compartment="m" hasOnlySubstanceUnits="false" boundaryCondition="false" constant="false"/>

<species metaid="M_NADH__91__d__93____91__p__93__" id="M_NADH__91__d__93____91__p__93__" name="NADH[d][p]" compartment="p" hasOnlySubstanceUnits="false" boundaryCondition="false" constant="false"/>

<species metaid="M_NADH__91__n__93__" id="M_NADH__91__n__93__" name="NADH[n]" compartment="n" hasOnlySubstanceUnits="false" boundaryCondition="false" constant="false"/>

<species metaid="M_NADH__91__n__93____91__c__93__" id="M_NADH__91__n__93____91__c__93__" name="NADH[n][c]" compartment="c" hasOnlySubstanceUnits="false" boundaryCondition="false" constant="false"/>

<species metaid="M_NADH__91__n__93____91__m__93__" id="M_NADH__91__n__93____91__m__93__" name="NADH[n][m]" compartment="m" hasOnlySubstanceUnits="false" boundaryCondition="false" constant="false"/>

<species metaid="M_NADH__91__n__93____91__p__93__" id="M_NADH__91__n__93____91__p__93__" name="NADH[n][p]" compartment="p" hasOnlySubstanceUnits="false" boundaryCondition="false" constant="false"/>

<species metaid="M_NADPH__91__d__93__" id="M_NADPH__91__d__93__" name="NADPH[d]" compartment="d" hasOnlySubstanceUnits="false" boundaryCondition="false" constant="false"/>

<species metaid="M_NADPH__91__d__93____91__c__93__" id="M_NADPH__91__d__93____91__c__93__" name="NADPH[d][c]" compartment="c" hasOnlySubstanceUnits="false" boundaryCondition="false" constant="false"/>

<species metaid="M_NADPH__91__d__93____91__m__93__" id="M_NADPH__91__d__93____91__m__93__" name="NADPH[d][m]" compartment="m" hasOnlySubstanceUnits="false" boundaryCondition="false" constant="false"/>

<species metaid="M_NADPH__91__n__93__" id="M_NADPH__91__n__93__" name="NADPH[n]" compartment="n" hasOnlySubstanceUnits="false" boundaryCondition="false" constant="false"/>

<species metaid="M_NADPH__91__n__93____91__c__93__" id="M_NADPH__91__n__93____91__c__93__" name="NADPH[n][c]" compartment="c" hasOnlySubstanceUnits="false" boundaryCondition="false" constant="false"/>

<species metaid="M_NADPH__91__n__93____91__m__93__" id="M_NADPH__91__n__93____91__m__93__" name="NADPH[n][m]" compartment="m" hasOnlySubstanceUnits="false" boundaryCondition="false" constant="false"/>

<species metaid="M_NADP__91__d__93__" id="M_NADP__91__d__93__" name="NADP[d]" compartment="d" hasOnlySubstanceUnits="false" boundaryCondition="false" constant="false"/>

<species metaid="M_NADP__91__d__93____91__c__93__" id="M_NADP__91__d__93____91__c__93__" name="NADP[d][c]" compartment="c" hasOnlySubstanceUnits="false" boundaryCondition="false" constant="false"/>

<species metaid="M_NADP__91__d__93____91__m__93__" id="M_NADP__91__d__93____91__m__93__" name="NADP[d][m]" compartment="m" hasOnlySubstanceUnits="false" boundaryCondition="false" constant="false"/>

<species metaid="M_NADP__91__n__93__" id="M_NADP__91__n__93__" name="NADP[n]" compartment="n" hasOnlySubstanceUnits="false" boundaryCondition="false" constant="false"/>

<species metaid="M_NADP__91__n__93____91__c__93__" id="M_NADP__91__n__93____91__c__93__" name="NADP[n][c]" compartment="c" hasOnlySubstanceUnits="false" boundaryCondition="false" constant="false"/>

<species metaid="M_NADP__91__n__93____91__m__93__" id="M_NADP__91__n__93____91__m__93__" name="NADP[n][m]" compartment="m" hasOnlySubstanceUnits="false" boundaryCondition="false" constant="false"/>

<species metaid="M_NAD__91__d__93__" id="M_NAD__91__d__93__" name="NAD[d]" compartment="d" hasOnlySubstanceUnits="false" boundaryCondition="false" constant="false"/>

<species metaid="M_NAD__91__d__93____91__c__93__" id="M_NAD__91__d__93____91__c__93__" name="NAD[d][c]" compartment="c" hasOnlySubstanceUnits="false" boundaryCondition="false" constant="false"/>

<species metaid="M_NAD__91__d__93____91__m__93__" id="M_NAD__91__d__93____91__m__93__" name="NAD[d][m]" compartment="m" hasOnlySubstanceUnits="false" boundaryCondition="false" constant="false"/>

<species metaid="M_NAD__91__d__93____91__p__93__" id="M_NAD__91__d__93____91__p__93__" name="NAD[d][p]" compartment="p" hasOnlySubstanceUnits="false" boundaryCondition="false" constant="false"/>

<species metaid="M_NAD__91__n__93__" id="M_NAD__91__n__93__" name="NAD[n]" compartment="n" hasOnlySubstanceUnits="false" boundaryCondition="false" constant="false"/>

<species metaid="M_NAD__91__n__93____91__c__93__" id="M_NAD__91__n__93____91__c__93__" name="NAD[n][c]" compartment="c" hasOnlySubstanceUnits="false" boundaryCondition="false" constant="false"/>

<species metaid="M_NAD__91__n__93____91__m__93__" id="M_NAD__91__n__93____91__m__93__" name="NAD[n][m]" compartment="m" hasOnlySubstanceUnits="false" boundaryCondition="false" constant="false"/>

<species metaid="M_NAD__91__n__93____91__p__93__" id="M_NAD__91__n__93____91__p__93__" name="NAD[n][p]" compartment="p" hasOnlySubstanceUnits="false" boundaryCondition="false" constant="false"/>

<species metaid="M_NH3__91__d__93__" id="M_NH3__91__d__93__" name="NH3[d]" compartment="d" hasOnlySubstanceUnits="false" boundaryCondition="false" constant="false"/>

<species metaid="M_NH3__91__d__93____91__c__93__" id="M_NH3__91__d__93____91__c__93__" name="NH3[d][c]" compartment="c" hasOnlySubstanceUnits="false" boundaryCondition="false" constant="false"/>

<species metaid="M_NH3__91__d__93____91__m__93__" id="M_NH3__91__d__93____91__m__93__" name="NH3[d][m]" compartment="m" hasOnlySubstanceUnits="false" boundaryCondition="false" constant="false"/>

<species metaid="M_NH3__91__n__93__" id="M_NH3__91__n__93__" name="NH3[n]" compartment="n" hasOnlySubstanceUnits="false" boundaryCondition="false" constant="false"/>

<species metaid="M_NH3__91__n__93____91__c__93__" id="M_NH3__91__n__93____91__c__93__" name="NH3[n][c]" compartment="c" hasOnlySubstanceUnits="false" boundaryCondition="false" constant="false"/>

<species metaid="M_NH3__91__n__93____91__m__93__" id="M_NH3__91__n__93____91__m__93__" name="NH3[n][m]" compartment="m" hasOnlySubstanceUnits="false" boundaryCondition="false" constant="false"/>

<species metaid="M_NO2__91__d__93__" id="M_NO2__91__d__93__" name="NO2[d]" compartment="d" hasOnlySubstanceUnits="false" boundaryCondition="false" constant="false"/>

<species metaid="M_NO2__91__d__93____91__c__93__" id="M_NO2__91__d__93____91__c__93__" name="NO2[d][c]" compartment="c" hasOnlySubstanceUnits="false" boundaryCondition="false" constant="false"/>

<species metaid="M_NO2__91__n__93__" id="M_NO2__91__n__93__" name="NO2[n]" compartment="n" hasOnlySubstanceUnits="false" boundaryCondition="false" constant="false"/>

<species metaid="M_NO2__91__n__93____91__c__93__" id="M_NO2__91__n__93____91__c__93__" name="NO2[n][c]" compartment="c" hasOnlySubstanceUnits="false" boundaryCondition="false" constant="false"/>

<species metaid="M_O2__91__d__93__" id="M_O2__91__d__93__" name="O2[d]" compartment="d" hasOnlySubstanceUnits="false" boundaryCondition="false" constant="false"/>

<species metaid="M_O2__91__n__93__" id="M_O2__91__n__93__" name="O2[n]" compartment="n" hasOnlySubstanceUnits="false" boundaryCondition="false" constant="false"/>

<species metaid="M_OAS__91__d__93__" id="M_OAS__91__d__93__" name="OAS[d]" compartment="d" hasOnlySubstanceUnits="false" boundaryCondition="false" constant="false"/>

<species metaid="M_OAS__91__d__93____91__c__93__" id="M_OAS__91__d__93____91__c__93__" name="OAS[d][c]" compartment="c" hasOnlySubstanceUnits="false" boundaryCondition="false" constant="false"/>

<species metaid="M_OAS__91__d__93____91__m__93__" id="M_OAS__91__d__93____91__m__93__" name="OAS[d][m]" compartment="m" hasOnlySubstanceUnits="false" boundaryCondition="false" constant="false"/>

<species metaid="M_OAS__91__n__93__" id="M_OAS__91__n__93__" name="OAS[n]" compartment="n" hasOnlySubstanceUnits="false" boundaryCondition="false" constant="false"/>

<species metaid="M_OAS__91__n__93____91__c__93__" id="M_OAS__91__n__93____91__c__93__" name="OAS[n][c]" compartment="c" hasOnlySubstanceUnits="false" boundaryCondition="false" constant="false"/>

<species metaid="M_OAS__91__n__93____91__m__93__" id="M_OAS__91__n__93____91__m__93__" name="OAS[n][m]" compartment="m" hasOnlySubstanceUnits="false" boundaryCondition="false" constant="false"/>

<species metaid="M_Oleate__91__d__93__" id="M_Oleate__91__d__93__" name="Oleate[d]" compartment="d" hasOnlySubstanceUnits="false" boundaryCondition="false" constant="false"/>

<species metaid="M_Oleate__91__n__93__" id="M_Oleate__91__n__93__" name="Oleate[n]" compartment="n" hasOnlySubstanceUnits="false" boundaryCondition="false" constant="false"/>

<species metaid="M_Ornithine__91__d__93____91__c__93__" id="M_Ornithine__91__d__93____91__c__93__" name="Ornithine[d][c]" compartment="c" hasOnlySubstanceUnits="false" boundaryCondition="false" constant="false"/>

<species metaid="M_Ornithine__91__n__93____91__c__93__" id="M_Ornithine__91__n__93____91__c__93__" name="Ornithine[n][c]" compartment="c" hasOnlySubstanceUnits="false" boundaryCondition="false" constant="false"/>

<species metaid="M_Oxaloacetate__91__d__93__" id="M_Oxaloacetate__91__d__93__" name="Oxaloacetate[d]" compartment="d" hasOnlySubstanceUnits="false" boundaryCondition="false" constant="false"/>

<species metaid="M_Oxaloacetate__91__d__93____91__c__93__" id="M_Oxaloacetate__91__d__93____91__c__93__" name="Oxaloacetate[d][c]" compartment="c" hasOnlySubstanceUnits="false" boundaryCondition="false" constant="false"/>

<species metaid="M_Oxaloacetate__91__d__93____91__m__93__" id="M_Oxaloacetate__91__d__93____91__m__93__" name="Oxaloacetate[d][m]" compartment="m" hasOnlySubstanceUnits="false" boundaryCondition="false" constant="false"/>

<species metaid="M_Oxaloacetate__91__d__93____91__p__93__" id="M_Oxaloacetate__91__d__93____91__p__93__" name="Oxaloacetate[d][p]" compartment="p" hasOnlySubstanceUnits="false" boundaryCondition="false" constant="false"/>

<species metaid="M_Oxaloacetate__91__n__93__" id="M_Oxaloacetate__91__n__93__" name="Oxaloacetate[n]" compartment="n" hasOnlySubstanceUnits="false" boundaryCondition="false" constant="false"/>

<species metaid="M_Oxaloacetate__91__n__93____91__c__93__" id="M_Oxaloacetate__91__n__93____91__c__93__" name="Oxaloacetate[n][c]" compartment="c" hasOnlySubstanceUnits="false" boundaryCondition="false" constant="false"/>

<species metaid="M_Oxaloacetate__91__n__93____91__m__93__" id="M_Oxaloacetate__91__n__93____91__m__93__" name="Oxaloacetate[n][m]" compartment="m" hasOnlySubstanceUnits="false" boundaryCondition="false" constant="false"/>

<species metaid="M_Oxaloacetate__91__n__93____91__p__93__" id="M_Oxaloacetate__91__n__93____91__p__93__" name="Oxaloacetate[n][p]" compartment="p" hasOnlySubstanceUnits="false" boundaryCondition="false" constant="false"/>

<species metaid="M_Oxoglutarate__91__d__93__" id="M_Oxoglutarate__91__d__93__" name="Oxoglutarate[d]" compartment="d" hasOnlySubstanceUnits="false" boundaryCondition="false" constant="false"/>

<species metaid="M_Oxoglutarate__91__d__93____91__c__93__" id="M_Oxoglutarate__91__d__93____91__c__93__" name="Oxoglutarate[d][c]" compartment="c" hasOnlySubstanceUnits="false" boundaryCondition="false" constant="false"/>

<species metaid="M_Oxoglutarate__91__d__93____91__m__93__" id="M_Oxoglutarate__91__d__93____91__m__93__" name="Oxoglutarate[d][m]" compartment="m" hasOnlySubstanceUnits="false" boundaryCondition="false" constant="false"/>

<species metaid="M_Oxoglutarate__91__d__93____91__p__93__" id="M_Oxoglutarate__91__d__93____91__p__93__" name="Oxoglutarate[d][p]" compartment="p" hasOnlySubstanceUnits="false" boundaryCondition="false" constant="false"/>

<species metaid="M_Oxoglutarate__91__n__93__" id="M_Oxoglutarate__91__n__93__" name="Oxoglutarate[n]" compartment="n" hasOnlySubstanceUnits="false" boundaryCondition="false" constant="false"/>

<species metaid="M_Oxoglutarate__91__n__93____91__c__93__" id="M_Oxoglutarate__91__n__93____91__c__93__" name="Oxoglutarate[n][c]" compartment="c" hasOnlySubstanceUnits="false" boundaryCondition="false" constant="false"/>

<species metaid="M_Oxoglutarate__91__n__93____91__m__93__" id="M_Oxoglutarate__91__n__93____91__m__93__" name="Oxoglutarate[n][m]" compartment="m" hasOnlySubstanceUnits="false" boundaryCondition="false" constant="false"/>

<species metaid="M_Oxoglutarate__91__n__93____91__p__93__" id="M_Oxoglutarate__91__n__93____91__p__93__" name="Oxoglutarate[n][p]" compartment="p" hasOnlySubstanceUnits="false" boundaryCondition="false" constant="false"/>

<species metaid="M_PEP__91__d__93__" id="M_PEP__91__d__93__" name="PEP[d]" compartment="d" hasOnlySubstanceUnits="false" boundaryCondition="false" constant="false"/>

<species metaid="M_PEP__91__d__93____91__c__93__" id="M_PEP__91__d__93____91__c__93__" name="PEP[d][c]" compartment="c" hasOnlySubstanceUnits="false" boundaryCondition="false" constant="false"/>

<species metaid="M_PEP__91__n__93__" id="M_PEP__91__n__93__" name="PEP[n]" compartment="n" hasOnlySubstanceUnits="false" boundaryCondition="false" constant="false"/>

<species metaid="M_PEP__91__n__93____91__c__93__" id="M_PEP__91__n__93____91__c__93__" name="PEP[n][c]" compartment="c" hasOnlySubstanceUnits="false" boundaryCondition="false" constant="false"/>

<species metaid="M_PGIA__91__d__93____91__c__93__" id="M_PGIA__91__d__93____91__c__93__" name="PGIA[d][c]" compartment="c" hasOnlySubstanceUnits="false" boundaryCondition="false" constant="false"/>

<species metaid="M_PGIA__91__n__93____91__c__93__" id="M_PGIA__91__n__93____91__c__93__" name="PGIA[n][c]" compartment="c" hasOnlySubstanceUnits="false" boundaryCondition="false" constant="false"/>

<species metaid="M_PPi__91__d__93__" id="M_PPi__91__d__93__" name="PPi[d]" compartment="d" hasOnlySubstanceUnits="false" boundaryCondition="false" constant="false"/>

<species metaid="M_PPi__91__d__93____91__c__93__" id="M_PPi__91__d__93____91__c__93__" name="PPi[d][c]" compartment="c" hasOnlySubstanceUnits="false" boundaryCondition="false" constant="false"/>

<species metaid="M_PPi__91__d__93____91__m__93__" id="M_PPi__91__d__93____91__m__93__" name="PPi[d][m]" compartment="m" hasOnlySubstanceUnits="false" boundaryCondition="false" constant="false"/>

<species metaid="M_PPi__91__n__93__" id="M_PPi__91__n__93__" name="PPi[n]" compartment="n" hasOnlySubstanceUnits="false" boundaryCondition="false" constant="false"/>

<species metaid="M_PPi__91__n__93____91__c__93__" id="M_PPi__91__n__93____91__c__93__" name="PPi[n][c]" compartment="c" hasOnlySubstanceUnits="false" boundaryCondition="false" constant="false"/>

<species metaid="M_PPi__91__n__93____91__m__93__" id="M_PPi__91__n__93____91__m__93__" name="PPi[n][m]" compartment="m" hasOnlySubstanceUnits="false" boundaryCondition="false" constant="false"/>

<species metaid="M_Palmitate__91__d__93__" id="M_Palmitate__91__d__93__" name="Palmitate[d]" compartment="d" hasOnlySubstanceUnits="false" boundaryCondition="false" constant="false"/>

<species metaid="M_Palmitate__91__n__93__" id="M_Palmitate__91__n__93__" name="Palmitate[n]" compartment="n" hasOnlySubstanceUnits="false" boundaryCondition="false" constant="false"/>

<species metaid="M_Palmitoleate__91__d__93__" id="M_Palmitoleate__91__d__93__" name="Palmitoleate[d]" compartment="d" hasOnlySubstanceUnits="false" boundaryCondition="false" constant="false"/>

<species metaid="M_Palmitoleate__91__n__93__" id="M_Palmitoleate__91__n__93__" name="Palmitoleate[n]" compartment="n" hasOnlySubstanceUnits="false" boundaryCondition="false" constant="false"/>

<species metaid="M_Phenylalanine__91__d__93__" id="M_Phenylalanine__91__d__93__" name="Phenylalanine[d]" compartment="d" hasOnlySubstanceUnits="false" boundaryCondition="false" constant="false"/>

<species metaid="M_Phenylalanine__91__d__93____91__c__93__" id="M_Phenylalanine__91__d__93____91__c__93__" name="Phenylalanine[d][c]" compartment="c" hasOnlySubstanceUnits="false" boundaryCondition="false" constant="false"/>

<species metaid="M_Phenylalanine__91__n__93__" id="M_Phenylalanine__91__n__93__" name="Phenylalanine[n]" compartment="n" hasOnlySubstanceUnits="false" boundaryCondition="false" constant="false"/>

<species metaid="M_Phenylalanine__91__n__93____91__c__93__" id="M_Phenylalanine__91__n__93____91__c__93__" name="Phenylalanine[n][c]" compartment="c" hasOnlySubstanceUnits="false" boundaryCondition="false" constant="false"/>

<species metaid="M_Phenylalanine_exp__91__d__93__" id="M_Phenylalanine_exp__91__d__93__" name="Phenylalanine_exp[d]" compartment="d" hasOnlySubstanceUnits="false" boundaryCondition="false" constant="false"/>

<species metaid="M_Phenylalanine_exp__91__n__93__" id="M_Phenylalanine_exp__91__n__93__" name="Phenylalanine_exp[n]" compartment="n" hasOnlySubstanceUnits="false" boundaryCondition="false" constant="false"/>

<species metaid="M_Phenylpyruvate__91__d__93____91__c__93__" id="M_Phenylpyruvate__91__d__93____91__c__93__" name="Phenylpyruvate[d][c]" compartment="c" hasOnlySubstanceUnits="false" boundaryCondition="false" constant="false"/>

<species metaid="M_Phenylpyruvate__91__n__93____91__c__93__" id="M_Phenylpyruvate__91__n__93____91__c__93__" name="Phenylpyruvate[n][c]" compartment="c" hasOnlySubstanceUnits="false" boundaryCondition="false" constant="false"/>

<species metaid="M_Phosphohomoserine__91__d__93____91__c__93__" id="M_Phosphohomoserine__91__d__93____91__c__93__" name="Phosphohomoserine[d][c]" compartment="c" hasOnlySubstanceUnits="false" boundaryCondition="false" constant="false"/>

<species metaid="M_Phosphohomoserine__91__n__93____91__c__93__" id="M_Phosphohomoserine__91__n__93____91__c__93__" name="Phosphohomoserine[n][c]" compartment="c" hasOnlySubstanceUnits="false" boundaryCondition="false" constant="false"/>

<species metaid="M_Pi__91__d__93__" id="M_Pi__91__d__93__" name="Pi[d]" compartment="d" hasOnlySubstanceUnits="false" boundaryCondition="false" constant="false"/>

<species metaid="M_Pi__91__d__93____91__c__93__" id="M_Pi__91__d__93____91__c__93__" name="Pi[d][c]" compartment="c" hasOnlySubstanceUnits="false" boundaryCondition="false" constant="false"/>

<species metaid="M_Pi__91__d__93____91__m__93__" id="M_Pi__91__d__93____91__m__93__" name="Pi[d][m]" compartment="m" hasOnlySubstanceUnits="false" boundaryCondition="false" constant="false"/>

<species metaid="M_Pi__91__n__93__" id="M_Pi__91__n__93__" name="Pi[n]" compartment="n" hasOnlySubstanceUnits="false" boundaryCondition="false" constant="false"/>

<species metaid="M_Pi__91__n__93____91__c__93__" id="M_Pi__91__n__93____91__c__93__" name="Pi[n][c]" compartment="c" hasOnlySubstanceUnits="false" boundaryCondition="false" constant="false"/>

<species metaid="M_Pi__91__n__93____91__m__93__" id="M_Pi__91__n__93____91__m__93__" name="Pi[n][m]" compartment="m" hasOnlySubstanceUnits="false" boundaryCondition="false" constant="false"/>

<species metaid="M_PolyunsaturatedFats__91__d__93__" id="M_PolyunsaturatedFats__91__d__93__" name="PolyunsaturatedFats[d]" compartment="d" hasOnlySubstanceUnits="false" boundaryCondition="false" constant="false"/>

<species metaid="M_PolyunsaturatedFats__91__n__93__" id="M_PolyunsaturatedFats__91__n__93__" name="PolyunsaturatedFats[n]" compartment="n" hasOnlySubstanceUnits="false" boundaryCondition="false" constant="false"/>

<species metaid="M_Proline__91__d__93__" id="M_Proline__91__d__93__" name="Proline[d]" compartment="d" hasOnlySubstanceUnits="false" boundaryCondition="false" constant="false"/>

<species metaid="M_Proline__91__d__93____91__c__93__" id="M_Proline__91__d__93____91__c__93__" name="Proline[d][c]" compartment="c" hasOnlySubstanceUnits="false" boundaryCondition="false" constant="false"/>

<species metaid="M_Proline__91__n__93__" id="M_Proline__91__n__93__" name="Proline[n]" compartment="n" hasOnlySubstanceUnits="false" boundaryCondition="false" constant="false"/>

<species metaid="M_Proline__91__n__93____91__c__93__" id="M_Proline__91__n__93____91__c__93__" name="Proline[n][c]" compartment="c" hasOnlySubstanceUnits="false" boundaryCondition="false" constant="false"/>

<species metaid="M_Prot__91__d__93__" id="M_Prot__91__d__93__" name="Prot[d]" compartment="d" hasOnlySubstanceUnits="false" boundaryCondition="false" constant="false"/>

<species metaid="M_Prot__91__n__93__" id="M_Prot__91__n__93__" name="Prot[n]" compartment="n" hasOnlySubstanceUnits="false" boundaryCondition="false" constant="false"/>

<species metaid="M_Pyruvate__91__d__93__" id="M_Pyruvate__91__d__93__" name="Pyruvate[d]" compartment="d" hasOnlySubstanceUnits="false" boundaryCondition="false" constant="false"/>

<species metaid="M_Pyruvate__91__d__93____91__c__93__" id="M_Pyruvate__91__d__93____91__c__93__" name="Pyruvate[d][c]" compartment="c" hasOnlySubstanceUnits="false" boundaryCondition="false" constant="false"/>

<species metaid="M_Pyruvate__91__d__93____91__m__93__" id="M_Pyruvate__91__d__93____91__m__93__" name="Pyruvate[d][m]" compartment="m" hasOnlySubstanceUnits="false" boundaryCondition="false" constant="false"/>

<species metaid="M_Pyruvate__91__n__93__" id="M_Pyruvate__91__n__93__" name="Pyruvate[n]" compartment="n" hasOnlySubstanceUnits="false" boundaryCondition="false" constant="false"/>

<species metaid="M_Pyruvate__91__n__93____91__c__93__" id="M_Pyruvate__91__n__93____91__c__93__" name="Pyruvate[n][c]" compartment="c" hasOnlySubstanceUnits="false" boundaryCondition="false" constant="false"/>

<species metaid="M_Pyruvate__91__n__93____91__m__93__" id="M_Pyruvate__91__n__93____91__m__93__" name="Pyruvate[n][m]" compartment="m" hasOnlySubstanceUnits="false" boundaryCondition="false" constant="false"/>

<species metaid="M_R5P__91__d__93__" id="M_R5P__91__d__93__" name="R5P[d]" compartment="d" hasOnlySubstanceUnits="false" boundaryCondition="false" constant="false"/>

<species metaid="M_R5P__91__d__93____91__c__93__" id="M_R5P__91__d__93____91__c__93__" name="R5P[d][c]" compartment="c" hasOnlySubstanceUnits="false" boundaryCondition="false" constant="false"/>

<species metaid="M_R5P__91__n__93__" id="M_R5P__91__n__93__" name="R5P[n]" compartment="n" hasOnlySubstanceUnits="false" boundaryCondition="false" constant="false"/>

<species metaid="M_R5P__91__n__93____91__c__93__" id="M_R5P__91__n__93____91__c__93__" name="R5P[n][c]" compartment="c" hasOnlySubstanceUnits="false" boundaryCondition="false" constant="false"/>

<species metaid="M_RBP__91__d__93__" id="M_RBP__91__d__93__" name="RBP[d]" compartment="d" hasOnlySubstanceUnits="false" boundaryCondition="false" constant="false"/>

<species metaid="M_RBP__91__d__93____91__c__93__" id="M_RBP__91__d__93____91__c__93__" name="RBP[d][c]" compartment="c" hasOnlySubstanceUnits="false" boundaryCondition="false" constant="false"/>

<species metaid="M_RBP__91__n__93__" id="M_RBP__91__n__93__" name="RBP[n]" compartment="n" hasOnlySubstanceUnits="false" boundaryCondition="false" constant="false"/>

<species metaid="M_RBP__91__n__93____91__c__93__" id="M_RBP__91__n__93____91__c__93__" name="RBP[n][c]" compartment="c" hasOnlySubstanceUnits="false" boundaryCondition="false" constant="false"/>

<species metaid="M_Ru5P__91__d__93__" id="M_Ru5P__91__d__93__" name="Ru5P[d]" compartment="d" hasOnlySubstanceUnits="false" boundaryCondition="false" constant="false"/>

<species metaid="M_Ru5P__91__d__93____91__c__93__" id="M_Ru5P__91__d__93____91__c__93__" name="Ru5P[d][c]" compartment="c" hasOnlySubstanceUnits="false" boundaryCondition="false" constant="false"/>

<species metaid="M_Ru5P__91__n__93__" id="M_Ru5P__91__n__93__" name="Ru5P[n]" compartment="n" hasOnlySubstanceUnits="false" boundaryCondition="false" constant="false"/>

<species metaid="M_Ru5P__91__n__93____91__c__93__" id="M_Ru5P__91__n__93____91__c__93__" name="Ru5P[n][c]" compartment="c" hasOnlySubstanceUnits="false" boundaryCondition="false" constant="false"/>

<species metaid="M_RuBP__91__d__93____91__c__93__" id="M_RuBP__91__d__93____91__c__93__" name="RuBP[d][c]" compartment="c" hasOnlySubstanceUnits="false" boundaryCondition="false" constant="false"/>

<species metaid="M_RuBP__91__n__93____91__c__93__" id="M_RuBP__91__n__93____91__c__93__" name="RuBP[n][c]" compartment="c" hasOnlySubstanceUnits="false" boundaryCondition="false" constant="false"/>

<species metaid="M_S7P__91__d__93__" id="M_S7P__91__d__93__" name="S7P[d]" compartment="d" hasOnlySubstanceUnits="false" boundaryCondition="false" constant="false"/>

<species metaid="M_S7P__91__d__93____91__c__93__" id="M_S7P__91__d__93____91__c__93__" name="S7P[d][c]" compartment="c" hasOnlySubstanceUnits="false" boundaryCondition="false" constant="false"/>

<species metaid="M_S7P__91__n__93__" id="M_S7P__91__n__93__" name="S7P[n]" compartment="n" hasOnlySubstanceUnits="false" boundaryCondition="false" constant="false"/>

<species metaid="M_S7P__91__n__93____91__c__93__" id="M_S7P__91__n__93____91__c__93__" name="S7P[n][c]" compartment="c" hasOnlySubstanceUnits="false" boundaryCondition="false" constant="false"/>

<species metaid="M_SBP__91__d__93__" id="M_SBP__91__d__93__" name="SBP[d]" compartment="d" hasOnlySubstanceUnits="false" boundaryCondition="false" constant="false"/>

<species metaid="M_SBP__91__d__93____91__c__93__" id="M_SBP__91__d__93____91__c__93__" name="SBP[d][c]" compartment="c" hasOnlySubstanceUnits="false" boundaryCondition="false" constant="false"/>

<species metaid="M_SBP__91__n__93__" id="M_SBP__91__n__93__" name="SBP[n]" compartment="n" hasOnlySubstanceUnits="false" boundaryCondition="false" constant="false"/>

<species metaid="M_SBP__91__n__93____91__c__93__" id="M_SBP__91__n__93____91__c__93__" name="SBP[n][c]" compartment="c" hasOnlySubstanceUnits="false" boundaryCondition="false" constant="false"/>

<species metaid="M_S_Ad_Homocysteine__91__d__93__" id="M_S_Ad_Homocysteine__91__d__93__" name="S_Ad_Homocysteine[d]" compartment="d" hasOnlySubstanceUnits="false" boundaryCondition="false" constant="false"/>

<species metaid="M_S_Ad_Homocysteine__91__n__93__" id="M_S_Ad_Homocysteine__91__n__93__" name="S_Ad_Homocysteine[n]" compartment="n" hasOnlySubstanceUnits="false" boundaryCondition="false" constant="false"/>

<species metaid="M_S_Ade_Methionine__91__d__93__" id="M_S_Ade_Methionine__91__d__93__" name="S_Ade_Methionine[d]" compartment="d" hasOnlySubstanceUnits="false" boundaryCondition="false" constant="false"/>

<species metaid="M_S_Ade_Methionine__91__n__93__" id="M_S_Ade_Methionine__91__n__93__" name="S_Ade_Methionine[n]" compartment="n" hasOnlySubstanceUnits="false" boundaryCondition="false" constant="false"/>

<species metaid="M_Saturated_Fats__91__d__93__" id="M_Saturated_Fats__91__d__93__" name="Saturated_Fats[d]" compartment="d" hasOnlySubstanceUnits="false" boundaryCondition="false" constant="false"/>

<species metaid="M_Saturated_Fats__91__n__93__" id="M_Saturated_Fats__91__n__93__" name="Saturated_Fats[n]" compartment="n" hasOnlySubstanceUnits="false" boundaryCondition="false" constant="false"/>

<species metaid="M_Serine__91__d__93__" id="M_Serine__91__d__93__" name="Serine[d]" compartment="d" hasOnlySubstanceUnits="false" boundaryCondition="false" constant="false"/>

<species metaid="M_Serine__91__d__93____91__c__93__" id="M_Serine__91__d__93____91__c__93__" name="Serine[d][c]" compartment="c" hasOnlySubstanceUnits="false" boundaryCondition="false" constant="false"/>

<species metaid="M_Serine__91__d__93____91__m__93__" id="M_Serine__91__d__93____91__m__93__" name="Serine[d][m]" compartment="m" hasOnlySubstanceUnits="false" boundaryCondition="false" constant="false"/>

<species metaid="M_Serine__91__d__93____91__p__93__" id="M_Serine__91__d__93____91__p__93__" name="Serine[d][p]" compartment="p" hasOnlySubstanceUnits="false" boundaryCondition="false" constant="false"/>

<species metaid="M_Serine__91__n__93__" id="M_Serine__91__n__93__" name="Serine[n]" compartment="n" hasOnlySubstanceUnits="false" boundaryCondition="false" constant="false"/>

<species metaid="M_Serine__91__n__93____91__c__93__" id="M_Serine__91__n__93____91__c__93__" name="Serine[n][c]" compartment="c" hasOnlySubstanceUnits="false" boundaryCondition="false" constant="false"/>

<species metaid="M_Serine__91__n__93____91__m__93__" id="M_Serine__91__n__93____91__m__93__" name="Serine[n][m]" compartment="m" hasOnlySubstanceUnits="false" boundaryCondition="false" constant="false"/>

<species metaid="M_Serine__91__n__93____91__p__93__" id="M_Serine__91__n__93____91__p__93__" name="Serine[n][p]" compartment="p" hasOnlySubstanceUnits="false" boundaryCondition="false" constant="false"/>

<species metaid="M_Serine_exp__91__d__93__" id="M_Serine_exp__91__d__93__" name="Serine_exp[d]" compartment="d" hasOnlySubstanceUnits="false" boundaryCondition="false" constant="false"/>

<species metaid="M_Serine_exp__91__n__93__" id="M_Serine_exp__91__n__93__" name="Serine_exp[n]" compartment="n" hasOnlySubstanceUnits="false" boundaryCondition="false" constant="false"/>

<species metaid="M_Stearate__91__d__93__" id="M_Stearate__91__d__93__" name="Stearate[d]" compartment="d" hasOnlySubstanceUnits="false" boundaryCondition="false" constant="false"/>

<species metaid="M_Stearate__91__n__93__" id="M_Stearate__91__n__93__" name="Stearate[n]" compartment="n" hasOnlySubstanceUnits="false" boundaryCondition="false" constant="false"/>

<species metaid="M_Succinate__91__d__93__" id="M_Succinate__91__d__93__" name="Succinate[d]" compartment="d" hasOnlySubstanceUnits="false" boundaryCondition="false" constant="false"/>

<species metaid="M_Succinate__91__d__93____91__m__93__" id="M_Succinate__91__d__93____91__m__93__" name="Succinate[d][m]" compartment="m" hasOnlySubstanceUnits="false" boundaryCondition="false" constant="false"/>

<species metaid="M_Succinate__91__n__93__" id="M_Succinate__91__n__93__" name="Succinate[n]" compartment="n" hasOnlySubstanceUnits="false" boundaryCondition="false" constant="false"/>

<species metaid="M_Succinate__91__n__93____91__m__93__" id="M_Succinate__91__n__93____91__m__93__" name="Succinate[n][m]" compartment="m" hasOnlySubstanceUnits="false" boundaryCondition="false" constant="false"/>

<species metaid="M_SuccinylCoA__91__d__93____91__m__93__" id="M_SuccinylCoA__91__d__93____91__m__93__" name="SuccinylCoA[d][m]" compartment="m" hasOnlySubstanceUnits="false" boundaryCondition="false" constant="false"/>

<species metaid="M_SuccinylCoA__91__n__93____91__m__93__" id="M_SuccinylCoA__91__n__93____91__m__93__" name="SuccinylCoA[n][m]" compartment="m" hasOnlySubstanceUnits="false" boundaryCondition="false" constant="false"/>

<species metaid="M_Sulfate__91__d__93__" id="M_Sulfate__91__d__93__" name="Sulfate[d]" compartment="d" hasOnlySubstanceUnits="false" boundaryCondition="false" constant="false"/>

<species metaid="M_Sulfate__91__d__93____91__c__93__" id="M_Sulfate__91__d__93____91__c__93__" name="Sulfate[d][c]" compartment="c" hasOnlySubstanceUnits="false" boundaryCondition="false" constant="false"/>

<species metaid="M_Sulfate__91__n__93__" id="M_Sulfate__91__n__93__" name="Sulfate[n]" compartment="n" hasOnlySubstanceUnits="false" boundaryCondition="false" constant="false"/>

<species metaid="M_Sulfate__91__n__93____91__c__93__" id="M_Sulfate__91__n__93____91__c__93__" name="Sulfate[n][c]" compartment="c" hasOnlySubstanceUnits="false" boundaryCondition="false" constant="false"/>

<species metaid="M_Sulfide__91__d__93____91__c__93__" id="M_Sulfide__91__d__93____91__c__93__" name="Sulfide[d][c]" compartment="c" hasOnlySubstanceUnits="false" boundaryCondition="false" constant="false"/>

<species metaid="M_Sulfide__91__n__93____91__c__93__" id="M_Sulfide__91__n__93____91__c__93__" name="Sulfide[n][c]" compartment="c" hasOnlySubstanceUnits="false" boundaryCondition="false" constant="false"/>

<species metaid="M_THF__91__d__93__" id="M_THF__91__d__93__" name="THF[d]" compartment="d" hasOnlySubstanceUnits="false" boundaryCondition="false" constant="false"/>

<species metaid="M_THF__91__d__93____91__m__93__" id="M_THF__91__d__93____91__m__93__" name="THF[d][m]" compartment="m" hasOnlySubstanceUnits="false" boundaryCondition="false" constant="false"/>

<species metaid="M_THF__91__n__93__" id="M_THF__91__n__93__" name="THF[n]" compartment="n" hasOnlySubstanceUnits="false" boundaryCondition="false" constant="false"/>

<species metaid="M_THF__91__n__93____91__m__93__" id="M_THF__91__n__93____91__m__93__" name="THF[n][m]" compartment="m" hasOnlySubstanceUnits="false" boundaryCondition="false" constant="false"/>

<species metaid="M_Threonine__91__d__93__" id="M_Threonine__91__d__93__" name="Threonine[d]" compartment="d" hasOnlySubstanceUnits="false" boundaryCondition="false" constant="false"/>

<species metaid="M_Threonine__91__d__93____91__c__93__" id="M_Threonine__91__d__93____91__c__93__" name="Threonine[d][c]" compartment="c" hasOnlySubstanceUnits="false" boundaryCondition="false" constant="false"/>

<species metaid="M_Threonine__91__n__93__" id="M_Threonine__91__n__93__" name="Threonine[n]" compartment="n" hasOnlySubstanceUnits="false" boundaryCondition="false" constant="false"/>

<species metaid="M_Threonine__91__n__93____91__c__93__" id="M_Threonine__91__n__93____91__c__93__" name="Threonine[n][c]" compartment="c" hasOnlySubstanceUnits="false" boundaryCondition="false" constant="false"/>

<species metaid="M_Threonine_exp__91__d__93__" id="M_Threonine_exp__91__d__93__" name="Threonine_exp[d]" compartment="d" hasOnlySubstanceUnits="false" boundaryCondition="false" constant="false"/>

<species metaid="M_Threonine_exp__91__n__93__" id="M_Threonine_exp__91__n__93__" name="Threonine_exp[n]" compartment="n" hasOnlySubstanceUnits="false" boundaryCondition="false" constant="false"/>

<species metaid="M_Tryptophan__91__d__93__" id="M_Tryptophan__91__d__93__" name="Tryptophan[d]" compartment="d" hasOnlySubstanceUnits="false" boundaryCondition="false" constant="false"/>

<species metaid="M_Tryptophan__91__d__93____91__c__93__" id="M_Tryptophan__91__d__93____91__c__93__" name="Tryptophan[d][c]" compartment="c" hasOnlySubstanceUnits="false" boundaryCondition="false" constant="false"/>

<species metaid="M_Tryptophan__91__n__93__" id="M_Tryptophan__91__n__93__" name="Tryptophan[n]" compartment="n" hasOnlySubstanceUnits="false" boundaryCondition="false" constant="false"/>

<species metaid="M_Tryptophan__91__n__93____91__c__93__" id="M_Tryptophan__91__n__93____91__c__93__" name="Tryptophan[n][c]" compartment="c" hasOnlySubstanceUnits="false" boundaryCondition="false" constant="false"/>

<species metaid="M_Tryptophan_exp__91__d__93__" id="M_Tryptophan_exp__91__d__93__" name="Tryptophan_exp[d]" compartment="d" hasOnlySubstanceUnits="false" boundaryCondition="false" constant="false"/>

<species metaid="M_Tryptophan_exp__91__n__93__" id="M_Tryptophan_exp__91__n__93__" name="Tryptophan_exp[n]" compartment="n" hasOnlySubstanceUnits="false" boundaryCondition="false" constant="false"/>

<species metaid="M_Tyrosine__91__d__93__" id="M_Tyrosine__91__d__93__" name="Tyrosine[d]" compartment="d" hasOnlySubstanceUnits="false" boundaryCondition="false" constant="false"/>

<species metaid="M_Tyrosine__91__d__93____91__c__93__" id="M_Tyrosine__91__d__93____91__c__93__" name="Tyrosine[d][c]" compartment="c" hasOnlySubstanceUnits="false" boundaryCondition="false" constant="false"/>

<species metaid="M_Tyrosine__91__n__93__" id="M_Tyrosine__91__n__93__" name="Tyrosine[n]" compartment="n" hasOnlySubstanceUnits="false" boundaryCondition="false" constant="false"/>

<species metaid="M_Tyrosine__91__n__93____91__c__93__" id="M_Tyrosine__91__n__93____91__c__93__" name="Tyrosine[n][c]" compartment="c" hasOnlySubstanceUnits="false" boundaryCondition="false" constant="false"/>

<species metaid="M_Tyrosine_exp__91__d__93__" id="M_Tyrosine_exp__91__d__93__" name="Tyrosine_exp[d]" compartment="d" hasOnlySubstanceUnits="false" boundaryCondition="false" constant="false"/>

<species metaid="M_Tyrosine_exp__91__n__93__" id="M_Tyrosine_exp__91__n__93__" name="Tyrosine_exp[n]" compartment="n" hasOnlySubstanceUnits="false" boundaryCondition="false" constant="false"/>

<species metaid="M_UDP__91__d__93__" id="M_UDP__91__d__93__" name="UDP[d]" compartment="d" hasOnlySubstanceUnits="false" boundaryCondition="false" constant="false"/>

<species metaid="M_UDP__91__n__93__" id="M_UDP__91__n__93__" name="UDP[n]" compartment="n" hasOnlySubstanceUnits="false" boundaryCondition="false" constant="false"/>

<species metaid="M_UDP_glucose__91__d__93__" id="M_UDP_glucose__91__d__93__" name="UDP_glucose[d]" compartment="d" hasOnlySubstanceUnits="false" boundaryCondition="false" constant="false"/>

<species metaid="M_UDP_glucose__91__n__93__" id="M_UDP_glucose__91__n__93__" name="UDP_glucose[n]" compartment="n" hasOnlySubstanceUnits="false" boundaryCondition="false" constant="false"/>

<species metaid="M_UMP__91__d__93__" id="M_UMP__91__d__93__" name="UMP[d]" compartment="d" hasOnlySubstanceUnits="false" boundaryCondition="false" constant="false"/>

<species metaid="M_UMP__91__n__93__" id="M_UMP__91__n__93__" name="UMP[n]" compartment="n" hasOnlySubstanceUnits="false" boundaryCondition="false" constant="false"/>

<species metaid="M_Unsaturated_Fats__91__d__93__" id="M_Unsaturated_Fats__91__d__93__" name="Unsaturated_Fats[d]" compartment="d" hasOnlySubstanceUnits="false" boundaryCondition="false" constant="false"/>

<species metaid="M_Unsaturated_Fats__91__n__93__" id="M_Unsaturated_Fats__91__n__93__" name="Unsaturated_Fats[n]" compartment="n" hasOnlySubstanceUnits="false" boundaryCondition="false" constant="false"/>

<species metaid="M_Valine__91__d__93__" id="M_Valine__91__d__93__" name="Valine[d]" compartment="d" hasOnlySubstanceUnits="false" boundaryCondition="false" constant="false"/>

<species metaid="M_Valine__91__d__93____91__c__93__" id="M_Valine__91__d__93____91__c__93__" name="Valine[d][c]" compartment="c" hasOnlySubstanceUnits="false" boundaryCondition="false" constant="false"/>

<species metaid="M_Valine__91__n__93__" id="M_Valine__91__n__93__" name="Valine[n]" compartment="n" hasOnlySubstanceUnits="false" boundaryCondition="false" constant="false"/>

<species metaid="M_Valine__91__n__93____91__c__93__" id="M_Valine__91__n__93____91__c__93__" name="Valine[n][c]" compartment="c" hasOnlySubstanceUnits="false" boundaryCondition="false" constant="false"/>

<species metaid="M_Valine_exp__91__d__93__" id="M_Valine_exp__91__d__93__" name="Valine_exp[d]" compartment="d" hasOnlySubstanceUnits="false" boundaryCondition="false" constant="false"/>

<species metaid="M_Valine_exp__91__n__93__" id="M_Valine_exp__91__n__93__" name="Valine_exp[n]" compartment="n" hasOnlySubstanceUnits="false" boundaryCondition="false" constant="false"/>

<species metaid="M_Xu5P__91__d__93__" id="M_Xu5P__91__d__93__" name="Xu5P[d]" compartment="d" hasOnlySubstanceUnits="false" boundaryCondition="false" constant="false"/>

<species metaid="M_Xu5P__91__d__93____91__c__93__" id="M_Xu5P__91__d__93____91__c__93__" name="Xu5P[d][c]" compartment="c" hasOnlySubstanceUnits="false" boundaryCondition="false" constant="false"/>

<species metaid="M_Xu5P__91__n__93__" id="M_Xu5P__91__n__93__" name="Xu5P[n]" compartment="n" hasOnlySubstanceUnits="false" boundaryCondition="false" constant="false"/>

<species metaid="M_Xu5P__91__n__93____91__c__93__" id="M_Xu5P__91__n__93____91__c__93__" name="Xu5P[n][c]" compartment="c" hasOnlySubstanceUnits="false" boundaryCondition="false" constant="false"/>

<species metaid="M_fibers__91__d__93__" id="M_fibers__91__d__93__" name="fibers[d]" compartment="d" hasOnlySubstanceUnits="false" boundaryCondition="false" constant="false"/>

<species metaid="M_fibers__91__n__93__" id="M_fibers__91__n__93__" name="fibers[n]" compartment="n" hasOnlySubstanceUnits="false" boundaryCondition="false" constant="false"/>

<species metaid="M_fructose__91__d__93__" id="M_fructose__91__d__93__" name="fructose[d]" compartment="d" hasOnlySubstanceUnits="false" boundaryCondition="false" constant="false"/>

<species metaid="M_fructose__91__n__93__" id="M_fructose__91__n__93__" name="fructose[n]" compartment="n" hasOnlySubstanceUnits="false" boundaryCondition="false" constant="false"/>

<species metaid="M_g_Linolenate__91__d__93__" id="M_g_Linolenate__91__d__93__" name="g_Linolenate[d]" compartment="d" hasOnlySubstanceUnits="false" boundaryCondition="false" constant="false"/>

<species metaid="M_g_Linolenate__91__n__93__" id="M_g_Linolenate__91__n__93__" name="g_Linolenate[n]" compartment="n" hasOnlySubstanceUnits="false" boundaryCondition="false" constant="false"/>

<species metaid="M_glucose__91__d__93__" id="M_glucose__91__d__93__" name="glucose[d]" compartment="d" hasOnlySubstanceUnits="false" boundaryCondition="false" constant="false"/>

<species metaid="M_glucose__91__d__93____91__c__93__" id="M_glucose__91__d__93____91__c__93__" name="glucose[d][c]" compartment="c" hasOnlySubstanceUnits="false" boundaryCondition="false" constant="false"/>

<species metaid="M_glucose__91__n__93__" id="M_glucose__91__n__93__" name="glucose[n]" compartment="n" hasOnlySubstanceUnits="false" boundaryCondition="false" constant="false"/>

<species metaid="M_glucose__91__n__93____91__c__93__" id="M_glucose__91__n__93____91__c__93__" name="glucose[n][c]" compartment="c" hasOnlySubstanceUnits="false" boundaryCondition="false" constant="false"/>

<species metaid="M_h700__91__c__93__" id="M_h700__91__c__93__" name="h700[c]" compartment="c" hasOnlySubstanceUnits="false" boundaryCondition="false" constant="false"/>

<species metaid="M_h700_n__91__c__93__" id="M_h700_n__91__c__93__" name="h700_n[c]" compartment="c" hasOnlySubstanceUnits="false" boundaryCondition="false" constant="false"/>

<species metaid="M_hydroxypyruvate__91__d__93____91__p__93__" id="M_hydroxypyruvate__91__d__93____91__p__93__" name="hydroxypyruvate[d][p]" compartment="p" hasOnlySubstanceUnits="false" boundaryCondition="false" constant="false"/>

<species metaid="M_hydroxypyruvate__91__n__93____91__p__93__" id="M_hydroxypyruvate__91__n__93____91__p__93__" name="hydroxypyruvate[n][p]" compartment="p" hasOnlySubstanceUnits="false" boundaryCondition="false" constant="false"/>

<species metaid="M_sucrose__91__d__93__" id="M_sucrose__91__d__93__" name="sucrose[d]" compartment="d" hasOnlySubstanceUnits="false" boundaryCondition="false" constant="false"/>

<species metaid="M_sucrose__91__n__93__" id="M_sucrose__91__n__93__" name="sucrose[n]" compartment="n" hasOnlySubstanceUnits="false" boundaryCondition="false" constant="false"/>

<species metaid="M_sucrose_exp__91__d__93__" id="M_sucrose_exp__91__d__93__" name="sucrose_exp[d]" compartment="d" hasOnlySubstanceUnits="false" boundaryCondition="false" constant="false"/>

<species metaid="M_sucrose_exp__91__n__93__" id="M_sucrose_exp__91__n__93__" name="sucrose_exp[n]" compartment="n" hasOnlySubstanceUnits="false" boundaryCondition="false" constant="false"/>

<species metaid="M_sugars__91__d__93__" id="M_sugars__91__d__93__" name="sugars[d]" compartment="d" hasOnlySubstanceUnits="false" boundaryCondition="false" constant="false"/>

<species metaid="M_sugars__91__n__93__" id="M_sugars__91__n__93__" name="sugars[n]" compartment="n" hasOnlySubstanceUnits="false" boundaryCondition="false" constant="false"/>

<species metaid="M_triglycerids__91__d__93__" id="M_triglycerids__91__d__93__" name="triglycerids[d]" compartment="d" hasOnlySubstanceUnits="false" boundaryCondition="false" constant="false"/>

<species metaid="M_triglycerids__91__n__93__" id="M_triglycerids__91__n__93__" name="triglycerids[n]" compartment="n" hasOnlySubstanceUnits="false" boundaryCondition="false" constant="false"/>

</listOfSpecies>

<listOfParameters>

<parameter sboTerm="SBO:0000626" id="FB1N1000" value="-1000" constant="true"/>

<parameter sboTerm="SBO:0000626" id="FB2N0" value="-0" constant="true"/>

<parameter sboTerm="SBO:0000626" id="FB3N1000" value="1000" constant="true"/>

</listOfParameters>

<listOfReactions>

<reaction metaid="Gly3ptr" id="Gly3ptr" name="Gly3ptr" reversible="true" fast="false" fbc:lowerFluxBound="FB1N1000" fbc:upperFluxBound="FB3N1000">

<listOfReactants>

<speciesReference species="M_Glycerol3P__91__d__93____91__m__93__" stoichiometry="1" constant="true"/>

</listOfReactants>

<listOfProducts>

<speciesReference species="M_Glycerol3P__91__d__93__" stoichiometry="1" constant="true"/>

</listOfProducts>

</reaction>

<reaction metaid="DHAPtr" id="DHAPtr" name="DHAPtr" reversible="true" fast="false" fbc:lowerFluxBound="FB1N1000" fbc:upperFluxBound="FB3N1000">

<listOfReactants>

<speciesReference species="M_DHAP__91__d__93____91__m__93__" stoichiometry="1" constant="true"/>

</listOfReactants>

<listOfProducts>

<speciesReference species="M_DHAP__91__d__93__" stoichiometry="1" constant="true"/>

</listOfProducts>

</reaction>

<reaction metaid="Gly3ptr_n" id="Gly3ptr_n" name="Gly3ptr_n" reversible="true" fast="false" fbc:lowerFluxBound="FB1N1000" fbc:upperFluxBound="FB3N1000">

<listOfReactants>

<speciesReference species="M_Glycerol3P__91__n__93____91__m__93__" stoichiometry="1" constant="true"/>

</listOfReactants>

<listOfProducts>

<speciesReference species="M_Glycerol3P__91__n__93__" stoichiometry="1" constant="true"/>

</listOfProducts>

</reaction>

<reaction metaid="DHAPtr_n" id="DHAPtr_n" name="DHAPtr_n" reversible="true" fast="false" fbc:lowerFluxBound="FB1N1000" fbc:upperFluxBound="FB3N1000">

<listOfReactants>

<speciesReference species="M_DHAP__91__n__93____91__m__93__" stoichiometry="1" constant="true"/>

</listOfReactants>

<listOfProducts>

<speciesReference species="M_DHAP__91__n__93__" stoichiometry="1" constant="true"/>

</listOfProducts>

</reaction>

<reaction metaid="NADconv_mit" id="NADconv_mit" name="NADconv_mit" reversible="true" fast="false" fbc:lowerFluxBound="FB1N1000" fbc:upperFluxBound="FB3N1000">

<listOfReactants>

<speciesReference species="M_NADPH__91__d__93____91__m__93__" stoichiometry="1" constant="true"/>

<speciesReference species="M_NAD__91__d__93____91__m__93__" stoichiometry="1" constant="true"/>

</listOfReactants>

<listOfProducts>

<speciesReference species="M_NADH__91__d__93____91__m__93__" stoichiometry="1" constant="true"/>

<speciesReference species="M_NADP__91__d__93____91__m__93__" stoichiometry="1" constant="true"/>

</listOfProducts>

</reaction>

<reaction metaid="NADconv_mit_n" id="NADconv_mit_n" name="NADconv_mit_n" reversible="true" fast="false" fbc:lowerFluxBound="FB1N1000" fbc:upperFluxBound="FB3N1000">

<listOfReactants>

<speciesReference species="M_NADPH__91__n__93____91__m__93__" stoichiometry="1" constant="true"/>

<speciesReference species="M_NAD__91__n__93____91__m__93__" stoichiometry="1" constant="true"/>

</listOfReactants>

<listOfProducts>

<speciesReference species="M_NADH__91__n__93____91__m__93__" stoichiometry="1" constant="true"/>

<speciesReference species="M_NADP__91__n__93____91__m__93__" stoichiometry="1" constant="true"/>

</listOfProducts>

</reaction>

<reaction metaid="NADconv" id="NADconv" name="NADconv" reversible="true" fast="false" fbc:lowerFluxBound="FB1N1000" fbc:upperFluxBound="FB3N1000">

<listOfReactants>

<speciesReference species="M_NADPH__91__d__93__" stoichiometry="1" constant="true"/>

<speciesReference species="M_NAD__91__d__93__" stoichiometry="1" constant="true"/>

</listOfReactants>

<listOfProducts>

<speciesReference species="M_NADH__91__d__93__" stoichiometry="1" constant="true"/>

<speciesReference species="M_NADP__91__d__93__" stoichiometry="1" constant="true"/>

</listOfProducts>

</reaction>

<reaction metaid="NADconv_n" id="NADconv_n" name="NADconv_n" reversible="true" fast="false" fbc:lowerFluxBound="FB1N1000" fbc:upperFluxBound="FB3N1000">

<listOfReactants>

<speciesReference species="M_NADPH__91__n__93__" stoichiometry="1" constant="true"/>

<speciesReference species="M_NAD__91__n__93__" stoichiometry="1" constant="true"/>

</listOfReactants>

<listOfProducts>

<speciesReference species="M_NADH__91__n__93__" stoichiometry="1" constant="true"/>

<speciesReference species="M_NADP__91__n__93__" stoichiometry="1" constant="true"/>

</listOfProducts>

</reaction>

<reaction metaid="NADconv_cl" id="NADconv_cl" name="NADconv_cl" reversible="true" fast="false" fbc:lowerFluxBound="FB1N1000" fbc:upperFluxBound="FB3N1000">

<listOfReactants>

<speciesReference species="M_NADPH__91__d__93____91__c__93__" stoichiometry="1" constant="true"/>

<speciesReference species="M_NAD__91__d__93____91__c__93__" stoichiometry="1" constant="true"/>

</listOfReactants>

<listOfProducts>

<speciesReference species="M_NADH__91__d__93____91__c__93__" stoichiometry="1" constant="true"/>

<speciesReference species="M_NADP__91__d__93____91__c__93__" stoichiometry="1" constant="true"/>

</listOfProducts>

</reaction>

<reaction metaid="NADconv_cl_n" id="NADconv_cl_n" name="NADconv_cl_n" reversible="true" fast="false" fbc:lowerFluxBound="FB1N1000" fbc:upperFluxBound="FB3N1000">

<listOfReactants>

<speciesReference species="M_NADPH__91__n__93____91__c__93__" stoichiometry="1" constant="true"/>

<speciesReference species="M_NAD__91__n__93____91__c__93__" stoichiometry="1" constant="true"/>

</listOfReactants>

<listOfProducts>

<speciesReference species="M_NADH__91__n__93____91__c__93__" stoichiometry="1" constant="true"/>

<speciesReference species="M_NADP__91__n__93____91__c__93__" stoichiometry="1" constant="true"/>

</listOfProducts>

</reaction>

<reaction metaid="Ex_CO2" id="Ex_CO2" name="Ex_CO2" reversible="true" fast="false" fbc:lowerFluxBound="FB1N1000" fbc:upperFluxBound="FB3N1000">

<listOfReactants>

<speciesReference species="M_CO2__91__d__93__" stoichiometry="1" constant="true"/>

</listOfReactants>

</reaction>

<reaction metaid="Ex_e" id="Ex_e" name="Ex_e" reversible="true" fast="false" fbc:lowerFluxBound="FB1N1000" fbc:upperFluxBound="FB3N1000">

<listOfReactants>

<speciesReference species="M_h700__91__c__93__" stoichiometry="1" constant="true"/>

</listOfReactants>

</reaction>

<reaction metaid="Ex_O2" id="Ex_O2" name="Ex_O2" reversible="true" fast="false" fbc:lowerFluxBound="FB1N1000" fbc:upperFluxBound="FB3N1000">

<listOfReactants>

<speciesReference species="M_O2__91__d__93__" stoichiometry="1" constant="true"/>

</listOfReactants>

</reaction>

<reaction metaid="Ex_H2O" id="Ex_H2O" name="Ex_H2O" reversible="true" fast="false" fbc:lowerFluxBound="FB1N1000" fbc:upperFluxBound="FB3N1000">

<listOfReactants>

<speciesReference species="M_H2O__91__d__93__" stoichiometry="1" constant="true"/>

</listOfReactants>

</reaction>

<reaction metaid="Ex_HNO3" id="Ex_HNO3" name="Ex_HNO3" reversible="true" fast="false" fbc:lowerFluxBound="FB1N1000" fbc:upperFluxBound="FB3N1000">

<listOfReactants>

<speciesReference species="M_HNO3__91__d__93__" stoichiometry="1" constant="true"/>

</listOfReactants>

</reaction>

<reaction metaid="Ex_Biomass" id="Ex_Biomass" name="Ex_Biomass" reversible="true" fast="false" fbc:lowerFluxBound="FB1N1000" fbc:upperFluxBound="FB3N1000">

<listOfReactants>

<speciesReference species="M_Biomass__91__d__93__" stoichiometry="1" constant="true"/>

</listOfReactants>

</reaction>

<reaction metaid="Ex_H2S" id="Ex_H2S" name="Ex_H2S" reversible="true" fast="false" fbc:lowerFluxBound="FB1N1000" fbc:upperFluxBound="FB3N1000">

<listOfReactants>

<speciesReference species="M_H2S__91__d__93__" stoichiometry="1" constant="true"/>

</listOfReactants>

</reaction>

<reaction metaid="Ex_Sulfate" id="Ex_Sulfate" name="Ex_Sulfate" reversible="true" fast="false" fbc:lowerFluxBound="FB1N1000" fbc:upperFluxBound="FB3N1000">

<listOfReactants>

<speciesReference species="M_Sulfate__91__d__93__" stoichiometry="1" constant="true"/>

</listOfReactants>

</reaction>

<reaction metaid="Exp_sucrosePhloemDay" id="Exp_sucrosePhloemDay" name="Exp_sucrosePhloemDay" reversible="false" fast="false" fbc:lowerFluxBound="FB2N0" fbc:upperFluxBound="FB3N1000">

<listOfReactants>

<speciesReference species="M_sucrose_exp__91__d__93__" stoichiometry="1" constant="true"/>

</listOfReactants>

</reaction>

<reaction metaid="Exp_AAPhloemDay" id="Exp_AAPhloemDay" name="Exp_AAPhloemDay" reversible="false" fast="false" fbc:lowerFluxBound="FB2N0" fbc:upperFluxBound="FB3N1000">

<listOfReactants>

<speciesReference species="M_AA_exp__91__d__93__" stoichiometry="1" constant="true"/>

</listOfReactants>

</reaction>

<reaction metaid="Asn_DayPhloeamExp" id="Asn_DayPhloeamExp" name="Asn_DayPhloeamExp" reversible="false" fast="false" fbc:lowerFluxBound="FB2N0" fbc:upperFluxBound="FB3N1000">

<listOfReactants>

<speciesReference species="M_Asparagine__91__d__93__" stoichiometry="1" constant="true"/>

</listOfReactants>

<listOfProducts>

<speciesReference species="M_Asparagine_exp__91__d__93__" stoichiometry="1" constant="true"/>

</listOfProducts>

</reaction>

<reaction metaid="Asp_DayPhloemExp" id="Asp_DayPhloemExp" name="Asp_DayPhloemExp" reversible="false" fast="false" fbc:lowerFluxBound="FB2N0" fbc:upperFluxBound="FB3N1000">

<listOfReactants>

<speciesReference species="M_Aspartate__91__d__93__" stoichiometry="1" constant="true"/>

</listOfReactants>

<listOfProducts>

<speciesReference species="M_Aspartate_exp__91__d__93__" stoichiometry="1" constant="true"/>

</listOfProducts>

</reaction>

<reaction metaid="Thr_DayPhloeamExp" id="Thr_DayPhloeamExp" name="Thr_DayPhloeamExp" reversible="false" fast="false" fbc:lowerFluxBound="FB2N0" fbc:upperFluxBound="FB3N1000">

<listOfReactants>

<speciesReference species="M_Threonine__91__d__93__" stoichiometry="1" constant="true"/>

</listOfReactants>

<listOfProducts>

<speciesReference species="M_Threonine_exp__91__d__93__" stoichiometry="1" constant="true"/>

</listOfProducts>

</reaction>

<reaction metaid="Gln_Day_Phloem_Exp" id="Gln_Day_Phloem_Exp" name="Gln_Day_Phloem_Exp" reversible="false" fast="false" fbc:lowerFluxBound="FB2N0" fbc:upperFluxBound="FB3N1000">

<listOfReactants>

<speciesReference species="M_Glutamate__91__d__93__" stoichiometry="1" constant="true"/>

</listOfReactants>

<listOfProducts>

<speciesReference species="M_Glutamate_exp__91__d__93__" stoichiometry="1" constant="true"/>

</listOfProducts>

</reaction>

<reaction metaid="Iso_DayPhloemExp" id="Iso_DayPhloemExp" name="Iso_DayPhloemExp" reversible="false" fast="false" fbc:lowerFluxBound="FB2N0" fbc:upperFluxBound="FB3N1000">

<listOfReactants>

<speciesReference species="M_Isoleucine__91__d__93__" stoichiometry="1" constant="true"/>

</listOfReactants>

<listOfProducts>

<speciesReference species="M_Isoleucine_exp__91__d__93__" stoichiometry="1" constant="true"/>

</listOfProducts>

</reaction>

<reaction metaid="Val_DayPhloemExp" id="Val_DayPhloemExp" name="Val_DayPhloemExp" reversible="false" fast="false" fbc:lowerFluxBound="FB2N0" fbc:upperFluxBound="FB3N1000">

<listOfReactants>

<speciesReference species="M_Valine__91__d__93__" stoichiometry="1" constant="true"/>

</listOfReactants>

<listOfProducts>

<speciesReference species="M_Valine_exp__91__d__93__" stoichiometry="1" constant="true"/>

</listOfProducts>

</reaction>

<reaction metaid="Ala_DayPhloemExp" id="Ala_DayPhloemExp" name="Ala_DayPhloemExp" reversible="false" fast="false" fbc:lowerFluxBound="FB2N0" fbc:upperFluxBound="FB3N1000">

<listOfReactants>

<speciesReference species="M_Alanine__91__d__93__" stoichiometry="1" constant="true"/>

</listOfReactants>

<listOfProducts>

<speciesReference species="M_Alanine_exp__91__d__93__" stoichiometry="1" constant="true"/>

</listOfProducts>

</reaction>

<reaction metaid="Leu_DayPhloemExp" id="Leu_DayPhloemExp" name="Leu_DayPhloemExp" reversible="false" fast="false" fbc:lowerFluxBound="FB2N0" fbc:upperFluxBound="FB3N1000">

<listOfReactants>

<speciesReference species="M_Leucine__91__d__93__" stoichiometry="1" constant="true"/>

</listOfReactants>

<listOfProducts>

<speciesReference species="M_Leucine_exp__91__d__93__" stoichiometry="1" constant="true"/>

</listOfProducts>

</reaction>

<reaction metaid="Ser_DayPhloemExp" id="Ser_DayPhloemExp" name="Ser_DayPhloemExp" reversible="false" fast="false" fbc:lowerFluxBound="FB2N0" fbc:upperFluxBound="FB3N1000">

<listOfReactants>

<speciesReference species="M_Serine__91__d__93__" stoichiometry="1" constant="true"/>

</listOfReactants>

<listOfProducts>

<speciesReference species="M_Serine_exp__91__d__93__" stoichiometry="1" constant="true"/>

</listOfProducts>

</reaction>

<reaction metaid="Gly_Day_PhloemExp" id="Gly_Day_PhloemExp" name="Gly_Day_PhloemExp" reversible="false" fast="false" fbc:lowerFluxBound="FB2N0" fbc:upperFluxBound="FB3N1000">

<listOfReactants>

<speciesReference species="M_Glycine__91__d__93__" stoichiometry="1" constant="true"/>

</listOfReactants>

<listOfProducts>

<speciesReference species="M_Glycine_exp__91__d__93__" stoichiometry="1" constant="true"/>

</listOfProducts>

</reaction>

<reaction metaid="Glu_DayPhloemExp" id="Glu_DayPhloemExp" name="Glu_DayPhloemExp" reversible="false" fast="false" fbc:lowerFluxBound="FB2N0" fbc:upperFluxBound="FB3N1000">

<listOfReactants>

<speciesReference species="M_Glutamine__91__d__93__" stoichiometry="1" constant="true"/>

</listOfReactants>

<listOfProducts>

<speciesReference species="M_Glutamine_exp__91__d__93__" stoichiometry="1" constant="true"/>

</listOfProducts>

</reaction>

<reaction metaid="Arg_DayPhloemExp" id="Arg_DayPhloemExp" name="Arg_DayPhloemExp" reversible="false" fast="false" fbc:lowerFluxBound="FB2N0" fbc:upperFluxBound="FB3N1000">

<listOfReactants>

<speciesReference species="M_Arginine__91__d__93__" stoichiometry="1" constant="true"/>

</listOfReactants>

<listOfProducts>

<speciesReference species="M_Arginine_exp__91__d__93__" stoichiometry="1" constant="true"/>

</listOfProducts>

</reaction>

<reaction metaid="Tyr_DayPhloemExp" id="Tyr_DayPhloemExp" name="Tyr_DayPhloemExp" reversible="false" fast="false" fbc:lowerFluxBound="FB2N0" fbc:upperFluxBound="FB3N1000">

<listOfReactants>

<speciesReference species="M_Tyrosine__91__d__93__" stoichiometry="1" constant="true"/>

</listOfReactants>

<listOfProducts>

<speciesReference species="M_Tyrosine_exp__91__d__93__" stoichiometry="1" constant="true"/>

</listOfProducts>

</reaction>

<reaction metaid="Phe_DayPhloemExp" id="Phe_DayPhloemExp" name="Phe_DayPhloemExp" reversible="false" fast="false" fbc:lowerFluxBound="FB2N0" fbc:upperFluxBound="FB3N1000">

<listOfReactants>

<speciesReference species="M_Phenylalanine__91__d__93__" stoichiometry="1" constant="true"/>

</listOfReactants>

<listOfProducts>

<speciesReference species="M_Phenylalanine_exp__91__d__93__" stoichiometry="1" constant="true"/>

</listOfProducts>

</reaction>

<reaction metaid="Try_DayPhloemExp" id="Try_DayPhloemExp" name="Try_DayPhloemExp" reversible="false" fast="false" fbc:lowerFluxBound="FB2N0" fbc:upperFluxBound="FB3N1000">

<listOfReactants>

<speciesReference species="M_Tryptophan__91__d__93__" stoichiometry="1" constant="true"/>

</listOfReactants>

<listOfProducts>

<speciesReference species="M_Tryptophan_exp__91__d__93__" stoichiometry="1" constant="true"/>

</listOfProducts>

</reaction>

<reaction metaid="Lys_DayPhloemExp" id="Lys_DayPhloemExp" name="Lys_DayPhloemExp" reversible="false" fast="false" fbc:lowerFluxBound="FB2N0" fbc:upperFluxBound="FB3N1000">

<listOfReactants>

<speciesReference species="M_Lysine__91__d__93__" stoichiometry="1" constant="true"/>

</listOfReactants>

<listOfProducts>

<speciesReference species="M_Lysine_exp__91__d__93__" stoichiometry="1" constant="true"/>

</listOfProducts>

</reaction>

<reaction metaid="His_DayPhloemExp" id="His_DayPhloemExp" name="His_DayPhloemExp" reversible="false" fast="false" fbc:lowerFluxBound="FB2N0" fbc:upperFluxBound="FB3N1000">

<listOfReactants>

<speciesReference species="M_Histidine__91__d__93__" stoichiometry="1" constant="true"/>

</listOfReactants>

<listOfProducts>

<speciesReference species="M_Histidine_exp__91__d__93__" stoichiometry="1" constant="true"/>

</listOfProducts>

</reaction>

<reaction metaid="Met_DayPhloemExp" id="Met_DayPhloemExp" name="Met_DayPhloemExp" reversible="false" fast="false" fbc:lowerFluxBound="FB2N0" fbc:upperFluxBound="FB3N1000">

<listOfReactants>

<speciesReference species="M_Methionine__91__d__93__" stoichiometry="1" constant="true"/>

</listOfReactants>

<listOfProducts>

<speciesReference species="M_Methionine_exp__91__d__93__" stoichiometry="1" constant="true"/>

</listOfProducts>

</reaction>

<reaction metaid="Suc_DayPhloem_Exp" id="Suc_DayPhloem_Exp" name="Suc_DayPhloem_Exp" reversible="false" fast="false" fbc:lowerFluxBound="FB2N0" fbc:upperFluxBound="FB3N1000">

<listOfReactants>

<speciesReference species="M_sucrose__91__d__93__" stoichiometry="1" constant="true"/>

</listOfReactants>

<listOfProducts>

<speciesReference species="M_sucrose_exp__91__d__93__" stoichiometry="1" constant="true"/>

</listOfProducts>

</reaction>

<reaction metaid="AminoAcidExport_Day" id="AminoAcidExport_Day" name="AminoAcidExport_Day" reversible="false" fast="false" fbc:lowerFluxBound="FB2N0" fbc:upperFluxBound="FB3N1000">

<listOfReactants>

<speciesReference species="M_Alanine_exp__91__d__93__" stoichiometry="9.7" constant="true"/>

<speciesReference species="M_Arginine_exp__91__d__93__" stoichiometry="1.6" constant="true"/>

<speciesReference species="M_Asparagine_exp__91__d__93__" stoichiometry="10.1" constant="true"/>

<speciesReference species="M_Aspartate_exp__91__d__93__" stoichiometry="9.5" constant="true"/>

<speciesReference species="M_Glutamate_exp__91__d__93__" stoichiometry="11.4" constant="true"/>

<speciesReference species="M_Glutamine_exp__91__d__93__" stoichiometry="33.2" constant="true"/>

<speciesReference species="M_Glycine_exp__91__d__93__" stoichiometry="0.7" constant="true"/>

<speciesReference species="M_Histidine_exp__91__d__93__" stoichiometry="0.4" constant="true"/>

<speciesReference species="M_Isoleucine_exp__91__d__93__" stoichiometry="1.2" constant="true"/>

<speciesReference species="M_Leucine_exp__91__d__93__" stoichiometry="1.2" constant="true"/>

<speciesReference species="M_Lysine_exp__91__d__93__" stoichiometry="1.8" constant="true"/>

<speciesReference species="M_Methionine_exp__91__d__93__" stoichiometry="0.7" constant="true"/>

<speciesReference species="M_Phenylalanine_exp__91__d__93__" stoichiometry="1.1" constant="true"/>

<speciesReference species="M_Serine_exp__91__d__93__" stoichiometry="7.7" constant="true"/>

<speciesReference species="M_Threonine_exp__91__d__93__" stoichiometry="5.6" constant="true"/>

<speciesReference species="M_Tryptophan_exp__91__d__93__" stoichiometry="0.9" constant="true"/>

<speciesReference species="M_Tyrosine_exp__91__d__93__" stoichiometry="0.6" constant="true"/>

<speciesReference species="M_Valine_exp__91__d__93__" stoichiometry="2.4" constant="true"/>

</listOfReactants>

<listOfProducts>

<speciesReference species="M_AA_exp__91__d__93__" stoichiometry="1" constant="true"/>

</listOfProducts>

</reaction>

<reaction metaid="Thr_Cl_export" id="Thr_Cl_export" name="Thr_Cl_export" reversible="true" fast="false" fbc:lowerFluxBound="FB1N1000" fbc:upperFluxBound="FB3N1000">

<listOfReactants>

<speciesReference species="M_Threonine__91__d__93____91__c__93__" stoichiometry="1" constant="true"/>

</listOfReactants>

<listOfProducts>

<speciesReference species="M_Threonine__91__d__93__" stoichiometry="1" constant="true"/>

</listOfProducts>

</reaction>

<reaction metaid="Iso_cl_export" id="Iso_cl_export" name="Iso_cl_export" reversible="true" fast="false" fbc:lowerFluxBound="FB1N1000" fbc:upperFluxBound="FB3N1000">

<listOfReactants>

<speciesReference species="M_Isoleucine__91__d__93____91__c__93__" stoichiometry="1" constant="true"/>

</listOfReactants>

<listOfProducts>

<speciesReference species="M_Isoleucine__91__d__93__" stoichiometry="1" constant="true"/>

</listOfProducts>

</reaction>

<reaction metaid="Val_cl_export" id="Val_cl_export" name="Val_cl_export" reversible="true" fast="false" fbc:lowerFluxBound="FB1N1000" fbc:upperFluxBound="FB3N1000">

<listOfReactants>

<speciesReference species="M_Valine__91__d__93____91__c__93__" stoichiometry="1" constant="true"/>

</listOfReactants>

<listOfProducts>

<speciesReference species="M_Valine__91__d__93__" stoichiometry="1" constant="true"/>

</listOfProducts>

</reaction>

<reaction metaid="Ala_cl_export" id="Ala_cl_export" name="Ala_cl_export" reversible="true" fast="false" fbc:lowerFluxBound="FB1N1000" fbc:upperFluxBound="FB3N1000">

<listOfReactants>

<speciesReference species="M_Alanine__91__d__93____91__c__93__" stoichiometry="1" constant="true"/>

</listOfReactants>

<listOfProducts>

<speciesReference species="M_Alanine__91__d__93__" stoichiometry="1" constant="true"/>

</listOfProducts>

</reaction>

<reaction metaid="Leu_cl_export" id="Leu_cl_export" name="Leu_cl_export" reversible="true" fast="false" fbc:lowerFluxBound="FB1N1000" fbc:upperFluxBound="FB3N1000">

<listOfReactants>

<speciesReference species="M_Leucine__91__d__93____91__c__93__" stoichiometry="1" constant="true"/>

</listOfReactants>

<listOfProducts>

<speciesReference species="M_Leucine__91__d__93__" stoichiometry="1" constant="true"/>

</listOfProducts>

</reaction>

<reaction metaid="Cys_cl_exp" id="Cys_cl_exp" name="Cys_cl_exp" reversible="true" fast="false" fbc:lowerFluxBound="FB1N1000" fbc:upperFluxBound="FB3N1000">

<listOfReactants>

<speciesReference species="M_Cystine__91__d__93____91__c__93__" stoichiometry="1" constant="true"/>

</listOfReactants>

<listOfProducts>

<speciesReference species="M_Cystine__91__d__93__" stoichiometry="1" constant="true"/>

</listOfProducts>

</reaction>

<reaction metaid="Gln_cl_export" id="Gln_cl_export" name="Gln_cl_export" reversible="false" fast="false" fbc:lowerFluxBound="FB2N0" fbc:upperFluxBound="FB3N1000">

<listOfReactants>

<speciesReference species="M_Glutamine__91__d__93__" stoichiometry="1" constant="true"/>

</listOfReactants>

<listOfProducts>

<speciesReference species="M_Glutamine__91__d__93____91__c__93__" stoichiometry="1" constant="true"/>

</listOfProducts>

</reaction>

<reaction metaid="Glu_cl_export" id="Glu_cl_export" name="Glu_cl_export" reversible="true" fast="false" fbc:lowerFluxBound="FB1N1000" fbc:upperFluxBound="FB3N1000">

<listOfReactants>

<speciesReference species="M_Glutamate__91__d__93____91__c__93__" stoichiometry="1" constant="true"/>

</listOfReactants>

<listOfProducts>

<speciesReference species="M_Glutamate__91__d__93__" stoichiometry="1" constant="true"/>

</listOfProducts>

</reaction>

<reaction metaid="Glu_cl_per_export" id="Glu_cl_per_export" name="Glu_cl_per_export" reversible="true" fast="false" fbc:lowerFluxBound="FB1N1000" fbc:upperFluxBound="FB3N1000">

<listOfReactants>

<speciesReference species="M_Glutamate__91__d__93____91__p__93__" stoichiometry="1" constant="true"/>

</listOfReactants>

<listOfProducts>

<speciesReference species="M_Glutamate__91__d__93__" stoichiometry="1" constant="true"/>

</listOfProducts>

</reaction>

<reaction metaid="Pro_cl_export" id="Pro_cl_export" name="Pro_cl_export" reversible="true" fast="false" fbc:lowerFluxBound="FB1N1000" fbc:upperFluxBound="FB3N1000">

<listOfReactants>

<speciesReference species="M_Proline__91__d__93____91__c__93__" stoichiometry="1" constant="true"/>

</listOfReactants>

<listOfProducts>

<speciesReference species="M_Proline__91__d__93__" stoichiometry="1" constant="true"/>

</listOfProducts>

</reaction>

<reaction metaid="Arg_cl_export" id="Arg_cl_export" name="Arg_cl_export" reversible="true" fast="false" fbc:lowerFluxBound="FB1N1000" fbc:upperFluxBound="FB3N1000">

<listOfReactants>

<speciesReference species="M_Arginine__91__d__93____91__c__93__" stoichiometry="1" constant="true"/>

</listOfReactants>

<listOfProducts>

<speciesReference species="M_Arginine__91__d__93__" stoichiometry="1" constant="true"/>

</listOfProducts>

</reaction>

<reaction metaid="Tyr_cl_export" id="Tyr_cl_export" name="Tyr_cl_export" reversible="true" fast="false" fbc:lowerFluxBound="FB1N1000" fbc:upperFluxBound="FB3N1000">

<listOfReactants>

<speciesReference species="M_Tyrosine__91__d__93____91__c__93__" stoichiometry="1" constant="true"/>

</listOfReactants>

<listOfProducts>

<speciesReference species="M_Tyrosine__91__d__93__" stoichiometry="1" constant="true"/>

</listOfProducts>

</reaction>

<reaction metaid="Phe_cl_export" id="Phe_cl_export" name="Phe_cl_export" reversible="true" fast="false" fbc:lowerFluxBound="FB1N1000" fbc:upperFluxBound="FB3N1000">

<listOfReactants>

<speciesReference species="M_Phenylalanine__91__d__93____91__c__93__" stoichiometry="1" constant="true"/>

</listOfReactants>

<listOfProducts>

<speciesReference species="M_Phenylalanine__91__d__93__" stoichiometry="1" constant="true"/>

</listOfProducts>

</reaction>

<reaction metaid="Trp_cl_export" id="Trp_cl_export" name="Trp_cl_export" reversible="true" fast="false" fbc:lowerFluxBound="FB1N1000" fbc:upperFluxBound="FB3N1000">

<listOfReactants>

<speciesReference species="M_Tryptophan__91__d__93____91__c__93__" stoichiometry="1" constant="true"/>

</listOfReactants>

<listOfProducts>

<speciesReference species="M_Tryptophan__91__d__93__" stoichiometry="1" constant="true"/>

</listOfProducts>

</reaction>

<reaction metaid="His_cl_export" id="His_cl_export" name="His_cl_export" reversible="true" fast="false" fbc:lowerFluxBound="FB1N1000" fbc:upperFluxBound="FB3N1000">

<listOfReactants>

<speciesReference species="M_Histidine__91__d__93____91__c__93__" stoichiometry="1" constant="true"/>

</listOfReactants>

<listOfProducts>

<speciesReference species="M_Histidine__91__d__93__" stoichiometry="1" constant="true"/>

</listOfProducts>

</reaction>

<reaction metaid="Met_cl_export" id="Met_cl_export" name="Met_cl_export" reversible="true" fast="false" fbc:lowerFluxBound="FB1N1000" fbc:upperFluxBound="FB3N1000">

<listOfReactants>

<speciesReference species="M_Homocysteine__91__d__93____91__c__93__" stoichiometry="1" constant="true"/>

</listOfReactants>

<listOfProducts>

<speciesReference species="M_Homocysteine__91__d__93__" stoichiometry="1" constant="true"/>

</listOfProducts>

</reaction>

<reaction metaid="Ser_cl_export" id="Ser_cl_export" name="Ser_cl_export" reversible="true" fast="false" fbc:lowerFluxBound="FB1N1000" fbc:upperFluxBound="FB3N1000">

<listOfReactants>

<speciesReference species="M_Serine__91__d__93____91__c__93__" stoichiometry="1" constant="true"/>

</listOfReactants>

<listOfProducts>

<speciesReference species="M_Serine__91__d__93__" stoichiometry="1" constant="true"/>

</listOfProducts>

</reaction>

<reaction metaid="Lys_cl_exp" id="Lys_cl_exp" name="Lys_cl_exp" reversible="true" fast="false" fbc:lowerFluxBound="FB1N1000" fbc:upperFluxBound="FB3N1000">

<listOfReactants>

<speciesReference species="M_Lysine__91__d__93____91__c__93__" stoichiometry="1" constant="true"/>

</listOfReactants>

<listOfProducts>

<speciesReference species="M_Lysine__91__d__93__" stoichiometry="1" constant="true"/>

</listOfProducts>

</reaction>

<reaction metaid="Cysteine_cl_exp" id="Cysteine_cl_exp" name="Cysteine_cl_exp" reversible="true" fast="false" fbc:lowerFluxBound="FB1N1000" fbc:upperFluxBound="FB3N1000">

<listOfReactants>

<speciesReference species="M_Cysteine__91__d__93____91__c__93__" stoichiometry="1" constant="true"/>

</listOfReactants>

<listOfProducts>

<speciesReference species="M_Cysteine__91__d__93__" stoichiometry="1" constant="true"/>

</listOfProducts>

</reaction>

<reaction metaid="Gly_exp" id="Gly_exp" name="Gly_exp" reversible="true" fast="false" fbc:lowerFluxBound="FB1N1000" fbc:upperFluxBound="FB3N1000">

<listOfReactants>

<speciesReference species="M_Glycine__91__d__93____91__m__93__" stoichiometry="1" constant="true"/>

</listOfReactants>

<listOfProducts>

<speciesReference species="M_Glycine__91__d__93__" stoichiometry="1" constant="true"/>

</listOfProducts>

</reaction>

<reaction metaid="Ser_exp" id="Ser_exp" name="Ser_exp" reversible="true" fast="false" fbc:lowerFluxBound="FB1N1000" fbc:upperFluxBound="FB3N1000">

<listOfReactants>

<speciesReference species="M_Serine__91__d__93____91__m__93__" stoichiometry="1" constant="true"/>

</listOfReactants>

<listOfProducts>

<speciesReference species="M_Serine__91__d__93__" stoichiometry="1" constant="true"/>

</listOfProducts>

</reaction>

<reaction metaid="Gly_cl_exp" id="Gly_cl_exp" name="Gly_cl_exp" reversible="true" fast="false" fbc:lowerFluxBound="FB1N1000" fbc:upperFluxBound="FB3N1000">

<listOfReactants>

<speciesReference species="M_Glycine__91__d__93____91__c__93__" stoichiometry="1" constant="true"/>

</listOfReactants>

<listOfProducts>

<speciesReference species="M_Glycine__91__d__93__" stoichiometry="1" constant="true"/>

</listOfProducts>

</reaction>

<reaction metaid="Sulfate_cl_trans" id="Sulfate_cl_trans" name="Sulfate_cl_trans" reversible="true" fast="false" fbc:lowerFluxBound="FB1N1000" fbc:upperFluxBound="FB3N1000">

<listOfReactants>

<speciesReference species="M_Sulfate__91__d__93____91__c__93__" stoichiometry="1" constant="true"/>

</listOfReactants>

<listOfProducts>

<speciesReference species="M_Sulfate__91__d__93__" stoichiometry="1" constant="true"/>

</listOfProducts>

</reaction>

<reaction metaid="H2S_cl_trans" id="H2S_cl_trans" name="H2S_cl_trans" reversible="true" fast="false" fbc:lowerFluxBound="FB1N1000" fbc:upperFluxBound="FB3N1000">

<listOfReactants>

<speciesReference species="M_H2S__91__d__93____91__c__93__" stoichiometry="1" constant="true"/>

</listOfReactants>

<listOfProducts>

<speciesReference species="M_H2S__91__d__93__" stoichiometry="1" constant="true"/>

</listOfProducts>

</reaction>

<reaction metaid="H2S_mit_trans" id="H2S_mit_trans" name="H2S_mit_trans" reversible="true" fast="false" fbc:lowerFluxBound="FB1N1000" fbc:upperFluxBound="FB3N1000">

<listOfReactants>

<speciesReference species="M_H2S__91__d__93____91__m__93__" stoichiometry="1" constant="true"/>

</listOfReactants>

<listOfProducts>

<speciesReference species="M_H2S__91__d__93__" stoichiometry="1" constant="true"/>

</listOfProducts>

</reaction>

<reaction metaid="ATP_mitexport" id="ATP_mitexport" name="ATP_mitexport" reversible="false" fast="false" fbc:lowerFluxBound="FB2N0" fbc:upperFluxBound="FB3N1000">

<listOfReactants>

<speciesReference species="M_ADP__91__d__93__" stoichiometry="1" constant="true"/>

<speciesReference species="M_ATP__91__d__93____91__m__93__" stoichiometry="1" constant="true"/>

</listOfReactants>

<listOfProducts>

<speciesReference species="M_ADP__91__d__93____91__m__93__" stoichiometry="1" constant="true"/>

<speciesReference species="M_ATP__91__d__93__" stoichiometry="1" constant="true"/>

</listOfProducts>

</reaction>

<reaction metaid="ATP_clexport" id="ATP_clexport" name="ATP_clexport" reversible="false" fast="false" fbc:lowerFluxBound="FB2N0" fbc:upperFluxBound="FB3N1000">

<listOfReactants>

<speciesReference species="M_ADP__91__d__93____91__c__93__" stoichiometry="1" constant="true"/>

<speciesReference species="M_ATP__91__d__93__" stoichiometry="1" constant="true"/>

<speciesReference species="M_Pi__91__d__93____91__c__93__" stoichiometry="1" constant="true"/>

</listOfReactants>

<listOfProducts>

<speciesReference species="M_ADP__91__d__93__" stoichiometry="1" constant="true"/>

<speciesReference species="M_ATP__91__d__93____91__c__93__" stoichiometry="1" constant="true"/>

<speciesReference species="M_Pi__91__d__93__" stoichiometry="1" constant="true"/>

</listOfProducts>

</reaction>

<reaction metaid="GluAsp_Antiporter_mit" id="GluAsp_Antiporter_mit" name="GluAsp_Antiporter_mit" reversible="false" fast="false" fbc:lowerFluxBound="FB2N0" fbc:upperFluxBound="FB3N1000">

<listOfReactants>

<speciesReference species="M_Aspartate__91__d__93____91__m__93__" stoichiometry="1" constant="true"/>

<speciesReference species="M_Glutamate__91__d__93__" stoichiometry="1" constant="true"/>

</listOfReactants>

<listOfProducts>

<speciesReference species="M_Aspartate__91__d__93__" stoichiometry="1" constant="true"/>

<speciesReference species="M_Glutamate__91__d__93____91__m__93__" stoichiometry="1" constant="true"/>

</listOfProducts>

</reaction>

<reaction metaid="DiT1" id="DiT1" name="DiT1" reversible="false" fast="false" fbc:lowerFluxBound="FB2N0" fbc:upperFluxBound="FB3N1000">

<listOfReactants>

<speciesReference species="M_Glutamate__91__d__93____91__c__93__" stoichiometry="1" constant="true"/>

<speciesReference species="M_Malate__91__d__93__" stoichiometry="1" constant="true"/>

</listOfReactants>

<listOfProducts>

<speciesReference species="M_Glutamate__91__d__93__" stoichiometry="1" constant="true"/>

<speciesReference species="M_Malate__91__d__93____91__c__93__" stoichiometry="1" constant="true"/>

</listOfProducts>

</reaction>

<reaction metaid="DiT2" id="DiT2" name="DiT2" reversible="false" fast="false" fbc:lowerFluxBound="FB2N0" fbc:upperFluxBound="FB3N1000">

<listOfReactants>

<speciesReference species="M_Malate__91__d__93____91__c__93__" stoichiometry="1" constant="true"/>

<speciesReference species="M_Oxoglutarate__91__d__93__" stoichiometry="1" constant="true"/>

</listOfReactants>

<listOfProducts>

<speciesReference species="M_Malate__91__d__93__" stoichiometry="1" constant="true"/>

<speciesReference species="M_Oxoglutarate__91__d__93____91__c__93__" stoichiometry="1" constant="true"/>

</listOfProducts>

</reaction>

<reaction metaid="MalOAA_cl" id="MalOAA_cl" name="MalOAA_cl" reversible="true" fast="false" fbc:lowerFluxBound="FB1N1000" fbc:upperFluxBound="FB3N1000">

<listOfReactants>

<speciesReference species="M_Malate__91__d__93__" stoichiometry="1" constant="true"/>

<speciesReference species="M_Oxaloacetate__91__d__93____91__c__93__" stoichiometry="1" constant="true"/>

</listOfReactants>

<listOfProducts>

<speciesReference species="M_Malate__91__d__93____91__c__93__" stoichiometry="1" constant="true"/>

<speciesReference species="M_Oxaloacetate__91__d__93__" stoichiometry="1" constant="true"/>

</listOfProducts>

</reaction>

<reaction metaid="MalOAA_per" id="MalOAA_per" name="MalOAA_per" reversible="true" fast="false" fbc:lowerFluxBound="FB1N1000" fbc:upperFluxBound="FB3N1000">

<listOfReactants>

<speciesReference species="M_Malate__91__d__93__" stoichiometry="1" constant="true"/>

<speciesReference species="M_Oxaloacetate__91__d__93____91__p__93__" stoichiometry="1" constant="true"/>

</listOfReactants>

<listOfProducts>

<speciesReference species="M_Malate__91__d__93____91__p__93__" stoichiometry="1" constant="true"/>

<speciesReference species="M_Oxaloacetate__91__d__93__" stoichiometry="1" constant="true"/>

</listOfProducts>

</reaction>

<reaction metaid="MalOAA_mit" id="MalOAA_mit" name="MalOAA_mit" reversible="true" fast="false" fbc:lowerFluxBound="FB1N1000" fbc:upperFluxBound="FB3N1000">

<listOfReactants>

<speciesReference species="M_Malate__91__d__93__" stoichiometry="1" constant="true"/>

<speciesReference species="M_Oxaloacetate__91__d__93____91__m__93__" stoichiometry="1" constant="true"/>

</listOfReactants>

<listOfProducts>

<speciesReference species="M_Malate__91__d__93____91__m__93__" stoichiometry="1" constant="true"/>

<speciesReference species="M_Oxaloacetate__91__d__93__" stoichiometry="1" constant="true"/>

</listOfProducts>

</reaction>

<reaction metaid="AAT_cyt" id="AAT_cyt" name="AAT_cyt" reversible="true" fast="false" fbc:lowerFluxBound="FB1N1000" fbc:upperFluxBound="FB3N1000">

<listOfReactants>

<speciesReference species="M_Glutamate__91__d__93____91__c__93__" stoichiometry="1" constant="true"/>

<speciesReference species="M_Oxaloacetate__91__d__93____91__c__93__" stoichiometry="1" constant="true"/>

</listOfReactants>

<listOfProducts>

<speciesReference species="M_Aspartate__91__d__93____91__c__93__" stoichiometry="1" constant="true"/>

<speciesReference species="M_Oxoglutarate__91__d__93____91__c__93__" stoichiometry="1" constant="true"/>

</listOfProducts>

</reaction>

<reaction metaid="AAT_mit" id="AAT_mit" name="AAT_mit" reversible="true" fast="false" fbc:lowerFluxBound="FB1N1000" fbc:upperFluxBound="FB3N1000">

<listOfReactants>

<speciesReference species="M_Glutamate__91__d__93____91__m__93__" stoichiometry="1" constant="true"/>

<speciesReference species="M_Oxaloacetate__91__d__93____91__m__93__" stoichiometry="1" constant="true"/>

</listOfReactants>

<listOfProducts>

<speciesReference species="M_Aspartate__91__d__93____91__m__93__" stoichiometry="1" constant="true"/>

<speciesReference species="M_Oxoglutarate__91__d__93____91__m__93__" stoichiometry="1" constant="true"/>

</listOfProducts>

</reaction>

<reaction metaid="AAT_per" id="AAT_per" name="AAT_per" reversible="true" fast="false" fbc:lowerFluxBound="FB1N1000" fbc:upperFluxBound="FB3N1000">

<listOfReactants>

<speciesReference species="M_Glutamate__91__d__93____91__p__93__" stoichiometry="1" constant="true"/>

</listOfReactants>

<listOfProducts>

<speciesReference species="M_Glutamate__91__d__93__" stoichiometry="1" constant="true"/>

</listOfProducts>

</reaction>

<reaction metaid="Oxo_mit_cyt" id="Oxo_mit_cyt" name="Oxo_mit_cyt" reversible="false" fast="false" fbc:lowerFluxBound="FB2N0" fbc:upperFluxBound="FB3N1000">

<listOfReactants>

<speciesReference species="M_Oxoglutarate__91__d__93____91__p__93__" stoichiometry="1" constant="true"/>

</listOfReactants>

<listOfProducts>

<speciesReference species="M_Oxoglutarate__91__d__93__" stoichiometry="1" constant="true"/>

</listOfProducts>

</reaction>

<reaction metaid="Glu_mit_cyt" id="Glu_mit_cyt" name="Glu_mit_cyt" reversible="true" fast="false" fbc:lowerFluxBound="FB1N1000" fbc:upperFluxBound="FB3N1000">

<listOfReactants>

<speciesReference species="M_Glutamate__91__d__93__" stoichiometry="1" constant="true"/>

</listOfReactants>

<listOfProducts>

<speciesReference species="M_Glutamate__91__d__93____91__p__93__" stoichiometry="1" constant="true"/>

</listOfProducts>

</reaction>

<reaction metaid="DTC_CitOxo" id="DTC_CitOxo" name="DTC_CitOxo" reversible="true" fast="false" fbc:lowerFluxBound="FB1N1000" fbc:upperFluxBound="FB3N1000">

<listOfReactants>

<speciesReference species="M_Citrate__91__d__93____91__m__93__" stoichiometry="1" constant="true"/>

<speciesReference species="M_Oxoglutarate__91__d__93__" stoichiometry="1" constant="true"/>

</listOfReactants>

<listOfProducts>

<speciesReference species="M_Citrate__91__d__93__" stoichiometry="1" constant="true"/>

<speciesReference species="M_Oxoglutarate__91__d__93____91__m__93__" stoichiometry="1" constant="true"/>

</listOfProducts>

</reaction>

<reaction metaid="DTC_CitIso" id="DTC_CitIso" name="DTC_CitIso" reversible="true" fast="false" fbc:lowerFluxBound="FB1N1000" fbc:upperFluxBound="FB3N1000">

<listOfReactants>

<speciesReference species="M_Citrate__91__d__93____91__m__93__" stoichiometry="1" constant="true"/>

<speciesReference species="M_Isocitrate__91__d__93__" stoichiometry="1" constant="true"/>

</listOfReactants>

<listOfProducts>

<speciesReference species="M_Citrate__91__d__93__" stoichiometry="1" constant="true"/>

<speciesReference species="M_Isocitrate__91__d__93____91__m__93__" stoichiometry="1" constant="true"/>

</listOfProducts>

</reaction>

<reaction metaid="DTC_CitCis" id="DTC_CitCis" name="DTC_CitCis" reversible="true" fast="false" fbc:lowerFluxBound="FB1N1000" fbc:upperFluxBound="FB3N1000">

<listOfReactants>

<speciesReference species="M_Cisaconitate__91__d__93__" stoichiometry="1" constant="true"/>

<speciesReference species="M_Citrate__91__d__93____91__m__93__" stoichiometry="1" constant="true"/>

</listOfReactants>

<listOfProducts>

<speciesReference species="M_Cisaconitate__91__d__93____91__m__93__" stoichiometry="1" constant="true"/>

<speciesReference species="M_Citrate__91__d__93__" stoichiometry="1" constant="true"/>

</listOfProducts>

</reaction>

<reaction metaid="DTC_IsoCis" id="DTC_IsoCis" name="DTC_IsoCis" reversible="true" fast="false" fbc:lowerFluxBound="FB1N1000" fbc:upperFluxBound="FB3N1000">

<listOfReactants>

<speciesReference species="M_Cisaconitate__91__d__93__" stoichiometry="1" constant="true"/>

<speciesReference species="M_Isocitrate__91__d__93____91__m__93__" stoichiometry="1" constant="true"/>

</listOfReactants>

<listOfProducts>

<speciesReference species="M_Cisaconitate__91__d__93____91__m__93__" stoichiometry="1" constant="true"/>

<speciesReference species="M_Isocitrate__91__d__93__" stoichiometry="1" constant="true"/>

</listOfProducts>

</reaction>

<reaction metaid="DTC_Oxo_Iso" id="DTC_Oxo_Iso" name="DTC_Oxo_Iso" reversible="true" fast="false" fbc:lowerFluxBound="FB1N1000" fbc:upperFluxBound="FB3N1000">

<listOfReactants>

<speciesReference species="M_Isocitrate__91__d__93__" stoichiometry="1" constant="true"/>

<speciesReference species="M_Oxoglutarate__91__d__93____91__m__93__" stoichiometry="1" constant="true"/>

</listOfReactants>

<listOfProducts>

<speciesReference species="M_Isocitrate__91__d__93____91__m__93__" stoichiometry="1" constant="true"/>

<speciesReference species="M_Oxoglutarate__91__d__93__" stoichiometry="1" constant="true"/>

</listOfProducts>

</reaction>

<reaction metaid="DTC_Oxo_Cisacon" id="DTC_Oxo_Cisacon" name="DTC_Oxo_Cisacon" reversible="true" fast="false" fbc:lowerFluxBound="FB1N1000" fbc:upperFluxBound="FB3N1000">

<listOfReactants>

<speciesReference species="M_Cisaconitate__91__d__93__" stoichiometry="1" constant="true"/>

<speciesReference species="M_Oxoglutarate__91__d__93____91__m__93__" stoichiometry="1" constant="true"/>

</listOfReactants>

<listOfProducts>

<speciesReference species="M_Cisaconitate__91__d__93____91__m__93__" stoichiometry="1" constant="true"/>

<speciesReference species="M_Oxoglutarate__91__d__93__" stoichiometry="1" constant="true"/>

</listOfProducts>

</reaction>

<reaction metaid="DTC_OAA_Succ" id="DTC_OAA_Succ" name="DTC_OAA_Succ" reversible="true" fast="false" fbc:lowerFluxBound="FB1N1000" fbc:upperFluxBound="FB3N1000">

<listOfReactants>

<speciesReference species="M_Oxaloacetate__91__d__93____91__m__93__" stoichiometry="1" constant="true"/>

<speciesReference species="M_Succinate__91__d__93__" stoichiometry="1" constant="true"/>

</listOfReactants>

<listOfProducts>

<speciesReference species="M_Oxaloacetate__91__d__93__" stoichiometry="1" constant="true"/>

<speciesReference species="M_Succinate__91__d__93____91__m__93__" stoichiometry="1" constant="true"/>

</listOfProducts>

</reaction>

<reaction metaid="DTC_OAA_Oxo" id="DTC_OAA_Oxo" name="DTC_OAA_Oxo" reversible="true" fast="false" fbc:lowerFluxBound="FB1N1000" fbc:upperFluxBound="FB3N1000">

<listOfReactants>

<speciesReference species="M_Oxaloacetate__91__d__93____91__m__93__" stoichiometry="1" constant="true"/>

<speciesReference species="M_Oxoglutarate__91__d__93__" stoichiometry="1" constant="true"/>

</listOfReactants>

<listOfProducts>

<speciesReference species="M_Oxaloacetate__91__d__93__" stoichiometry="1" constant="true"/>

<speciesReference species="M_Oxoglutarate__91__d__93____91__m__93__" stoichiometry="1" constant="true"/>

</listOfProducts>

</reaction>

<reaction metaid="DTC_OAA_Cit" id="DTC_OAA_Cit" name="DTC_OAA_Cit" reversible="true" fast="false" fbc:lowerFluxBound="FB1N1000" fbc:upperFluxBound="FB3N1000">

<listOfReactants>

<speciesReference species="M_Citrate__91__d__93__" stoichiometry="1" constant="true"/>

<speciesReference species="M_Oxaloacetate__91__d__93____91__m__93__" stoichiometry="1" constant="true"/>

</listOfReactants>

<listOfProducts>

<speciesReference species="M_Citrate__91__d__93____91__m__93__" stoichiometry="1" constant="true"/>

<speciesReference species="M_Oxaloacetate__91__d__93__" stoichiometry="1" constant="true"/>

</listOfProducts>

</reaction>

<reaction metaid="DTC_OAA_Isocit" id="DTC_OAA_Isocit" name="DTC_OAA_Isocit" reversible="true" fast="false" fbc:lowerFluxBound="FB1N1000" fbc:upperFluxBound="FB3N1000">

<listOfReactants>

<speciesReference species="M_Isocitrate__91__d__93__" stoichiometry="1" constant="true"/>

<speciesReference species="M_Oxaloacetate__91__d__93____91__m__93__" stoichiometry="1" constant="true"/>

</listOfReactants>

<listOfProducts>

<speciesReference species="M_Isocitrate__91__d__93____91__m__93__" stoichiometry="1" constant="true"/>

<speciesReference species="M_Oxaloacetate__91__d__93__" stoichiometry="1" constant="true"/>

</listOfProducts>

</reaction>

<reaction metaid="DTC_OAA_Cis" id="DTC_OAA_Cis" name="DTC_OAA_Cis" reversible="true" fast="false" fbc:lowerFluxBound="FB1N1000" fbc:upperFluxBound="FB3N1000">

<listOfReactants>

<speciesReference species="M_Cisaconitate__91__d__93__" stoichiometry="1" constant="true"/>

<speciesReference species="M_Oxaloacetate__91__d__93____91__m__93__" stoichiometry="1" constant="true"/>

</listOfReactants>

<listOfProducts>

<speciesReference species="M_Cisaconitate__91__d__93____91__m__93__" stoichiometry="1" constant="true"/>

<speciesReference species="M_Oxaloacetate__91__d__93__" stoichiometry="1" constant="true"/>

</listOfProducts>

</reaction>

<reaction metaid="DTC_MAL_CIS" id="DTC_MAL_CIS" name="DTC_MAL_CIS" reversible="true" fast="false" fbc:lowerFluxBound="FB1N1000" fbc:upperFluxBound="FB3N1000">

<listOfReactants>

<speciesReference species="M_Cisaconitate__91__d__93__" stoichiometry="1" constant="true"/>

<speciesReference species="M_Malate__91__d__93____91__m__93__" stoichiometry="1" constant="true"/>

</listOfReactants>

<listOfProducts>

<speciesReference species="M_Cisaconitate__91__d__93____91__m__93__" stoichiometry="1" constant="true"/>

<speciesReference species="M_Malate__91__d__93__" stoichiometry="1" constant="true"/>

</listOfProducts>

</reaction>

<reaction metaid="DTC_Mal_Suc" id="DTC_Mal_Suc" name="DTC_Mal_Suc" reversible="true" fast="false" fbc:lowerFluxBound="FB1N1000" fbc:upperFluxBound="FB3N1000">

<listOfReactants>

<speciesReference species="M_Malate__91__d__93____91__m__93__" stoichiometry="1" constant="true"/>

<speciesReference species="M_Succinate__91__d__93__" stoichiometry="1" constant="true"/>

</listOfReactants>

<listOfProducts>

<speciesReference species="M_Malate__91__d__93__" stoichiometry="1" constant="true"/>

<speciesReference species="M_Succinate__91__d__93____91__m__93__" stoichiometry="1" constant="true"/>

</listOfProducts>

</reaction>

<reaction metaid="DTC_Mal_Oxo" id="DTC_Mal_Oxo" name="DTC_Mal_Oxo" reversible="true" fast="false" fbc:lowerFluxBound="FB1N1000" fbc:upperFluxBound="FB3N1000">

<listOfReactants>

<speciesReference species="M_Malate__91__d__93____91__m__93__" stoichiometry="1" constant="true"/>

<speciesReference species="M_Oxoglutarate__91__d__93__" stoichiometry="1" constant="true"/>

</listOfReactants>

<listOfProducts>

<speciesReference species="M_Malate__91__d__93__" stoichiometry="1" constant="true"/>

<speciesReference species="M_Oxoglutarate__91__d__93____91__m__93__" stoichiometry="1" constant="true"/>

</listOfProducts>

</reaction>

<reaction metaid="DTC_Mal_Cit" id="DTC_Mal_Cit" name="DTC_Mal_Cit" reversible="true" fast="false" fbc:lowerFluxBound="FB1N1000" fbc:upperFluxBound="FB3N1000">

<listOfReactants>

<speciesReference species="M_Citrate__91__d__93__" stoichiometry="1" constant="true"/>

<speciesReference species="M_Malate__91__d__93____91__m__93__" stoichiometry="1" constant="true"/>

</listOfReactants>

<listOfProducts>

<speciesReference species="M_Citrate__91__d__93____91__m__93__" stoichiometry="1" constant="true"/>

<speciesReference species="M_Malate__91__d__93__" stoichiometry="1" constant="true"/>

</listOfProducts>

</reaction>

<reaction metaid="DTC_Mal_Isocit" id="DTC_Mal_Isocit" name="DTC_Mal_Isocit" reversible="true" fast="false" fbc:lowerFluxBound="FB1N1000" fbc:upperFluxBound="FB3N1000">

<listOfReactants>

<speciesReference species="M_Isocitrate__91__d__93__" stoichiometry="1" constant="true"/>

<speciesReference species="M_Malate__91__d__93____91__m__93__" stoichiometry="1" constant="true"/>

</listOfReactants>

<listOfProducts>

<speciesReference species="M_Isocitrate__91__d__93____91__m__93__" stoichiometry="1" constant="true"/>

<speciesReference species="M_Malate__91__d__93__" stoichiometry="1" constant="true"/>

</listOfProducts>

</reaction>

<reaction metaid="DTC_Suc_Oxo" id="DTC_Suc_Oxo" name="DTC_Suc_Oxo" reversible="true" fast="false" fbc:lowerFluxBound="FB1N1000" fbc:upperFluxBound="FB3N1000">

<listOfReactants>

<speciesReference species="M_Oxoglutarate__91__d__93__" stoichiometry="1" constant="true"/>

<speciesReference species="M_Succinate__91__d__93____91__m__93__" stoichiometry="1" constant="true"/>

</listOfReactants>

<listOfProducts>

<speciesReference species="M_Oxoglutarate__91__d__93____91__m__93__" stoichiometry="1" constant="true"/>

<speciesReference species="M_Succinate__91__d__93__" stoichiometry="1" constant="true"/>

</listOfProducts>

</reaction>

<reaction metaid="DTC_Suc_Cit" id="DTC_Suc_Cit" name="DTC_Suc_Cit" reversible="true" fast="false" fbc:lowerFluxBound="FB1N1000" fbc:upperFluxBound="FB3N1000">

<listOfReactants>

<speciesReference species="M_Citrate__91__d__93__" stoichiometry="1" constant="true"/>

<speciesReference species="M_Succinate__91__d__93____91__m__93__" stoichiometry="1" constant="true"/>

</listOfReactants>

<listOfProducts>

<speciesReference species="M_Citrate__91__d__93____91__m__93__" stoichiometry="1" constant="true"/>

<speciesReference species="M_Succinate__91__d__93__" stoichiometry="1" constant="true"/>

</listOfProducts>

</reaction>

<reaction metaid="DTC_Suc_Isocit" id="DTC_Suc_Isocit" name="DTC_Suc_Isocit" reversible="true" fast="false" fbc:lowerFluxBound="FB1N1000" fbc:upperFluxBound="FB3N1000">

<listOfReactants>

<speciesReference species="M_Isocitrate__91__d__93__" stoichiometry="1" constant="true"/>

<speciesReference species="M_Succinate__91__d__93____91__m__93__" stoichiometry="1" constant="true"/>

</listOfReactants>

<listOfProducts>

<speciesReference species="M_Isocitrate__91__d__93____91__m__93__" stoichiometry="1" constant="true"/>

<speciesReference species="M_Succinate__91__d__93__" stoichiometry="1" constant="true"/>

</listOfProducts>

</reaction>

<reaction metaid="DTC_Suc_Cisacont" id="DTC_Suc_Cisacont" name="DTC_Suc_Cisacont" reversible="true" fast="false" fbc:lowerFluxBound="FB1N1000" fbc:upperFluxBound="FB3N1000">

<listOfReactants>

<speciesReference species="M_Cisaconitate__91__d__93__" stoichiometry="1" constant="true"/>

<speciesReference species="M_Succinate__91__d__93____91__m__93__" stoichiometry="1" constant="true"/>

</listOfReactants>

<listOfProducts>

<speciesReference species="M_Cisaconitate__91__d__93____91__m__93__" stoichiometry="1" constant="true"/>

<speciesReference species="M_Succinate__91__d__93__" stoichiometry="1" constant="true"/>

</listOfProducts>

</reaction>

<reaction metaid="Succ_Fum_transport" id="Succ_Fum_transport" name="Succ_Fum_transport" reversible="true" fast="false" fbc:lowerFluxBound="FB1N1000" fbc:upperFluxBound="FB3N1000">

<listOfReactants>

<speciesReference species="M_Fumarate__91__d__93__" stoichiometry="1" constant="true"/>

<speciesReference species="M_Succinate__91__d__93____91__m__93__" stoichiometry="1" constant="true"/>

</listOfReactants>

<listOfProducts>

<speciesReference species="M_Fumarate__91__d__93____91__m__93__" stoichiometry="1" constant="true"/>

<speciesReference species="M_Succinate__91__d__93__" stoichiometry="1" constant="true"/>

</listOfProducts>

</reaction>

<reaction metaid="OAA_Pi_trans" id="OAA_Pi_trans" name="OAA_Pi_trans" reversible="true" fast="false" fbc:lowerFluxBound="FB1N1000" fbc:upperFluxBound="FB3N1000">

<listOfReactants>

<speciesReference species="M_Oxaloacetate__91__d__93____91__m__93__" stoichiometry="1" constant="true"/>

<speciesReference species="M_Pi__91__d__93__" stoichiometry="1" constant="true"/>

</listOfReactants>

<listOfProducts>

<speciesReference species="M_Oxaloacetate__91__d__93__" stoichiometry="1" constant="true"/>

<speciesReference species="M_Pi__91__d__93____91__m__93__" stoichiometry="1" constant="true"/>

</listOfProducts>

</reaction>

<reaction metaid="GlutamateGln_Trans" id="GlutamateGln_Trans" name="GlutamateGln_Trans" reversible="true" fast="false" fbc:lowerFluxBound="FB1N1000" fbc:upperFluxBound="FB3N1000">

<listOfReactants>

<speciesReference species="M_Glutamate__91__d__93__" stoichiometry="1" constant="true"/>

<speciesReference species="M_Glutamine__91__d__93____91__m__93__" stoichiometry="1" constant="true"/>

</listOfReactants>

<listOfProducts>

<speciesReference species="M_Glutamate__91__d__93____91__m__93__" stoichiometry="1" constant="true"/>

<speciesReference species="M_Glutamine__91__d__93__" stoichiometry="1" constant="true"/>

</listOfProducts>

</reaction>

<reaction metaid="Mal_Pi_trans" id="Mal_Pi_trans" name="Mal_Pi_trans" reversible="true" fast="false" fbc:lowerFluxBound="FB1N1000" fbc:upperFluxBound="FB3N1000">

<listOfReactants>

<speciesReference species="M_Malate__91__d__93____91__m__93__" stoichiometry="1" constant="true"/>

<speciesReference species="M_Pi__91__d__93__" stoichiometry="1" constant="true"/>

</listOfReactants>

<listOfProducts>

<speciesReference species="M_Malate__91__d__93__" stoichiometry="1" constant="true"/>

<speciesReference species="M_Pi__91__d__93____91__m__93__" stoichiometry="1" constant="true"/>

</listOfProducts>

</reaction>

<reaction metaid="Succ_Pi_transport" id="Succ_Pi_transport" name="Succ_Pi_transport" reversible="true" fast="false" fbc:lowerFluxBound="FB1N1000" fbc:upperFluxBound="FB3N1000">

<listOfReactants>

<speciesReference species="M_Pi__91__d__93__" stoichiometry="1" constant="true"/>

<speciesReference species="M_Succinate__91__d__93____91__m__93__" stoichiometry="1" constant="true"/>

</listOfReactants>

<listOfProducts>

<speciesReference species="M_Pi__91__d__93____91__m__93__" stoichiometry="1" constant="true"/>

<speciesReference species="M_Succinate__91__d__93__" stoichiometry="1" constant="true"/>

</listOfProducts>

</reaction>

<reaction metaid="ExportPEP" id="ExportPEP" name="ExportPEP" reversible="true" fast="false" fbc:lowerFluxBound="FB1N1000" fbc:upperFluxBound="FB3N1000">

<listOfReactants>

<speciesReference species="M_PEP__91__d__93____91__c__93__" stoichiometry="1" constant="true"/>

<speciesReference species="M_Pyruvate__91__d__93__" stoichiometry="1" constant="true"/>

</listOfReactants>

<listOfProducts>

<speciesReference species="M_PEP__91__d__93__" stoichiometry="1" constant="true"/>

<speciesReference species="M_Pyruvate__91__d__93____91__c__93__" stoichiometry="1" constant="true"/>

</listOfProducts>

</reaction>

<reaction metaid="ExportHexose" id="ExportHexose" name="ExportHexose" reversible="true" fast="false" fbc:lowerFluxBound="FB1N1000" fbc:upperFluxBound="FB3N1000">

<listOfReactants>

<speciesReference species="M_glucose__91__d__93____91__c__93__" stoichiometry="1" constant="true"/>

</listOfReactants>

<listOfProducts>

<speciesReference species="M_glucose__91__d__93__" stoichiometry="1" constant="true"/>

</listOfProducts>

</reaction>

<reaction metaid="Xu5P_tra" id="Xu5P_tra" name="Xu5P_tra" reversible="true" fast="false" fbc:lowerFluxBound="FB1N1000" fbc:upperFluxBound="FB3N1000">

<listOfReactants>

<speciesReference species="M_Pi__91__d__93__" stoichiometry="1" constant="true"/>

<speciesReference species="M_Xu5P__91__d__93____91__c__93__" stoichiometry="1" constant="true"/>

</listOfReactants>

<listOfProducts>

<speciesReference species="M_Pi__91__d__93____91__c__93__" stoichiometry="1" constant="true"/>

<speciesReference species="M_Xu5P__91__d__93__" stoichiometry="1" constant="true"/>

</listOfProducts>

</reaction>

<reaction metaid="Pyruvate_to_mit" id="Pyruvate_to_mit" name="Pyruvate_to_mit" reversible="false" fast="false" fbc:lowerFluxBound="FB2N0" fbc:upperFluxBound="FB3N1000">

<listOfReactants>

<speciesReference species="M_Pyruvate__91__d__93__" stoichiometry="1" constant="true"/>

</listOfReactants>

<listOfProducts>

<speciesReference species="M_Pyruvate__91__d__93____91__m__93__" stoichiometry="1" constant="true"/>

</listOfProducts>

</reaction>

<reaction metaid="Formate_Transp" id="Formate_Transp" name="Formate_Transp" reversible="true" fast="false" fbc:lowerFluxBound="FB1N1000" fbc:upperFluxBound="FB3N1000">

<listOfReactants>

<speciesReference species="M_Formate__91__d__93____91__m__93__" stoichiometry="1" constant="true"/>

</listOfReactants>

<listOfProducts>

<speciesReference species="M_Formate__91__d__93__" stoichiometry="1" constant="true"/>

</listOfProducts>

</reaction>

<reaction metaid="THF_Transp" id="THF_Transp" name="THF_Transp" reversible="true" fast="false" fbc:lowerFluxBound="FB1N1000" fbc:upperFluxBound="FB3N1000">

<listOfReactants>

<speciesReference species="M_THF__91__d__93__" stoichiometry="1" constant="true"/>

</listOfReactants>

<listOfProducts>

<speciesReference species="M_THF__91__d__93____91__m__93__" stoichiometry="1" constant="true"/>

</listOfProducts>

</reaction>

<reaction metaid="Glycolate_cl_per" id="Glycolate_cl_per" name="Glycolate_cl_per" reversible="false" fast="false" fbc:lowerFluxBound="FB2N0" fbc:upperFluxBound="FB3N1000">

<listOfReactants>

<speciesReference species="M_Glycolate__91__d__93____91__c__93__" stoichiometry="1" constant="true"/>

</listOfReactants>

<listOfProducts>

<speciesReference species="M_Glycolate__91__d__93____91__p__93__" stoichiometry="1" constant="true"/>

</listOfProducts>

</reaction>

<reaction metaid="Glycerate_per_cit" id="Glycerate_per_cit" name="Glycerate_per_cit" reversible="false" fast="false" fbc:lowerFluxBound="FB2N0" fbc:upperFluxBound="FB3N1000">

<listOfReactants>

<speciesReference species="M_Glycerate__91__d__93____91__p__93__" stoichiometry="1" constant="true"/>

</listOfReactants>

<listOfProducts>

<speciesReference species="M_Glycerate__91__d__93____91__c__93__" stoichiometry="1" constant="true"/>

</listOfProducts>

</reaction>

<reaction metaid="Serine_mit_per" id="Serine_mit_per" name="Serine_mit_per" reversible="false" fast="false" fbc:lowerFluxBound="FB2N0" fbc:upperFluxBound="FB3N1000">

<listOfReactants>

<speciesReference species="M_Serine__91__d__93____91__m__93__" stoichiometry="1" constant="true"/>

</listOfReactants>

<listOfProducts>

<speciesReference species="M_Serine__91__d__93____91__p__93__" stoichiometry="1" constant="true"/>

</listOfProducts>

</reaction>

<reaction metaid="Glycine_per_mit" id="Glycine_per_mit" name="Glycine_per_mit" reversible="true" fast="false" fbc:lowerFluxBound="FB1N1000" fbc:upperFluxBound="FB3N1000">

<listOfReactants>

<speciesReference species="M_Glycine__91__d__93____91__p__93__" stoichiometry="1" constant="true"/>

</listOfReactants>

<listOfProducts>

<speciesReference species="M_Glycine__91__d__93____91__m__93__" stoichiometry="1" constant="true"/>

</listOfProducts>

</reaction>

<reaction metaid="Cysteine_mit_cyt" id="Cysteine_mit_cyt" name="Cysteine_mit_cyt" reversible="true" fast="false" fbc:lowerFluxBound="FB1N1000" fbc:upperFluxBound="FB3N1000">

<listOfReactants>

<speciesReference species="M_Cysteine__91__d__93____91__m__93__" stoichiometry="1" constant="true"/>

</listOfReactants>

<listOfProducts>

<speciesReference species="M_Cysteine__91__d__93__" stoichiometry="1" constant="true"/>

</listOfProducts>

</reaction>

<reaction metaid="Nitrite_transport" id="Nitrite_transport" name="Nitrite_transport" reversible="true" fast="false" fbc:lowerFluxBound="FB1N1000" fbc:upperFluxBound="FB3N1000">

<listOfReactants>

<speciesReference species="M_NO2__91__d__93____91__c__93__" stoichiometry="1" constant="true"/>

</listOfReactants>

<listOfProducts>

<speciesReference species="M_NO2__91__d__93__" stoichiometry="1" constant="true"/>

</listOfProducts>

</reaction>

<reaction metaid="Nitrate_transport" id="Nitrate_transport" name="Nitrate_transport" reversible="true" fast="false" fbc:lowerFluxBound="FB1N1000" fbc:upperFluxBound="FB3N1000">

<listOfReactants>

<speciesReference species="M_NH3__91__d__93____91__m__93__" stoichiometry="1" constant="true"/>

</listOfReactants>

<listOfProducts>

<speciesReference species="M_NH3__91__d__93__" stoichiometry="1" constant="true"/>

</listOfProducts>

</reaction>

<reaction metaid="Pitrans" id="Pitrans" name="Pitrans" reversible="true" fast="false" fbc:lowerFluxBound="FB1N1000" fbc:upperFluxBound="FB3N1000">

<listOfReactants>

<speciesReference species="M_Pi__91__d__93__" stoichiometry="1" constant="true"/>

</listOfReactants>

<listOfProducts>

<speciesReference species="M_Pi__91__d__93____91__m__93__" stoichiometry="1" constant="true"/>

</listOfProducts>

</reaction>

<reaction metaid="G3P_trans" id="G3P_trans" name="G3P_trans" reversible="true" fast="false" fbc:lowerFluxBound="FB1N1000" fbc:upperFluxBound="FB3N1000">

<listOfReactants>

<speciesReference species="M_G3P__91__d__93____91__c__93__" stoichiometry="1" constant="true"/>

<speciesReference species="M_Pi__91__d__93__" stoichiometry="1" constant="true"/>

</listOfReactants>

<listOfProducts>

<speciesReference species="M_G3P__91__d__93__" stoichiometry="1" constant="true"/>

<speciesReference species="M_Pi__91__d__93____91__c__93__" stoichiometry="1" constant="true"/>

</listOfProducts>

</reaction>

<reaction metaid="DHAP_trans" id="DHAP_trans" name="DHAP_trans" reversible="false" fast="false" fbc:lowerFluxBound="FB2N0" fbc:upperFluxBound="FB3N1000">

<listOfReactants>

<speciesReference species="M_DHAP__91__d__93____91__c__93__" stoichiometry="1" constant="true"/>

<speciesReference species="M_Pi__91__d__93__" stoichiometry="1" constant="true"/>

</listOfReactants>

<listOfProducts>

<speciesReference species="M_DHAP__91__d__93__" stoichiometry="1" constant="true"/>

<speciesReference species="M_Pi__91__d__93____91__c__93__" stoichiometry="1" constant="true"/>

</listOfProducts>

</reaction>

<reaction metaid="PGA_trans" id="PGA_trans" name="PGA_trans" reversible="false" fast="false" fbc:lowerFluxBound="FB2N0" fbc:upperFluxBound="FB3N1000">

<listOfReactants>

<speciesReference species="M_3PGA__91__d__93__" stoichiometry="1" constant="true"/>

<speciesReference species="M_Pi__91__d__93____91__c__93__" stoichiometry="1" constant="true"/>

</listOfReactants>

<listOfProducts>

<speciesReference species="M_3PGA__91__d__93____91__c__93__" stoichiometry="1" constant="true"/>

<speciesReference species="M_Pi__91__d__93__" stoichiometry="1" constant="true"/>

</listOfProducts>

</reaction>

<reaction metaid="Ru5P_trans" id="Ru5P_trans" name="Ru5P_trans" reversible="false" fast="false" fbc:lowerFluxBound="FB2N0" fbc:upperFluxBound="FB3N1000">

<listOfReactants>

<speciesReference species="M_Pi__91__d__93____91__c__93__" stoichiometry="1" constant="true"/>

<speciesReference species="M_Ru5P__91__d__93__" stoichiometry="1" constant="true"/>

</listOfReactants>

<listOfProducts>

<speciesReference species="M_Pi__91__d__93__" stoichiometry="1" constant="true"/>

<speciesReference species="M_Ru5P__91__d__93____91__c__93__" stoichiometry="1" constant="true"/>

</listOfProducts>

</reaction>

<reaction metaid="E4P_trans" id="E4P_trans" name="E4P_trans" reversible="false" fast="false" fbc:lowerFluxBound="FB2N0" fbc:upperFluxBound="FB3N1000">

<listOfReactants>

<speciesReference species="M_E4P__91__d__93__" stoichiometry="1" constant="true"/>

<speciesReference species="M_Pi__91__d__93____91__c__93__" stoichiometry="1" constant="true"/>

</listOfReactants>

<listOfProducts>

<speciesReference species="M_E4P__91__d__93____91__c__93__" stoichiometry="1" constant="true"/>

<speciesReference species="M_Pi__91__d__93__" stoichiometry="1" constant="true"/>

</listOfProducts>

</reaction>

<reaction metaid="G6P_trans" id="G6P_trans" name="G6P_trans" reversible="false" fast="false" fbc:lowerFluxBound="FB2N0" fbc:upperFluxBound="FB3N1000">

<listOfReactants>

<speciesReference species="M_G6P__91__d__93__" stoichiometry="1" constant="true"/>

<speciesReference species="M_Pi__91__d__93____91__c__93__" stoichiometry="1" constant="true"/>

</listOfReactants>

<listOfProducts>

<speciesReference species="M_G6P__91__d__93____91__c__93__" stoichiometry="1" constant="true"/>

<speciesReference species="M_Pi__91__d__93__" stoichiometry="1" constant="true"/>

</listOfProducts>

</reaction>

<reaction metaid="LETC" id="LETC" name="LETC" reversible="false" fast="false" fbc:lowerFluxBound="FB2N0" fbc:upperFluxBound="FB3N1000">

<listOfReactants>

<speciesReference species="M_ADP__91__d__93____91__c__93__" stoichiometry="3" constant="true"/>

<speciesReference species="M_NADP__91__d__93____91__c__93__" stoichiometry="1" constant="true"/>

<speciesReference species="M_Pi__91__d__93____91__c__93__" stoichiometry="3" constant="true"/>

<speciesReference species="M_h700__91__c__93__" stoichiometry="7" constant="true"/>

</listOfReactants>

<listOfProducts>

<speciesReference species="M_ATP__91__d__93____91__c__93__" stoichiometry="3" constant="true"/>

<speciesReference species="M_H2O__91__d__93__" stoichiometry="2" constant="true"/>

<speciesReference species="M_NADPH__91__d__93____91__c__93__" stoichiometry="1" constant="true"/>

<speciesReference species="M_O2__91__d__93__" stoichiometry="0.5" constant="true"/>

</listOfProducts>

</reaction>

<reaction metaid="MP1" id="MP1" name="MP1" reversible="false" fast="false" fbc:lowerFluxBound="FB2N0" fbc:upperFluxBound="FB3N1000">

<listOfReactants>

<speciesReference species="M_ADP__91__d__93____91__m__93__" stoichiometry="2" constant="true"/>

<speciesReference species="M_FADH2__91__d__93____91__m__93__" stoichiometry="1" constant="true"/>

<speciesReference species="M_O2__91__d__93__" stoichiometry="0.5" constant="true"/>

<speciesReference species="M_Pi__91__d__93____91__m__93__" stoichiometry="2" constant="true"/>

</listOfReactants>

<listOfProducts>

<speciesReference species="M_ATP__91__d__93____91__m__93__" stoichiometry="2" constant="true"/>

<speciesReference species="M_FAD__91__d__93____91__m__93__" stoichiometry="1" constant="true"/>

<speciesReference species="M_H2O__91__d__93__" stoichiometry="3" constant="true"/>

</listOfProducts>

</reaction>

<reaction metaid="MP2" id="MP2" name="MP2" reversible="false" fast="false" fbc:lowerFluxBound="FB2N0" fbc:upperFluxBound="FB3N1000">

<listOfReactants>

<speciesReference species="M_ADP__91__d__93____91__m__93__" stoichiometry="3.33" constant="true"/>

<speciesReference species="M_NADH__91__d__93____91__m__93__" stoichiometry="1" constant="true"/>

<speciesReference species="M_O2__91__d__93__" stoichiometry="0.5" constant="true"/>

<speciesReference species="M_Pi__91__d__93____91__m__93__" stoichiometry="3.33" constant="true"/>

</listOfReactants>

<listOfProducts>

<speciesReference species="M_ATP__91__d__93____91__m__93__" stoichiometry="3.33" constant="true"/>

<speciesReference species="M_H2O__91__d__93__" stoichiometry="4.33" constant="true"/>

<speciesReference species="M_NAD__91__d__93____91__m__93__" stoichiometry="1" constant="true"/>

</listOfProducts>

</reaction>

<reaction metaid="NARyc" id="NARyc" name="NARyc" reversible="false" fast="false" fbc:lowerFluxBound="FB2N0" fbc:upperFluxBound="FB3N1000">

<listOfReactants>

<speciesReference species="M_HNO3__91__d__93__" stoichiometry="1" constant="true"/>

<speciesReference species="M_NADPH__91__d__93__" stoichiometry="1" constant="true"/>

</listOfReactants>

<listOfProducts>

<speciesReference species="M_NADP__91__d__93__" stoichiometry="1" constant="true"/>

<speciesReference species="M_NO2__91__d__93__" stoichiometry="1" constant="true"/>

</listOfProducts>

</reaction>

<reaction metaid="NiRy" id="NiRy" name="NiRy" reversible="false" fast="false" fbc:lowerFluxBound="FB2N0" fbc:upperFluxBound="FB3N1000">

<listOfReactants>

<speciesReference species="M_NADPH__91__d__93____91__c__93__" stoichiometry="3" constant="true"/>

<speciesReference species="M_NO2__91__d__93____91__c__93__" stoichiometry="1" constant="true"/>

</listOfReactants>

<listOfProducts>

<speciesReference species="M_NADP__91__d__93____91__c__93__" stoichiometry="3" constant="true"/>

<speciesReference species="M_NH3__91__d__93____91__c__93__" stoichiometry="1" constant="true"/>

</listOfProducts>

</reaction>

<reaction metaid="NARc" id="NARc" name="NARc" reversible="false" fast="false" fbc:lowerFluxBound="FB2N0" fbc:upperFluxBound="FB3N1000">

<listOfReactants>

<speciesReference species="M_HNO3__91__d__93__" stoichiometry="1" constant="true"/>

<speciesReference species="M_NADH__91__d__93__" stoichiometry="1" constant="true"/>

</listOfReactants>

<listOfProducts>

<speciesReference species="M_NAD__91__d__93__" stoichiometry="1" constant="true"/>

<speciesReference species="M_NO2__91__d__93__" stoichiometry="1" constant="true"/>

</listOfProducts>

</reaction>

<reaction metaid="NiR" id="NiR" name="NiR" reversible="false" fast="false" fbc:lowerFluxBound="FB2N0" fbc:upperFluxBound="FB3N1000">

<listOfReactants>

<speciesReference species="M_NADH__91__d__93____91__c__93__" stoichiometry="3" constant="true"/>

<speciesReference species="M_NO2__91__d__93____91__c__93__" stoichiometry="1" constant="true"/>

</listOfReactants>

<listOfProducts>

<speciesReference species="M_NAD__91__d__93____91__c__93__" stoichiometry="3" constant="true"/>

<speciesReference species="M_NH3__91__d__93____91__c__93__" stoichiometry="1" constant="true"/>

</listOfProducts>

</reaction>

<reaction metaid="RuBisCo" id="RuBisCo" name="RuBisCo" reversible="false" fast="false" fbc:lowerFluxBound="FB2N0" fbc:upperFluxBound="FB3N1000">

<listOfReactants>

<speciesReference species="M_CO2__91__d__93__" stoichiometry="1" constant="true"/>

<speciesReference species="M_H2O__91__d__93__" stoichiometry="1" constant="true"/>

<speciesReference species="M_RuBP__91__d__93____91__c__93__" stoichiometry="1" constant="true"/>

</listOfReactants>

<listOfProducts>

<speciesReference species="M_3PGA__91__d__93____91__c__93__" stoichiometry="2" constant="true"/>

</listOfProducts>

</reaction>

<reaction metaid="RuBisO" id="RuBisO" name="RuBisO" reversible="false" fast="false" fbc:lowerFluxBound="FB2N0" fbc:upperFluxBound="FB3N1000">

<listOfReactants>

<speciesReference species="M_H2O__91__d__93__" stoichiometry="1" constant="true"/>

<speciesReference species="M_O2__91__d__93__" stoichiometry="1" constant="true"/>

<speciesReference species="M_RuBP__91__d__93____91__c__93__" stoichiometry="1" constant="true"/>

</listOfReactants>

<listOfProducts>

<speciesReference species="M_3PGA__91__d__93____91__c__93__" stoichiometry="1" constant="true"/>

<speciesReference species="M_PGIA__91__d__93____91__c__93__" stoichiometry="1" constant="true"/>

</listOfProducts>

</reaction>

<reaction metaid="PGK" id="PGK" name="PGK" reversible="false" fast="false" fbc:lowerFluxBound="FB2N0" fbc:upperFluxBound="FB3N1000">

<listOfReactants>

<speciesReference species="M_3PGA__91__d__93____91__c__93__" stoichiometry="1" constant="true"/>

<speciesReference species="M_ATP__91__d__93____91__c__93__" stoichiometry="1" constant="true"/>

</listOfReactants>

<listOfProducts>

<speciesReference species="M_1_3BPGA__91__d__93____91__c__93__" stoichiometry="1" constant="true"/>

<speciesReference species="M_ADP__91__d__93____91__c__93__" stoichiometry="1" constant="true"/>

</listOfProducts>

</reaction>

<reaction metaid="GAPDHy" id="GAPDHy" name="GAPDHy" reversible="false" fast="false" fbc:lowerFluxBound="FB2N0" fbc:upperFluxBound="FB3N1000">

<listOfReactants>

<speciesReference species="M_1_3BPGA__91__d__93____91__c__93__" stoichiometry="1" constant="true"/>

<speciesReference species="M_NADPH__91__d__93____91__c__93__" stoichiometry="1" constant="true"/>

</listOfReactants>

<listOfProducts>

<speciesReference species="M_G3P__91__d__93____91__c__93__" stoichiometry="1" constant="true"/>

<speciesReference species="M_NADP__91__d__93____91__c__93__" stoichiometry="1" constant="true"/>

<speciesReference species="M_Pi__91__d__93____91__c__93__" stoichiometry="1" constant="true"/>

</listOfProducts>

</reaction>

<reaction metaid="GAPDH" id="GAPDH" name="GAPDH" reversible="true" fast="false" fbc:lowerFluxBound="FB1N1000" fbc:upperFluxBound="FB3N1000">

<listOfReactants>

<speciesReference species="M_1_3BPGA__91__d__93____91__c__93__" stoichiometry="1" constant="true"/>

<speciesReference species="M_NADH__91__d__93____91__c__93__" stoichiometry="1" constant="true"/>

</listOfReactants>

<listOfProducts>

<speciesReference species="M_G3P__91__d__93____91__c__93__" stoichiometry="1" constant="true"/>

<speciesReference species="M_NAD__91__d__93____91__c__93__" stoichiometry="1" constant="true"/>

<speciesReference species="M_Pi__91__d__93____91__c__93__" stoichiometry="1" constant="true"/>

</listOfProducts>

</reaction>

<reaction metaid="TPI" id="TPI" name="TPI" reversible="true" fast="false" fbc:lowerFluxBound="FB1N1000" fbc:upperFluxBound="FB3N1000">

<listOfReactants>

<speciesReference species="M_G3P__91__d__93____91__c__93__" stoichiometry="1" constant="true"/>

</listOfReactants>

<listOfProducts>

<speciesReference species="M_DHAP__91__d__93____91__c__93__" stoichiometry="1" constant="true"/>

</listOfProducts>

</reaction>

<reaction metaid="FBA" id="FBA" name="FBA" reversible="true" fast="false" fbc:lowerFluxBound="FB1N1000" fbc:upperFluxBound="FB3N1000">

<listOfReactants>

<speciesReference species="M_DHAP__91__d__93____91__c__93__" stoichiometry="1" constant="true"/>

<speciesReference species="M_G3P__91__d__93____91__c__93__" stoichiometry="1" constant="true"/>

</listOfReactants>

<listOfProducts>

<speciesReference species="M_FBP__91__d__93____91__c__93__" stoichiometry="1" constant="true"/>

</listOfProducts>

</reaction>

<reaction metaid="PFK1" id="PFK1" name="PFK1" reversible="false" fast="false" fbc:lowerFluxBound="FB2N0" fbc:upperFluxBound="FB3N1000">

<listOfReactants>

<speciesReference species="M_FBP__91__d__93____91__c__93__" stoichiometry="1" constant="true"/>

<speciesReference species="M_H2O__91__d__93__" stoichiometry="1" constant="true"/>

</listOfReactants>

<listOfProducts>

<speciesReference species="M_F6P__91__d__93____91__c__93__" stoichiometry="1" constant="true"/>

<speciesReference species="M_Pi__91__d__93____91__c__93__" stoichiometry="1" constant="true"/>

</listOfProducts>

</reaction>

<reaction metaid="TKT2" id="TKT2" name="TKT2" reversible="false" fast="false" fbc:lowerFluxBound="FB2N0" fbc:upperFluxBound="FB3N1000">

<listOfReactants>

<speciesReference species="M_F6P__91__d__93____91__c__93__" stoichiometry="1" constant="true"/>

<speciesReference species="M_G3P__91__d__93____91__c__93__" stoichiometry="1" constant="true"/>

</listOfReactants>

<listOfProducts>

<speciesReference species="M_E4P__91__d__93____91__c__93__" stoichiometry="1" constant="true"/>

<speciesReference species="M_Xu5P__91__d__93____91__c__93__" stoichiometry="1" constant="true"/>

</listOfProducts>

</reaction>

<reaction metaid="TKT2c" id="TKT2c" name="TKT2c" reversible="false" fast="false" fbc:lowerFluxBound="FB2N0" fbc:upperFluxBound="FB3N1000">

<listOfReactants>

<speciesReference species="M_E4P__91__d__93__" stoichiometry="1" constant="true"/>

<speciesReference species="M_Xu5P__91__d__93__" stoichiometry="1" constant="true"/>

</listOfReactants>

<listOfProducts>

<speciesReference species="M_F6P__91__d__93__" stoichiometry="1" constant="true"/>

<speciesReference species="M_G3P__91__d__93__" stoichiometry="1" constant="true"/>

</listOfProducts>

</reaction>

<reaction metaid="RPE" id="RPE" name="RPE" reversible="true" fast="false" fbc:lowerFluxBound="FB1N1000" fbc:upperFluxBound="FB3N1000">

<listOfReactants>

<speciesReference species="M_Ru5P__91__d__93____91__c__93__" stoichiometry="1" constant="true"/>

</listOfReactants>

<listOfProducts>

<speciesReference species="M_Xu5P__91__d__93____91__c__93__" stoichiometry="1" constant="true"/>

</listOfProducts>

</reaction>

<reaction metaid="RPEc" id="RPEc" name="RPEc" reversible="true" fast="false" fbc:lowerFluxBound="FB1N1000" fbc:upperFluxBound="FB3N1000">

<listOfReactants>

<speciesReference species="M_Ru5P__91__d__93__" stoichiometry="1" constant="true"/>

</listOfReactants>

<listOfProducts>

<speciesReference species="M_Xu5P__91__d__93__" stoichiometry="1" constant="true"/>

</listOfProducts>

</reaction>

<reaction metaid="TKT" id="TKT" name="TKT" reversible="false" fast="false" fbc:lowerFluxBound="FB2N0" fbc:upperFluxBound="FB3N1000">

<listOfReactants>

<speciesReference species="M_G3P__91__d__93____91__c__93__" stoichiometry="1" constant="true"/>

<speciesReference species="M_S7P__91__d__93____91__c__93__" stoichiometry="1" constant="true"/>

</listOfReactants>

<listOfProducts>

<speciesReference species="M_R5P__91__d__93____91__c__93__" stoichiometry="1" constant="true"/>

<speciesReference species="M_Xu5P__91__d__93____91__c__93__" stoichiometry="1" constant="true"/>

</listOfProducts>

</reaction>

<reaction metaid="TKTc" id="TKTc" name="TKTc" reversible="true" fast="false" fbc:lowerFluxBound="FB1N1000" fbc:upperFluxBound="FB3N1000">

<listOfReactants>

<speciesReference species="M_R5P__91__d__93__" stoichiometry="1" constant="true"/>

<speciesReference species="M_Xu5P__91__d__93__" stoichiometry="1" constant="true"/>

</listOfReactants>

<listOfProducts>

<speciesReference species="M_G3P__91__d__93__" stoichiometry="1" constant="true"/>

<speciesReference species="M_S7P__91__d__93__" stoichiometry="1" constant="true"/>

</listOfProducts>

</reaction>

<reaction metaid="Aldolase" id="Aldolase" name="Aldolase" reversible="true" fast="false" fbc:lowerFluxBound="FB1N1000" fbc:upperFluxBound="FB3N1000">

<listOfReactants>

<speciesReference species="M_DHAP__91__d__93____91__c__93__" stoichiometry="1" constant="true"/>

<speciesReference species="M_E4P__91__d__93____91__c__93__" stoichiometry="1" constant="true"/>

</listOfReactants>

<listOfProducts>

<speciesReference species="M_SBP__91__d__93____91__c__93__" stoichiometry="1" constant="true"/>

</listOfProducts>

</reaction>

<reaction metaid="Aldolasec" id="Aldolasec" name="Aldolasec" reversible="true" fast="false" fbc:lowerFluxBound="FB1N1000" fbc:upperFluxBound="FB3N1000">

<listOfReactants>

<speciesReference species="M_DHAP__91__d__93__" stoichiometry="1" constant="true"/>

<speciesReference species="M_E4P__91__d__93__" stoichiometry="1" constant="true"/>

</listOfReactants>

<listOfProducts>

<speciesReference species="M_SBP__91__d__93__" stoichiometry="1" constant="true"/>

</listOfProducts>

</reaction>

<reaction metaid="PFK" id="PFK" name="PFK" reversible="false" fast="false" fbc:lowerFluxBound="FB2N0" fbc:upperFluxBound="FB3N1000">

<listOfReactants>

<speciesReference species="M_H2O__91__d__93__" stoichiometry="1" constant="true"/>

<speciesReference species="M_SBP__91__d__93____91__c__93__" stoichiometry="1" constant="true"/>

</listOfReactants>

<listOfProducts>

<speciesReference species="M_Pi__91__d__93____91__c__93__" stoichiometry="1" constant="true"/>

<speciesReference species="M_S7P__91__d__93____91__c__93__" stoichiometry="1" constant="true"/>

</listOfProducts>

</reaction>

<reaction metaid="PFKc" id="PFKc" name="PFKc" reversible="false" fast="false" fbc:lowerFluxBound="FB2N0" fbc:upperFluxBound="FB3N1000">

<listOfReactants>

<speciesReference species="M_H2O__91__d__93__" stoichiometry="1" constant="true"/>

<speciesReference species="M_SBP__91__d__93__" stoichiometry="1" constant="true"/>

</listOfReactants>

<listOfProducts>

<speciesReference species="M_Pi__91__d__93__" stoichiometry="1" constant="true"/>

<speciesReference species="M_S7P__91__d__93__" stoichiometry="1" constant="true"/>

</listOfProducts>

</reaction>

<reaction metaid="RPI" id="RPI" name="RPI" reversible="true" fast="false" fbc:lowerFluxBound="FB1N1000" fbc:upperFluxBound="FB3N1000">

<listOfReactants>

<speciesReference species="M_R5P__91__d__93____91__c__93__" stoichiometry="1" constant="true"/>

</listOfReactants>

<listOfProducts>

<speciesReference species="M_Ru5P__91__d__93____91__c__93__" stoichiometry="1" constant="true"/>

</listOfProducts>

</reaction>

<reaction metaid="RPIc" id="RPIc" name="RPIc" reversible="true" fast="false" fbc:lowerFluxBound="FB1N1000" fbc:upperFluxBound="FB3N1000">

<listOfReactants>

<speciesReference species="M_R5P__91__d__93__" stoichiometry="1" constant="true"/>

</listOfReactants>

<listOfProducts>

<speciesReference species="M_Ru5P__91__d__93__" stoichiometry="1" constant="true"/>

</listOfProducts>

</reaction>

<reaction metaid="PRK" id="PRK" name="PRK" reversible="false" fast="false" fbc:lowerFluxBound="FB2N0" fbc:upperFluxBound="FB3N1000">

<listOfReactants>

<speciesReference species="M_ATP__91__d__93____91__c__93__" stoichiometry="1" constant="true"/>

<speciesReference species="M_Ru5P__91__d__93____91__c__93__" stoichiometry="1" constant="true"/>

</listOfReactants>

<listOfProducts>

<speciesReference species="M_ADP__91__d__93____91__c__93__" stoichiometry="1" constant="true"/>

<speciesReference species="M_RuBP__91__d__93____91__c__93__" stoichiometry="1" constant="true"/>

</listOfProducts>

</reaction>

<reaction metaid="PGI" id="PGI" name="PGI" reversible="true" fast="false" fbc:lowerFluxBound="FB1N1000" fbc:upperFluxBound="FB3N1000">

<listOfReactants>

<speciesReference species="M_F6P__91__d__93____91__c__93__" stoichiometry="1" constant="true"/>

</listOfReactants>

<listOfProducts>

<speciesReference species="M_G6P__91__d__93____91__c__93__" stoichiometry="1" constant="true"/>

</listOfProducts>

</reaction>

<reaction metaid="G6Pase" id="G6Pase" name="G6Pase" reversible="false" fast="false" fbc:lowerFluxBound="FB2N0" fbc:upperFluxBound="FB3N1000">

<listOfReactants>

<speciesReference species="M_G6P__91__d__93____91__c__93__" stoichiometry="1" constant="true"/>

<speciesReference species="M_H2O__91__d__93__" stoichiometry="1" constant="true"/>

</listOfReactants>

<listOfProducts>

<speciesReference species="M_Pi__91__d__93____91__c__93__" stoichiometry="1" constant="true"/>

<speciesReference species="M_glucose__91__d__93____91__c__93__" stoichiometry="1" constant="true"/>

</listOfProducts>

</reaction>

<reaction metaid="GltS" id="GltS" name="GltS" reversible="true" fast="false" fbc:lowerFluxBound="FB1N1000" fbc:upperFluxBound="FB3N1000">

<listOfReactants>

<speciesReference species="M_Glutamine__91__d__93____91__c__93__" stoichiometry="1" constant="true"/>

<speciesReference species="M_Oxoglutarate__91__d__93____91__c__93__" stoichiometry="1" constant="true"/>

</listOfReactants>

<listOfProducts>

<speciesReference species="M_Glutamate__91__d__93____91__c__93__" stoichiometry="2" constant="true"/>

</listOfProducts>

</reaction>

<reaction metaid="GS" id="GS" name="GS" reversible="true" fast="false" fbc:lowerFluxBound="FB1N1000" fbc:upperFluxBound="FB3N1000">

<listOfReactants>

<speciesReference species="M_ATP__91__d__93____91__c__93__" stoichiometry="1" constant="true"/>

<speciesReference species="M_Glutamate__91__d__93____91__c__93__" stoichiometry="1" constant="true"/>

<speciesReference species="M_NH3__91__d__93____91__c__93__" stoichiometry="1" constant="true"/>

</listOfReactants>

<listOfProducts>

<speciesReference species="M_ADP__91__d__93____91__c__93__" stoichiometry="1" constant="true"/>

<speciesReference species="M_Glutamine__91__d__93____91__c__93__" stoichiometry="1" constant="true"/>

<speciesReference species="M_Pi__91__d__93____91__c__93__" stoichiometry="1" constant="true"/>

</listOfProducts>

</reaction>

<reaction metaid="PGP" id="PGP" name="PGP" reversible="false" fast="false" fbc:lowerFluxBound="FB2N0" fbc:upperFluxBound="FB3N1000">

<listOfReactants>

<speciesReference species="M_H2O__91__d__93__" stoichiometry="1" constant="true"/>

<speciesReference species="M_PGIA__91__d__93____91__c__93__" stoichiometry="1" constant="true"/>

</listOfReactants>

<listOfProducts>

<speciesReference species="M_Glycolate__91__d__93____91__c__93__" stoichiometry="1" constant="true"/>

<speciesReference species="M_Pi__91__d__93____91__c__93__" stoichiometry="1" constant="true"/>

</listOfProducts>

</reaction>

<reaction metaid="GOXper" id="GOXper" name="GOXper" reversible="false" fast="false" fbc:lowerFluxBound="FB2N0" fbc:upperFluxBound="FB3N1000">

<listOfReactants>

<speciesReference species="M_Glycolate__91__d__93____91__p__93__" stoichiometry="1" constant="true"/>

<speciesReference species="M_O2__91__d__93__" stoichiometry="1" constant="true"/>

</listOfReactants>

<listOfProducts>

<speciesReference species="M_Glyoxylate__91__d__93____91__p__93__" stoichiometry="1" constant="true"/>

<speciesReference species="M_H2O2__91__d__93__" stoichiometry="1" constant="true"/>

</listOfProducts>

</reaction>

<reaction metaid="Catalasec" id="Catalasec" name="Catalasec" reversible="false" fast="false" fbc:lowerFluxBound="FB2N0" fbc:upperFluxBound="FB3N1000">

<listOfReactants>

<speciesReference species="M_H2O2__91__d__93__" stoichiometry="1" constant="true"/>

</listOfReactants>

<listOfProducts>

<speciesReference species="M_H2O__91__d__93__" stoichiometry="1" constant="true"/>

<speciesReference species="M_O2__91__d__93__" stoichiometry="0.5" constant="true"/>

</listOfProducts>

</reaction>

<reaction metaid="SGTper" id="SGTper" name="SGTper" reversible="false" fast="false" fbc:lowerFluxBound="FB2N0" fbc:upperFluxBound="FB3N1000">

<listOfReactants>

<speciesReference species="M_Glyoxylate__91__d__93____91__p__93__" stoichiometry="1" constant="true"/>

<speciesReference species="M_Serine__91__d__93____91__p__93__" stoichiometry="1" constant="true"/>

</listOfReactants>

<listOfProducts>

<speciesReference species="M_Glycine__91__d__93____91__p__93__" stoichiometry="1" constant="true"/>

<speciesReference species="M_hydroxypyruvate__91__d__93____91__p__93__" stoichiometry="1" constant="true"/>

</listOfProducts>

</reaction>

<reaction metaid="GTper" id="GTper" name="GTper" reversible="false" fast="false" fbc:lowerFluxBound="FB2N0" fbc:upperFluxBound="FB3N1000">

<listOfReactants>

<speciesReference species="M_Glutamate__91__d__93____91__p__93__" stoichiometry="1" constant="true"/>

<speciesReference species="M_Glyoxylate__91__d__93____91__p__93__" stoichiometry="1" constant="true"/>

</listOfReactants>

<listOfProducts>

<speciesReference species="M_Glycine__91__d__93____91__p__93__" stoichiometry="1" constant="true"/>

<speciesReference species="M_Oxoglutarate__91__d__93____91__p__93__" stoichiometry="1" constant="true"/>

</listOfProducts>

</reaction>

<reaction metaid="HPRper" id="HPRper" name="HPRper" reversible="false" fast="false" fbc:lowerFluxBound="FB2N0" fbc:upperFluxBound="FB3N1000">

<listOfReactants>

<speciesReference species="M_NADH__91__d__93____91__p__93__" stoichiometry="1" constant="true"/>

<speciesReference species="M_hydroxypyruvate__91__d__93____91__p__93__" stoichiometry="1" constant="true"/>

</listOfReactants>

<listOfProducts>

<speciesReference species="M_Glycerate__91__d__93____91__p__93__" stoichiometry="1" constant="true"/>

<speciesReference species="M_NAD__91__d__93____91__p__93__" stoichiometry="1" constant="true"/>

</listOfProducts>

</reaction>

<reaction metaid="GK" id="GK" name="GK" reversible="false" fast="false" fbc:lowerFluxBound="FB2N0" fbc:upperFluxBound="FB3N1000">

<listOfReactants>

<speciesReference species="M_ATP__91__d__93____91__c__93__" stoichiometry="1" constant="true"/>

<speciesReference species="M_Glycerate__91__d__93____91__c__93__" stoichiometry="1" constant="true"/>

</listOfReactants>

<listOfProducts>

<speciesReference species="M_3PGA__91__d__93____91__c__93__" stoichiometry="1" constant="true"/>

<speciesReference species="M_ADP__91__d__93____91__c__93__" stoichiometry="1" constant="true"/>

</listOfProducts>

</reaction>

<reaction metaid="TALAc" id="TALAc" name="TALAc" reversible="true" fast="false" fbc:lowerFluxBound="FB1N1000" fbc:upperFluxBound="FB3N1000">

<listOfReactants>

<speciesReference species="M_G3P__91__d__93__" stoichiometry="1" constant="true"/>

<speciesReference species="M_S7P__91__d__93__" stoichiometry="1" constant="true"/>

</listOfReactants>

<listOfProducts>

<speciesReference species="M_E4P__91__d__93__" stoichiometry="1" constant="true"/>

<speciesReference species="M_F6P__91__d__93__" stoichiometry="1" constant="true"/>

</listOfProducts>

</reaction>

<reaction metaid="OPPPc" id="OPPPc" name="OPPPc" reversible="false" fast="false" fbc:lowerFluxBound="FB2N0" fbc:upperFluxBound="FB3N1000">

<listOfReactants>

<speciesReference species="M_G6P__91__d__93__" stoichiometry="1" constant="true"/>

<speciesReference species="M_H2O__91__d__93__" stoichiometry="1" constant="true"/>

<speciesReference species="M_NADP__91__d__93__" stoichiometry="2" constant="true"/>

</listOfReactants>

<listOfProducts>

<speciesReference species="M_CO2__91__d__93__" stoichiometry="1" constant="true"/>

<speciesReference species="M_NADPH__91__d__93__" stoichiometry="2" constant="true"/>

<speciesReference species="M_Ru5P__91__d__93__" stoichiometry="1" constant="true"/>

</listOfProducts>

</reaction>

<reaction metaid="TALA" id="TALA" name="TALA" reversible="true" fast="false" fbc:lowerFluxBound="FB1N1000" fbc:upperFluxBound="FB3N1000">

<listOfReactants>

<speciesReference species="M_G3P__91__d__93____91__c__93__" stoichiometry="1" constant="true"/>

<speciesReference species="M_S7P__91__d__93____91__c__93__" stoichiometry="1" constant="true"/>

</listOfReactants>

<listOfProducts>

<speciesReference species="M_E4P__91__d__93____91__c__93__" stoichiometry="1" constant="true"/>

<speciesReference species="M_F6P__91__d__93____91__c__93__" stoichiometry="1" constant="true"/>

</listOfProducts>

</reaction>

<reaction metaid="OPPP" id="OPPP" name="OPPP" reversible="false" fast="false" fbc:lowerFluxBound="FB2N0" fbc:upperFluxBound="FB3N1000">

<listOfReactants>

<speciesReference species="M_G6P__91__d__93____91__c__93__" stoichiometry="1" constant="true"/>

<speciesReference species="M_H2O__91__d__93__" stoichiometry="1" constant="true"/>

<speciesReference species="M_NADP__91__d__93____91__c__93__" stoichiometry="2" constant="true"/>

</listOfReactants>

<listOfProducts>

<speciesReference species="M_CO2__91__d__93__" stoichiometry="1" constant="true"/>

<speciesReference species="M_NADPH__91__d__93____91__c__93__" stoichiometry="2" constant="true"/>

<speciesReference species="M_Ru5P__91__d__93____91__c__93__" stoichiometry="1" constant="true"/>

</listOfProducts>

</reaction>

<reaction metaid="HEX1c" id="HEX1c" name="HEX1c" reversible="false" fast="false" fbc:lowerFluxBound="FB2N0" fbc:upperFluxBound="FB3N1000">

<listOfReactants>

<speciesReference species="M_ATP__91__d__93__" stoichiometry="1" constant="true"/>

<speciesReference species="M_glucose__91__d__93__" stoichiometry="1" constant="true"/>

</listOfReactants>

<listOfProducts>

<speciesReference species="M_ADP__91__d__93__" stoichiometry="1" constant="true"/>

<speciesReference species="M_G6P__91__d__93__" stoichiometry="1" constant="true"/>

</listOfProducts>

</reaction>

<reaction metaid="PGIc" id="PGIc" name="PGIc" reversible="true" fast="false" fbc:lowerFluxBound="FB1N1000" fbc:upperFluxBound="FB3N1000">

<listOfReactants>

<speciesReference species="M_F6P__91__d__93__" stoichiometry="1" constant="true"/>

</listOfReactants>

<listOfProducts>

<speciesReference species="M_G6P__91__d__93__" stoichiometry="1" constant="true"/>

</listOfProducts>

</reaction>

<reaction metaid="PFKc1" id="PFKc1" name="PFKc1" reversible="false" fast="false" fbc:lowerFluxBound="FB2N0" fbc:upperFluxBound="FB3N1000">

<listOfReactants>

<speciesReference species="M_ATP__91__d__93__" stoichiometry="1" constant="true"/>

<speciesReference species="M_F6P__91__d__93__" stoichiometry="1" constant="true"/>

</listOfReactants>

<listOfProducts>

<speciesReference species="M_ADP__91__d__93__" stoichiometry="1" constant="true"/>

<speciesReference species="M_FBP__91__d__93__" stoichiometry="1" constant="true"/>

</listOfProducts>

</reaction>

<reaction metaid="FBAc" id="FBAc" name="FBAc" reversible="true" fast="false" fbc:lowerFluxBound="FB1N1000" fbc:upperFluxBound="FB3N1000">

<listOfReactants>

<speciesReference species="M_DHAP__91__d__93__" stoichiometry="1" constant="true"/>

<speciesReference species="M_G3P__91__d__93__" stoichiometry="1" constant="true"/>

</listOfReactants>

<listOfProducts>

<speciesReference species="M_FBP__91__d__93__" stoichiometry="1" constant="true"/>

</listOfProducts>

</reaction>

<reaction metaid="TPIc" id="TPIc" name="TPIc" reversible="true" fast="false" fbc:lowerFluxBound="FB1N1000" fbc:upperFluxBound="FB3N1000">

<listOfReactants>

<speciesReference species="M_G3P__91__d__93__" stoichiometry="1" constant="true"/>

</listOfReactants>

<listOfProducts>

<speciesReference species="M_DHAP__91__d__93__" stoichiometry="1" constant="true"/>

</listOfProducts>

</reaction>

<reaction metaid="GAPDHc" id="GAPDHc" name="GAPDHc" reversible="true" fast="false" fbc:lowerFluxBound="FB1N1000" fbc:upperFluxBound="FB3N1000">

<listOfReactants>

<speciesReference species="M_1_3BPGA__91__d__93__" stoichiometry="1" constant="true"/>

<speciesReference species="M_NADH__91__d__93__" stoichiometry="1" constant="true"/>

</listOfReactants>

<listOfProducts>

<speciesReference species="M_G3P__91__d__93__" stoichiometry="1" constant="true"/>

<speciesReference species="M_NAD__91__d__93__" stoichiometry="1" constant="true"/>

<speciesReference species="M_Pi__91__d__93__" stoichiometry="1" constant="true"/>

</listOfProducts>

</reaction>

<reaction metaid="NonP_GAPDHyc" id="NonP_GAPDHyc" name="NonP_GAPDHyc" reversible="false" fast="false" fbc:lowerFluxBound="FB2N0" fbc:upperFluxBound="FB3N1000">

<listOfReactants>

<speciesReference species="M_G3P__91__d__93__" stoichiometry="1" constant="true"/>

<speciesReference species="M_NADP__91__d__93__" stoichiometry="1" constant="true"/>

</listOfReactants>

<listOfProducts>

<speciesReference species="M_3PGA__91__d__93__" stoichiometry="1" constant="true"/>

<speciesReference species="M_NADPH__91__d__93__" stoichiometry="1" constant="true"/>

</listOfProducts>

</reaction>

<reaction metaid="PGK1" id="PGK1" name="PGK1" reversible="true" fast="false" fbc:lowerFluxBound="FB1N1000" fbc:upperFluxBound="FB3N1000">

<listOfReactants>

<speciesReference species="M_3PGA__91__d__93__" stoichiometry="1" constant="true"/>

<speciesReference species="M_ATP__91__d__93__" stoichiometry="1" constant="true"/>

</listOfReactants>

<listOfProducts>

<speciesReference species="M_1_3BPGA__91__d__93__" stoichiometry="1" constant="true"/>

<speciesReference species="M_ADP__91__d__93__" stoichiometry="1" constant="true"/>

</listOfProducts>

</reaction>

<reaction metaid="PGMc" id="PGMc" name="PGMc" reversible="true" fast="false" fbc:lowerFluxBound="FB1N1000" fbc:upperFluxBound="FB3N1000">

<listOfReactants>

<speciesReference species="M_3PGA__91__d__93__" stoichiometry="1" constant="true"/>

</listOfReactants>

<listOfProducts>

<speciesReference species="M_2PGA__91__d__93__" stoichiometry="1" constant="true"/>

</listOfProducts>

</reaction>

<reaction metaid="ENOc" id="ENOc" name="ENOc" reversible="true" fast="false" fbc:lowerFluxBound="FB1N1000" fbc:upperFluxBound="FB3N1000">

<listOfReactants>

<speciesReference species="M_2PGA__91__d__93__" stoichiometry="1" constant="true"/>

</listOfReactants>

<listOfProducts>

<speciesReference species="M_H2O__91__d__93__" stoichiometry="1" constant="true"/>

<speciesReference species="M_PEP__91__d__93__" stoichiometry="1" constant="true"/>

</listOfProducts>

</reaction>

<reaction metaid="PYKc" id="PYKc" name="PYKc" reversible="false" fast="false" fbc:lowerFluxBound="FB2N0" fbc:upperFluxBound="FB3N1000">

<listOfReactants>

<speciesReference species="M_ATP__91__d__93____91__c__93__" stoichiometry="1" constant="true"/>

<speciesReference species="M_Pyruvate__91__d__93____91__c__93__" stoichiometry="1" constant="true"/>

</listOfReactants>

<listOfProducts>

<speciesReference species="M_ADP__91__d__93____91__c__93__" stoichiometry="1" constant="true"/>

<speciesReference species="M_PEP__91__d__93____91__c__93__" stoichiometry="1" constant="true"/>

</listOfProducts>

</reaction>

<reaction metaid="EX1" id="EX1" name="EX1" reversible="false" fast="false" fbc:lowerFluxBound="FB2N0" fbc:upperFluxBound="FB3N1000">

<listOfReactants>

<speciesReference species="M_ATP__91__d__93____91__c__93__" stoichiometry="1" constant="true"/>

<speciesReference species="M_glucose__91__d__93____91__c__93__" stoichiometry="1" constant="true"/>

</listOfReactants>

<listOfProducts>

<speciesReference species="M_ADP__91__d__93____91__c__93__" stoichiometry="1" constant="true"/>

<speciesReference species="M_G6P__91__d__93____91__c__93__" stoichiometry="1" constant="true"/>

</listOfProducts>

</reaction>

<reaction metaid="PFK1c" id="PFK1c" name="PFK1c" reversible="false" fast="false" fbc:lowerFluxBound="FB2N0" fbc:upperFluxBound="FB3N1000">

<listOfReactants>

<speciesReference species="M_ATP__91__d__93____91__c__93__" stoichiometry="1" constant="true"/>

<speciesReference species="M_F6P__91__d__93____91__c__93__" stoichiometry="1" constant="true"/>

</listOfReactants>

<listOfProducts>

<speciesReference species="M_ADP__91__d__93____91__c__93__" stoichiometry="1" constant="true"/>

<speciesReference species="M_FBP__91__d__93____91__c__93__" stoichiometry="1" constant="true"/>

</listOfProducts>

</reaction>

<reaction metaid="PGM" id="PGM" name="PGM" reversible="true" fast="false" fbc:lowerFluxBound="FB1N1000" fbc:upperFluxBound="FB3N1000">

<listOfReactants>

<speciesReference species="M_3PGA__91__d__93____91__c__93__" stoichiometry="1" constant="true"/>

</listOfReactants>

<listOfProducts>

<speciesReference species="M_2PGA__91__d__93____91__c__93__" stoichiometry="1" constant="true"/>

</listOfProducts>

</reaction>

<reaction metaid="ENO" id="ENO" name="ENO" reversible="true" fast="false" fbc:lowerFluxBound="FB1N1000" fbc:upperFluxBound="FB3N1000">

<listOfReactants>

<speciesReference species="M_2PGA__91__d__93____91__c__93__" stoichiometry="1" constant="true"/>

</listOfReactants>

<listOfProducts>

<speciesReference species="M_H2O__91__d__93__" stoichiometry="1" constant="true"/>

<speciesReference species="M_PEP__91__d__93____91__c__93__" stoichiometry="1" constant="true"/>

</listOfProducts>

</reaction>

<reaction metaid="PYK" id="PYK" name="PYK" reversible="false" fast="false" fbc:lowerFluxBound="FB2N0" fbc:upperFluxBound="FB3N1000">

<listOfReactants>

<speciesReference species="M_ADP__91__d__93__" stoichiometry="1" constant="true"/>

<speciesReference species="M_PEP__91__d__93__" stoichiometry="1" constant="true"/>

</listOfReactants>

<listOfProducts>

<speciesReference species="M_ATP__91__d__93__" stoichiometry="1" constant="true"/>

<speciesReference species="M_Pyruvate__91__d__93__" stoichiometry="1" constant="true"/>

</listOfProducts>

</reaction>

<reaction metaid="PCm" id="PCm" name="PCm" reversible="false" fast="false" fbc:lowerFluxBound="FB2N0" fbc:upperFluxBound="FB3N1000">

<listOfReactants>

<speciesReference species="M_ATP__91__d__93____91__m__93__" stoichiometry="1" constant="true"/>

<speciesReference species="M_CO2__91__d__93__" stoichiometry="1" constant="true"/>

<speciesReference species="M_Pyruvate__91__d__93____91__m__93__" stoichiometry="1" constant="true"/>

</listOfReactants>

<listOfProducts>

<speciesReference species="M_ADP__91__d__93____91__m__93__" stoichiometry="1" constant="true"/>

<speciesReference species="M_Oxaloacetate__91__d__93____91__m__93__" stoichiometry="1" constant="true"/>

<speciesReference species="M_Pi__91__d__93____91__m__93__" stoichiometry="1" constant="true"/>

</listOfProducts>

</reaction>

<reaction metaid="PDHm" id="PDHm" name="PDHm" reversible="false" fast="false" fbc:lowerFluxBound="FB2N0" fbc:upperFluxBound="FB3N1000">

<listOfReactants>

<speciesReference species="M_CoenzymeA__91__d__93____91__m__93__" stoichiometry="1" constant="true"/>

<speciesReference species="M_NAD__91__d__93____91__m__93__" stoichiometry="1" constant="true"/>

<speciesReference species="M_Pyruvate__91__d__93____91__m__93__" stoichiometry="1" constant="true"/>

</listOfReactants>

<listOfProducts>

<speciesReference species="M_AcetylCoA__91__d__93____91__m__93__" stoichiometry="1" constant="true"/>

<speciesReference species="M_CO2__91__d__93__" stoichiometry="1" constant="true"/>

<speciesReference species="M_NADH__91__d__93____91__m__93__" stoichiometry="1" constant="true"/>

</listOfProducts>

</reaction>

<reaction metaid="PDH" id="PDH" name="PDH" reversible="false" fast="false" fbc:lowerFluxBound="FB2N0" fbc:upperFluxBound="FB3N1000">

<listOfReactants>

<speciesReference species="M_CoenzymeA__91__d__93____91__c__93__" stoichiometry="1" constant="true"/>

<speciesReference species="M_NAD__91__d__93____91__c__93__" stoichiometry="1" constant="true"/>

<speciesReference species="M_Pyruvate__91__d__93____91__c__93__" stoichiometry="1" constant="true"/>

</listOfReactants>

<listOfProducts>

<speciesReference species="M_AcetylCoA__91__d__93____91__c__93__" stoichiometry="1" constant="true"/>

<speciesReference species="M_CO2__91__d__93__" stoichiometry="1" constant="true"/>

<speciesReference species="M_NADH__91__d__93____91__c__93__" stoichiometry="1" constant="true"/>

</listOfProducts>

</reaction>

<reaction metaid="CSm" id="CSm" name="CSm" reversible="false" fast="false" fbc:lowerFluxBound="FB2N0" fbc:upperFluxBound="FB3N1000">

<listOfReactants>

<speciesReference species="M_AcetylCoA__91__d__93____91__m__93__" stoichiometry="1" constant="true"/>

<speciesReference species="M_H2O__91__d__93__" stoichiometry="1" constant="true"/>

<speciesReference species="M_Oxaloacetate__91__d__93____91__m__93__" stoichiometry="1" constant="true"/>

</listOfReactants>

<listOfProducts>

<speciesReference species="M_Citrate__91__d__93____91__m__93__" stoichiometry="1" constant="true"/>

<speciesReference species="M_CoenzymeA__91__d__93____91__m__93__" stoichiometry="1" constant="true"/>

</listOfProducts>

</reaction>

<reaction metaid="ACONTm" id="ACONTm" name="ACONTm" reversible="true" fast="false" fbc:lowerFluxBound="FB1N1000" fbc:upperFluxBound="FB3N1000">

<listOfReactants>

<speciesReference species="M_Citrate__91__d__93____91__m__93__" stoichiometry="1" constant="true"/>

</listOfReactants>

<listOfProducts>

<speciesReference species="M_Isocitrate__91__d__93____91__m__93__" stoichiometry="1" constant="true"/>

</listOfProducts>

</reaction>

<reaction metaid="ICDHym" id="ICDHym" name="ICDHym" reversible="true" fast="false" fbc:lowerFluxBound="FB1N1000" fbc:upperFluxBound="FB3N1000">

<listOfReactants>

<speciesReference species="M_Isocitrate__91__d__93____91__m__93__" stoichiometry="1" constant="true"/>

<speciesReference species="M_NADP__91__d__93____91__m__93__" stoichiometry="1" constant="true"/>

</listOfReactants>

<listOfProducts>

<speciesReference species="M_CO2__91__d__93__" stoichiometry="1" constant="true"/>

<speciesReference species="M_NADPH__91__d__93____91__m__93__" stoichiometry="1" constant="true"/>

<speciesReference species="M_Oxoglutarate__91__d__93____91__m__93__" stoichiometry="1" constant="true"/>

</listOfProducts>

</reaction>

<reaction metaid="ACONTc" id="ACONTc" name="ACONTc" reversible="true" fast="false" fbc:lowerFluxBound="FB1N1000" fbc:upperFluxBound="FB3N1000">

<listOfReactants>

<speciesReference species="M_Citrate__91__d__93__" stoichiometry="1" constant="true"/>

</listOfReactants>

<listOfProducts>

<speciesReference species="M_Isocitrate__91__d__93__" stoichiometry="1" constant="true"/>

</listOfProducts>

</reaction>

<reaction metaid="ICDHyc" id="ICDHyc" name="ICDHyc" reversible="true" fast="false" fbc:lowerFluxBound="FB1N1000" fbc:upperFluxBound="FB3N1000">

<listOfReactants>

<speciesReference species="M_Isocitrate__91__d__93__" stoichiometry="1" constant="true"/>

<speciesReference species="M_NADP__91__d__93__" stoichiometry="1" constant="true"/>

</listOfReactants>

<listOfProducts>

<speciesReference species="M_CO2__91__d__93__" stoichiometry="1" constant="true"/>

<speciesReference species="M_NADPH__91__d__93__" stoichiometry="1" constant="true"/>

<speciesReference species="M_Oxoglutarate__91__d__93__" stoichiometry="1" constant="true"/>

</listOfProducts>

</reaction>

<reaction metaid="ICDHc" id="ICDHc" name="ICDHc" reversible="true" fast="false" fbc:lowerFluxBound="FB1N1000" fbc:upperFluxBound="FB3N1000">

<listOfReactants>

<speciesReference species="M_Isocitrate__91__d__93__" stoichiometry="1" constant="true"/>

<speciesReference species="M_NAD__91__d__93__" stoichiometry="1" constant="true"/>

</listOfReactants>

<listOfProducts>

<speciesReference species="M_CO2__91__d__93__" stoichiometry="1" constant="true"/>

<speciesReference species="M_NADH__91__d__93__" stoichiometry="1" constant="true"/>

<speciesReference species="M_Oxoglutarate__91__d__93__" stoichiometry="1" constant="true"/>

</listOfProducts>

</reaction>

<reaction metaid="AKGDm" id="AKGDm" name="AKGDm" reversible="true" fast="false" fbc:lowerFluxBound="FB1N1000" fbc:upperFluxBound="FB3N1000">

<listOfReactants>

<speciesReference species="M_CoenzymeA__91__d__93____91__m__93__" stoichiometry="1" constant="true"/>

<speciesReference species="M_NAD__91__d__93____91__m__93__" stoichiometry="1" constant="true"/>

<speciesReference species="M_Oxoglutarate__91__d__93____91__m__93__" stoichiometry="1" constant="true"/>

</listOfReactants>

<listOfProducts>

<speciesReference species="M_CO2__91__d__93__" stoichiometry="1" constant="true"/>

<speciesReference species="M_NADH__91__d__93____91__m__93__" stoichiometry="1" constant="true"/>

<speciesReference species="M_SuccinylCoA__91__d__93____91__m__93__" stoichiometry="1" constant="true"/>

</listOfProducts>

</reaction>

<reaction metaid="SUCOASm" id="SUCOASm" name="SUCOASm" reversible="true" fast="false" fbc:lowerFluxBound="FB1N1000" fbc:upperFluxBound="FB3N1000">

<listOfReactants>

<speciesReference species="M_ADP__91__d__93____91__m__93__" stoichiometry="1" constant="true"/>

<speciesReference species="M_Pi__91__d__93____91__m__93__" stoichiometry="1" constant="true"/>

<speciesReference species="M_SuccinylCoA__91__d__93____91__m__93__" stoichiometry="1" constant="true"/>

</listOfReactants>

<listOfProducts>

<speciesReference species="M_ATP__91__d__93____91__m__93__" stoichiometry="1" constant="true"/>

<speciesReference species="M_CoenzymeA__91__d__93____91__m__93__" stoichiometry="1" constant="true"/>

<speciesReference species="M_Succinate__91__d__93____91__m__93__" stoichiometry="1" constant="true"/>

</listOfProducts>

</reaction>

<reaction metaid="FUMm" id="FUMm" name="FUMm" reversible="true" fast="false" fbc:lowerFluxBound="FB1N1000" fbc:upperFluxBound="FB3N1000">

<listOfReactants>

<speciesReference species="M_Fumarate__91__d__93____91__m__93__" stoichiometry="1" constant="true"/>

<speciesReference species="M_H2O__91__d__93__" stoichiometry="1" constant="true"/>

</listOfReactants>

<listOfProducts>

<speciesReference species="M_Malate__91__d__93____91__m__93__" stoichiometry="1" constant="true"/>

</listOfProducts>

</reaction>

<reaction metaid="FUMc" id="FUMc" name="FUMc" reversible="true" fast="false" fbc:lowerFluxBound="FB1N1000" fbc:upperFluxBound="FB3N1000">

<listOfReactants>

<speciesReference species="M_Fumarate__91__d__93__" stoichiometry="1" constant="true"/>

<speciesReference species="M_H2O__91__d__93__" stoichiometry="1" constant="true"/>

</listOfReactants>

<listOfProducts>

<speciesReference species="M_Malate__91__d__93__" stoichiometry="1" constant="true"/>

</listOfProducts>

</reaction>

<reaction metaid="MDHm" id="MDHm" name="MDHm" reversible="true" fast="false" fbc:lowerFluxBound="FB1N1000" fbc:upperFluxBound="FB3N1000">

<listOfReactants>

<speciesReference species="M_Malate__91__d__93____91__m__93__" stoichiometry="1" constant="true"/>

<speciesReference species="M_NAD__91__d__93____91__m__93__" stoichiometry="1" constant="true"/>

</listOfReactants>

<listOfProducts>

<speciesReference species="M_NADH__91__d__93____91__m__93__" stoichiometry="1" constant="true"/>

<speciesReference species="M_Oxaloacetate__91__d__93____91__m__93__" stoichiometry="1" constant="true"/>

</listOfProducts>

</reaction>

<reaction metaid="MDHc" id="MDHc" name="MDHc" reversible="true" fast="false" fbc:lowerFluxBound="FB1N1000" fbc:upperFluxBound="FB3N1000">

<listOfReactants>

<speciesReference species="M_Malate__91__d__93__" stoichiometry="1" constant="true"/>

<speciesReference species="M_NAD__91__d__93__" stoichiometry="1" constant="true"/>

</listOfReactants>

<listOfProducts>

<speciesReference species="M_NADH__91__d__93__" stoichiometry="1" constant="true"/>

<speciesReference species="M_Oxaloacetate__91__d__93__" stoichiometry="1" constant="true"/>

</listOfProducts>

</reaction>

<reaction metaid="MDHper" id="MDHper" name="MDHper" reversible="true" fast="false" fbc:lowerFluxBound="FB1N1000" fbc:upperFluxBound="FB3N1000">

<listOfReactants>

<speciesReference species="M_Malate__91__d__93____91__p__93__" stoichiometry="1" constant="true"/>

<speciesReference species="M_NAD__91__d__93____91__p__93__" stoichiometry="1" constant="true"/>

</listOfReactants>

<listOfProducts>

<speciesReference species="M_NADH__91__d__93____91__p__93__" stoichiometry="1" constant="true"/>

<speciesReference species="M_Oxaloacetate__91__d__93____91__p__93__" stoichiometry="1" constant="true"/>

</listOfProducts>

</reaction>

<reaction metaid="MDH" id="MDH" name="MDH" reversible="true" fast="false" fbc:lowerFluxBound="FB1N1000" fbc:upperFluxBound="FB3N1000">

<listOfReactants>

<speciesReference species="M_NADH__91__d__93____91__c__93__" stoichiometry="1" constant="true"/>

<speciesReference species="M_Oxaloacetate__91__d__93____91__c__93__" stoichiometry="1" constant="true"/>

</listOfReactants>

<listOfProducts>

<speciesReference species="M_Malate__91__d__93____91__c__93__" stoichiometry="1" constant="true"/>

<speciesReference species="M_NAD__91__d__93____91__c__93__" stoichiometry="1" constant="true"/>

</listOfProducts>

</reaction>

<reaction metaid="MDHy" id="MDHy" name="MDHy" reversible="false" fast="false" fbc:lowerFluxBound="FB2N0" fbc:upperFluxBound="FB3N1000">

<listOfReactants>

<speciesReference species="M_NADPH__91__d__93____91__c__93__" stoichiometry="1" constant="true"/>

<speciesReference species="M_Oxaloacetate__91__d__93____91__c__93__" stoichiometry="1" constant="true"/>

</listOfReactants>

<listOfProducts>

<speciesReference species="M_Malate__91__d__93____91__c__93__" stoichiometry="1" constant="true"/>

<speciesReference species="M_NADP__91__d__93____91__c__93__" stoichiometry="1" constant="true"/>

</listOfProducts>

</reaction>

<reaction metaid="SUCD1m" id="SUCD1m" name="SUCD1m" reversible="false" fast="false" fbc:lowerFluxBound="FB2N0" fbc:upperFluxBound="FB3N1000">

<listOfReactants>

<speciesReference species="M_FAD__91__d__93____91__m__93__" stoichiometry="1" constant="true"/>

<speciesReference species="M_Succinate__91__d__93____91__m__93__" stoichiometry="1" constant="true"/>

</listOfReactants>

<listOfProducts>

<speciesReference species="M_FADH2__91__d__93____91__m__93__" stoichiometry="1" constant="true"/>

<speciesReference species="M_Fumarate__91__d__93____91__m__93__" stoichiometry="1" constant="true"/>

</listOfProducts>

</reaction>

<reaction metaid="GPDHm" id="GPDHm" name="GPDHm" reversible="false" fast="false" fbc:lowerFluxBound="FB2N0" fbc:upperFluxBound="FB3N1000">

<listOfReactants>

<speciesReference species="M_FAD__91__d__93____91__m__93__" stoichiometry="1" constant="true"/>

<speciesReference species="M_Glycerol3P__91__d__93____91__m__93__" stoichiometry="1" constant="true"/>

</listOfReactants>

<listOfProducts>

<speciesReference species="M_DHAP__91__d__93____91__m__93__" stoichiometry="1" constant="true"/>

<speciesReference species="M_FADH2__91__d__93____91__m__93__" stoichiometry="1" constant="true"/>

</listOfProducts>

</reaction>

<reaction metaid="GPDHc" id="GPDHc" name="GPDHc" reversible="false" fast="false" fbc:lowerFluxBound="FB2N0" fbc:upperFluxBound="FB3N1000">

<listOfReactants>

<speciesReference species="M_DHAP__91__d__93__" stoichiometry="1" constant="true"/>

<speciesReference species="M_NADH__91__d__93__" stoichiometry="1" constant="true"/>

</listOfReactants>

<listOfProducts>

<speciesReference species="M_Glycerol3P__91__d__93__" stoichiometry="1" constant="true"/>

<speciesReference species="M_NAD__91__d__93__" stoichiometry="1" constant="true"/>

</listOfProducts>

</reaction>

<reaction metaid="GDHm" id="GDHm" name="GDHm" reversible="true" fast="false" fbc:lowerFluxBound="FB1N1000" fbc:upperFluxBound="FB3N1000">

<listOfReactants>

<speciesReference species="M_Glutamate__91__d__93____91__m__93__" stoichiometry="1" constant="true"/>

<speciesReference species="M_H2O__91__d__93__" stoichiometry="1" constant="true"/>

<speciesReference species="M_NAD__91__d__93____91__m__93__" stoichiometry="1" constant="true"/>

</listOfReactants>

<listOfProducts>

<speciesReference species="M_NADH__91__d__93____91__m__93__" stoichiometry="1" constant="true"/>

<speciesReference species="M_NH3__91__d__93____91__m__93__" stoichiometry="1" constant="true"/>

<speciesReference species="M_Oxoglutarate__91__d__93____91__m__93__" stoichiometry="1" constant="true"/>

</listOfProducts>

</reaction>

<reaction metaid="Glutaminasem" id="Glutaminasem" name="Glutaminasem" reversible="false" fast="false" fbc:lowerFluxBound="FB2N0" fbc:upperFluxBound="FB3N1000">

<listOfReactants>

<speciesReference species="M_Glutamine__91__d__93____91__m__93__" stoichiometry="1" constant="true"/>

<speciesReference species="M_H2O__91__d__93__" stoichiometry="1" constant="true"/>

</listOfReactants>

<listOfProducts>

<speciesReference species="M_Glutamate__91__d__93____91__m__93__" stoichiometry="1" constant="true"/>

<speciesReference species="M_NH3__91__d__93____91__m__93__" stoichiometry="1" constant="true"/>

</listOfProducts>

</reaction>

<reaction metaid="EB1c" id="EB1c" name="EB1c" reversible="true" fast="false" fbc:lowerFluxBound="FB1N1000" fbc:upperFluxBound="FB3N1000">

<listOfReactants>

<speciesReference species="M_H2O__91__d__93__" stoichiometry="1" constant="true"/>

<speciesReference species="M_PPi__91__d__93__" stoichiometry="1" constant="true"/>

</listOfReactants>

<listOfProducts>

<speciesReference species="M_Pi__91__d__93__" stoichiometry="2" constant="true"/>

</listOfProducts>

</reaction>

<reaction metaid="EB1" id="EB1" name="EB1" reversible="true" fast="false" fbc:lowerFluxBound="FB1N1000" fbc:upperFluxBound="FB3N1000">

<listOfReactants>

<speciesReference species="M_H2O__91__d__93__" stoichiometry="1" constant="true"/>

<speciesReference species="M_PPi__91__d__93____91__c__93__" stoichiometry="1" constant="true"/>

</listOfReactants>

<listOfProducts>

<speciesReference species="M_Pi__91__d__93____91__c__93__" stoichiometry="2" constant="true"/>

</listOfProducts>

</reaction>

<reaction metaid="EB1m" id="EB1m" name="EB1m" reversible="true" fast="false" fbc:lowerFluxBound="FB1N1000" fbc:upperFluxBound="FB3N1000">

<listOfReactants>

<speciesReference species="M_H2O__91__d__93__" stoichiometry="1" constant="true"/>

<speciesReference species="M_PPi__91__d__93____91__m__93__" stoichiometry="1" constant="true"/>

</listOfReactants>

<listOfProducts>

<speciesReference species="M_Pi__91__d__93____91__m__93__" stoichiometry="2" constant="true"/>

</listOfProducts>

</reaction>

<reaction metaid="EB2c" id="EB2c" name="EB2c" reversible="true" fast="false" fbc:lowerFluxBound="FB1N1000" fbc:upperFluxBound="FB3N1000">

<listOfReactants>

<speciesReference species="M_AMP__91__d__93__" stoichiometry="1" constant="true"/>

<speciesReference species="M_ATP__91__d__93__" stoichiometry="1" constant="true"/>

</listOfReactants>

<listOfProducts>

<speciesReference species="M_ADP__91__d__93__" stoichiometry="2" constant="true"/>

</listOfProducts>

</reaction>

<reaction metaid="EB2" id="EB2" name="EB2" reversible="true" fast="false" fbc:lowerFluxBound="FB1N1000" fbc:upperFluxBound="FB3N1000">

<listOfReactants>

<speciesReference species="M_AMP__91__d__93____91__c__93__" stoichiometry="1" constant="true"/>

<speciesReference species="M_ATP__91__d__93____91__c__93__" stoichiometry="1" constant="true"/>

</listOfReactants>

<listOfProducts>

<speciesReference species="M_ADP__91__d__93____91__c__93__" stoichiometry="2" constant="true"/>

</listOfProducts>

</reaction>

<reaction metaid="EB2m" id="EB2m" name="EB2m" reversible="true" fast="false" fbc:lowerFluxBound="FB1N1000" fbc:upperFluxBound="FB3N1000">

<listOfReactants>

<speciesReference species="M_AMP__91__d__93____91__m__93__" stoichiometry="1" constant="true"/>

<speciesReference species="M_ATP__91__d__93____91__m__93__" stoichiometry="1" constant="true"/>

</listOfReactants>

<listOfProducts>

<speciesReference species="M_ADP__91__d__93____91__m__93__" stoichiometry="2" constant="true"/>

</listOfProducts>

</reaction>

<reaction metaid="CR1c" id="CR1c" name="CR1c" reversible="false" fast="false" fbc:lowerFluxBound="FB2N0" fbc:upperFluxBound="FB3N1000">

<listOfReactants>

<speciesReference species="M_NADPH__91__d__93____91__c__93__" stoichiometry="1" constant="true"/>

<speciesReference species="M_Pyruvate__91__d__93____91__c__93__" stoichiometry="2" constant="true"/>

</listOfReactants>

<listOfProducts>

<speciesReference species="M_1_2Oxoisovalerate__91__d__93____91__c__93__" stoichiometry="1" constant="true"/>

<speciesReference species="M_CO2__91__d__93__" stoichiometry="1" constant="true"/>

<speciesReference species="M_H2O__91__d__93__" stoichiometry="1" constant="true"/>

<speciesReference species="M_NADP__91__d__93____91__c__93__" stoichiometry="1" constant="true"/>

</listOfProducts>

</reaction>

<reaction metaid="PEPCx" id="PEPCx" name="PEPCx" reversible="false" fast="false" fbc:lowerFluxBound="FB2N0" fbc:upperFluxBound="FB3N1000">

<listOfReactants>

<speciesReference species="M_CO2__91__d__93__" stoichiometry="1" constant="true"/>

<speciesReference species="M_H2O__91__d__93__" stoichiometry="1" constant="true"/>

<speciesReference species="M_PEP__91__d__93__" stoichiometry="1" constant="true"/>

</listOfReactants>

<listOfProducts>

<speciesReference species="M_Oxaloacetate__91__d__93__" stoichiometry="1" constant="true"/>

<speciesReference species="M_Pi__91__d__93__" stoichiometry="1" constant="true"/>

</listOfProducts>

</reaction>

<reaction metaid="CR2" id="CR2" name="CR2" reversible="true" fast="false" fbc:lowerFluxBound="FB1N1000" fbc:upperFluxBound="FB3N1000">

<listOfReactants>

<speciesReference species="M_ATP__91__d__93____91__c__93__" stoichiometry="1" constant="true"/>

<speciesReference species="M_R5P__91__d__93____91__c__93__" stoichiometry="1" constant="true"/>

</listOfReactants>

<listOfProducts>

<speciesReference species="M_ADP__91__d__93____91__c__93__" stoichiometry="1" constant="true"/>

<speciesReference species="M_RBP__91__d__93____91__c__93__" stoichiometry="1" constant="true"/>

</listOfProducts>

</reaction>

<reaction metaid="CR2c" id="CR2c" name="CR2c" reversible="true" fast="false" fbc:lowerFluxBound="FB1N1000" fbc:upperFluxBound="FB3N1000">

<listOfReactants>

<speciesReference species="M_ATP__91__d__93__" stoichiometry="1" constant="true"/>

<speciesReference species="M_R5P__91__d__93__" stoichiometry="1" constant="true"/>

</listOfReactants>

<listOfProducts>

<speciesReference species="M_ADP__91__d__93__" stoichiometry="1" constant="true"/>

<speciesReference species="M_RBP__91__d__93__" stoichiometry="1" constant="true"/>

</listOfProducts>

</reaction>

<reaction metaid="FDHm" id="FDHm" name="FDHm" reversible="false" fast="false" fbc:lowerFluxBound="FB2N0" fbc:upperFluxBound="FB3N1000">

<listOfReactants>

<speciesReference species="M_Formate__91__d__93____91__m__93__" stoichiometry="1" constant="true"/>

<speciesReference species="M_NAD__91__d__93____91__m__93__" stoichiometry="1" constant="true"/>

</listOfReactants>

<listOfProducts>

<speciesReference species="M_CO2__91__d__93__" stoichiometry="1" constant="true"/>

<speciesReference species="M_NADH__91__d__93____91__m__93__" stoichiometry="1" constant="true"/>

</listOfProducts>

</reaction>

<reaction metaid="FDH" id="FDH" name="FDH" reversible="false" fast="false" fbc:lowerFluxBound="FB2N0" fbc:upperFluxBound="FB3N1000">

<listOfReactants>

<speciesReference species="M_Formate__91__d__93____91__c__93__" stoichiometry="1" constant="true"/>

<speciesReference species="M_NAD__91__d__93____91__c__93__" stoichiometry="1" constant="true"/>

</listOfReactants>

<listOfProducts>

<speciesReference species="M_CO2__91__d__93__" stoichiometry="1" constant="true"/>

<speciesReference species="M_NADH__91__d__93____91__c__93__" stoichiometry="1" constant="true"/>

</listOfProducts>

</reaction>

<reaction metaid="FDHc" id="FDHc" name="FDHc" reversible="false" fast="false" fbc:lowerFluxBound="FB2N0" fbc:upperFluxBound="FB3N1000">

<listOfReactants>

<speciesReference species="M_Formate__91__d__93__" stoichiometry="1" constant="true"/>

<speciesReference species="M_NAD__91__d__93__" stoichiometry="1" constant="true"/>

</listOfReactants>

<listOfProducts>

<speciesReference species="M_CO2__91__d__93__" stoichiometry="1" constant="true"/>

<speciesReference species="M_NADH__91__d__93__" stoichiometry="1" constant="true"/>

</listOfProducts>

</reaction>

<reaction metaid="ACS" id="ACS" name="ACS" reversible="true" fast="false" fbc:lowerFluxBound="FB1N1000" fbc:upperFluxBound="FB3N1000">

<listOfReactants>

<speciesReference species="M_ATP__91__d__93____91__c__93__" stoichiometry="1" constant="true"/>

<speciesReference species="M_Acetate__91__d__93____91__c__93__" stoichiometry="1" constant="true"/>

<speciesReference species="M_CoenzymeA__91__d__93____91__c__93__" stoichiometry="1" constant="true"/>

</listOfReactants>

<listOfProducts>

<speciesReference species="M_AMP__91__d__93____91__c__93__" stoichiometry="1" constant="true"/>

<speciesReference species="M_AcetylCoA__91__d__93____91__c__93__" stoichiometry="1" constant="true"/>

<speciesReference species="M_PPi__91__d__93____91__c__93__" stoichiometry="1" constant="true"/>

</listOfProducts>

</reaction>

<reaction metaid="ACSm" id="ACSm" name="ACSm" reversible="true" fast="false" fbc:lowerFluxBound="FB1N1000" fbc:upperFluxBound="FB3N1000">

<listOfReactants>

<speciesReference species="M_ATP__91__d__93____91__m__93__" stoichiometry="1" constant="true"/>

<speciesReference species="M_Acetate__91__d__93____91__m__93__" stoichiometry="1" constant="true"/>

<speciesReference species="M_CoenzymeA__91__d__93____91__m__93__" stoichiometry="1" constant="true"/>

</listOfReactants>

<listOfProducts>

<speciesReference species="M_AMP__91__d__93____91__m__93__" stoichiometry="1" constant="true"/>

<speciesReference species="M_AcetylCoA__91__d__93____91__m__93__" stoichiometry="1" constant="true"/>

<speciesReference species="M_PPi__91__d__93____91__m__93__" stoichiometry="1" constant="true"/>

</listOfProducts>

</reaction>

<reaction metaid="CLc" id="CLc" name="CLc" reversible="false" fast="false" fbc:lowerFluxBound="FB2N0" fbc:upperFluxBound="FB3N1000">

<listOfReactants>

<speciesReference species="M_ATP__91__d__93__" stoichiometry="1" constant="true"/>

<speciesReference species="M_Citrate__91__d__93__" stoichiometry="1" constant="true"/>

<speciesReference species="M_CoenzymeA__91__d__93__" stoichiometry="1" constant="true"/>

</listOfReactants>

<listOfProducts>

<speciesReference species="M_ADP__91__d__93__" stoichiometry="1" constant="true"/>

<speciesReference species="M_AcetylCoA__91__d__93__" stoichiometry="1" constant="true"/>

<speciesReference species="M_Oxaloacetate__91__d__93__" stoichiometry="1" constant="true"/>

<speciesReference species="M_Pi__91__d__93__" stoichiometry="1" constant="true"/>

</listOfProducts>

</reaction>

<reaction metaid="MEyc" id="MEyc" name="MEyc" reversible="false" fast="false" fbc:lowerFluxBound="FB2N0" fbc:upperFluxBound="FB3N1000">

<listOfReactants>

<speciesReference species="M_Malate__91__d__93__" stoichiometry="1" constant="true"/>

<speciesReference species="M_NADP__91__d__93__" stoichiometry="1" constant="true"/>

</listOfReactants>

<listOfProducts>

<speciesReference species="M_CO2__91__d__93__" stoichiometry="1" constant="true"/>

<speciesReference species="M_NADPH__91__d__93__" stoichiometry="1" constant="true"/>

<speciesReference species="M_Pyruvate__91__d__93__" stoichiometry="1" constant="true"/>

</listOfProducts>

</reaction>

<reaction metaid="MEc" id="MEc" name="MEc" reversible="false" fast="false" fbc:lowerFluxBound="FB2N0" fbc:upperFluxBound="FB3N1000">

<listOfReactants>

<speciesReference species="M_Malate__91__d__93__" stoichiometry="1" constant="true"/>

<speciesReference species="M_NAD__91__d__93__" stoichiometry="1" constant="true"/>

</listOfReactants>

<listOfProducts>

<speciesReference species="M_CO2__91__d__93__" stoichiometry="1" constant="true"/>

<speciesReference species="M_NADH__91__d__93__" stoichiometry="1" constant="true"/>

<speciesReference species="M_Pyruvate__91__d__93__" stoichiometry="1" constant="true"/>

</listOfProducts>

</reaction>

<reaction metaid="MEy" id="MEy" name="MEy" reversible="false" fast="false" fbc:lowerFluxBound="FB2N0" fbc:upperFluxBound="FB3N1000">

<listOfReactants>

<speciesReference species="M_Malate__91__d__93____91__c__93__" stoichiometry="1" constant="true"/>

<speciesReference species="M_NADP__91__d__93____91__c__93__" stoichiometry="1" constant="true"/>

</listOfReactants>

<listOfProducts>

<speciesReference species="M_CO2__91__d__93__" stoichiometry="1" constant="true"/>

<speciesReference species="M_NADPH__91__d__93____91__c__93__" stoichiometry="1" constant="true"/>

<speciesReference species="M_Pyruvate__91__d__93____91__c__93__" stoichiometry="1" constant="true"/>

</listOfProducts>

</reaction>

<reaction metaid="ME" id="ME" name="ME" reversible="false" fast="false" fbc:lowerFluxBound="FB2N0" fbc:upperFluxBound="FB3N1000">

<listOfReactants>

<speciesReference species="M_Malate__91__d__93____91__c__93__" stoichiometry="1" constant="true"/>

<speciesReference species="M_NAD__91__d__93____91__c__93__" stoichiometry="1" constant="true"/>

</listOfReactants>

<listOfProducts>

<speciesReference species="M_CO2__91__d__93__" stoichiometry="1" constant="true"/>

<speciesReference species="M_NADH__91__d__93____91__c__93__" stoichiometry="1" constant="true"/>

<speciesReference species="M_Pyruvate__91__d__93____91__c__93__" stoichiometry="1" constant="true"/>

</listOfProducts>

</reaction>

<reaction metaid="MEm" id="MEm" name="MEm" reversible="false" fast="false" fbc:lowerFluxBound="FB2N0" fbc:upperFluxBound="FB3N1000">

<listOfReactants>

<speciesReference species="M_Malate__91__d__93____91__m__93__" stoichiometry="1" constant="true"/>

<speciesReference species="M_NAD__91__d__93____91__m__93__" stoichiometry="1" constant="true"/>

</listOfReactants>

<listOfProducts>

<speciesReference species="M_CO2__91__d__93__" stoichiometry="1" constant="true"/>

<speciesReference species="M_NADH__91__d__93____91__m__93__" stoichiometry="1" constant="true"/>

<speciesReference species="M_Pyruvate__91__d__93____91__m__93__" stoichiometry="1" constant="true"/>

</listOfProducts>

</reaction>

<reaction metaid="CP1" id="CP1" name="CP1" reversible="true" fast="false" fbc:lowerFluxBound="FB1N1000" fbc:upperFluxBound="FB3N1000">

<listOfReactants>

<speciesReference species="M_glucose__91__d__93__" stoichiometry="1" constant="true"/>

</listOfReactants>

<listOfProducts>

<speciesReference species="M_fructose__91__d__93__" stoichiometry="1" constant="true"/>

</listOfProducts>

</reaction>

<reaction metaid="CP2" id="CP2" name="CP2" reversible="true" fast="false" fbc:lowerFluxBound="FB1N1000" fbc:upperFluxBound="FB3N1000">

<listOfReactants>

<speciesReference species="M_UDP_glucose__91__d__93__" stoichiometry="1" constant="true"/>

<speciesReference species="M_fructose__91__d__93__" stoichiometry="1" constant="true"/>

</listOfReactants>

<listOfProducts>

<speciesReference species="M_UDP__91__d__93__" stoichiometry="1" constant="true"/>

<speciesReference species="M_sucrose__91__d__93__" stoichiometry="1" constant="true"/>

</listOfProducts>

</reaction>

<reaction metaid="CP3" id="CP3" name="CP3" reversible="true" fast="false" fbc:lowerFluxBound="FB1N1000" fbc:upperFluxBound="FB3N1000">

<listOfReactants>

<speciesReference species="M_G1P__91__d__93__" stoichiometry="1" constant="true"/>

<speciesReference species="M_UMP__91__d__93__" stoichiometry="1" constant="true"/>

</listOfReactants>

<listOfProducts>

<speciesReference species="M_H2O__91__d__93__" stoichiometry="1" constant="true"/>

<speciesReference species="M_UDP_glucose__91__d__93__" stoichiometry="1" constant="true"/>

</listOfProducts>

</reaction>

<reaction metaid="CP4" id="CP4" name="CP4" reversible="true" fast="false" fbc:lowerFluxBound="FB1N1000" fbc:upperFluxBound="FB3N1000">

<listOfReactants>

<speciesReference species="M_G6P__91__d__93__" stoichiometry="1" constant="true"/>

</listOfReactants>

<listOfProducts>

<speciesReference species="M_G1P__91__d__93__" stoichiometry="1" constant="true"/>

</listOfProducts>

</reaction>

<reaction metaid="CP5" id="CP5" name="CP5" reversible="true" fast="false" fbc:lowerFluxBound="FB1N1000" fbc:upperFluxBound="FB3N1000">

<listOfReactants>

<speciesReference species="M_H2O__91__d__93__" stoichiometry="1" constant="true"/>

<speciesReference species="M_UDP__91__d__93__" stoichiometry="1" constant="true"/>

</listOfReactants>

<listOfProducts>

<speciesReference species="M_Pi__91__d__93__" stoichiometry="1" constant="true"/>

<speciesReference species="M_UMP__91__d__93__" stoichiometry="1" constant="true"/>

</listOfProducts>

</reaction>

<reaction metaid="CP6" id="CP6" name="CP6" reversible="false" fast="false" fbc:lowerFluxBound="FB2N0" fbc:upperFluxBound="FB3N1000">

<listOfReactants>

<speciesReference species="M_fructose__91__d__93__" stoichiometry="0.037" constant="true"/>

<speciesReference species="M_glucose__91__d__93__" stoichiometry="0.032" constant="true"/>

<speciesReference species="M_sucrose__91__d__93__" stoichiometry="0.0487" constant="true"/>

</listOfReactants>

<listOfProducts>

<speciesReference species="M_sugars__91__d__93__" stoichiometry="1" constant="true"/>

</listOfProducts>

</reaction>

<reaction metaid="CP7" id="CP7" name="CP7" reversible="false" fast="false" fbc:lowerFluxBound="FB2N0" fbc:upperFluxBound="FB3N1000">

<listOfReactants>

<speciesReference species="M_ATP__91__d__93__" stoichiometry="0.167" constant="true"/>

<speciesReference species="M_G6P__91__d__93__" stoichiometry="0.167" constant="true"/>

</listOfReactants>

<listOfProducts>

<speciesReference species="M_ADP__91__d__93__" stoichiometry="0.167" constant="true"/>

<speciesReference species="M_PPi__91__d__93__" stoichiometry="0.167" constant="true"/>

<speciesReference species="M_fibers__91__d__93__" stoichiometry="1" constant="true"/>

</listOfProducts>

</reaction>

<reaction metaid="Ser_bio_cl" id="Ser_bio_cl" name="Ser_bio_cl" reversible="false" fast="false" fbc:lowerFluxBound="FB2N0" fbc:upperFluxBound="FB3N1000">

<listOfReactants>

<speciesReference species="M_3PGA__91__d__93____91__c__93__" stoichiometry="1" constant="true"/>

<speciesReference species="M_Glutamate__91__d__93____91__c__93__" stoichiometry="1" constant="true"/>

<speciesReference species="M_H2O__91__d__93__" stoichiometry="1" constant="true"/>

<speciesReference species="M_NAD__91__d__93____91__c__93__" stoichiometry="1" constant="true"/>

</listOfReactants>

<listOfProducts>

<speciesReference species="M_NADH__91__d__93____91__c__93__" stoichiometry="1" constant="true"/>

<speciesReference species="M_Oxoglutarate__91__d__93____91__c__93__" stoichiometry="1" constant="true"/>

<speciesReference species="M_Pi__91__d__93____91__c__93__" stoichiometry="1" constant="true"/>

<speciesReference species="M_Serine__91__d__93____91__c__93__" stoichiometry="1" constant="true"/>

</listOfProducts>

</reaction>

<reaction metaid="Ser_bio_cyt" id="Ser_bio_cyt" name="Ser_bio_cyt" reversible="false" fast="false" fbc:lowerFluxBound="FB2N0" fbc:upperFluxBound="FB3N1000">

<listOfReactants>

<speciesReference species="M_3PGA__91__d__93__" stoichiometry="1" constant="true"/>

<speciesReference species="M_Alanine__91__d__93__" stoichiometry="1" constant="true"/>

<speciesReference species="M_H2O__91__d__93__" stoichiometry="1" constant="true"/>

<speciesReference species="M_NAD__91__d__93__" stoichiometry="1" constant="true"/>

</listOfReactants>

<listOfProducts>

<speciesReference species="M_NADH__91__d__93__" stoichiometry="1" constant="true"/>

<speciesReference species="M_Pi__91__d__93__" stoichiometry="1" constant="true"/>

<speciesReference species="M_Pyruvate__91__d__93__" stoichiometry="1" constant="true"/>

<speciesReference species="M_Serine__91__d__93__" stoichiometry="1" constant="true"/>

</listOfProducts>

</reaction>

<reaction metaid="AsnA" id="AsnA" name="AsnA" reversible="true" fast="false" fbc:lowerFluxBound="FB1N1000" fbc:upperFluxBound="FB3N1000">

<listOfReactants>

<speciesReference species="M_Aspartate__91__d__93__" stoichiometry="1" constant="true"/>

<speciesReference species="M_NH3__91__d__93__" stoichiometry="1" constant="true"/>

</listOfReactants>

<listOfProducts>

<speciesReference species="M_Asparagine__91__d__93__" stoichiometry="1" constant="true"/>

<speciesReference species="M_H2O__91__d__93__" stoichiometry="1" constant="true"/>

</listOfProducts>

</reaction>

<reaction metaid="HSK" id="HSK" name="HSK" reversible="true" fast="false" fbc:lowerFluxBound="FB1N1000" fbc:upperFluxBound="FB3N1000">

<listOfReactants>

<speciesReference species="M_ATP__91__d__93____91__c__93__" stoichiometry="1" constant="true"/>

<speciesReference species="M_Homoserine__91__d__93____91__c__93__" stoichiometry="1" constant="true"/>

</listOfReactants>

<listOfProducts>

<speciesReference species="M_ADP__91__d__93____91__c__93__" stoichiometry="1" constant="true"/>

<speciesReference species="M_Phosphohomoserine__91__d__93____91__c__93__" stoichiometry="1" constant="true"/>

</listOfProducts>

</reaction>

<reaction metaid="Threonine_synthase" id="Threonine_synthase" name="Threonine_synthase" reversible="true" fast="false" fbc:lowerFluxBound="FB1N1000" fbc:upperFluxBound="FB3N1000">

<listOfReactants>

<speciesReference species="M_H2O__91__d__93__" stoichiometry="1" constant="true"/>

<speciesReference species="M_Phosphohomoserine__91__d__93____91__c__93__" stoichiometry="1" constant="true"/>

</listOfReactants>

<listOfProducts>

<speciesReference species="M_Pi__91__d__93____91__c__93__" stoichiometry="1" constant="true"/>

<speciesReference species="M_Threonine__91__d__93____91__c__93__" stoichiometry="1" constant="true"/>

</listOfProducts>

</reaction>

<reaction metaid="IleBiosynth" id="IleBiosynth" name="IleBiosynth" reversible="true" fast="false" fbc:lowerFluxBound="FB1N1000" fbc:upperFluxBound="FB3N1000">

<listOfReactants>

<speciesReference species="M_2Oxobutanoate__91__d__93____91__c__93__" stoichiometry="1" constant="true"/>

<speciesReference species="M_Glutamate__91__d__93____91__c__93__" stoichiometry="1" constant="true"/>

<speciesReference species="M_NADPH__91__d__93____91__c__93__" stoichiometry="1" constant="true"/>

<speciesReference species="M_Pyruvate__91__d__93____91__c__93__" stoichiometry="1" constant="true"/>

</listOfReactants>

<listOfProducts>

<speciesReference species="M_CO2__91__d__93__" stoichiometry="1" constant="true"/>

<speciesReference species="M_H2O__91__d__93__" stoichiometry="1" constant="true"/>

<speciesReference species="M_Isoleucine__91__d__93____91__c__93__" stoichiometry="1" constant="true"/>

<speciesReference species="M_NADP__91__d__93____91__c__93__" stoichiometry="1" constant="true"/>

<speciesReference species="M_Oxoglutarate__91__d__93____91__c__93__" stoichiometry="1" constant="true"/>

</listOfProducts>

</reaction>

<reaction metaid="TransaminaseB" id="TransaminaseB" name="TransaminaseB" reversible="false" fast="false" fbc:lowerFluxBound="FB2N0" fbc:upperFluxBound="FB3N1000">

<listOfReactants>

<speciesReference species="M_1_2Oxoisovalerate__91__d__93____91__c__93__" stoichiometry="1" constant="true"/>

<speciesReference species="M_Glutamate__91__d__93____91__c__93__" stoichiometry="1" constant="true"/>

</listOfReactants>

<listOfProducts>

<speciesReference species="M_Oxoglutarate__91__d__93____91__c__93__" stoichiometry="1" constant="true"/>

<speciesReference species="M_Valine__91__d__93____91__c__93__" stoichiometry="1" constant="true"/>

</listOfProducts>

</reaction>

<reaction metaid="GPT" id="GPT" name="GPT" reversible="false" fast="false" fbc:lowerFluxBound="FB2N0" fbc:upperFluxBound="FB3N1000">

<listOfReactants>

<speciesReference species="M_Glutamate__91__d__93____91__c__93__" stoichiometry="1" constant="true"/>

<speciesReference species="M_Pyruvate__91__d__93____91__c__93__" stoichiometry="1" constant="true"/>

</listOfReactants>

<listOfProducts>

<speciesReference species="M_Alanine__91__d__93____91__c__93__" stoichiometry="1" constant="true"/>

<speciesReference species="M_Oxoglutarate__91__d__93____91__c__93__" stoichiometry="1" constant="true"/>

</listOfProducts>

</reaction>

<reaction metaid="LeuBiosynth" id="LeuBiosynth" name="LeuBiosynth" reversible="false" fast="false" fbc:lowerFluxBound="FB2N0" fbc:upperFluxBound="FB3N1000">

<listOfReactants>

<speciesReference species="M_1_2Oxoisovalerate__91__d__93____91__c__93__" stoichiometry="1" constant="true"/>

<speciesReference species="M_AcetylCoA__91__d__93____91__c__93__" stoichiometry="1" constant="true"/>

<speciesReference species="M_Glutamate__91__d__93____91__c__93__" stoichiometry="1" constant="true"/>

<speciesReference species="M_H2O__91__d__93__" stoichiometry="1" constant="true"/>

<speciesReference species="M_NAD__91__d__93____91__c__93__" stoichiometry="1" constant="true"/>

</listOfReactants>

<listOfProducts>

<speciesReference species="M_CO2__91__d__93__" stoichiometry="1" constant="true"/>

<speciesReference species="M_CoenzymeA__91__d__93____91__c__93__" stoichiometry="1" constant="true"/>

<speciesReference species="M_Leucine__91__d__93____91__c__93__" stoichiometry="1" constant="true"/>

<speciesReference species="M_NADH__91__d__93____91__c__93__" stoichiometry="1" constant="true"/>

<speciesReference species="M_Oxoglutarate__91__d__93____91__c__93__" stoichiometry="1" constant="true"/>

</listOfProducts>

</reaction>

<reaction metaid="CysBiosynth" id="CysBiosynth" name="CysBiosynth" reversible="false" fast="false" fbc:lowerFluxBound="FB2N0" fbc:upperFluxBound="FB3N1000">

<listOfReactants>

<speciesReference species="M_Homocysteine__91__d__93____91__c__93__" stoichiometry="1" constant="true"/>

<speciesReference species="M_Serine__91__d__93____91__c__93__" stoichiometry="1" constant="true"/>

</listOfReactants>

<listOfProducts>

<speciesReference species="M_2Oxobutanoate__91__d__93____91__c__93__" stoichiometry="1" constant="true"/>

<speciesReference species="M_Cysteine__91__d__93____91__c__93__" stoichiometry="1" constant="true"/>

<speciesReference species="M_NH3__91__d__93____91__c__93__" stoichiometry="1" constant="true"/>

</listOfProducts>

</reaction>

<reaction metaid="CysOxidation" id="CysOxidation" name="CysOxidation" reversible="true" fast="false" fbc:lowerFluxBound="FB1N1000" fbc:upperFluxBound="FB3N1000">

<listOfReactants>

<speciesReference species="M_Cysteine__91__d__93____91__c__93__" stoichiometry="2" constant="true"/>

<speciesReference species="M_NAD__91__d__93____91__c__93__" stoichiometry="1" constant="true"/>

</listOfReactants>

<listOfProducts>

<speciesReference species="M_Cystine__91__d__93____91__c__93__" stoichiometry="1" constant="true"/>

<speciesReference species="M_NADH__91__d__93____91__c__93__" stoichiometry="1" constant="true"/>

</listOfProducts>

</reaction>

<reaction metaid="SHMTm" id="SHMTm" name="SHMTm" reversible="true" fast="false" fbc:lowerFluxBound="FB1N1000" fbc:upperFluxBound="FB3N1000">

<listOfReactants>

<speciesReference species="M_Glycine__91__d__93____91__m__93__" stoichiometry="1" constant="true"/>

<speciesReference species="M_NAD__91__d__93____91__m__93__" stoichiometry="1" constant="true"/>

<speciesReference species="M_THF__91__d__93____91__m__93__" stoichiometry="1" constant="true"/>

</listOfReactants>

<listOfProducts>

<speciesReference species="M_CH2THF__91__d__93____91__m__93__" stoichiometry="1" constant="true"/>

<speciesReference species="M_CO2__91__d__93__" stoichiometry="1" constant="true"/>

<speciesReference species="M_NADH__91__d__93____91__m__93__" stoichiometry="1" constant="true"/>

<speciesReference species="M_NH3__91__d__93____91__m__93__" stoichiometry="1" constant="true"/>

</listOfProducts>

</reaction>

<reaction metaid="SHMTm2" id="SHMTm2" name="SHMTm2" reversible="true" fast="false" fbc:lowerFluxBound="FB1N1000" fbc:upperFluxBound="FB3N1000">

<listOfReactants>

<speciesReference species="M_CH2THF__91__d__93____91__m__93__" stoichiometry="1" constant="true"/>

<speciesReference species="M_Glycine__91__d__93____91__m__93__" stoichiometry="1" constant="true"/>

</listOfReactants>

<listOfProducts>

<speciesReference species="M_Serine__91__d__93____91__m__93__" stoichiometry="1" constant="true"/>

<speciesReference species="M_THF__91__d__93____91__m__93__" stoichiometry="1" constant="true"/>

</listOfProducts>

</reaction>

<reaction metaid="SHMT_cyt" id="SHMT_cyt" name="SHMT_cyt" reversible="true" fast="false" fbc:lowerFluxBound="FB1N1000" fbc:upperFluxBound="FB3N1000">

<listOfReactants>

<speciesReference species="M_CH2THF__91__d__93__" stoichiometry="1" constant="true"/>

<speciesReference species="M_Glycine__91__d__93__" stoichiometry="1" constant="true"/>

</listOfReactants>

<listOfProducts>

<speciesReference species="M_Serine__91__d__93__" stoichiometry="1" constant="true"/>

<speciesReference species="M_THF__91__d__93__" stoichiometry="1" constant="true"/>

</listOfProducts>

</reaction>

<reaction metaid="GOGAT" id="GOGAT" name="GOGAT" reversible="false" fast="false" fbc:lowerFluxBound="FB2N0" fbc:upperFluxBound="FB3N1000">

<listOfReactants>

<speciesReference species="M_Glutamine__91__d__93____91__c__93__" stoichiometry="1" constant="true"/>

<speciesReference species="M_NADH__91__d__93____91__c__93__" stoichiometry="1" constant="true"/>

<speciesReference species="M_Oxoglutarate__91__d__93____91__c__93__" stoichiometry="1" constant="true"/>

</listOfReactants>

<listOfProducts>

<speciesReference species="M_Glutamate__91__d__93____91__c__93__" stoichiometry="2" constant="true"/>

<speciesReference species="M_NAD__91__d__93____91__c__93__" stoichiometry="1" constant="true"/>

</listOfProducts>

</reaction>

<reaction metaid="GSAc" id="GSAc" name="GSAc" reversible="false" fast="false" fbc:lowerFluxBound="FB2N0" fbc:upperFluxBound="FB3N1000">

<listOfReactants>

<speciesReference species="M_ATP__91__d__93____91__c__93__" stoichiometry="1" constant="true"/>

<speciesReference species="M_Glutamate__91__d__93____91__c__93__" stoichiometry="1" constant="true"/>

<speciesReference species="M_NADPH__91__d__93____91__c__93__" stoichiometry="1" constant="true"/>

</listOfReactants>

<listOfProducts>

<speciesReference species="M_ADP__91__d__93____91__c__93__" stoichiometry="1" constant="true"/>

<speciesReference species="M_Glutamate_y_Semialdehyde__91__d__93____91__c__93__" stoichiometry="1" constant="true"/>

<speciesReference species="M_NADP__91__d__93____91__c__93__" stoichiometry="1" constant="true"/>

<speciesReference species="M_Pi__91__d__93____91__c__93__" stoichiometry="1" constant="true"/>

</listOfProducts>

</reaction>

<reaction metaid="NonEc" id="NonEc" name="NonEc" reversible="false" fast="false" fbc:lowerFluxBound="FB2N0" fbc:upperFluxBound="FB3N1000">

<listOfReactants>

<speciesReference species="M_Glutamate_y_Semialdehyde__91__d__93____91__c__93__" stoichiometry="1" constant="true"/>

</listOfReactants>

<listOfProducts>

<speciesReference species="M_1_pyrroline_5_Carboxylate__91__d__93____91__c__93__" stoichiometry="1" constant="true"/>

<speciesReference species="M_H2O__91__d__93__" stoichiometry="1" constant="true"/>

</listOfProducts>

</reaction>

<reaction metaid="ProlineOxidasec" id="ProlineOxidasec" name="ProlineOxidasec" reversible="false" fast="false" fbc:lowerFluxBound="FB2N0" fbc:upperFluxBound="FB3N1000">

<listOfReactants>

<speciesReference species="M_1_pyrroline_5_Carboxylate__91__d__93____91__c__93__" stoichiometry="1" constant="true"/>

<speciesReference species="M_NADPH__91__d__93____91__c__93__" stoichiometry="1" constant="true"/>

</listOfReactants>

<listOfProducts>

<speciesReference species="M_NADP__91__d__93____91__c__93__" stoichiometry="1" constant="true"/>

<speciesReference species="M_Proline__91__d__93____91__c__93__" stoichiometry="1" constant="true"/>

</listOfProducts>

</reaction>

<reaction metaid="GSA" id="GSA" name="GSA" reversible="false" fast="false" fbc:lowerFluxBound="FB2N0" fbc:upperFluxBound="FB3N1000">

<listOfReactants>

<speciesReference species="M_ATP__91__d__93__" stoichiometry="1" constant="true"/>

<speciesReference species="M_Glutamate__91__d__93__" stoichiometry="1" constant="true"/>

<speciesReference species="M_NADPH__91__d__93__" stoichiometry="1" constant="true"/>

</listOfReactants>

<listOfProducts>

<speciesReference species="M_ADP__91__d__93__" stoichiometry="1" constant="true"/>

<speciesReference species="M_Glutamate_y_Semialdehyde__91__d__93__" stoichiometry="1" constant="true"/>

<speciesReference species="M_NADP__91__d__93__" stoichiometry="1" constant="true"/>

<speciesReference species="M_Pi__91__d__93__" stoichiometry="1" constant="true"/>

</listOfProducts>

</reaction>

<reaction metaid="NonE" id="NonE" name="NonE" reversible="false" fast="false" fbc:lowerFluxBound="FB2N0" fbc:upperFluxBound="FB3N1000">

<listOfReactants>

<speciesReference species="M_Glutamate_y_Semialdehyde__91__d__93__" stoichiometry="1" constant="true"/>

</listOfReactants>

<listOfProducts>

<speciesReference species="M_1_pyrroline_5_Carboxylate__91__d__93__" stoichiometry="1" constant="true"/>

<speciesReference species="M_H2O__91__d__93__" stoichiometry="1" constant="true"/>

</listOfProducts>

</reaction>

<reaction metaid="ProlineOxidase" id="ProlineOxidase" name="ProlineOxidase" reversible="false" fast="false" fbc:lowerFluxBound="FB2N0" fbc:upperFluxBound="FB3N1000">

<listOfReactants>

<speciesReference species="M_1_pyrroline_5_Carboxylate__91__d__93__" stoichiometry="1" constant="true"/>

<speciesReference species="M_NADPH__91__d__93__" stoichiometry="1" constant="true"/>

</listOfReactants>

<listOfProducts>

<speciesReference species="M_NADP__91__d__93__" stoichiometry="1" constant="true"/>

<speciesReference species="M_Proline__91__d__93__" stoichiometry="1" constant="true"/>

</listOfProducts>

</reaction>

<reaction metaid="OAT" id="OAT" name="OAT" reversible="true" fast="false" fbc:lowerFluxBound="FB1N1000" fbc:upperFluxBound="FB3N1000">

<listOfReactants>

<speciesReference species="M_Glutamate__91__d__93____91__c__93__" stoichiometry="1" constant="true"/>

<speciesReference species="M_Glutamate_y_Semialdehyde__91__d__93____91__c__93__" stoichiometry="1" constant="true"/>

</listOfReactants>

<listOfProducts>

<speciesReference species="M_Ornithine__91__d__93____91__c__93__" stoichiometry="1" constant="true"/>

<speciesReference species="M_Oxoglutarate__91__d__93____91__c__93__" stoichiometry="1" constant="true"/>

</listOfProducts>

</reaction>

<reaction metaid="OTC" id="OTC" name="OTC" reversible="true" fast="false" fbc:lowerFluxBound="FB1N1000" fbc:upperFluxBound="FB3N1000">

<listOfReactants>

<speciesReference species="M_CO2__91__d__93__" stoichiometry="1" constant="true"/>

<speciesReference species="M_NH3__91__d__93____91__c__93__" stoichiometry="1" constant="true"/>

<speciesReference species="M_Ornithine__91__d__93____91__c__93__" stoichiometry="1" constant="true"/>

</listOfReactants>

<listOfProducts>

<speciesReference species="M_Citrulline__91__d__93____91__c__93__" stoichiometry="1" constant="true"/>

<speciesReference species="M_H2O__91__d__93__" stoichiometry="1" constant="true"/>

</listOfProducts>

</reaction>

<reaction metaid="ASSASL" id="ASSASL" name="ASSASL" reversible="true" fast="false" fbc:lowerFluxBound="FB1N1000" fbc:upperFluxBound="FB3N1000">

<listOfReactants>

<speciesReference species="M_ATP__91__d__93____91__c__93__" stoichiometry="1" constant="true"/>

<speciesReference species="M_Aspartate__91__d__93____91__c__93__" stoichiometry="1" constant="true"/>

<speciesReference species="M_Citrulline__91__d__93____91__c__93__" stoichiometry="1" constant="true"/>

</listOfReactants>

<listOfProducts>

<speciesReference species="M_AMP__91__d__93____91__c__93__" stoichiometry="1" constant="true"/>

<speciesReference species="M_Arginine__91__d__93____91__c__93__" stoichiometry="1" constant="true"/>

<speciesReference species="M_Fumarate__91__d__93__" stoichiometry="1" constant="true"/>

<speciesReference species="M_PPi__91__d__93____91__c__93__" stoichiometry="1" constant="true"/>

</listOfProducts>

</reaction>

<reaction metaid="Shikimate" id="Shikimate" name="Shikimate" reversible="false" fast="false" fbc:lowerFluxBound="FB2N0" fbc:upperFluxBound="FB3N1000">

<listOfReactants>

<speciesReference species="M_ATP__91__d__93____91__c__93__" stoichiometry="1" constant="true"/>

<speciesReference species="M_E4P__91__d__93____91__c__93__" stoichiometry="1" constant="true"/>

<speciesReference species="M_NADPH__91__d__93____91__c__93__" stoichiometry="1" constant="true"/>

<speciesReference species="M_PEP__91__d__93____91__c__93__" stoichiometry="2" constant="true"/>

</listOfReactants>

<listOfProducts>

<speciesReference species="M_ADP__91__d__93____91__c__93__" stoichiometry="1" constant="true"/>

<speciesReference species="M_Chorismate__91__d__93____91__c__93__" stoichiometry="1" constant="true"/>

<speciesReference species="M_NADP__91__d__93____91__c__93__" stoichiometry="1" constant="true"/>

<speciesReference species="M_Pi__91__d__93____91__c__93__" stoichiometry="4" constant="true"/>

</listOfProducts>

</reaction>

<reaction metaid="PLP" id="PLP" name="PLP" reversible="true" fast="false" fbc:lowerFluxBound="FB1N1000" fbc:upperFluxBound="FB3N1000">

<listOfReactants>

<speciesReference species="M_4_HydroxyPhenylpyruvate__91__d__93____91__c__93__" stoichiometry="1" constant="true"/>

<speciesReference species="M_Glutamate__91__d__93____91__c__93__" stoichiometry="1" constant="true"/>

</listOfReactants>

<listOfProducts>

<speciesReference species="M_Oxoglutarate__91__d__93____91__c__93__" stoichiometry="1" constant="true"/>

<speciesReference species="M_Tyrosine__91__d__93____91__c__93__" stoichiometry="1" constant="true"/>

</listOfProducts>

</reaction>

<reaction metaid="ChorismateMutase" id="ChorismateMutase" name="ChorismateMutase" reversible="true" fast="false" fbc:lowerFluxBound="FB1N1000" fbc:upperFluxBound="FB3N1000">

<listOfReactants>

<speciesReference species="M_Chorismate__91__d__93____91__c__93__" stoichiometry="1" constant="true"/>

<speciesReference species="M_NADP__91__d__93____91__c__93__" stoichiometry="1" constant="true"/>

</listOfReactants>

<listOfProducts>

<speciesReference species="M_4_HydroxyPhenylpyruvate__91__d__93____91__c__93__" stoichiometry="1" constant="true"/>

<speciesReference species="M_CO2__91__d__93__" stoichiometry="1" constant="true"/>

<speciesReference species="M_NADPH__91__d__93____91__c__93__" stoichiometry="1" constant="true"/>

</listOfProducts>

</reaction>

<reaction metaid="ChorismateMutasePrephenate" id="ChorismateMutasePrephenate" name="ChorismateMutasePrephenate" reversible="false" fast="false" fbc:lowerFluxBound="FB2N0" fbc:upperFluxBound="FB3N1000">

<listOfReactants>

<speciesReference species="M_Glutamate__91__d__93____91__c__93__" stoichiometry="1" constant="true"/>

<speciesReference species="M_Phenylpyruvate__91__d__93____91__c__93__" stoichiometry="1" constant="true"/>

</listOfReactants>

<listOfProducts>

<speciesReference species="M_Oxoglutarate__91__d__93____91__c__93__" stoichiometry="1" constant="true"/>

<speciesReference species="M_Phenylalanine__91__d__93____91__c__93__" stoichiometry="1" constant="true"/>

</listOfProducts>

</reaction>

<reaction metaid="TryptophanSynthase" id="TryptophanSynthase" name="TryptophanSynthase" reversible="false" fast="false" fbc:lowerFluxBound="FB2N0" fbc:upperFluxBound="FB3N1000">

<listOfReactants>

<speciesReference species="M_5p_Ribosyl_1_pp__91__d__93____91__c__93__" stoichiometry="1" constant="true"/>

<speciesReference species="M_Chorismate__91__d__93____91__c__93__" stoichiometry="1" constant="true"/>

<speciesReference species="M_Glutamine__91__d__93____91__c__93__" stoichiometry="1" constant="true"/>

<speciesReference species="M_Serine__91__d__93____91__c__93__" stoichiometry="1" constant="true"/>

</listOfReactants>

<listOfProducts>

<speciesReference species="M_G3P__91__d__93____91__c__93__" stoichiometry="1" constant="true"/>

<speciesReference species="M_Glutamate__91__d__93____91__c__93__" stoichiometry="1" constant="true"/>

<speciesReference species="M_H2O__91__d__93__" stoichiometry="2" constant="true"/>

<speciesReference species="M_Oxaloacetate__91__d__93____91__c__93__" stoichiometry="1" constant="true"/>

<speciesReference species="M_PPi__91__d__93____91__c__93__" stoichiometry="1" constant="true"/>

<speciesReference species="M_Tryptophan__91__d__93____91__c__93__" stoichiometry="1" constant="true"/>

</listOfProducts>

</reaction>

<reaction metaid="R15Pi" id="R15Pi" name="R15Pi" reversible="true" fast="false" fbc:lowerFluxBound="FB1N1000" fbc:upperFluxBound="FB3N1000">

<listOfReactants>

<speciesReference species="M_Pi__91__d__93____91__c__93__" stoichiometry="1" constant="true"/>

<speciesReference species="M_RBP__91__d__93____91__c__93__" stoichiometry="1" constant="true"/>

</listOfReactants>

<listOfProducts>

<speciesReference species="M_5p_Ribosyl_1_pp__91__d__93____91__c__93__" stoichiometry="1" constant="true"/>

<speciesReference species="M_H2O__91__d__93__" stoichiometry="1" constant="true"/>

</listOfProducts>

</reaction>

<reaction metaid="LysBiosinth" id="LysBiosinth" name="LysBiosinth" reversible="false" fast="false" fbc:lowerFluxBound="FB2N0" fbc:upperFluxBound="FB3N1000">

<listOfReactants>

<speciesReference species="M_Aspartate_semialdehyde__91__d__93____91__c__93__" stoichiometry="1" constant="true"/>

<speciesReference species="M_Glutamate__91__d__93____91__c__93__" stoichiometry="1" constant="true"/>

<speciesReference species="M_NADH__91__d__93____91__c__93__" stoichiometry="1" constant="true"/>

<speciesReference species="M_Pyruvate__91__d__93____91__c__93__" stoichiometry="1" constant="true"/>

</listOfReactants>

<listOfProducts>

<speciesReference species="M_CO2__91__d__93__" stoichiometry="1" constant="true"/>

<speciesReference species="M_Lysine__91__d__93____91__c__93__" stoichiometry="1" constant="true"/>

<speciesReference species="M_NAD__91__d__93____91__c__93__" stoichiometry="1" constant="true"/>

<speciesReference species="M_Oxoglutarate__91__d__93____91__c__93__" stoichiometry="1" constant="true"/>

</listOfProducts>

</reaction>

<reaction metaid="Asd" id="Asd" name="Asd" reversible="true" fast="false" fbc:lowerFluxBound="FB1N1000" fbc:upperFluxBound="FB3N1000">

<listOfReactants>

<speciesReference species="M_ATP__91__d__93____91__c__93__" stoichiometry="1" constant="true"/>

<speciesReference species="M_Aspartate__91__d__93____91__c__93__" stoichiometry="1" constant="true"/>

<speciesReference species="M_NADH__91__d__93____91__c__93__" stoichiometry="1" constant="true"/>

</listOfReactants>

<listOfProducts>

<speciesReference species="M_ADP__91__d__93____91__c__93__" stoichiometry="1" constant="true"/>

<speciesReference species="M_Aspartate_semialdehyde__91__d__93____91__c__93__" stoichiometry="1" constant="true"/>

<speciesReference species="M_NAD__91__d__93____91__c__93__" stoichiometry="1" constant="true"/>

<speciesReference species="M_Pi__91__d__93____91__c__93__" stoichiometry="1" constant="true"/>

</listOfProducts>

</reaction>

<reaction metaid="HomoserineDehydrogenase" id="HomoserineDehydrogenase" name="HomoserineDehydrogenase" reversible="true" fast="false" fbc:lowerFluxBound="FB1N1000" fbc:upperFluxBound="FB3N1000">

<listOfReactants>

<speciesReference species="M_Aspartate_semialdehyde__91__d__93____91__c__93__" stoichiometry="1" constant="true"/>

<speciesReference species="M_NADH__91__d__93____91__c__93__" stoichiometry="1" constant="true"/>

</listOfReactants>

<listOfProducts>

<speciesReference species="M_Homoserine__91__d__93____91__c__93__" stoichiometry="1" constant="true"/>

<speciesReference species="M_NAD__91__d__93____91__c__93__" stoichiometry="1" constant="true"/>

</listOfProducts>

</reaction>

<reaction metaid="HisBiosynth" id="HisBiosynth" name="HisBiosynth" reversible="false" fast="false" fbc:lowerFluxBound="FB2N0" fbc:upperFluxBound="FB3N1000">

<listOfReactants>

<speciesReference species="M_ATP__91__d__93____91__c__93__" stoichiometry="3" constant="true"/>

<speciesReference species="M_G6P__91__d__93____91__c__93__" stoichiometry="1" constant="true"/>

<speciesReference species="M_NADP__91__d__93____91__c__93__" stoichiometry="2" constant="true"/>

<speciesReference species="M_NH3__91__d__93____91__c__93__" stoichiometry="3" constant="true"/>

</listOfReactants>

<listOfProducts>

<speciesReference species="M_ADP__91__d__93____91__c__93__" stoichiometry="3" constant="true"/>

<speciesReference species="M_Histidine__91__d__93____91__c__93__" stoichiometry="1" constant="true"/>

<speciesReference species="M_NADPH__91__d__93____91__c__93__" stoichiometry="2" constant="true"/>

<speciesReference species="M_Pi__91__d__93____91__c__93__" stoichiometry="4" constant="true"/>

</listOfProducts>

</reaction>

<reaction metaid="PrephenateDehydratase" id="PrephenateDehydratase" name="PrephenateDehydratase" reversible="true" fast="false" fbc:lowerFluxBound="FB1N1000" fbc:upperFluxBound="FB3N1000">

<listOfReactants>

<speciesReference species="M_Chorismate__91__d__93____91__c__93__" stoichiometry="1" constant="true"/>

</listOfReactants>

<listOfProducts>

<speciesReference species="M_CO2__91__d__93__" stoichiometry="1" constant="true"/>

<speciesReference species="M_H2O__91__d__93__" stoichiometry="1" constant="true"/>

<speciesReference species="M_Phenylpyruvate__91__d__93____91__c__93__" stoichiometry="1" constant="true"/>

</listOfProducts>

</reaction>

<reaction metaid="GlutamineSynthetase" id="GlutamineSynthetase" name="GlutamineSynthetase" reversible="false" fast="false" fbc:lowerFluxBound="FB2N0" fbc:upperFluxBound="FB3N1000">

<listOfReactants>

<speciesReference species="M_ATP__91__d__93__" stoichiometry="1" constant="true"/>

<speciesReference species="M_Glutamate__91__d__93__" stoichiometry="1" constant="true"/>

<speciesReference species="M_NH3__91__d__93__" stoichiometry="1" constant="true"/>

</listOfReactants>

<listOfProducts>

<speciesReference species="M_ADP__91__d__93__" stoichiometry="1" constant="true"/>

<speciesReference species="M_Glutamine__91__d__93__" stoichiometry="1" constant="true"/>

<speciesReference species="M_Pi__91__d__93__" stoichiometry="1" constant="true"/>

</listOfProducts>

</reaction>

<reaction metaid="GOT" id="GOT" name="GOT" reversible="true" fast="false" fbc:lowerFluxBound="FB1N1000" fbc:upperFluxBound="FB3N1000">

<listOfReactants>

<speciesReference species="M_Glutamate__91__d__93__" stoichiometry="1" constant="true"/>

<speciesReference species="M_Oxaloacetate__91__d__93__" stoichiometry="1" constant="true"/>

</listOfReactants>

<listOfProducts>

<speciesReference species="M_Aspartate__91__d__93__" stoichiometry="1" constant="true"/>

<speciesReference species="M_Oxoglutarate__91__d__93__" stoichiometry="1" constant="true"/>

</listOfProducts>

</reaction>

<reaction metaid="HomoserineAcetlyTransf" id="HomoserineAcetlyTransf" name="HomoserineAcetlyTransf" reversible="true" fast="false" fbc:lowerFluxBound="FB1N1000" fbc:upperFluxBound="FB3N1000">

<listOfReactants>

<speciesReference species="M_AcetylCoA__91__d__93____91__c__93__" stoichiometry="1" constant="true"/>

<speciesReference species="M_Homoserine__91__d__93____91__c__93__" stoichiometry="1" constant="true"/>

</listOfReactants>

<listOfProducts>

<speciesReference species="M_AcetylHomoserine__91__d__93____91__c__93__" stoichiometry="1" constant="true"/>

<speciesReference species="M_CoenzymeA__91__d__93____91__c__93__" stoichiometry="1" constant="true"/>

</listOfProducts>

</reaction>

<reaction metaid="OAcetlyHomoserineSulfhydrylase" id="OAcetlyHomoserineSulfhydrylase" name="OAcetlyHomoserineSulfhydrylase" reversible="true" fast="false" fbc:lowerFluxBound="FB1N1000" fbc:upperFluxBound="FB3N1000">

<listOfReactants>

<speciesReference species="M_AcetylHomoserine__91__d__93____91__c__93__" stoichiometry="1" constant="true"/>

<speciesReference species="M_H2S__91__d__93____91__c__93__" stoichiometry="1" constant="true"/>

</listOfReactants>

<listOfProducts>

<speciesReference species="M_Acetate__91__d__93____91__c__93__" stoichiometry="1" constant="true"/>

<speciesReference species="M_Homocysteine__91__d__93____91__c__93__" stoichiometry="1" constant="true"/>

</listOfProducts>

</reaction>

<reaction metaid="Prot32" id="Prot32" name="Prot32" reversible="false" fast="false" fbc:lowerFluxBound="FB2N0" fbc:upperFluxBound="FB3N1000">

<listOfReactants>

<speciesReference species="M_Cysteine__91__d__93____91__c__93__" stoichiometry="1" constant="true"/>

<speciesReference species="M_H2O__91__d__93__" stoichiometry="1" constant="true"/>

</listOfReactants>

<listOfProducts>

<speciesReference species="M_H2S__91__d__93__" stoichiometry="1" constant="true"/>

<speciesReference species="M_NH3__91__d__93____91__c__93__" stoichiometry="1" constant="true"/>

<speciesReference species="M_Pyruvate__91__d__93____91__c__93__" stoichiometry="1" constant="true"/>

</listOfProducts>

</reaction>

<reaction metaid="Prot33" id="Prot33" name="Prot33" reversible="true" fast="false" fbc:lowerFluxBound="FB1N1000" fbc:upperFluxBound="FB3N1000">

<listOfReactants>

<speciesReference species="M_2Oxobutanoate__91__d__93____91__c__93__" stoichiometry="1" constant="true"/>

<speciesReference species="M_H2S__91__d__93__" stoichiometry="1" constant="true"/>

<speciesReference species="M_NH3__91__d__93____91__c__93__" stoichiometry="1" constant="true"/>

</listOfReactants>

<listOfProducts>

<speciesReference species="M_H2O__91__d__93__" stoichiometry="1" constant="true"/>

<speciesReference species="M_Homocysteine__91__d__93____91__c__93__" stoichiometry="1" constant="true"/>

</listOfProducts>

</reaction>

<reaction metaid="BHMT" id="BHMT" name="BHMT" reversible="false" fast="false" fbc:lowerFluxBound="FB2N0" fbc:upperFluxBound="FB3N1000">

<listOfReactants>

<speciesReference species="M_CH3THF__91__d__93__" stoichiometry="1" constant="true"/>

<speciesReference species="M_Homocysteine__91__d__93__" stoichiometry="1" constant="true"/>

</listOfReactants>

<listOfProducts>

<speciesReference species="M_Methionine__91__d__93__" stoichiometry="1" constant="true"/>

<speciesReference species="M_THF__91__d__93__" stoichiometry="1" constant="true"/>

</listOfProducts>

</reaction>

<reaction metaid="Glutathione_reductase" id="Glutathione_reductase" name="Glutathione_reductase" reversible="false" fast="false" fbc:lowerFluxBound="FB2N0" fbc:upperFluxBound="FB3N1000">

<listOfReactants>

<speciesReference species="M_GSSG__91__d__93____91__c__93__" stoichiometry="1" constant="true"/>

<speciesReference species="M_NADPH__91__d__93____91__c__93__" stoichiometry="1" constant="true"/>

</listOfReactants>

<listOfProducts>

<speciesReference species="M_GSH__91__d__93____91__c__93__" stoichiometry="2" constant="true"/>

<speciesReference species="M_NADP__91__d__93____91__c__93__" stoichiometry="1" constant="true"/>

</listOfProducts>

</reaction>

<reaction metaid="Glutithione_Peroxidase" id="Glutithione_Peroxidase" name="Glutithione_Peroxidase" reversible="false" fast="false" fbc:lowerFluxBound="FB2N0" fbc:upperFluxBound="FB3N1000">

<listOfReactants>

<speciesReference species="M_GSH__91__d__93____91__c__93__" stoichiometry="2" constant="true"/>

<speciesReference species="M_H2O2__91__d__93____91__c__93__" stoichiometry="1" constant="true"/>

</listOfReactants>

<listOfProducts>

<speciesReference species="M_GSSG__91__d__93____91__c__93__" stoichiometry="1" constant="true"/>

<speciesReference species="M_H2O__91__d__93__" stoichiometry="1" constant="true"/>

</listOfProducts>

</reaction>

<reaction metaid="ATPS" id="ATPS" name="ATPS" reversible="false" fast="false" fbc:lowerFluxBound="FB2N0" fbc:upperFluxBound="FB3N1000">

<listOfReactants>

<speciesReference species="M_ATP__91__d__93____91__c__93__" stoichiometry="1" constant="true"/>

<speciesReference species="M_Sulfate__91__d__93____91__c__93__" stoichiometry="1" constant="true"/>

</listOfReactants>

<listOfProducts>

<speciesReference species="M_APS__91__d__93____91__c__93__" stoichiometry="1" constant="true"/>

<speciesReference species="M_PPi__91__d__93____91__c__93__" stoichiometry="1" constant="true"/>

</listOfProducts>

</reaction>

<reaction metaid="APR" id="APR" name="APR" reversible="false" fast="false" fbc:lowerFluxBound="FB2N0" fbc:upperFluxBound="FB3N1000">

<listOfReactants>

<speciesReference species="M_APS__91__d__93____91__c__93__" stoichiometry="1" constant="true"/>

</listOfReactants>

<listOfProducts>

<speciesReference species="M_AMP__91__d__93____91__c__93__" stoichiometry="1" constant="true"/>

<speciesReference species="M_Sulfide__91__d__93____91__c__93__" stoichiometry="1" constant="true"/>

</listOfProducts>

</reaction>

<reaction metaid="SiR" id="SiR" name="SiR" reversible="false" fast="false" fbc:lowerFluxBound="FB2N0" fbc:upperFluxBound="FB3N1000">

<listOfReactants>

<speciesReference species="M_Sulfide__91__d__93____91__c__93__" stoichiometry="1" constant="true"/>

</listOfReactants>

<listOfProducts>

<speciesReference species="M_H2O__91__d__93__" stoichiometry="3" constant="true"/>

<speciesReference species="M_H2S__91__d__93____91__c__93__" stoichiometry="1" constant="true"/>

</listOfProducts>

</reaction>

<reaction metaid="SERAT" id="SERAT" name="SERAT" reversible="false" fast="false" fbc:lowerFluxBound="FB2N0" fbc:upperFluxBound="FB3N1000">

<listOfReactants>

<speciesReference species="M_AcetylCoA__91__d__93____91__c__93__" stoichiometry="1" constant="true"/>

<speciesReference species="M_Serine__91__d__93____91__c__93__" stoichiometry="1" constant="true"/>

</listOfReactants>

<listOfProducts>

<speciesReference species="M_CoenzymeA__91__d__93____91__c__93__" stoichiometry="1" constant="true"/>

<speciesReference species="M_OAS__91__d__93____91__c__93__" stoichiometry="1" constant="true"/>

</listOfProducts>

</reaction>

<reaction metaid="serat_mit" id="serat_mit" name="serat_mit" reversible="false" fast="false" fbc:lowerFluxBound="FB2N0" fbc:upperFluxBound="FB3N1000">

<listOfReactants>

<speciesReference species="M_AcetylCoA__91__d__93____91__m__93__" stoichiometry="1" constant="true"/>

<speciesReference species="M_Serine__91__d__93____91__m__93__" stoichiometry="1" constant="true"/>

</listOfReactants>

<listOfProducts>

<speciesReference species="M_CoenzymeA__91__d__93____91__m__93__" stoichiometry="1" constant="true"/>

<speciesReference species="M_OAS__91__d__93____91__m__93__" stoichiometry="1" constant="true"/>

</listOfProducts>

</reaction>

<reaction metaid="serate_cyt" id="serate_cyt" name="serate_cyt" reversible="false" fast="false" fbc:lowerFluxBound="FB2N0" fbc:upperFluxBound="FB3N1000">

<listOfReactants>

<speciesReference species="M_AcetylCoA__91__d__93__" stoichiometry="1" constant="true"/>

<speciesReference species="M_Serine__91__d__93__" stoichiometry="1" constant="true"/>

</listOfReactants>

<listOfProducts>

<speciesReference species="M_CoenzymeA__91__d__93__" stoichiometry="1" constant="true"/>

<speciesReference species="M_OAS__91__d__93__" stoichiometry="1" constant="true"/>

</listOfProducts>

</reaction>

<reaction metaid="OASTL" id="OASTL" name="OASTL" reversible="false" fast="false" fbc:lowerFluxBound="FB2N0" fbc:upperFluxBound="FB3N1000">

<listOfReactants>

<speciesReference species="M_H2S__91__d__93____91__c__93__" stoichiometry="1" constant="true"/>

<speciesReference species="M_OAS__91__d__93____91__c__93__" stoichiometry="1" constant="true"/>

</listOfReactants>

<listOfProducts>

<speciesReference species="M_Acetate__91__d__93____91__c__93__" stoichiometry="1" constant="true"/>

<speciesReference species="M_Cysteine__91__d__93____91__c__93__" stoichiometry="1" constant="true"/>

</listOfProducts>

</reaction>

<reaction metaid="OASTL_mit" id="OASTL_mit" name="OASTL_mit" reversible="false" fast="false" fbc:lowerFluxBound="FB2N0" fbc:upperFluxBound="FB3N1000">

<listOfReactants>

<speciesReference species="M_H2S__91__d__93____91__m__93__" stoichiometry="1" constant="true"/>

<speciesReference species="M_OAS__91__d__93____91__m__93__" stoichiometry="1" constant="true"/>

</listOfReactants>

<listOfProducts>

<speciesReference species="M_Acetate__91__d__93____91__m__93__" stoichiometry="1" constant="true"/>

<speciesReference species="M_Cysteine__91__d__93____91__m__93__" stoichiometry="1" constant="true"/>

</listOfProducts>

</reaction>

<reaction metaid="OASTL_cyt" id="OASTL_cyt" name="OASTL_cyt" reversible="false" fast="false" fbc:lowerFluxBound="FB2N0" fbc:upperFluxBound="FB3N1000">

<listOfReactants>

<speciesReference species="M_H2S__91__d__93__" stoichiometry="1" constant="true"/>

<speciesReference species="M_OAS__91__d__93__" stoichiometry="1" constant="true"/>

</listOfReactants>

<listOfProducts>

<speciesReference species="M_Acetate__91__d__93__" stoichiometry="1" constant="true"/>

<speciesReference species="M_Cysteine__91__d__93__" stoichiometry="1" constant="true"/>

</listOfProducts>

</reaction>

<reaction metaid="gECS_GSHS" id="gECS_GSHS" name="gECS_GSHS" reversible="false" fast="false" fbc:lowerFluxBound="FB2N0" fbc:upperFluxBound="FB3N1000">

<listOfReactants>

<speciesReference species="M_ATP__91__d__93____91__c__93__" stoichiometry="2" constant="true"/>

<speciesReference species="M_Cysteine__91__d__93____91__c__93__" stoichiometry="1" constant="true"/>

<speciesReference species="M_Glutamate__91__d__93____91__c__93__" stoichiometry="1" constant="true"/>

<speciesReference species="M_Glycine__91__d__93____91__c__93__" stoichiometry="1" constant="true"/>

</listOfReactants>

<listOfProducts>

<speciesReference species="M_ADP__91__d__93____91__c__93__" stoichiometry="2" constant="true"/>

<speciesReference species="M_GSH__91__d__93____91__c__93__" stoichiometry="1" constant="true"/>

<speciesReference species="M_Pi__91__d__93____91__c__93__" stoichiometry="2" constant="true"/>

</listOfProducts>

</reaction>

<reaction metaid="Peroxide_r" id="Peroxide_r" name="Peroxide_r" reversible="false" fast="false" fbc:lowerFluxBound="FB2N0" fbc:upperFluxBound="FB3N1000">

<listOfReactants>

<speciesReference species="M_H2O2__91__d__93____91__c__93__" stoichiometry="1" constant="true"/>

</listOfReactants>

<listOfProducts>

<speciesReference species="M_H2O__91__d__93__" stoichiometry="1" constant="true"/>

<speciesReference species="M_O2__91__d__93__" stoichiometry="0.5" constant="true"/>

</listOfProducts>

</reaction>

<reaction metaid="Cystathionine_gamma_lyase" id="Cystathionine_gamma_lyase" name="Cystathionine_gamma_lyase" reversible="false" fast="false" fbc:lowerFluxBound="FB2N0" fbc:upperFluxBound="FB3N1000">

<listOfReactants>

<speciesReference species="M_Cysteine__91__d__93____91__c__93__" stoichiometry="1" constant="true"/>

<speciesReference species="M_Phosphohomoserine__91__d__93____91__c__93__" stoichiometry="1" constant="true"/>

</listOfReactants>

<listOfProducts>

<speciesReference species="M_Cystathionine__91__d__93____91__c__93__" stoichiometry="1" constant="true"/>

</listOfProducts>

</reaction>

<reaction metaid="Cystathionine_beta_lyase" id="Cystathionine_beta_lyase" name="Cystathionine_beta_lyase" reversible="false" fast="false" fbc:lowerFluxBound="FB2N0" fbc:upperFluxBound="FB3N1000">

<listOfReactants>

<speciesReference species="M_Cystathionine__91__d__93____91__c__93__" stoichiometry="1" constant="true"/>

<speciesReference species="M_H2O__91__d__93__" stoichiometry="1" constant="true"/>

</listOfReactants>

<listOfProducts>

<speciesReference species="M_Homocysteine__91__d__93____91__c__93__" stoichiometry="1" constant="true"/>

<speciesReference species="M_NH3__91__d__93____91__c__93__" stoichiometry="1" constant="true"/>

<speciesReference species="M_Pyruvate__91__d__93____91__c__93__" stoichiometry="1" constant="true"/>

</listOfProducts>

</reaction>

<reaction metaid="FTL" id="FTL" name="FTL" reversible="true" fast="false" fbc:lowerFluxBound="FB1N1000" fbc:upperFluxBound="FB3N1000">

<listOfReactants>

<speciesReference species="M_10_FormylTHF__91__d__93__" stoichiometry="1" constant="true"/>

<speciesReference species="M_ADP__91__d__93__" stoichiometry="1" constant="true"/>

<speciesReference species="M_Pi__91__d__93__" stoichiometry="1" constant="true"/>

</listOfReactants>

<listOfProducts>

<speciesReference species="M_ATP__91__d__93__" stoichiometry="1" constant="true"/>

<speciesReference species="M_Formate__91__d__93__" stoichiometry="1" constant="true"/>

<speciesReference species="M_THF__91__d__93__" stoichiometry="1" constant="true"/>

</listOfProducts>

</reaction>

<reaction metaid="MTHFD1" id="MTHFD1" name="MTHFD1" reversible="true" fast="false" fbc:lowerFluxBound="FB1N1000" fbc:upperFluxBound="FB3N1000">

<listOfReactants>

<speciesReference species="M_H2O__91__d__93__" stoichiometry="1" constant="true"/>

<speciesReference species="M_MethenylTHF__91__d__93__" stoichiometry="1" constant="true"/>

</listOfReactants>

<listOfProducts>

<speciesReference species="M_10_FormylTHF__91__d__93__" stoichiometry="1" constant="true"/>

</listOfProducts>

</reaction>

<reaction metaid="MTHFD2" id="MTHFD2" name="MTHFD2" reversible="true" fast="false" fbc:lowerFluxBound="FB1N1000" fbc:upperFluxBound="FB3N1000">

<listOfReactants>

<speciesReference species="M_CH2THF__91__d__93__" stoichiometry="1" constant="true"/>

<speciesReference species="M_NAD__91__d__93__" stoichiometry="1" constant="true"/>

</listOfReactants>

<listOfProducts>

<speciesReference species="M_MethenylTHF__91__d__93__" stoichiometry="1" constant="true"/>

<speciesReference species="M_NADH__91__d__93__" stoichiometry="1" constant="true"/>

</listOfProducts>

</reaction>

<reaction metaid="MTHFR" id="MTHFR" name="MTHFR" reversible="true" fast="false" fbc:lowerFluxBound="FB1N1000" fbc:upperFluxBound="FB3N1000">

<listOfReactants>

<speciesReference species="M_CH2THF__91__d__93__" stoichiometry="1" constant="true"/>

<speciesReference species="M_NADH__91__d__93__" stoichiometry="1" constant="true"/>

</listOfReactants>

<listOfProducts>

<speciesReference species="M_CH3THF__91__d__93__" stoichiometry="1" constant="true"/>

<speciesReference species="M_NAD__91__d__93__" stoichiometry="1" constant="true"/>

</listOfProducts>

</reaction>

<reaction metaid="GPDHyc" id="GPDHyc" name="GPDHyc" reversible="false" fast="false" fbc:lowerFluxBound="FB2N0" fbc:upperFluxBound="FB3N1000">

<listOfReactants>

<speciesReference species="M_DHAP__91__d__93__" stoichiometry="1" constant="true"/>

<speciesReference species="M_NADPH__91__d__93__" stoichiometry="1" constant="true"/>

</listOfReactants>

<listOfProducts>

<speciesReference species="M_Glycerol3P__91__d__93__" stoichiometry="1" constant="true"/>

<speciesReference species="M_NADP__91__d__93__" stoichiometry="1" constant="true"/>

</listOfProducts>

</reaction>

<reaction metaid="GLycerokinase" id="GLycerokinase" name="GLycerokinase" reversible="true" fast="false" fbc:lowerFluxBound="FB1N1000" fbc:upperFluxBound="FB3N1000">

<listOfReactants>

<speciesReference species="M_ADP__91__d__93__" stoichiometry="1" constant="true"/>

<speciesReference species="M_Glycerol3P__91__d__93__" stoichiometry="1" constant="true"/>

</listOfReactants>

<listOfProducts>

<speciesReference species="M_ATP__91__d__93__" stoichiometry="1" constant="true"/>

<speciesReference species="M_Glycerol__91__d__93__" stoichiometry="1" constant="true"/>

</listOfProducts>

</reaction>

<reaction metaid="SCD1" id="SCD1" name="SCD1" reversible="false" fast="false" fbc:lowerFluxBound="FB2N0" fbc:upperFluxBound="FB3N1000">

<listOfReactants>

<speciesReference species="M_NADP__91__d__93__" stoichiometry="1" constant="true"/>

<speciesReference species="M_Palmitate__91__d__93__" stoichiometry="1" constant="true"/>

</listOfReactants>

<listOfProducts>

<speciesReference species="M_NADPH__91__d__93__" stoichiometry="1" constant="true"/>

<speciesReference species="M_Palmitoleate__91__d__93__" stoichiometry="1" constant="true"/>

</listOfProducts>

</reaction>

<reaction metaid="FAD2" id="FAD2" name="FAD2" reversible="false" fast="false" fbc:lowerFluxBound="FB2N0" fbc:upperFluxBound="FB3N1000">

<listOfReactants>

<speciesReference species="M_NADP__91__d__93__" stoichiometry="1" constant="true"/>

<speciesReference species="M_Oleate__91__d__93__" stoichiometry="1" constant="true"/>

</listOfReactants>

<listOfProducts>

<speciesReference species="M_Linoleate__91__d__93__" stoichiometry="1" constant="true"/>

<speciesReference species="M_NADPH__91__d__93__" stoichiometry="1" constant="true"/>

</listOfProducts>

</reaction>

<reaction metaid="FAD3" id="FAD3" name="FAD3" reversible="false" fast="false" fbc:lowerFluxBound="FB2N0" fbc:upperFluxBound="FB3N1000">

<listOfReactants>

<speciesReference species="M_Linoleate__91__d__93__" stoichiometry="1" constant="true"/>

<speciesReference species="M_NADP__91__d__93__" stoichiometry="1" constant="true"/>

</listOfReactants>

<listOfProducts>

<speciesReference species="M_NADPH__91__d__93__" stoichiometry="1" constant="true"/>

<speciesReference species="M_g_Linolenate__91__d__93__" stoichiometry="1" constant="true"/>

</listOfProducts>

</reaction>

<reaction metaid="AcetylCoACarboxylase" id="AcetylCoACarboxylase" name="AcetylCoACarboxylase" reversible="false" fast="false" fbc:lowerFluxBound="FB2N0" fbc:upperFluxBound="FB3N1000">

<listOfReactants>

<speciesReference species="M_ATP__91__d__93__" stoichiometry="1" constant="true"/>

<speciesReference species="M_AcetylCoA__91__d__93__" stoichiometry="1" constant="true"/>

<speciesReference species="M_CO2__91__d__93__" stoichiometry="1" constant="true"/>

</listOfReactants>

<listOfProducts>

<speciesReference species="M_ADP__91__d__93__" stoichiometry="1" constant="true"/>

<speciesReference species="M_MalonylCoA__91__d__93__" stoichiometry="1" constant="true"/>

<speciesReference species="M_Pi__91__d__93__" stoichiometry="1" constant="true"/>

</listOfProducts>

</reaction>

<reaction metaid="PalmitateBiosynth" id="PalmitateBiosynth" name="PalmitateBiosynth" reversible="false" fast="false" fbc:lowerFluxBound="FB2N0" fbc:upperFluxBound="FB3N1000">

<listOfReactants>

<speciesReference species="M_AcetylCoA__91__d__93__" stoichiometry="1" constant="true"/>

<speciesReference species="M_MalonylCoA__91__d__93__" stoichiometry="7" constant="true"/>

<speciesReference species="M_NADPH__91__d__93__" stoichiometry="14" constant="true"/>

</listOfReactants>

<listOfProducts>

<speciesReference species="M_CO2__91__d__93__" stoichiometry="7" constant="true"/>

<speciesReference species="M_CoenzymeA__91__d__93__" stoichiometry="8" constant="true"/>

<speciesReference species="M_H2O__91__d__93__" stoichiometry="6" constant="true"/>

<speciesReference species="M_NADP__91__d__93__" stoichiometry="14" constant="true"/>

<speciesReference species="M_Palmitate__91__d__93__" stoichiometry="1" constant="true"/>

</listOfProducts>

</reaction>

<reaction metaid="StearoylCoadesaturase" id="StearoylCoadesaturase" name="StearoylCoadesaturase" reversible="false" fast="false" fbc:lowerFluxBound="FB2N0" fbc:upperFluxBound="FB3N1000">

<listOfReactants>

<speciesReference species="M_NADP__91__d__93__" stoichiometry="1" constant="true"/>

<speciesReference species="M_Stearate__91__d__93__" stoichiometry="1" constant="true"/>

</listOfReactants>

<listOfProducts>

<speciesReference species="M_NADPH__91__d__93__" stoichiometry="1" constant="true"/>

<speciesReference species="M_Oleate__91__d__93__" stoichiometry="1" constant="true"/>

</listOfProducts>

</reaction>

<reaction metaid="Elongase" id="Elongase" name="Elongase" reversible="false" fast="false" fbc:lowerFluxBound="FB2N0" fbc:upperFluxBound="FB3N1000">

<listOfReactants>

<speciesReference species="M_MalonylCoA__91__d__93__" stoichiometry="1" constant="true"/>

<speciesReference species="M_NADPH__91__d__93__" stoichiometry="2" constant="true"/>

<speciesReference species="M_Palmitate__91__d__93__" stoichiometry="1" constant="true"/>

</listOfReactants>

<listOfProducts>

<speciesReference species="M_CO2__91__d__93__" stoichiometry="1" constant="true"/>

<speciesReference species="M_CoenzymeA__91__d__93__" stoichiometry="1" constant="true"/>

<speciesReference species="M_H2O__91__d__93__" stoichiometry="1" constant="true"/>

<speciesReference species="M_NADP__91__d__93__" stoichiometry="2" constant="true"/>

<speciesReference species="M_Stearate__91__d__93__" stoichiometry="1" constant="true"/>

</listOfProducts>

</reaction>

<reaction metaid="MethCycle" id="MethCycle" name="MethCycle" reversible="true" fast="false" fbc:lowerFluxBound="FB1N1000" fbc:upperFluxBound="FB3N1000">

<listOfReactants>

<speciesReference species="M_Methionine__91__d__93__" stoichiometry="1" constant="true"/>

<speciesReference species="M_S_Ad_Homocysteine__91__d__93__" stoichiometry="1" constant="true"/>

</listOfReactants>

<listOfProducts>

<speciesReference species="M_Homocysteine__91__d__93__" stoichiometry="1" constant="true"/>

<speciesReference species="M_S_Ade_Methionine__91__d__93__" stoichiometry="1" constant="true"/>

</listOfProducts>

</reaction>

<reaction metaid="BrassicasterolBiosynth" id="BrassicasterolBiosynth" name="BrassicasterolBiosynth" reversible="false" fast="false" fbc:lowerFluxBound="FB2N0" fbc:upperFluxBound="FB3N1000">

<listOfReactants>

<speciesReference species="M_G3P__91__d__93__" stoichiometry="0.6429" constant="true"/>

<speciesReference species="M_NAD__91__d__93__" stoichiometry="0.4643" constant="true"/>

<speciesReference species="M_O2__91__d__93__" stoichiometry="0.357" constant="true"/>

<speciesReference species="M_S_Ade_Methionine__91__d__93__" stoichiometry="0.0357" constant="true"/>

</listOfReactants>

<listOfProducts>

<speciesReference species="M_Brassicasterol__91__d__93__" stoichiometry="1" constant="true"/>

<speciesReference species="M_CO2__91__d__93__" stoichiometry="0.9643" constant="true"/>

<speciesReference species="M_H2O__91__d__93__" stoichiometry="0.0357" constant="true"/>

<speciesReference species="M_NADH__91__d__93__" stoichiometry="0.4643" constant="true"/>

<speciesReference species="M_Pi__91__d__93__" stoichiometry="0.6429" constant="true"/>

<speciesReference species="M_S_Ad_Homocysteine__91__d__93__" stoichiometry="0.0357" constant="true"/>

</listOfProducts>

</reaction>

<reaction metaid="SaturatedFatsBiosynth" id="SaturatedFatsBiosynth" name="SaturatedFatsBiosynth" reversible="false" fast="false" fbc:lowerFluxBound="FB2N0" fbc:upperFluxBound="FB3N1000">

<listOfReactants>

<speciesReference species="M_Palmitate__91__d__93__" stoichiometry="0.0143" constant="true"/>

<speciesReference species="M_Stearate__91__d__93__" stoichiometry="0.0428" constant="true"/>

</listOfReactants>

<listOfProducts>

<speciesReference species="M_H2O__91__d__93__" stoichiometry="0.0143" constant="true"/>

<speciesReference species="M_Saturated_Fats__91__d__93__" stoichiometry="1" constant="true"/>

</listOfProducts>

</reaction>

<reaction metaid="UnsaturatedFats_Biosynth" id="UnsaturatedFats_Biosynth" name="UnsaturatedFats_Biosynth" reversible="false" fast="false" fbc:lowerFluxBound="FB2N0" fbc:upperFluxBound="FB3N1000">

<listOfReactants>

<speciesReference species="M_Oleate__91__d__93__" stoichiometry="0.0385" constant="true"/>

<speciesReference species="M_Palmitoleate__91__d__93__" stoichiometry="0.0192" constant="true"/>

</listOfReactants>

<listOfProducts>

<speciesReference species="M_H2O__91__d__93__" stoichiometry="0.0192" constant="true"/>

<speciesReference species="M_Unsaturated_Fats__91__d__93__" stoichiometry="1" constant="true"/>

</listOfProducts>

</reaction>

<reaction metaid="PoliUnsaturatedFats_Biosynth" id="PoliUnsaturatedFats_Biosynth" name="PoliUnsaturatedFats_Biosynth" reversible="false" fast="false" fbc:lowerFluxBound="FB2N0" fbc:upperFluxBound="FB3N1000">

<listOfReactants>

<speciesReference species="M_Linoleate__91__d__93__" stoichiometry="0.0138" constant="true"/>

<speciesReference species="M_g_Linolenate__91__d__93__" stoichiometry="0.0416" constant="true"/>

</listOfReactants>

<listOfProducts>

<speciesReference species="M_H2O__91__d__93__" stoichiometry="0.0138" constant="true"/>

<speciesReference species="M_PolyunsaturatedFats__91__d__93__" stoichiometry="1" constant="true"/>

</listOfProducts>

</reaction>

<reaction metaid="Prot_Total" id="Prot_Total" name="Prot_Total" reversible="false" fast="false" fbc:lowerFluxBound="FB2N0" fbc:upperFluxBound="FB3N1000">

<listOfReactants>

<speciesReference species="M_Alanine__91__d__93__" stoichiometry="0.0126" constant="true"/>

<speciesReference species="M_Arginine__91__d__93__" stoichiometry="0.00802" constant="true"/>

<speciesReference species="M_Asparagine__91__d__93__" stoichiometry="0.00734" constant="true"/>

<speciesReference species="M_Aspartate__91__d__93__" stoichiometry="0.0214" constant="true"/>

<speciesReference species="M_Cystine__91__d__93__" stoichiometry="0.0014" constant="true"/>

<speciesReference species="M_Glutamate__91__d__93__" stoichiometry="0.0251" constant="true"/>

<speciesReference species="M_Glutamine__91__d__93__" stoichiometry="0.0259" constant="true"/>

<speciesReference species="M_Glycine__91__d__93__" stoichiometry="0.0157" constant="true"/>

<speciesReference species="M_Histidine__91__d__93__" stoichiometry="0.00287" constant="true"/>

<speciesReference species="M_Isoleucine__91__d__93__" stoichiometry="0.0128" constant="true"/>

<speciesReference species="M_Leucine__91__d__93__" stoichiometry="0.0119" constant="true"/>

<speciesReference species="M_Lysine__91__d__93__" stoichiometry="0.0114" constant="true"/>

<speciesReference species="M_Methionine__91__d__93__" stoichiometry="0.0026" constant="true"/>

<speciesReference species="M_Phenylalanine__91__d__93__" stoichiometry="0.00618" constant="true"/>

<speciesReference species="M_Proline__91__d__93__" stoichiometry="0.00826" constant="true"/>

<speciesReference species="M_Serine__91__d__93__" stoichiometry="0.00744" constant="true"/>

<speciesReference species="M_Threonine__91__d__93__" stoichiometry="0.00989" constant="true"/>

<speciesReference species="M_Tryptophan__91__d__93__" stoichiometry="0.00102" constant="true"/>

<speciesReference species="M_Tyrosine__91__d__93__" stoichiometry="0.00324" constant="true"/>

<speciesReference species="M_Valine__91__d__93__" stoichiometry="0.0119" constant="true"/>

</listOfReactants>

<listOfProducts>

<speciesReference species="M_H2O__91__d__93__" stoichiometry="1" constant="true"/>

<speciesReference species="M_Prot__91__d__93__" stoichiometry="1" constant="true"/>

</listOfProducts>

</reaction>

<reaction metaid="Lipids" id="Lipids" name="Lipids" reversible="false" fast="false" fbc:lowerFluxBound="FB2N0" fbc:upperFluxBound="FB3N1000">

<listOfReactants>

<speciesReference species="M_Glycerol__91__d__93__" stoichiometry="0.167" constant="true"/>

<speciesReference species="M_PolyunsaturatedFats__91__d__93__" stoichiometry="0.39" constant="true"/>

<speciesReference species="M_Saturated_Fats__91__d__93__" stoichiometry="0.0895" constant="true"/>

<speciesReference species="M_Unsaturated_Fats__91__d__93__" stoichiometry="0.0226" constant="true"/>

</listOfReactants>

<listOfProducts>

<speciesReference species="M_H2O__91__d__93__" stoichiometry="0.5013" constant="true"/>

<speciesReference species="M_triglycerids__91__d__93__" stoichiometry="1" constant="true"/>

</listOfProducts>

</reaction>

<reaction metaid="Biomass" id="Biomass" name="Biomass" reversible="false" fast="false" fbc:lowerFluxBound="FB2N0" fbc:upperFluxBound="FB3N1000">

<listOfReactants>

<speciesReference species="M_Brassicasterol__91__d__93__" stoichiometry="0.01678" constant="true"/>

<speciesReference species="M_Prot__91__d__93__" stoichiometry="0.3518" constant="true"/>

<speciesReference species="M_fibers__91__d__93__" stoichiometry="0.3089" constant="true"/>

<speciesReference species="M_sugars__91__d__93__" stoichiometry="0.2756" constant="true"/>

<speciesReference species="M_triglycerids__91__d__93__" stoichiometry="0.0469" constant="true"/>

</listOfReactants>

<listOfProducts>

<speciesReference species="M_Biomass__91__d__93__" stoichiometry="1" constant="true"/>

</listOfProducts>

</reaction>

<reaction metaid="ATPase_cyt" id="ATPase_cyt" name="ATPase_cyt" reversible="false" fast="false" fbc:lowerFluxBound="FB2N0" fbc:upperFluxBound="FB3N1000">

<listOfReactants>

<speciesReference species="M_ATP__91__d__93__" stoichiometry="1" constant="true"/>

<speciesReference species="M_H2O__91__d__93__" stoichiometry="1" constant="true"/>

</listOfReactants>

<listOfProducts>

<speciesReference species="M_ADP__91__d__93__" stoichiometry="1" constant="true"/>

<speciesReference species="M_Pi__91__d__93__" stoichiometry="1" constant="true"/>

</listOfProducts>

</reaction>

<reaction metaid="ATPase_cl" id="ATPase_cl" name="ATPase_cl" reversible="false" fast="false" fbc:lowerFluxBound="FB2N0" fbc:upperFluxBound="FB3N1000">

<listOfReactants>

<speciesReference species="M_ATP__91__d__93____91__c__93__" stoichiometry="1" constant="true"/>

<speciesReference species="M_H2O__91__d__93__" stoichiometry="1" constant="true"/>

</listOfReactants>

<listOfProducts>

<speciesReference species="M_ADP__91__d__93____91__c__93__" stoichiometry="1" constant="true"/>

<speciesReference species="M_Pi__91__d__93____91__c__93__" stoichiometry="1" constant="true"/>

</listOfProducts>

</reaction>

<reaction metaid="ATPase_mit" id="ATPase_mit" name="ATPase_mit" reversible="false" fast="false" fbc:lowerFluxBound="FB2N0" fbc:upperFluxBound="FB3N1000">

<listOfReactants>

<speciesReference species="M_ATP__91__d__93____91__m__93__" stoichiometry="1" constant="true"/>

<speciesReference species="M_H2O__91__d__93__" stoichiometry="1" constant="true"/>

</listOfReactants>

<listOfProducts>

<speciesReference species="M_ADP__91__d__93____91__m__93__" stoichiometry="1" constant="true"/>

<speciesReference species="M_Pi__91__d__93____91__m__93__" stoichiometry="1" constant="true"/>

</listOfProducts>

</reaction>

<reaction metaid="NADPHoxidase_cyt" id="NADPHoxidase_cyt" name="NADPHoxidase_cyt" reversible="false" fast="false" fbc:lowerFluxBound="FB2N0" fbc:upperFluxBound="FB3N1000">

<listOfReactants>

<speciesReference species="M_NADPH__91__d__93__" stoichiometry="1" constant="true"/>

<speciesReference species="M_O2__91__d__93__" stoichiometry="1" constant="true"/>

</listOfReactants>

<listOfProducts>

<speciesReference species="M_H2O__91__d__93__" stoichiometry="1" constant="true"/>

<speciesReference species="M_NADP__91__d__93__" stoichiometry="1" constant="true"/>

</listOfProducts>

</reaction>

<reaction metaid="NADPHoxidase_cl" id="NADPHoxidase_cl" name="NADPHoxidase_cl" reversible="false" fast="false" fbc:lowerFluxBound="FB2N0" fbc:upperFluxBound="FB3N1000">

<listOfReactants>

<speciesReference species="M_NADPH__91__d__93____91__c__93__" stoichiometry="1" constant="true"/>

<speciesReference species="M_O2__91__d__93__" stoichiometry="1" constant="true"/>

</listOfReactants>

<listOfProducts>

<speciesReference species="M_H2O__91__d__93__" stoichiometry="1" constant="true"/>

<speciesReference species="M_NADP__91__d__93____91__c__93__" stoichiometry="1" constant="true"/>

</listOfProducts>

</reaction>

<reaction metaid="NADPHoxidase_mit" id="NADPHoxidase_mit" name="NADPHoxidase_mit" reversible="false" fast="false" fbc:lowerFluxBound="FB2N0" fbc:upperFluxBound="FB3N1000">

<listOfReactants>

<speciesReference species="M_NADPH__91__d__93____91__m__93__" stoichiometry="1" constant="true"/>

<speciesReference species="M_O2__91__d__93__" stoichiometry="1" constant="true"/>

</listOfReactants>

<listOfProducts>

<speciesReference species="M_H2O__91__d__93__" stoichiometry="1" constant="true"/>

<speciesReference species="M_NADP__91__d__93____91__m__93__" stoichiometry="1" constant="true"/>

</listOfProducts>

</reaction>

<reaction metaid="Ex_CO2_night" id="Ex_CO2_night" name="Ex_CO2_night" reversible="true" fast="false" fbc:lowerFluxBound="FB1N1000" fbc:upperFluxBound="FB3N1000">

<listOfReactants>

<speciesReference species="M_CO2__91__n__93__" stoichiometry="1" constant="true"/>

</listOfReactants>

</reaction>

<reaction metaid="Ex_O2_night" id="Ex_O2_night" name="Ex_O2_night" reversible="true" fast="false" fbc:lowerFluxBound="FB1N1000" fbc:upperFluxBound="FB3N1000">

<listOfReactants>

<speciesReference species="M_O2__91__n__93__" stoichiometry="1" constant="true"/>

</listOfReactants>

</reaction>

<reaction metaid="Ex_H2O_night" id="Ex_H2O_night" name="Ex_H2O_night" reversible="true" fast="false" fbc:lowerFluxBound="FB1N1000" fbc:upperFluxBound="FB3N1000">

<listOfReactants>

<speciesReference species="M_H2O__91__n__93__" stoichiometry="1" constant="true"/>

</listOfReactants>

</reaction>

<reaction metaid="Ex_HNO3_night" id="Ex_HNO3_night" name="Ex_HNO3_night" reversible="true" fast="false" fbc:lowerFluxBound="FB1N1000" fbc:upperFluxBound="FB3N1000">

<listOfReactants>

<speciesReference species="M_HNO3__91__n__93__" stoichiometry="1" constant="true"/>

</listOfReactants>

</reaction>

<reaction metaid="Ex_Biomass_night" id="Ex_Biomass_night" name="Ex_Biomass_night" reversible="true" fast="false" fbc:lowerFluxBound="FB1N1000" fbc:upperFluxBound="FB3N1000">

<listOfReactants>

<speciesReference species="M_Biomass__91__n__93__" stoichiometry="1" constant="true"/>

</listOfReactants>

</reaction>

<reaction metaid="Ex_H2S_night" id="Ex_H2S_night" name="Ex_H2S_night" reversible="true" fast="false" fbc:lowerFluxBound="FB1N1000" fbc:upperFluxBound="FB3N1000">

<listOfReactants>

<speciesReference species="M_H2S__91__n__93__" stoichiometry="1" constant="true"/>

</listOfReactants>

</reaction>

<reaction metaid="Ex_Sulfate_night" id="Ex_Sulfate_night" name="Ex_Sulfate_night" reversible="true" fast="false" fbc:lowerFluxBound="FB1N1000" fbc:upperFluxBound="FB3N1000">

<listOfReactants>

<speciesReference species="M_Sulfate__91__n__93__" stoichiometry="1" constant="true"/>

</listOfReactants>

</reaction>

<reaction metaid="Exp_sucrosePhloemDay_night" id="Exp_sucrosePhloemDay_night" name="Exp_sucrosePhloemDay_night" reversible="false" fast="false" fbc:lowerFluxBound="FB2N0" fbc:upperFluxBound="FB3N1000">

<listOfReactants>

<speciesReference species="M_sucrose_exp__91__n__93__" stoichiometry="1" constant="true"/>

</listOfReactants>

</reaction>

<reaction metaid="Exp_AAPhloemDay_night" id="Exp_AAPhloemDay_night" name="Exp_AAPhloemDay_night" reversible="false" fast="false" fbc:lowerFluxBound="FB2N0" fbc:upperFluxBound="FB3N1000">

<listOfReactants>

<speciesReference species="M_AA_exp__91__n__93__" stoichiometry="1" constant="true"/>

</listOfReactants>

</reaction>

<reaction metaid="Asn_DayPhloeamExp_night" id="Asn_DayPhloeamExp_night" name="Asn_DayPhloeamExp_night" reversible="false" fast="false" fbc:lowerFluxBound="FB2N0" fbc:upperFluxBound="FB3N1000">

<listOfReactants>

<speciesReference species="M_Asparagine__91__n__93__" stoichiometry="1" constant="true"/>

</listOfReactants>

<listOfProducts>

<speciesReference species="M_Asparagine_exp__91__n__93__" stoichiometry="1" constant="true"/>

</listOfProducts>

</reaction>

<reaction metaid="Asp_DayPhloemExp_night" id="Asp_DayPhloemExp_night" name="Asp_DayPhloemExp_night" reversible="false" fast="false" fbc:lowerFluxBound="FB2N0" fbc:upperFluxBound="FB3N1000">

<listOfReactants>

<speciesReference species="M_Aspartate__91__n__93__" stoichiometry="1" constant="true"/>

</listOfReactants>

<listOfProducts>

<speciesReference species="M_Aspartate_exp__91__n__93__" stoichiometry="1" constant="true"/>

</listOfProducts>

</reaction>

<reaction metaid="Thr_DayPhloeamExp_night" id="Thr_DayPhloeamExp_night" name="Thr_DayPhloeamExp_night" reversible="false" fast="false" fbc:lowerFluxBound="FB2N0" fbc:upperFluxBound="FB3N1000">

<listOfReactants>

<speciesReference species="M_Threonine__91__n__93__" stoichiometry="1" constant="true"/>

</listOfReactants>

<listOfProducts>

<speciesReference species="M_Threonine_exp__91__n__93__" stoichiometry="1" constant="true"/>

</listOfProducts>

</reaction>

<reaction metaid="Gln_Day_Phloem_Exp_night" id="Gln_Day_Phloem_Exp_night" name="Gln_Day_Phloem_Exp_night" reversible="false" fast="false" fbc:lowerFluxBound="FB2N0" fbc:upperFluxBound="FB3N1000">

<listOfReactants>

<speciesReference species="M_Glutamate__91__n__93__" stoichiometry="1" constant="true"/>

</listOfReactants>

<listOfProducts>

<speciesReference species="M_Glutamate_exp__91__n__93__" stoichiometry="1" constant="true"/>

</listOfProducts>

</reaction>

<reaction metaid="Iso_DayPhloemExp_night" id="Iso_DayPhloemExp_night" name="Iso_DayPhloemExp_night" reversible="false" fast="false" fbc:lowerFluxBound="FB2N0" fbc:upperFluxBound="FB3N1000">

<listOfReactants>

<speciesReference species="M_Isoleucine__91__n__93__" stoichiometry="1" constant="true"/>

</listOfReactants>

<listOfProducts>

<speciesReference species="M_Isoleucine_exp__91__n__93__" stoichiometry="1" constant="true"/>

</listOfProducts>

</reaction>

<reaction metaid="Val_DayPhloemExp_night" id="Val_DayPhloemExp_night" name="Val_DayPhloemExp_night" reversible="false" fast="false" fbc:lowerFluxBound="FB2N0" fbc:upperFluxBound="FB3N1000">

<listOfReactants>

<speciesReference species="M_Valine__91__n__93__" stoichiometry="1" constant="true"/>

</listOfReactants>

<listOfProducts>

<speciesReference species="M_Valine_exp__91__n__93__" stoichiometry="1" constant="true"/>

</listOfProducts>

</reaction>

<reaction metaid="Ala_DayPhloemExp_night" id="Ala_DayPhloemExp_night" name="Ala_DayPhloemExp_night" reversible="false" fast="false" fbc:lowerFluxBound="FB2N0" fbc:upperFluxBound="FB3N1000">

<listOfReactants>

<speciesReference species="M_Alanine__91__n__93__" stoichiometry="1" constant="true"/>

</listOfReactants>

<listOfProducts>

<speciesReference species="M_Alanine_exp__91__n__93__" stoichiometry="1" constant="true"/>

</listOfProducts>

</reaction>

<reaction metaid="Leu_DayPhloemExp_night" id="Leu_DayPhloemExp_night" name="Leu_DayPhloemExp_night" reversible="false" fast="false" fbc:lowerFluxBound="FB2N0" fbc:upperFluxBound="FB3N1000">

<listOfReactants>

<speciesReference species="M_Leucine__91__n__93__" stoichiometry="1" constant="true"/>

</listOfReactants>

<listOfProducts>

<speciesReference species="M_Leucine_exp__91__n__93__" stoichiometry="1" constant="true"/>

</listOfProducts>

</reaction>

<reaction metaid="Ser_DayPhloemExp_night" id="Ser_DayPhloemExp_night" name="Ser_DayPhloemExp_night" reversible="false" fast="false" fbc:lowerFluxBound="FB2N0" fbc:upperFluxBound="FB3N1000">

<listOfReactants>

<speciesReference species="M_Serine__91__n__93__" stoichiometry="1" constant="true"/>

</listOfReactants>

<listOfProducts>

<speciesReference species="M_Serine_exp__91__n__93__" stoichiometry="1" constant="true"/>

</listOfProducts>

</reaction>

<reaction metaid="Gly_Day_PhloemExp_night" id="Gly_Day_PhloemExp_night" name="Gly_Day_PhloemExp_night" reversible="false" fast="false" fbc:lowerFluxBound="FB2N0" fbc:upperFluxBound="FB3N1000">

<listOfReactants>

<speciesReference species="M_Glycine__91__n__93__" stoichiometry="1" constant="true"/>

</listOfReactants>

<listOfProducts>

<speciesReference species="M_Glycine_exp__91__n__93__" stoichiometry="1" constant="true"/>

</listOfProducts>

</reaction>

<reaction metaid="Glu_DayPhloemExp_night" id="Glu_DayPhloemExp_night" name="Glu_DayPhloemExp_night" reversible="false" fast="false" fbc:lowerFluxBound="FB2N0" fbc:upperFluxBound="FB3N1000">

<listOfReactants>

<speciesReference species="M_Glutamine__91__n__93__" stoichiometry="1" constant="true"/>

</listOfReactants>

<listOfProducts>

<speciesReference species="M_Glutamine_exp__91__n__93__" stoichiometry="1" constant="true"/>

</listOfProducts>

</reaction>

<reaction metaid="Arg_DayPhloemExp_night" id="Arg_DayPhloemExp_night" name="Arg_DayPhloemExp_night" reversible="false" fast="false" fbc:lowerFluxBound="FB2N0" fbc:upperFluxBound="FB3N1000">

<listOfReactants>

<speciesReference species="M_Arginine__91__n__93__" stoichiometry="1" constant="true"/>

</listOfReactants>

<listOfProducts>

<speciesReference species="M_Arginine_exp__91__n__93__" stoichiometry="1" constant="true"/>

</listOfProducts>

</reaction>

<reaction metaid="Tyr_DayPhloemExp_night" id="Tyr_DayPhloemExp_night" name="Tyr_DayPhloemExp_night" reversible="false" fast="false" fbc:lowerFluxBound="FB2N0" fbc:upperFluxBound="FB3N1000">

<listOfReactants>

<speciesReference species="M_Tyrosine__91__n__93__" stoichiometry="1" constant="true"/>

</listOfReactants>

<listOfProducts>

<speciesReference species="M_Tyrosine_exp__91__n__93__" stoichiometry="1" constant="true"/>

</listOfProducts>

</reaction>

<reaction metaid="Phe_DayPhloemExp_night" id="Phe_DayPhloemExp_night" name="Phe_DayPhloemExp_night" reversible="false" fast="false" fbc:lowerFluxBound="FB2N0" fbc:upperFluxBound="FB3N1000">

<listOfReactants>

<speciesReference species="M_Phenylalanine__91__n__93__" stoichiometry="1" constant="true"/>

</listOfReactants>

<listOfProducts>

<speciesReference species="M_Phenylalanine_exp__91__n__93__" stoichiometry="1" constant="true"/>

</listOfProducts>

</reaction>

<reaction metaid="Try_DayPhloemExp_night" id="Try_DayPhloemExp_night" name="Try_DayPhloemExp_night" reversible="false" fast="false" fbc:lowerFluxBound="FB2N0" fbc:upperFluxBound="FB3N1000">

<listOfReactants>

<speciesReference species="M_Tryptophan__91__n__93__" stoichiometry="1" constant="true"/>

</listOfReactants>

<listOfProducts>

<speciesReference species="M_Tryptophan_exp__91__n__93__" stoichiometry="1" constant="true"/>

</listOfProducts>

</reaction>

<reaction metaid="Lys_DayPhloemExp_night" id="Lys_DayPhloemExp_night" name="Lys_DayPhloemExp_night" reversible="false" fast="false" fbc:lowerFluxBound="FB2N0" fbc:upperFluxBound="FB3N1000">

<listOfReactants>

<speciesReference species="M_Lysine__91__n__93__" stoichiometry="1" constant="true"/>

</listOfReactants>

<listOfProducts>

<speciesReference species="M_Lysine_exp__91__n__93__" stoichiometry="1" constant="true"/>

</listOfProducts>

</reaction>

<reaction metaid="His_DayPhloemExp_night" id="His_DayPhloemExp_night" name="His_DayPhloemExp_night" reversible="false" fast="false" fbc:lowerFluxBound="FB2N0" fbc:upperFluxBound="FB3N1000">

<listOfReactants>

<speciesReference species="M_Histidine__91__n__93__" stoichiometry="1" constant="true"/>

</listOfReactants>

<listOfProducts>

<speciesReference species="M_Histidine_exp__91__n__93__" stoichiometry="1" constant="true"/>

</listOfProducts>

</reaction>

<reaction metaid="Met_DayPhloemExp_night" id="Met_DayPhloemExp_night" name="Met_DayPhloemExp_night" reversible="false" fast="false" fbc:lowerFluxBound="FB2N0" fbc:upperFluxBound="FB3N1000">

<listOfReactants>

<speciesReference species="M_Methionine__91__n__93__" stoichiometry="1" constant="true"/>

</listOfReactants>

<listOfProducts>

<speciesReference species="M_Methionine_exp__91__n__93__" stoichiometry="1" constant="true"/>

</listOfProducts>

</reaction>

<reaction metaid="Suc_DayPhloem_Exp_night" id="Suc_DayPhloem_Exp_night" name="Suc_DayPhloem_Exp_night" reversible="false" fast="false" fbc:lowerFluxBound="FB2N0" fbc:upperFluxBound="FB3N1000">

<listOfReactants>

<speciesReference species="M_sucrose__91__n__93__" stoichiometry="1" constant="true"/>

</listOfReactants>

<listOfProducts>

<speciesReference species="M_sucrose_exp__91__n__93__" stoichiometry="1" constant="true"/>

</listOfProducts>

</reaction>

<reaction metaid="AminoAcidExport_Day_night" id="AminoAcidExport_Day_night" name="AminoAcidExport_Day_night" reversible="false" fast="false" fbc:lowerFluxBound="FB2N0" fbc:upperFluxBound="FB3N1000">

<listOfReactants>

<speciesReference species="M_Alanine_exp__91__n__93__" stoichiometry="9.7" constant="true"/>

<speciesReference species="M_Arginine_exp__91__n__93__" stoichiometry="1.6" constant="true"/>

<speciesReference species="M_Asparagine_exp__91__n__93__" stoichiometry="10.1" constant="true"/>

<speciesReference species="M_Aspartate_exp__91__n__93__" stoichiometry="9.5" constant="true"/>

<speciesReference species="M_Glutamate_exp__91__n__93__" stoichiometry="11.4" constant="true"/>

<speciesReference species="M_Glutamine_exp__91__n__93__" stoichiometry="33.2" constant="true"/>

<speciesReference species="M_Glycine_exp__91__n__93__" stoichiometry="0.7" constant="true"/>

<speciesReference species="M_Histidine_exp__91__n__93__" stoichiometry="0.4" constant="true"/>

<speciesReference species="M_Isoleucine_exp__91__n__93__" stoichiometry="1.2" constant="true"/>

<speciesReference species="M_Leucine_exp__91__n__93__" stoichiometry="1.2" constant="true"/>

<speciesReference species="M_Lysine_exp__91__n__93__" stoichiometry="1.8" constant="true"/>

<speciesReference species="M_Methionine_exp__91__n__93__" stoichiometry="0.7" constant="true"/>

<speciesReference species="M_Phenylalanine_exp__91__n__93__" stoichiometry="1.1" constant="true"/>

<speciesReference species="M_Serine_exp__91__n__93__" stoichiometry="7.7" constant="true"/>

<speciesReference species="M_Threonine_exp__91__n__93__" stoichiometry="5.6" constant="true"/>

<speciesReference species="M_Tryptophan_exp__91__n__93__" stoichiometry="0.9" constant="true"/>

<speciesReference species="M_Tyrosine_exp__91__n__93__" stoichiometry="0.6" constant="true"/>

<speciesReference species="M_Valine_exp__91__n__93__" stoichiometry="2.4" constant="true"/>

</listOfReactants>

<listOfProducts>

<speciesReference species="M_AA_exp__91__n__93__" stoichiometry="1" constant="true"/>

</listOfProducts>

</reaction>

<reaction metaid="Thr_Cl_export_night" id="Thr_Cl_export_night" name="Thr_Cl_export_night" reversible="true" fast="false" fbc:lowerFluxBound="FB1N1000" fbc:upperFluxBound="FB3N1000">

<listOfReactants>

<speciesReference species="M_Threonine__91__n__93____91__c__93__" stoichiometry="1" constant="true"/>

</listOfReactants>

<listOfProducts>

<speciesReference species="M_Threonine__91__n__93__" stoichiometry="1" constant="true"/>

</listOfProducts>

</reaction>

<reaction metaid="Iso_cl_export_night" id="Iso_cl_export_night" name="Iso_cl_export_night" reversible="true" fast="false" fbc:lowerFluxBound="FB1N1000" fbc:upperFluxBound="FB3N1000">

<listOfReactants>

<speciesReference species="M_Isoleucine__91__n__93____91__c__93__" stoichiometry="1" constant="true"/>

</listOfReactants>

<listOfProducts>

<speciesReference species="M_Isoleucine__91__n__93__" stoichiometry="1" constant="true"/>

</listOfProducts>

</reaction>

<reaction metaid="Val_cl_export_night" id="Val_cl_export_night" name="Val_cl_export_night" reversible="true" fast="false" fbc:lowerFluxBound="FB1N1000" fbc:upperFluxBound="FB3N1000">

<listOfReactants>

<speciesReference species="M_Valine__91__n__93____91__c__93__" stoichiometry="1" constant="true"/>

</listOfReactants>

<listOfProducts>

<speciesReference species="M_Valine__91__n__93__" stoichiometry="1" constant="true"/>

</listOfProducts>

</reaction>

<reaction metaid="Ala_cl_export_night" id="Ala_cl_export_night" name="Ala_cl_export_night" reversible="true" fast="false" fbc:lowerFluxBound="FB1N1000" fbc:upperFluxBound="FB3N1000">

<listOfReactants>

<speciesReference species="M_Alanine__91__n__93____91__c__93__" stoichiometry="1" constant="true"/>

</listOfReactants>

<listOfProducts>

<speciesReference species="M_Alanine__91__n__93__" stoichiometry="1" constant="true"/>

</listOfProducts>

</reaction>

<reaction metaid="Leu_cl_export_night" id="Leu_cl_export_night" name="Leu_cl_export_night" reversible="true" fast="false" fbc:lowerFluxBound="FB1N1000" fbc:upperFluxBound="FB3N1000">

<listOfReactants>

<speciesReference species="M_Leucine__91__n__93____91__c__93__" stoichiometry="1" constant="true"/>

</listOfReactants>

<listOfProducts>

<speciesReference species="M_Leucine__91__n__93__" stoichiometry="1" constant="true"/>

</listOfProducts>

</reaction>

<reaction metaid="Cys_cl_exp_night" id="Cys_cl_exp_night" name="Cys_cl_exp_night" reversible="true" fast="false" fbc:lowerFluxBound="FB1N1000" fbc:upperFluxBound="FB3N1000">

<listOfReactants>

<speciesReference species="M_Cystine__91__n__93____91__c__93__" stoichiometry="1" constant="true"/>

</listOfReactants>

<listOfProducts>

<speciesReference species="M_Cystine__91__n__93__" stoichiometry="1" constant="true"/>

</listOfProducts>

</reaction>

<reaction metaid="Gln_cl_export_night" id="Gln_cl_export_night" name="Gln_cl_export_night" reversible="false" fast="false" fbc:lowerFluxBound="FB2N0" fbc:upperFluxBound="FB3N1000">

<listOfReactants>

<speciesReference species="M_Glutamine__91__n__93__" stoichiometry="1" constant="true"/>

</listOfReactants>

<listOfProducts>

<speciesReference species="M_Glutamine__91__n__93____91__c__93__" stoichiometry="1" constant="true"/>

</listOfProducts>

</reaction>

<reaction metaid="Glu_cl_export_night" id="Glu_cl_export_night" name="Glu_cl_export_night" reversible="true" fast="false" fbc:lowerFluxBound="FB1N1000" fbc:upperFluxBound="FB3N1000">

<listOfReactants>

<speciesReference species="M_Glutamate__91__n__93____91__c__93__" stoichiometry="1" constant="true"/>

</listOfReactants>

<listOfProducts>

<speciesReference species="M_Glutamate__91__n__93__" stoichiometry="1" constant="true"/>

</listOfProducts>

</reaction>

<reaction metaid="Glu_cl_per_export_night" id="Glu_cl_per_export_night" name="Glu_cl_per_export_night" reversible="true" fast="false" fbc:lowerFluxBound="FB1N1000" fbc:upperFluxBound="FB3N1000">

<listOfReactants>

<speciesReference species="M_Glutamate__91__n__93____91__p__93__" stoichiometry="1" constant="true"/>

</listOfReactants>

<listOfProducts>

<speciesReference species="M_Glutamate__91__n__93__" stoichiometry="1" constant="true"/>

</listOfProducts>

</reaction>

<reaction metaid="Pro_cl_export_night" id="Pro_cl_export_night" name="Pro_cl_export_night" reversible="true" fast="false" fbc:lowerFluxBound="FB1N1000" fbc:upperFluxBound="FB3N1000">

<listOfReactants>

<speciesReference species="M_Proline__91__n__93____91__c__93__" stoichiometry="1" constant="true"/>

</listOfReactants>

<listOfProducts>

<speciesReference species="M_Proline__91__n__93__" stoichiometry="1" constant="true"/>

</listOfProducts>

</reaction>

<reaction metaid="Arg_cl_export_night" id="Arg_cl_export_night" name="Arg_cl_export_night" reversible="true" fast="false" fbc:lowerFluxBound="FB1N1000" fbc:upperFluxBound="FB3N1000">

<listOfReactants>

<speciesReference species="M_Arginine__91__n__93____91__c__93__" stoichiometry="1" constant="true"/>

</listOfReactants>

<listOfProducts>

<speciesReference species="M_Arginine__91__n__93__" stoichiometry="1" constant="true"/>

</listOfProducts>

</reaction>

<reaction metaid="Tyr_cl_export_night" id="Tyr_cl_export_night" name="Tyr_cl_export_night" reversible="true" fast="false" fbc:lowerFluxBound="FB1N1000" fbc:upperFluxBound="FB3N1000">

<listOfReactants>

<speciesReference species="M_Tyrosine__91__n__93____91__c__93__" stoichiometry="1" constant="true"/>

</listOfReactants>

<listOfProducts>

<speciesReference species="M_Tyrosine__91__n__93__" stoichiometry="1" constant="true"/>

</listOfProducts>

</reaction>

<reaction metaid="Phe_cl_export_night" id="Phe_cl_export_night" name="Phe_cl_export_night" reversible="true" fast="false" fbc:lowerFluxBound="FB1N1000" fbc:upperFluxBound="FB3N1000">

<listOfReactants>

<speciesReference species="M_Phenylalanine__91__n__93____91__c__93__" stoichiometry="1" constant="true"/>

</listOfReactants>

<listOfProducts>

<speciesReference species="M_Phenylalanine__91__n__93__" stoichiometry="1" constant="true"/>

</listOfProducts>

</reaction>

<reaction metaid="Trp_cl_export_night" id="Trp_cl_export_night" name="Trp_cl_export_night" reversible="true" fast="false" fbc:lowerFluxBound="FB1N1000" fbc:upperFluxBound="FB3N1000">

<listOfReactants>

<speciesReference species="M_Tryptophan__91__n__93____91__c__93__" stoichiometry="1" constant="true"/>

</listOfReactants>

<listOfProducts>

<speciesReference species="M_Tryptophan__91__n__93__" stoichiometry="1" constant="true"/>

</listOfProducts>

</reaction>

<reaction metaid="His_cl_export_night" id="His_cl_export_night" name="His_cl_export_night" reversible="true" fast="false" fbc:lowerFluxBound="FB1N1000" fbc:upperFluxBound="FB3N1000">

<listOfReactants>

<speciesReference species="M_Histidine__91__n__93____91__c__93__" stoichiometry="1" constant="true"/>

</listOfReactants>

<listOfProducts>

<speciesReference species="M_Histidine__91__n__93__" stoichiometry="1" constant="true"/>

</listOfProducts>

</reaction>

<reaction metaid="Met_cl_export_night" id="Met_cl_export_night" name="Met_cl_export_night" reversible="true" fast="false" fbc:lowerFluxBound="FB1N1000" fbc:upperFluxBound="FB3N1000">

<listOfReactants>

<speciesReference species="M_Homocysteine__91__n__93____91__c__93__" stoichiometry="1" constant="true"/>

</listOfReactants>

<listOfProducts>

<speciesReference species="M_Homocysteine__91__n__93__" stoichiometry="1" constant="true"/>

</listOfProducts>

</reaction>

<reaction metaid="Ser_cl_export_night" id="Ser_cl_export_night" name="Ser_cl_export_night" reversible="true" fast="false" fbc:lowerFluxBound="FB1N1000" fbc:upperFluxBound="FB3N1000">

<listOfReactants>

<speciesReference species="M_Serine__91__n__93____91__c__93__" stoichiometry="1" constant="true"/>

</listOfReactants>

<listOfProducts>

<speciesReference species="M_Serine__91__n__93__" stoichiometry="1" constant="true"/>

</listOfProducts>

</reaction>

<reaction metaid="Lys_cl_exp_night" id="Lys_cl_exp_night" name="Lys_cl_exp_night" reversible="true" fast="false" fbc:lowerFluxBound="FB1N1000" fbc:upperFluxBound="FB3N1000">

<listOfReactants>

<speciesReference species="M_Lysine__91__n__93____91__c__93__" stoichiometry="1" constant="true"/>

</listOfReactants>

<listOfProducts>

<speciesReference species="M_Lysine__91__n__93__" stoichiometry="1" constant="true"/>

</listOfProducts>

</reaction>

<reaction metaid="Cysteine_cl_exp_night" id="Cysteine_cl_exp_night" name="Cysteine_cl_exp_night" reversible="true" fast="false" fbc:lowerFluxBound="FB1N1000" fbc:upperFluxBound="FB3N1000">

<listOfReactants>

<speciesReference species="M_Cysteine__91__n__93____91__c__93__" stoichiometry="1" constant="true"/>

</listOfReactants>

<listOfProducts>

<speciesReference species="M_Cysteine__91__n__93__" stoichiometry="1" constant="true"/>

</listOfProducts>

</reaction>

<reaction metaid="Gly_exp_night" id="Gly_exp_night" name="Gly_exp_night" reversible="true" fast="false" fbc:lowerFluxBound="FB1N1000" fbc:upperFluxBound="FB3N1000">

<listOfReactants>

<speciesReference species="M_Glycine__91__n__93____91__m__93__" stoichiometry="1" constant="true"/>

</listOfReactants>

<listOfProducts>

<speciesReference species="M_Glycine__91__n__93__" stoichiometry="1" constant="true"/>

</listOfProducts>

</reaction>

<reaction metaid="Ser_exp_night" id="Ser_exp_night" name="Ser_exp_night" reversible="true" fast="false" fbc:lowerFluxBound="FB1N1000" fbc:upperFluxBound="FB3N1000">

<listOfReactants>

<speciesReference species="M_Serine__91__n__93____91__m__93__" stoichiometry="1" constant="true"/>

</listOfReactants>

<listOfProducts>

<speciesReference species="M_Serine__91__n__93__" stoichiometry="1" constant="true"/>

</listOfProducts>

</reaction>

<reaction metaid="Gly_cl_exp_night" id="Gly_cl_exp_night" name="Gly_cl_exp_night" reversible="true" fast="false" fbc:lowerFluxBound="FB1N1000" fbc:upperFluxBound="FB3N1000">

<listOfReactants>

<speciesReference species="M_Glycine__91__n__93____91__c__93__" stoichiometry="1" constant="true"/>

</listOfReactants>

<listOfProducts>

<speciesReference species="M_Glycine__91__n__93__" stoichiometry="1" constant="true"/>

</listOfProducts>

</reaction>

<reaction metaid="Sulfate_cl_trans_night" id="Sulfate_cl_trans_night" name="Sulfate_cl_trans_night" reversible="true" fast="false" fbc:lowerFluxBound="FB1N1000" fbc:upperFluxBound="FB3N1000">

<listOfReactants>

<speciesReference species="M_Sulfate__91__n__93____91__c__93__" stoichiometry="1" constant="true"/>

</listOfReactants>

<listOfProducts>

<speciesReference species="M_Sulfate__91__n__93__" stoichiometry="1" constant="true"/>

</listOfProducts>

</reaction>

<reaction metaid="H2S_cl_trans_night" id="H2S_cl_trans_night" name="H2S_cl_trans_night" reversible="true" fast="false" fbc:lowerFluxBound="FB1N1000" fbc:upperFluxBound="FB3N1000">

<listOfReactants>

<speciesReference species="M_H2S__91__n__93____91__c__93__" stoichiometry="1" constant="true"/>

</listOfReactants>

<listOfProducts>

<speciesReference species="M_H2S__91__n__93__" stoichiometry="1" constant="true"/>

</listOfProducts>

</reaction>

<reaction metaid="H2S_mit_trans_night" id="H2S_mit_trans_night" name="H2S_mit_trans_night" reversible="true" fast="false" fbc:lowerFluxBound="FB1N1000" fbc:upperFluxBound="FB3N1000">

<listOfReactants>

<speciesReference species="M_H2S__91__n__93____91__m__93__" stoichiometry="1" constant="true"/>

</listOfReactants>

<listOfProducts>

<speciesReference species="M_H2S__91__n__93__" stoichiometry="1" constant="true"/>

</listOfProducts>

</reaction>

<reaction metaid="ATP_mitexport_night" id="ATP_mitexport_night" name="ATP_mitexport_night" reversible="false" fast="false" fbc:lowerFluxBound="FB2N0" fbc:upperFluxBound="FB3N1000">

<listOfReactants>

<speciesReference species="M_ADP__91__n__93__" stoichiometry="1" constant="true"/>

<speciesReference species="M_ATP__91__n__93____91__m__93__" stoichiometry="1" constant="true"/>

</listOfReactants>

<listOfProducts>

<speciesReference species="M_ADP__91__n__93____91__m__93__" stoichiometry="1" constant="true"/>

<speciesReference species="M_ATP__91__n__93__" stoichiometry="1" constant="true"/>

</listOfProducts>

</reaction>

<reaction metaid="ATP_clexport_night" id="ATP_clexport_night" name="ATP_clexport_night" reversible="true" fast="false" fbc:lowerFluxBound="FB1N1000" fbc:upperFluxBound="FB3N1000">

<listOfReactants>

<speciesReference species="M_ADP__91__n__93____91__c__93__" stoichiometry="1" constant="true"/>

<speciesReference species="M_ATP__91__n__93__" stoichiometry="1" constant="true"/>

<speciesReference species="M_Pi__91__n__93____91__c__93__" stoichiometry="1" constant="true"/>

</listOfReactants>

<listOfProducts>

<speciesReference species="M_ADP__91__n__93__" stoichiometry="1" constant="true"/>

<speciesReference species="M_ATP__91__n__93____91__c__93__" stoichiometry="1" constant="true"/>

<speciesReference species="M_Pi__91__n__93__" stoichiometry="1" constant="true"/>

</listOfProducts>

</reaction>

<reaction metaid="GluAsp_Antiporter_mit_night" id="GluAsp_Antiporter_mit_night" name="GluAsp_Antiporter_mit_night" reversible="false" fast="false" fbc:lowerFluxBound="FB2N0" fbc:upperFluxBound="FB3N1000">

<listOfReactants>

<speciesReference species="M_Aspartate__91__n__93____91__m__93__" stoichiometry="1" constant="true"/>

<speciesReference species="M_Glutamate__91__n__93__" stoichiometry="1" constant="true"/>

</listOfReactants>

<listOfProducts>

<speciesReference species="M_Aspartate__91__n__93__" stoichiometry="1" constant="true"/>

<speciesReference species="M_Glutamate__91__n__93____91__m__93__" stoichiometry="1" constant="true"/>

</listOfProducts>

</reaction>

<reaction metaid="DiT1_night" id="DiT1_night" name="DiT1_night" reversible="false" fast="false" fbc:lowerFluxBound="FB2N0" fbc:upperFluxBound="FB3N1000">

<listOfReactants>

<speciesReference species="M_Glutamate__91__n__93____91__c__93__" stoichiometry="1" constant="true"/>

<speciesReference species="M_Malate__91__n__93__" stoichiometry="1" constant="true"/>

</listOfReactants>

<listOfProducts>

<speciesReference species="M_Glutamate__91__n__93__" stoichiometry="1" constant="true"/>

<speciesReference species="M_Malate__91__n__93____91__c__93__" stoichiometry="1" constant="true"/>

</listOfProducts>

</reaction>

<reaction metaid="DiT2_night" id="DiT2_night" name="DiT2_night" reversible="false" fast="false" fbc:lowerFluxBound="FB2N0" fbc:upperFluxBound="FB3N1000">

<listOfReactants>

<speciesReference species="M_Malate__91__n__93____91__c__93__" stoichiometry="1" constant="true"/>

<speciesReference species="M_Oxoglutarate__91__n__93__" stoichiometry="1" constant="true"/>

</listOfReactants>

<listOfProducts>

<speciesReference species="M_Malate__91__n__93__" stoichiometry="1" constant="true"/>

<speciesReference species="M_Oxoglutarate__91__n__93____91__c__93__" stoichiometry="1" constant="true"/>

</listOfProducts>

</reaction>

<reaction metaid="MalOAA_cl_night" id="MalOAA_cl_night" name="MalOAA_cl_night" reversible="true" fast="false" fbc:lowerFluxBound="FB1N1000" fbc:upperFluxBound="FB3N1000">

<listOfReactants>

<speciesReference species="M_Malate__91__n__93__" stoichiometry="1" constant="true"/>

<speciesReference species="M_Oxaloacetate__91__n__93____91__c__93__" stoichiometry="1" constant="true"/>

</listOfReactants>

<listOfProducts>

<speciesReference species="M_Malate__91__n__93____91__c__93__" stoichiometry="1" constant="true"/>

<speciesReference species="M_Oxaloacetate__91__n__93__" stoichiometry="1" constant="true"/>

</listOfProducts>

</reaction>

<reaction metaid="MalOAA_per_night" id="MalOAA_per_night" name="MalOAA_per_night" reversible="true" fast="false" fbc:lowerFluxBound="FB1N1000" fbc:upperFluxBound="FB3N1000">

<listOfReactants>

<speciesReference species="M_Malate__91__n__93__" stoichiometry="1" constant="true"/>

<speciesReference species="M_Oxaloacetate__91__n__93____91__p__93__" stoichiometry="1" constant="true"/>

</listOfReactants>

<listOfProducts>

<speciesReference species="M_Malate__91__n__93____91__p__93__" stoichiometry="1" constant="true"/>

<speciesReference species="M_Oxaloacetate__91__n__93__" stoichiometry="1" constant="true"/>

</listOfProducts>

</reaction>

<reaction metaid="MalOAA_mit_night" id="MalOAA_mit_night" name="MalOAA_mit_night" reversible="true" fast="false" fbc:lowerFluxBound="FB1N1000" fbc:upperFluxBound="FB3N1000">

<listOfReactants>

<speciesReference species="M_Malate__91__n__93__" stoichiometry="1" constant="true"/>

<speciesReference species="M_Oxaloacetate__91__n__93____91__m__93__" stoichiometry="1" constant="true"/>

</listOfReactants>

<listOfProducts>

<speciesReference species="M_Malate__91__n__93____91__m__93__" stoichiometry="1" constant="true"/>

<speciesReference species="M_Oxaloacetate__91__n__93__" stoichiometry="1" constant="true"/>

</listOfProducts>

</reaction>

<reaction metaid="AAT_cyt_night" id="AAT_cyt_night" name="AAT_cyt_night" reversible="true" fast="false" fbc:lowerFluxBound="FB1N1000" fbc:upperFluxBound="FB3N1000">

<listOfReactants>

<speciesReference species="M_Glutamate__91__n__93____91__c__93__" stoichiometry="1" constant="true"/>

<speciesReference species="M_Oxaloacetate__91__n__93____91__c__93__" stoichiometry="1" constant="true"/>

</listOfReactants>

<listOfProducts>

<speciesReference species="M_Aspartate__91__n__93____91__c__93__" stoichiometry="1" constant="true"/>

<speciesReference species="M_Oxoglutarate__91__n__93____91__c__93__" stoichiometry="1" constant="true"/>

</listOfProducts>

</reaction>

<reaction metaid="AAT_mit_night" id="AAT_mit_night" name="AAT_mit_night" reversible="true" fast="false" fbc:lowerFluxBound="FB1N1000" fbc:upperFluxBound="FB3N1000">

<listOfReactants>

<speciesReference species="M_Glutamate__91__n__93____91__m__93__" stoichiometry="1" constant="true"/>

<speciesReference species="M_Oxaloacetate__91__n__93____91__m__93__" stoichiometry="1" constant="true"/>

</listOfReactants>

<listOfProducts>

<speciesReference species="M_Aspartate__91__n__93____91__m__93__" stoichiometry="1" constant="true"/>

<speciesReference species="M_Oxoglutarate__91__n__93____91__m__93__" stoichiometry="1" constant="true"/>

</listOfProducts>

</reaction>

<reaction metaid="AAT_per_night" id="AAT_per_night" name="AAT_per_night" reversible="true" fast="false" fbc:lowerFluxBound="FB1N1000" fbc:upperFluxBound="FB3N1000">

<listOfReactants>

<speciesReference species="M_Glutamate__91__n__93____91__p__93__" stoichiometry="1" constant="true"/>

</listOfReactants>

<listOfProducts>

<speciesReference species="M_Glutamate__91__n__93__" stoichiometry="1" constant="true"/>

</listOfProducts>

</reaction>

<reaction metaid="Oxo_mit_cyt_night" id="Oxo_mit_cyt_night" name="Oxo_mit_cyt_night" reversible="false" fast="false" fbc:lowerFluxBound="FB2N0" fbc:upperFluxBound="FB3N1000">

<listOfReactants>

<speciesReference species="M_Oxoglutarate__91__n__93____91__p__93__" stoichiometry="1" constant="true"/>

</listOfReactants>

<listOfProducts>

<speciesReference species="M_Oxoglutarate__91__n__93__" stoichiometry="1" constant="true"/>

</listOfProducts>

</reaction>

<reaction metaid="Glu_mit_cyt_night" id="Glu_mit_cyt_night" name="Glu_mit_cyt_night" reversible="true" fast="false" fbc:lowerFluxBound="FB1N1000" fbc:upperFluxBound="FB3N1000">

<listOfReactants>

<speciesReference species="M_Glutamate__91__n__93__" stoichiometry="1" constant="true"/>

</listOfReactants>

<listOfProducts>

<speciesReference species="M_Glutamate__91__n__93____91__p__93__" stoichiometry="1" constant="true"/>

</listOfProducts>

</reaction>

<reaction metaid="DTC_CitOxo_night" id="DTC_CitOxo_night" name="DTC_CitOxo_night" reversible="true" fast="false" fbc:lowerFluxBound="FB1N1000" fbc:upperFluxBound="FB3N1000">

<listOfReactants>

<speciesReference species="M_Citrate__91__n__93____91__m__93__" stoichiometry="1" constant="true"/>

<speciesReference species="M_Oxoglutarate__91__n__93__" stoichiometry="1" constant="true"/>

</listOfReactants>

<listOfProducts>

<speciesReference species="M_Citrate__91__n__93__" stoichiometry="1" constant="true"/>

<speciesReference species="M_Oxoglutarate__91__n__93____91__m__93__" stoichiometry="1" constant="true"/>

</listOfProducts>

</reaction>

<reaction metaid="DTC_CitIso_night" id="DTC_CitIso_night" name="DTC_CitIso_night" reversible="true" fast="false" fbc:lowerFluxBound="FB1N1000" fbc:upperFluxBound="FB3N1000">

<listOfReactants>

<speciesReference species="M_Citrate__91__n__93____91__m__93__" stoichiometry="1" constant="true"/>

<speciesReference species="M_Isocitrate__91__n__93__" stoichiometry="1" constant="true"/>

</listOfReactants>

<listOfProducts>

<speciesReference species="M_Citrate__91__n__93__" stoichiometry="1" constant="true"/>

<speciesReference species="M_Isocitrate__91__n__93____91__m__93__" stoichiometry="1" constant="true"/>

</listOfProducts>

</reaction>

<reaction metaid="DTC_CitCis_night" id="DTC_CitCis_night" name="DTC_CitCis_night" reversible="true" fast="false" fbc:lowerFluxBound="FB1N1000" fbc:upperFluxBound="FB3N1000">

<listOfReactants>

<speciesReference species="M_Cisaconitate__91__n__93__" stoichiometry="1" constant="true"/>

<speciesReference species="M_Citrate__91__n__93____91__m__93__" stoichiometry="1" constant="true"/>

</listOfReactants>

<listOfProducts>

<speciesReference species="M_Cisaconitate__91__n__93____91__m__93__" stoichiometry="1" constant="true"/>

<speciesReference species="M_Citrate__91__n__93__" stoichiometry="1" constant="true"/>

</listOfProducts>

</reaction>

<reaction metaid="DTC_IsoCis_night" id="DTC_IsoCis_night" name="DTC_IsoCis_night" reversible="true" fast="false" fbc:lowerFluxBound="FB1N1000" fbc:upperFluxBound="FB3N1000">

<listOfReactants>

<speciesReference species="M_Cisaconitate__91__n__93__" stoichiometry="1" constant="true"/>

<speciesReference species="M_Isocitrate__91__n__93____91__m__93__" stoichiometry="1" constant="true"/>

</listOfReactants>

<listOfProducts>

<speciesReference species="M_Cisaconitate__91__n__93____91__m__93__" stoichiometry="1" constant="true"/>

<speciesReference species="M_Isocitrate__91__n__93__" stoichiometry="1" constant="true"/>

</listOfProducts>

</reaction>

<reaction metaid="DTC_Oxo_Iso_night" id="DTC_Oxo_Iso_night" name="DTC_Oxo_Iso_night" reversible="true" fast="false" fbc:lowerFluxBound="FB1N1000" fbc:upperFluxBound="FB3N1000">

<listOfReactants>

<speciesReference species="M_Isocitrate__91__n__93__" stoichiometry="1" constant="true"/>

<speciesReference species="M_Oxoglutarate__91__n__93____91__m__93__" stoichiometry="1" constant="true"/>

</listOfReactants>

<listOfProducts>

<speciesReference species="M_Isocitrate__91__n__93____91__m__93__" stoichiometry="1" constant="true"/>

<speciesReference species="M_Oxoglutarate__91__n__93__" stoichiometry="1" constant="true"/>

</listOfProducts>

</reaction>

<reaction metaid="DTC_Oxo_Cisacon_night" id="DTC_Oxo_Cisacon_night" name="DTC_Oxo_Cisacon_night" reversible="true" fast="false" fbc:lowerFluxBound="FB1N1000" fbc:upperFluxBound="FB3N1000">

<listOfReactants>

<speciesReference species="M_Cisaconitate__91__n__93__" stoichiometry="1" constant="true"/>

<speciesReference species="M_Oxoglutarate__91__n__93____91__m__93__" stoichiometry="1" constant="true"/>

</listOfReactants>

<listOfProducts>

<speciesReference species="M_Cisaconitate__91__n__93____91__m__93__" stoichiometry="1" constant="true"/>

<speciesReference species="M_Oxoglutarate__91__n__93__" stoichiometry="1" constant="true"/>

</listOfProducts>

</reaction>

<reaction metaid="DTC_OAA_Succ_night" id="DTC_OAA_Succ_night" name="DTC_OAA_Succ_night" reversible="true" fast="false" fbc:lowerFluxBound="FB1N1000" fbc:upperFluxBound="FB3N1000">

<listOfReactants>

<speciesReference species="M_Oxaloacetate__91__n__93____91__m__93__" stoichiometry="1" constant="true"/>

<speciesReference species="M_Succinate__91__n__93__" stoichiometry="1" constant="true"/>

</listOfReactants>

<listOfProducts>

<speciesReference species="M_Oxaloacetate__91__n__93__" stoichiometry="1" constant="true"/>

<speciesReference species="M_Succinate__91__n__93____91__m__93__" stoichiometry="1" constant="true"/>

</listOfProducts>

</reaction>

<reaction metaid="DTC_OAA_Oxo_night" id="DTC_OAA_Oxo_night" name="DTC_OAA_Oxo_night" reversible="true" fast="false" fbc:lowerFluxBound="FB1N1000" fbc:upperFluxBound="FB3N1000">

<listOfReactants>

<speciesReference species="M_Oxaloacetate__91__n__93____91__m__93__" stoichiometry="1" constant="true"/>

<speciesReference species="M_Oxoglutarate__91__n__93__" stoichiometry="1" constant="true"/>

</listOfReactants>

<listOfProducts>

<speciesReference species="M_Oxaloacetate__91__n__93__" stoichiometry="1" constant="true"/>

<speciesReference species="M_Oxoglutarate__91__n__93____91__m__93__" stoichiometry="1" constant="true"/>

</listOfProducts>

</reaction>

<reaction metaid="DTC_OAA_Cit_night" id="DTC_OAA_Cit_night" name="DTC_OAA_Cit_night" reversible="true" fast="false" fbc:lowerFluxBound="FB1N1000" fbc:upperFluxBound="FB3N1000">

<listOfReactants>

<speciesReference species="M_Citrate__91__n__93__" stoichiometry="1" constant="true"/>

<speciesReference species="M_Oxaloacetate__91__n__93____91__m__93__" stoichiometry="1" constant="true"/>

</listOfReactants>

<listOfProducts>

<speciesReference species="M_Citrate__91__n__93____91__m__93__" stoichiometry="1" constant="true"/>

<speciesReference species="M_Oxaloacetate__91__n__93__" stoichiometry="1" constant="true"/>

</listOfProducts>

</reaction>

<reaction metaid="DTC_OAA_Isocit_night" id="DTC_OAA_Isocit_night" name="DTC_OAA_Isocit_night" reversible="true" fast="false" fbc:lowerFluxBound="FB1N1000" fbc:upperFluxBound="FB3N1000">

<listOfReactants>

<speciesReference species="M_Isocitrate__91__n__93__" stoichiometry="1" constant="true"/>

<speciesReference species="M_Oxaloacetate__91__n__93____91__m__93__" stoichiometry="1" constant="true"/>

</listOfReactants>

<listOfProducts>

<speciesReference species="M_Isocitrate__91__n__93____91__m__93__" stoichiometry="1" constant="true"/>

<speciesReference species="M_Oxaloacetate__91__n__93__" stoichiometry="1" constant="true"/>

</listOfProducts>

</reaction>

<reaction metaid="DTC_OAA_Cis_night" id="DTC_OAA_Cis_night" name="DTC_OAA_Cis_night" reversible="true" fast="false" fbc:lowerFluxBound="FB1N1000" fbc:upperFluxBound="FB3N1000">

<listOfReactants>

<speciesReference species="M_Cisaconitate__91__n__93__" stoichiometry="1" constant="true"/>

<speciesReference species="M_Oxaloacetate__91__n__93____91__m__93__" stoichiometry="1" constant="true"/>

</listOfReactants>

<listOfProducts>

<speciesReference species="M_Cisaconitate__91__n__93____91__m__93__" stoichiometry="1" constant="true"/>

<speciesReference species="M_Oxaloacetate__91__n__93__" stoichiometry="1" constant="true"/>

</listOfProducts>

</reaction>

<reaction metaid="DTC_MAL_CIS_night" id="DTC_MAL_CIS_night" name="DTC_MAL_CIS_night" reversible="true" fast="false" fbc:lowerFluxBound="FB1N1000" fbc:upperFluxBound="FB3N1000">

<listOfReactants>

<speciesReference species="M_Cisaconitate__91__n__93__" stoichiometry="1" constant="true"/>

<speciesReference species="M_Malate__91__n__93____91__m__93__" stoichiometry="1" constant="true"/>

</listOfReactants>

<listOfProducts>

<speciesReference species="M_Cisaconitate__91__n__93____91__m__93__" stoichiometry="1" constant="true"/>

<speciesReference species="M_Malate__91__n__93__" stoichiometry="1" constant="true"/>

</listOfProducts>

</reaction>

<reaction metaid="DTC_Mal_Suc_night" id="DTC_Mal_Suc_night" name="DTC_Mal_Suc_night" reversible="true" fast="false" fbc:lowerFluxBound="FB1N1000" fbc:upperFluxBound="FB3N1000">

<listOfReactants>

<speciesReference species="M_Malate__91__n__93____91__m__93__" stoichiometry="1" constant="true"/>

<speciesReference species="M_Succinate__91__n__93__" stoichiometry="1" constant="true"/>

</listOfReactants>

<listOfProducts>

<speciesReference species="M_Malate__91__n__93__" stoichiometry="1" constant="true"/>

<speciesReference species="M_Succinate__91__n__93____91__m__93__" stoichiometry="1" constant="true"/>

</listOfProducts>

</reaction>

<reaction metaid="DTC_Mal_Oxo_night" id="DTC_Mal_Oxo_night" name="DTC_Mal_Oxo_night" reversible="true" fast="false" fbc:lowerFluxBound="FB1N1000" fbc:upperFluxBound="FB3N1000">

<listOfReactants>

<speciesReference species="M_Malate__91__n__93____91__m__93__" stoichiometry="1" constant="true"/>

<speciesReference species="M_Oxoglutarate__91__n__93__" stoichiometry="1" constant="true"/>

</listOfReactants>

<listOfProducts>

<speciesReference species="M_Malate__91__n__93__" stoichiometry="1" constant="true"/>

<speciesReference species="M_Oxoglutarate__91__n__93____91__m__93__" stoichiometry="1" constant="true"/>

</listOfProducts>

</reaction>

<reaction metaid="DTC_Mal_Cit_night" id="DTC_Mal_Cit_night" name="DTC_Mal_Cit_night" reversible="true" fast="false" fbc:lowerFluxBound="FB1N1000" fbc:upperFluxBound="FB3N1000">

<listOfReactants>

<speciesReference species="M_Citrate__91__n__93__" stoichiometry="1" constant="true"/>

<speciesReference species="M_Malate__91__n__93____91__m__93__" stoichiometry="1" constant="true"/>

</listOfReactants>

<listOfProducts>

<speciesReference species="M_Citrate__91__n__93____91__m__93__" stoichiometry="1" constant="true"/>

<speciesReference species="M_Malate__91__n__93__" stoichiometry="1" constant="true"/>

</listOfProducts>

</reaction>

<reaction metaid="DTC_Mal_Isocit_night" id="DTC_Mal_Isocit_night" name="DTC_Mal_Isocit_night" reversible="true" fast="false" fbc:lowerFluxBound="FB1N1000" fbc:upperFluxBound="FB3N1000">

<listOfReactants>

<speciesReference species="M_Isocitrate__91__n__93__" stoichiometry="1" constant="true"/>

<speciesReference species="M_Malate__91__n__93____91__m__93__" stoichiometry="1" constant="true"/>

</listOfReactants>

<listOfProducts>

<speciesReference species="M_Isocitrate__91__n__93____91__m__93__" stoichiometry="1" constant="true"/>

<speciesReference species="M_Malate__91__n__93__" stoichiometry="1" constant="true"/>

</listOfProducts>

</reaction>

<reaction metaid="DTC_Suc_Oxo_night" id="DTC_Suc_Oxo_night" name="DTC_Suc_Oxo_night" reversible="true" fast="false" fbc:lowerFluxBound="FB1N1000" fbc:upperFluxBound="FB3N1000">

<listOfReactants>

<speciesReference species="M_Oxoglutarate__91__n__93__" stoichiometry="1" constant="true"/>

<speciesReference species="M_Succinate__91__n__93____91__m__93__" stoichiometry="1" constant="true"/>

</listOfReactants>

<listOfProducts>

<speciesReference species="M_Oxoglutarate__91__n__93____91__m__93__" stoichiometry="1" constant="true"/>

<speciesReference species="M_Succinate__91__n__93__" stoichiometry="1" constant="true"/>

</listOfProducts>

</reaction>

<reaction metaid="DTC_Suc_Cit_night" id="DTC_Suc_Cit_night" name="DTC_Suc_Cit_night" reversible="true" fast="false" fbc:lowerFluxBound="FB1N1000" fbc:upperFluxBound="FB3N1000">

<listOfReactants>

<speciesReference species="M_Citrate__91__n__93__" stoichiometry="1" constant="true"/>

<speciesReference species="M_Succinate__91__n__93____91__m__93__" stoichiometry="1" constant="true"/>

</listOfReactants>

<listOfProducts>

<speciesReference species="M_Citrate__91__n__93____91__m__93__" stoichiometry="1" constant="true"/>

<speciesReference species="M_Succinate__91__n__93__" stoichiometry="1" constant="true"/>

</listOfProducts>

</reaction>

<reaction metaid="DTC_Suc_Isocit_night" id="DTC_Suc_Isocit_night" name="DTC_Suc_Isocit_night" reversible="true" fast="false" fbc:lowerFluxBound="FB1N1000" fbc:upperFluxBound="FB3N1000">

<listOfReactants>

<speciesReference species="M_Isocitrate__91__n__93__" stoichiometry="1" constant="true"/>

<speciesReference species="M_Succinate__91__n__93____91__m__93__" stoichiometry="1" constant="true"/>

</listOfReactants>

<listOfProducts>

<speciesReference species="M_Isocitrate__91__n__93____91__m__93__" stoichiometry="1" constant="true"/>

<speciesReference species="M_Succinate__91__n__93__" stoichiometry="1" constant="true"/>

</listOfProducts>

</reaction>

<reaction metaid="DTC_Suc_Cisacont_night" id="DTC_Suc_Cisacont_night" name="DTC_Suc_Cisacont_night" reversible="true" fast="false" fbc:lowerFluxBound="FB1N1000" fbc:upperFluxBound="FB3N1000">

<listOfReactants>

<speciesReference species="M_Cisaconitate__91__n__93__" stoichiometry="1" constant="true"/>

<speciesReference species="M_Succinate__91__n__93____91__m__93__" stoichiometry="1" constant="true"/>

</listOfReactants>

<listOfProducts>

<speciesReference species="M_Cisaconitate__91__n__93____91__m__93__" stoichiometry="1" constant="true"/>

<speciesReference species="M_Succinate__91__n__93__" stoichiometry="1" constant="true"/>

</listOfProducts>

</reaction>

<reaction metaid="Succ_Fum_transport_night" id="Succ_Fum_transport_night" name="Succ_Fum_transport_night" reversible="true" fast="false" fbc:lowerFluxBound="FB1N1000" fbc:upperFluxBound="FB3N1000">

<listOfReactants>

<speciesReference species="M_Fumarate__91__n__93__" stoichiometry="1" constant="true"/>

<speciesReference species="M_Succinate__91__n__93____91__m__93__" stoichiometry="1" constant="true"/>

</listOfReactants>

<listOfProducts>

<speciesReference species="M_Fumarate__91__n__93____91__m__93__" stoichiometry="1" constant="true"/>

<speciesReference species="M_Succinate__91__n__93__" stoichiometry="1" constant="true"/>

</listOfProducts>

</reaction>

<reaction metaid="OAA_Pi_trans_night" id="OAA_Pi_trans_night" name="OAA_Pi_trans_night" reversible="true" fast="false" fbc:lowerFluxBound="FB1N1000" fbc:upperFluxBound="FB3N1000">

<listOfReactants>

<speciesReference species="M_Oxaloacetate__91__n__93____91__m__93__" stoichiometry="1" constant="true"/>

<speciesReference species="M_Pi__91__n__93__" stoichiometry="1" constant="true"/>

</listOfReactants>

<listOfProducts>

<speciesReference species="M_Oxaloacetate__91__n__93__" stoichiometry="1" constant="true"/>

<speciesReference species="M_Pi__91__n__93____91__m__93__" stoichiometry="1" constant="true"/>

</listOfProducts>

</reaction>

<reaction metaid="GlutamateGln_Trans_night" id="GlutamateGln_Trans_night" name="GlutamateGln_Trans_night" reversible="true" fast="false" fbc:lowerFluxBound="FB1N1000" fbc:upperFluxBound="FB3N1000">

<listOfReactants>

<speciesReference species="M_Glutamate__91__n__93__" stoichiometry="1" constant="true"/>

<speciesReference species="M_Glutamine__91__n__93____91__m__93__" stoichiometry="1" constant="true"/>

</listOfReactants>

<listOfProducts>

<speciesReference species="M_Glutamate__91__n__93____91__m__93__" stoichiometry="1" constant="true"/>

<speciesReference species="M_Glutamine__91__n__93__" stoichiometry="1" constant="true"/>

</listOfProducts>

</reaction>

<reaction metaid="Mal_Pi_trans_night" id="Mal_Pi_trans_night" name="Mal_Pi_trans_night" reversible="true" fast="false" fbc:lowerFluxBound="FB1N1000" fbc:upperFluxBound="FB3N1000">

<listOfReactants>

<speciesReference species="M_Malate__91__n__93____91__m__93__" stoichiometry="1" constant="true"/>

<speciesReference species="M_Pi__91__n__93__" stoichiometry="1" constant="true"/>

</listOfReactants>

<listOfProducts>

<speciesReference species="M_Malate__91__n__93__" stoichiometry="1" constant="true"/>

<speciesReference species="M_Pi__91__n__93____91__m__93__" stoichiometry="1" constant="true"/>

</listOfProducts>

</reaction>

<reaction metaid="Succ_Pi_transport_night" id="Succ_Pi_transport_night" name="Succ_Pi_transport_night" reversible="true" fast="false" fbc:lowerFluxBound="FB1N1000" fbc:upperFluxBound="FB3N1000">

<listOfReactants>

<speciesReference species="M_Pi__91__n__93__" stoichiometry="1" constant="true"/>

<speciesReference species="M_Succinate__91__n__93____91__m__93__" stoichiometry="1" constant="true"/>

</listOfReactants>

<listOfProducts>

<speciesReference species="M_Pi__91__n__93____91__m__93__" stoichiometry="1" constant="true"/>

<speciesReference species="M_Succinate__91__n__93__" stoichiometry="1" constant="true"/>

</listOfProducts>

</reaction>

<reaction metaid="ExportPEP_night" id="ExportPEP_night" name="ExportPEP_night" reversible="true" fast="false" fbc:lowerFluxBound="FB1N1000" fbc:upperFluxBound="FB3N1000">

<listOfReactants>

<speciesReference species="M_PEP__91__n__93____91__c__93__" stoichiometry="1" constant="true"/>

<speciesReference species="M_Pi__91__n__93__" stoichiometry="1" constant="true"/>

</listOfReactants>

<listOfProducts>

<speciesReference species="M_PEP__91__n__93__" stoichiometry="1" constant="true"/>

<speciesReference species="M_Pi__91__n__93____91__c__93__" stoichiometry="1" constant="true"/>

</listOfProducts>

</reaction>

<reaction metaid="ExportHexose_night" id="ExportHexose_night" name="ExportHexose_night" reversible="true" fast="false" fbc:lowerFluxBound="FB1N1000" fbc:upperFluxBound="FB3N1000">

<listOfReactants>

<speciesReference species="M_glucose__91__n__93____91__c__93__" stoichiometry="1" constant="true"/>

</listOfReactants>

<listOfProducts>

<speciesReference species="M_glucose__91__n__93__" stoichiometry="1" constant="true"/>

</listOfProducts>

</reaction>

<reaction metaid="Xu5P_tra_night" id="Xu5P_tra_night" name="Xu5P_tra_night" reversible="true" fast="false" fbc:lowerFluxBound="FB1N1000" fbc:upperFluxBound="FB3N1000">

<listOfReactants>

<speciesReference species="M_Pi__91__n__93__" stoichiometry="1" constant="true"/>

<speciesReference species="M_Xu5P__91__n__93____91__c__93__" stoichiometry="1" constant="true"/>

</listOfReactants>

<listOfProducts>

<speciesReference species="M_Pi__91__n__93____91__c__93__" stoichiometry="1" constant="true"/>

<speciesReference species="M_Xu5P__91__n__93__" stoichiometry="1" constant="true"/>

</listOfProducts>

</reaction>

<reaction metaid="Pyruvate_to_mit_night" id="Pyruvate_to_mit_night" name="Pyruvate_to_mit_night" reversible="false" fast="false" fbc:lowerFluxBound="FB2N0" fbc:upperFluxBound="FB3N1000">

<listOfReactants>

<speciesReference species="M_Pyruvate__91__n__93__" stoichiometry="1" constant="true"/>

</listOfReactants>

<listOfProducts>

<speciesReference species="M_Pyruvate__91__n__93____91__m__93__" stoichiometry="1" constant="true"/>

</listOfProducts>

</reaction>

<reaction metaid="Formate_Transp_night" id="Formate_Transp_night" name="Formate_Transp_night" reversible="true" fast="false" fbc:lowerFluxBound="FB1N1000" fbc:upperFluxBound="FB3N1000">

<listOfReactants>

<speciesReference species="M_Formate__91__n__93____91__m__93__" stoichiometry="1" constant="true"/>

</listOfReactants>

<listOfProducts>

<speciesReference species="M_Formate__91__n__93__" stoichiometry="1" constant="true"/>

</listOfProducts>

</reaction>

<reaction metaid="THF_Transp_night" id="THF_Transp_night" name="THF_Transp_night" reversible="true" fast="false" fbc:lowerFluxBound="FB1N1000" fbc:upperFluxBound="FB3N1000">

<listOfReactants>

<speciesReference species="M_THF__91__n__93__" stoichiometry="1" constant="true"/>

</listOfReactants>

<listOfProducts>

<speciesReference species="M_THF__91__n__93____91__m__93__" stoichiometry="1" constant="true"/>

</listOfProducts>

</reaction>

<reaction metaid="Glycolate_cl_per_night" id="Glycolate_cl_per_night" name="Glycolate_cl_per_night" reversible="false" fast="false" fbc:lowerFluxBound="FB2N0" fbc:upperFluxBound="FB3N1000">

<listOfReactants>

<speciesReference species="M_Glycolate__91__n__93____91__c__93__" stoichiometry="1" constant="true"/>

</listOfReactants>

<listOfProducts>

<speciesReference species="M_Glycolate__91__n__93____91__p__93__" stoichiometry="1" constant="true"/>

</listOfProducts>

</reaction>

<reaction metaid="Glycerate_per_cit_night" id="Glycerate_per_cit_night" name="Glycerate_per_cit_night" reversible="false" fast="false" fbc:lowerFluxBound="FB2N0" fbc:upperFluxBound="FB3N1000">

<listOfReactants>

<speciesReference species="M_Glycerate__91__n__93____91__p__93__" stoichiometry="1" constant="true"/>

</listOfReactants>

<listOfProducts>

<speciesReference species="M_Glycerate__91__n__93____91__c__93__" stoichiometry="1" constant="true"/>

</listOfProducts>

</reaction>

<reaction metaid="Serine_mit_per_night" id="Serine_mit_per_night" name="Serine_mit_per_night" reversible="false" fast="false" fbc:lowerFluxBound="FB2N0" fbc:upperFluxBound="FB3N1000">

<listOfReactants>

<speciesReference species="M_Serine__91__n__93____91__m__93__" stoichiometry="1" constant="true"/>

</listOfReactants>

<listOfProducts>

<speciesReference species="M_Serine__91__n__93____91__p__93__" stoichiometry="1" constant="true"/>

</listOfProducts>

</reaction>

<reaction metaid="Glycine_per_mit_night" id="Glycine_per_mit_night" name="Glycine_per_mit_night" reversible="true" fast="false" fbc:lowerFluxBound="FB1N1000" fbc:upperFluxBound="FB3N1000">

<listOfReactants>

<speciesReference species="M_Glycine__91__n__93____91__p__93__" stoichiometry="1" constant="true"/>

</listOfReactants>

<listOfProducts>

<speciesReference species="M_Glycine__91__n__93____91__m__93__" stoichiometry="1" constant="true"/>

</listOfProducts>

</reaction>

<reaction metaid="Cysteine_mit_cyt_night" id="Cysteine_mit_cyt_night" name="Cysteine_mit_cyt_night" reversible="true" fast="false" fbc:lowerFluxBound="FB1N1000" fbc:upperFluxBound="FB3N1000">

<listOfReactants>

<speciesReference species="M_Cysteine__91__n__93____91__m__93__" stoichiometry="1" constant="true"/>

</listOfReactants>

<listOfProducts>

<speciesReference species="M_Cysteine__91__n__93__" stoichiometry="1" constant="true"/>

</listOfProducts>

</reaction>

<reaction metaid="Nitrite_transport_night" id="Nitrite_transport_night" name="Nitrite_transport_night" reversible="true" fast="false" fbc:lowerFluxBound="FB1N1000" fbc:upperFluxBound="FB3N1000">

<listOfReactants>

<speciesReference species="M_NO2__91__n__93____91__c__93__" stoichiometry="1" constant="true"/>

</listOfReactants>

<listOfProducts>

<speciesReference species="M_NO2__91__n__93__" stoichiometry="1" constant="true"/>

</listOfProducts>

</reaction>

<reaction metaid="Nitrate_transport_night" id="Nitrate_transport_night" name="Nitrate_transport_night" reversible="true" fast="false" fbc:lowerFluxBound="FB1N1000" fbc:upperFluxBound="FB3N1000">

<listOfReactants>

<speciesReference species="M_NH3__91__n__93____91__m__93__" stoichiometry="1" constant="true"/>

</listOfReactants>

<listOfProducts>

<speciesReference species="M_NH3__91__n__93__" stoichiometry="1" constant="true"/>

</listOfProducts>

</reaction>

<reaction metaid="Pitrans_night" id="Pitrans_night" name="Pitrans_night" reversible="true" fast="false" fbc:lowerFluxBound="FB1N1000" fbc:upperFluxBound="FB3N1000">

<listOfReactants>

<speciesReference species="M_Pi__91__n__93__" stoichiometry="1" constant="true"/>

</listOfReactants>

<listOfProducts>

<speciesReference species="M_Pi__91__n__93____91__m__93__" stoichiometry="1" constant="true"/>

</listOfProducts>

</reaction>

<reaction metaid="G3P_trans_night" id="G3P_trans_night" name="G3P_trans_night" reversible="false" fast="false" fbc:lowerFluxBound="FB2N0" fbc:upperFluxBound="FB3N1000">

<listOfReactants>

<speciesReference species="M_G3P__91__n__93____91__c__93__" stoichiometry="1" constant="true"/>

<speciesReference species="M_Pi__91__n__93__" stoichiometry="1" constant="true"/>

</listOfReactants>

<listOfProducts>

<speciesReference species="M_G3P__91__n__93__" stoichiometry="1" constant="true"/>

<speciesReference species="M_Pi__91__n__93____91__c__93__" stoichiometry="1" constant="true"/>

</listOfProducts>

</reaction>

<reaction metaid="DHAP_trans_night" id="DHAP_trans_night" name="DHAP_trans_night" reversible="false" fast="false" fbc:lowerFluxBound="FB2N0" fbc:upperFluxBound="FB3N1000">

<listOfReactants>

<speciesReference species="M_DHAP__91__n__93____91__c__93__" stoichiometry="1" constant="true"/>

<speciesReference species="M_Pi__91__n__93__" stoichiometry="1" constant="true"/>

</listOfReactants>

<listOfProducts>

<speciesReference species="M_DHAP__91__n__93__" stoichiometry="1" constant="true"/>

<speciesReference species="M_Pi__91__n__93____91__c__93__" stoichiometry="1" constant="true"/>

</listOfProducts>

</reaction>

<reaction metaid="PGA_trans_night" id="PGA_trans_night" name="PGA_trans_night" reversible="false" fast="false" fbc:lowerFluxBound="FB2N0" fbc:upperFluxBound="FB3N1000">

<listOfReactants>

<speciesReference species="M_3PGA__91__n__93__" stoichiometry="1" constant="true"/>

<speciesReference species="M_Pi__91__n__93____91__c__93__" stoichiometry="1" constant="true"/>

</listOfReactants>

<listOfProducts>

<speciesReference species="M_3PGA__91__n__93____91__c__93__" stoichiometry="1" constant="true"/>

<speciesReference species="M_Pi__91__n__93__" stoichiometry="1" constant="true"/>

</listOfProducts>

</reaction>

<reaction metaid="Ru5P_trans_night" id="Ru5P_trans_night" name="Ru5P_trans_night" reversible="false" fast="false" fbc:lowerFluxBound="FB2N0" fbc:upperFluxBound="FB3N1000">

<listOfReactants>

<speciesReference species="M_Pi__91__n__93____91__c__93__" stoichiometry="1" constant="true"/>

<speciesReference species="M_Ru5P__91__n__93__" stoichiometry="1" constant="true"/>

</listOfReactants>

<listOfProducts>

<speciesReference species="M_Pi__91__n__93__" stoichiometry="1" constant="true"/>

<speciesReference species="M_Ru5P__91__n__93____91__c__93__" stoichiometry="1" constant="true"/>

</listOfProducts>

</reaction>

<reaction metaid="E4P_trans_night" id="E4P_trans_night" name="E4P_trans_night" reversible="false" fast="false" fbc:lowerFluxBound="FB2N0" fbc:upperFluxBound="FB3N1000">

<listOfReactants>

<speciesReference species="M_E4P__91__n__93__" stoichiometry="1" constant="true"/>

<speciesReference species="M_Pi__91__n__93____91__c__93__" stoichiometry="1" constant="true"/>

</listOfReactants>

<listOfProducts>

<speciesReference species="M_E4P__91__n__93____91__c__93__" stoichiometry="1" constant="true"/>

<speciesReference species="M_Pi__91__n__93__" stoichiometry="1" constant="true"/>

</listOfProducts>

</reaction>

<reaction metaid="G6P_trans_night" id="G6P_trans_night" name="G6P_trans_night" reversible="false" fast="false" fbc:lowerFluxBound="FB2N0" fbc:upperFluxBound="FB3N1000">

<listOfReactants>

<speciesReference species="M_G6P__91__n__93__" stoichiometry="1" constant="true"/>

<speciesReference species="M_Pi__91__n__93____91__c__93__" stoichiometry="1" constant="true"/>

</listOfReactants>

<listOfProducts>

<speciesReference species="M_G6P__91__n__93____91__c__93__" stoichiometry="1" constant="true"/>

<speciesReference species="M_Pi__91__n__93__" stoichiometry="1" constant="true"/>

</listOfProducts>

</reaction>

<reaction metaid="LETC_n" id="LETC_n" name="LETC_n" reversible="false" fast="false" fbc:lowerFluxBound="FB2N0" fbc:upperFluxBound="FB3N1000">

<listOfReactants>

<speciesReference species="M_ADP__91__n__93____91__c__93__" stoichiometry="3" constant="true"/>

<speciesReference species="M_NADP__91__n__93____91__c__93__" stoichiometry="1" constant="true"/>

<speciesReference species="M_Pi__91__n__93____91__c__93__" stoichiometry="3" constant="true"/>

<speciesReference species="M_h700_n__91__c__93__" stoichiometry="7" constant="true"/>

</listOfReactants>

<listOfProducts>

<speciesReference species="M_ATP__91__n__93____91__c__93__" stoichiometry="3" constant="true"/>

<speciesReference species="M_H2O__91__n__93__" stoichiometry="2" constant="true"/>

<speciesReference species="M_NADPH__91__n__93____91__c__93__" stoichiometry="1" constant="true"/>

<speciesReference species="M_O2__91__n__93__" stoichiometry="0.5" constant="true"/>

</listOfProducts>

</reaction>

<reaction metaid="MP1_n" id="MP1_n" name="MP1_n" reversible="false" fast="false" fbc:lowerFluxBound="FB2N0" fbc:upperFluxBound="FB3N1000">

<listOfReactants>

<speciesReference species="M_ADP__91__n__93____91__m__93__" stoichiometry="2" constant="true"/>

<speciesReference species="M_FADH2__91__n__93____91__m__93__" stoichiometry="1" constant="true"/>

<speciesReference species="M_O2__91__n__93__" stoichiometry="0.5" constant="true"/>

<speciesReference species="M_Pi__91__n__93____91__m__93__" stoichiometry="2" constant="true"/>

</listOfReactants>

<listOfProducts>

<speciesReference species="M_ATP__91__n__93____91__m__93__" stoichiometry="2" constant="true"/>

<speciesReference species="M_FAD__91__n__93____91__m__93__" stoichiometry="1" constant="true"/>

<speciesReference species="M_H2O__91__n__93__" stoichiometry="3" constant="true"/>

</listOfProducts>

</reaction>

<reaction metaid="MP2_n" id="MP2_n" name="MP2_n" reversible="false" fast="false" fbc:lowerFluxBound="FB2N0" fbc:upperFluxBound="FB3N1000">

<listOfReactants>

<speciesReference species="M_ADP__91__n__93____91__m__93__" stoichiometry="3.33" constant="true"/>

<speciesReference species="M_NADH__91__n__93____91__m__93__" stoichiometry="1" constant="true"/>

<speciesReference species="M_O2__91__n__93__" stoichiometry="0.5" constant="true"/>

<speciesReference species="M_Pi__91__n__93____91__m__93__" stoichiometry="3.33" constant="true"/>

</listOfReactants>

<listOfProducts>

<speciesReference species="M_ATP__91__n__93____91__m__93__" stoichiometry="3.33" constant="true"/>

<speciesReference species="M_H2O__91__n__93__" stoichiometry="4.33" constant="true"/>

<speciesReference species="M_NAD__91__n__93____91__m__93__" stoichiometry="1" constant="true"/>

</listOfProducts>

</reaction>

<reaction metaid="NARyc_n" id="NARyc_n" name="NARyc_n" reversible="false" fast="false" fbc:lowerFluxBound="FB2N0" fbc:upperFluxBound="FB3N1000">

<listOfReactants>

<speciesReference species="M_HNO3__91__n__93__" stoichiometry="1" constant="true"/>

<speciesReference species="M_NADPH__91__n__93__" stoichiometry="1" constant="true"/>

</listOfReactants>

<listOfProducts>

<speciesReference species="M_NADP__91__n__93__" stoichiometry="1" constant="true"/>

<speciesReference species="M_NO2__91__n__93__" stoichiometry="1" constant="true"/>

</listOfProducts>

</reaction>

<reaction metaid="NiRy_n" id="NiRy_n" name="NiRy_n" reversible="false" fast="false" fbc:lowerFluxBound="FB2N0" fbc:upperFluxBound="FB3N1000">

<listOfReactants>

<speciesReference species="M_NADPH__91__n__93____91__c__93__" stoichiometry="3" constant="true"/>

<speciesReference species="M_NO2__91__n__93____91__c__93__" stoichiometry="1" constant="true"/>

</listOfReactants>

<listOfProducts>

<speciesReference species="M_NADP__91__n__93____91__c__93__" stoichiometry="3" constant="true"/>

<speciesReference species="M_NH3__91__n__93____91__c__93__" stoichiometry="1" constant="true"/>

</listOfProducts>

</reaction>

<reaction metaid="NARc_n" id="NARc_n" name="NARc_n" reversible="false" fast="false" fbc:lowerFluxBound="FB2N0" fbc:upperFluxBound="FB3N1000">

<listOfReactants>

<speciesReference species="M_HNO3__91__d__93__" stoichiometry="1" constant="true"/>

<speciesReference species="M_NADH__91__d__93__" stoichiometry="1" constant="true"/>

</listOfReactants>

<listOfProducts>

<speciesReference species="M_NAD__91__d__93__" stoichiometry="1" constant="true"/>

<speciesReference species="M_NO2__91__d__93__" stoichiometry="1" constant="true"/>

</listOfProducts>

</reaction>

<reaction metaid="NiR_n" id="NiR_n" name="NiR_n" reversible="false" fast="false" fbc:lowerFluxBound="FB2N0" fbc:upperFluxBound="FB3N1000">

<listOfReactants>

<speciesReference species="M_NADH__91__d__93____91__c__93__" stoichiometry="3" constant="true"/>

<speciesReference species="M_NO2__91__d__93____91__c__93__" stoichiometry="1" constant="true"/>

</listOfReactants>

<listOfProducts>

<speciesReference species="M_NAD__91__d__93____91__c__93__" stoichiometry="3" constant="true"/>

<speciesReference species="M_NH3__91__d__93____91__c__93__" stoichiometry="1" constant="true"/>

</listOfProducts>

</reaction>

<reaction metaid="RuBisCo_n" id="RuBisCo_n" name="RuBisCo_n" reversible="false" fast="false" fbc:lowerFluxBound="FB2N0" fbc:upperFluxBound="FB3N1000">

<listOfReactants>

<speciesReference species="M_CO2__91__n__93__" stoichiometry="1" constant="true"/>

<speciesReference species="M_H2O__91__n__93__" stoichiometry="1" constant="true"/>

<speciesReference species="M_RuBP__91__n__93____91__c__93__" stoichiometry="1" constant="true"/>

</listOfReactants>

<listOfProducts>

<speciesReference species="M_3PGA__91__n__93____91__c__93__" stoichiometry="2" constant="true"/>

</listOfProducts>

</reaction>

<reaction metaid="RuBisO_n" id="RuBisO_n" name="RuBisO_n" reversible="false" fast="false" fbc:lowerFluxBound="FB2N0" fbc:upperFluxBound="FB3N1000">

<listOfReactants>

<speciesReference species="M_H2O__91__n__93__" stoichiometry="1" constant="true"/>

<speciesReference species="M_O2__91__n__93__" stoichiometry="1" constant="true"/>

<speciesReference species="M_RuBP__91__n__93____91__c__93__" stoichiometry="1" constant="true"/>

</listOfReactants>

<listOfProducts>

<speciesReference species="M_3PGA__91__n__93____91__c__93__" stoichiometry="1" constant="true"/>

<speciesReference species="M_PGIA__91__n__93____91__c__93__" stoichiometry="1" constant="true"/>

</listOfProducts>

</reaction>

<reaction metaid="PGK_n" id="PGK_n" name="PGK_n" reversible="false" fast="false" fbc:lowerFluxBound="FB2N0" fbc:upperFluxBound="FB3N1000">

<listOfReactants>

<speciesReference species="M_3PGA__91__n__93____91__c__93__" stoichiometry="1" constant="true"/>

<speciesReference species="M_ATP__91__n__93____91__c__93__" stoichiometry="1" constant="true"/>

</listOfReactants>

<listOfProducts>

<speciesReference species="M_1_3BPGA__91__n__93____91__c__93__" stoichiometry="1" constant="true"/>

<speciesReference species="M_ADP__91__n__93____91__c__93__" stoichiometry="1" constant="true"/>

</listOfProducts>

</reaction>

<reaction metaid="GAPDHy_n" id="GAPDHy_n" name="GAPDHy_n" reversible="false" fast="false" fbc:lowerFluxBound="FB2N0" fbc:upperFluxBound="FB3N1000">

<listOfReactants>

<speciesReference species="M_1_3BPGA__91__n__93____91__c__93__" stoichiometry="1" constant="true"/>

<speciesReference species="M_NADPH__91__n__93____91__c__93__" stoichiometry="1" constant="true"/>

</listOfReactants>

<listOfProducts>

<speciesReference species="M_G3P__91__n__93____91__c__93__" stoichiometry="1" constant="true"/>

<speciesReference species="M_NADP__91__n__93____91__c__93__" stoichiometry="1" constant="true"/>

<speciesReference species="M_Pi__91__n__93____91__c__93__" stoichiometry="1" constant="true"/>

</listOfProducts>

</reaction>

<reaction metaid="GAPDH_n" id="GAPDH_n" name="GAPDH_n" reversible="true" fast="false" fbc:lowerFluxBound="FB1N1000" fbc:upperFluxBound="FB3N1000">

<listOfReactants>

<speciesReference species="M_1_3BPGA__91__n__93____91__c__93__" stoichiometry="1" constant="true"/>

<speciesReference species="M_NADH__91__n__93____91__c__93__" stoichiometry="1" constant="true"/>

</listOfReactants>

<listOfProducts>

<speciesReference species="M_G3P__91__n__93____91__c__93__" stoichiometry="1" constant="true"/>

<speciesReference species="M_NAD__91__n__93____91__c__93__" stoichiometry="1" constant="true"/>

<speciesReference species="M_Pi__91__n__93____91__c__93__" stoichiometry="1" constant="true"/>

</listOfProducts>

</reaction>

<reaction metaid="TPI_n" id="TPI_n" name="TPI_n" reversible="true" fast="false" fbc:lowerFluxBound="FB1N1000" fbc:upperFluxBound="FB3N1000">

<listOfReactants>

<speciesReference species="M_G3P__91__n__93____91__c__93__" stoichiometry="1" constant="true"/>

</listOfReactants>

<listOfProducts>

<speciesReference species="M_DHAP__91__n__93____91__c__93__" stoichiometry="1" constant="true"/>

</listOfProducts>

</reaction>

<reaction metaid="FBA_n" id="FBA_n" name="FBA_n" reversible="true" fast="false" fbc:lowerFluxBound="FB1N1000" fbc:upperFluxBound="FB3N1000">

<listOfReactants>

<speciesReference species="M_DHAP__91__n__93____91__c__93__" stoichiometry="1" constant="true"/>

<speciesReference species="M_G3P__91__n__93____91__c__93__" stoichiometry="1" constant="true"/>

</listOfReactants>

<listOfProducts>

<speciesReference species="M_FBP__91__n__93____91__c__93__" stoichiometry="1" constant="true"/>

</listOfProducts>

</reaction>

<reaction metaid="PFK1_n" id="PFK1_n" name="PFK1_n" reversible="false" fast="false" fbc:lowerFluxBound="FB2N0" fbc:upperFluxBound="FB3N1000">

<listOfReactants>

<speciesReference species="M_FBP__91__n__93____91__c__93__" stoichiometry="1" constant="true"/>

<speciesReference species="M_H2O__91__n__93__" stoichiometry="1" constant="true"/>

</listOfReactants>

<listOfProducts>

<speciesReference species="M_F6P__91__n__93____91__c__93__" stoichiometry="1" constant="true"/>

<speciesReference species="M_Pi__91__n__93____91__c__93__" stoichiometry="1" constant="true"/>

</listOfProducts>

</reaction>

<reaction metaid="TKT2_n" id="TKT2_n" name="TKT2_n" reversible="false" fast="false" fbc:lowerFluxBound="FB2N0" fbc:upperFluxBound="FB3N1000">

<listOfReactants>

<speciesReference species="M_F6P__91__n__93____91__c__93__" stoichiometry="1" constant="true"/>

<speciesReference species="M_G3P__91__n__93____91__c__93__" stoichiometry="1" constant="true"/>

</listOfReactants>

<listOfProducts>

<speciesReference species="M_E4P__91__n__93____91__c__93__" stoichiometry="1" constant="true"/>

<speciesReference species="M_Xu5P__91__n__93____91__c__93__" stoichiometry="1" constant="true"/>

</listOfProducts>

</reaction>

<reaction metaid="TKT2c_n" id="TKT2c_n" name="TKT2c_n" reversible="false" fast="false" fbc:lowerFluxBound="FB2N0" fbc:upperFluxBound="FB3N1000">

<listOfReactants>

<speciesReference species="M_E4P__91__n__93__" stoichiometry="1" constant="true"/>

<speciesReference species="M_Xu5P__91__n__93__" stoichiometry="1" constant="true"/>

</listOfReactants>

<listOfProducts>

<speciesReference species="M_F6P__91__n__93__" stoichiometry="1" constant="true"/>

<speciesReference species="M_G3P__91__n__93__" stoichiometry="1" constant="true"/>

</listOfProducts>

</reaction>

<reaction metaid="RPE_n" id="RPE_n" name="RPE_n" reversible="true" fast="false" fbc:lowerFluxBound="FB1N1000" fbc:upperFluxBound="FB3N1000">

<listOfReactants>

<speciesReference species="M_Ru5P__91__n__93____91__c__93__" stoichiometry="1" constant="true"/>

</listOfReactants>

<listOfProducts>

<speciesReference species="M_Xu5P__91__n__93____91__c__93__" stoichiometry="1" constant="true"/>

</listOfProducts>

</reaction>

<reaction metaid="RPEc_n" id="RPEc_n" name="RPEc_n" reversible="true" fast="false" fbc:lowerFluxBound="FB1N1000" fbc:upperFluxBound="FB3N1000">

<listOfReactants>

<speciesReference species="M_Ru5P__91__n__93__" stoichiometry="1" constant="true"/>

</listOfReactants>

<listOfProducts>

<speciesReference species="M_Xu5P__91__n__93__" stoichiometry="1" constant="true"/>

</listOfProducts>

</reaction>

<reaction metaid="TKT_n" id="TKT_n" name="TKT_n" reversible="false" fast="false" fbc:lowerFluxBound="FB2N0" fbc:upperFluxBound="FB3N1000">

<listOfReactants>

<speciesReference species="M_G3P__91__n__93____91__c__93__" stoichiometry="1" constant="true"/>

<speciesReference species="M_S7P__91__n__93____91__c__93__" stoichiometry="1" constant="true"/>

</listOfReactants>

<listOfProducts>

<speciesReference species="M_R5P__91__n__93____91__c__93__" stoichiometry="1" constant="true"/>

<speciesReference species="M_Xu5P__91__n__93____91__c__93__" stoichiometry="1" constant="true"/>

</listOfProducts>

</reaction>

<reaction metaid="TKTc_n" id="TKTc_n" name="TKTc_n" reversible="true" fast="false" fbc:lowerFluxBound="FB1N1000" fbc:upperFluxBound="FB3N1000">

<listOfReactants>

<speciesReference species="M_R5P__91__n__93__" stoichiometry="1" constant="true"/>

<speciesReference species="M_Xu5P__91__n__93__" stoichiometry="1" constant="true"/>

</listOfReactants>

<listOfProducts>

<speciesReference species="M_G3P__91__n__93__" stoichiometry="1" constant="true"/>

<speciesReference species="M_S7P__91__n__93__" stoichiometry="1" constant="true"/>

</listOfProducts>

</reaction>

<reaction metaid="Aldolase_n" id="Aldolase_n" name="Aldolase_n" reversible="true" fast="false" fbc:lowerFluxBound="FB1N1000" fbc:upperFluxBound="FB3N1000">

<listOfReactants>

<speciesReference species="M_DHAP__91__n__93____91__c__93__" stoichiometry="1" constant="true"/>

<speciesReference species="M_E4P__91__n__93____91__c__93__" stoichiometry="1" constant="true"/>

</listOfReactants>

<listOfProducts>

<speciesReference species="M_SBP__91__n__93____91__c__93__" stoichiometry="1" constant="true"/>

</listOfProducts>

</reaction>

<reaction metaid="Aldolasec_n" id="Aldolasec_n" name="Aldolasec_n" reversible="true" fast="false" fbc:lowerFluxBound="FB1N1000" fbc:upperFluxBound="FB3N1000">

<listOfReactants>

<speciesReference species="M_DHAP__91__n__93__" stoichiometry="1" constant="true"/>

<speciesReference species="M_E4P__91__n__93__" stoichiometry="1" constant="true"/>

</listOfReactants>

<listOfProducts>

<speciesReference species="M_SBP__91__n__93__" stoichiometry="1" constant="true"/>

</listOfProducts>

</reaction>

<reaction metaid="PFK_n" id="PFK_n" name="PFK_n" reversible="false" fast="false" fbc:lowerFluxBound="FB2N0" fbc:upperFluxBound="FB3N1000">

<listOfReactants>

<speciesReference species="M_H2O__91__n__93__" stoichiometry="1" constant="true"/>

<speciesReference species="M_SBP__91__n__93____91__c__93__" stoichiometry="1" constant="true"/>

</listOfReactants>

<listOfProducts>

<speciesReference species="M_Pi__91__n__93____91__c__93__" stoichiometry="1" constant="true"/>

<speciesReference species="M_S7P__91__n__93____91__c__93__" stoichiometry="1" constant="true"/>

</listOfProducts>

</reaction>

<reaction metaid="PFKc_n" id="PFKc_n" name="PFKc_n" reversible="false" fast="false" fbc:lowerFluxBound="FB2N0" fbc:upperFluxBound="FB3N1000">

<listOfReactants>

<speciesReference species="M_H2O__91__n__93__" stoichiometry="1" constant="true"/>

<speciesReference species="M_SBP__91__n__93__" stoichiometry="1" constant="true"/>

</listOfReactants>

<listOfProducts>

<speciesReference species="M_Pi__91__n__93__" stoichiometry="1" constant="true"/>

<speciesReference species="M_S7P__91__n__93__" stoichiometry="1" constant="true"/>

</listOfProducts>

</reaction>

<reaction metaid="RPI_n" id="RPI_n" name="RPI_n" reversible="true" fast="false" fbc:lowerFluxBound="FB1N1000" fbc:upperFluxBound="FB3N1000">

<listOfReactants>

<speciesReference species="M_R5P__91__n__93____91__c__93__" stoichiometry="1" constant="true"/>

</listOfReactants>

<listOfProducts>

<speciesReference species="M_Ru5P__91__n__93____91__c__93__" stoichiometry="1" constant="true"/>

</listOfProducts>

</reaction>

<reaction metaid="RPIc_n" id="RPIc_n" name="RPIc_n" reversible="true" fast="false" fbc:lowerFluxBound="FB1N1000" fbc:upperFluxBound="FB3N1000">

<listOfReactants>

<speciesReference species="M_R5P__91__n__93__" stoichiometry="1" constant="true"/>

</listOfReactants>

<listOfProducts>

<speciesReference species="M_Ru5P__91__n__93__" stoichiometry="1" constant="true"/>

</listOfProducts>

</reaction>

<reaction metaid="PRK_n" id="PRK_n" name="PRK_n" reversible="false" fast="false" fbc:lowerFluxBound="FB2N0" fbc:upperFluxBound="FB3N1000">

<listOfReactants>

<speciesReference species="M_ATP__91__n__93____91__c__93__" stoichiometry="1" constant="true"/>

<speciesReference species="M_Ru5P__91__n__93____91__c__93__" stoichiometry="1" constant="true"/>

</listOfReactants>

<listOfProducts>

<speciesReference species="M_ADP__91__n__93____91__c__93__" stoichiometry="1" constant="true"/>

<speciesReference species="M_RuBP__91__n__93____91__c__93__" stoichiometry="1" constant="true"/>

</listOfProducts>

</reaction>

<reaction metaid="PGI_n" id="PGI_n" name="PGI_n" reversible="true" fast="false" fbc:lowerFluxBound="FB1N1000" fbc:upperFluxBound="FB3N1000">

<listOfReactants>

<speciesReference species="M_F6P__91__n__93____91__c__93__" stoichiometry="1" constant="true"/>

</listOfReactants>

<listOfProducts>

<speciesReference species="M_G6P__91__n__93____91__c__93__" stoichiometry="1" constant="true"/>

</listOfProducts>

</reaction>

<reaction metaid="G6Pase_n" id="G6Pase_n" name="G6Pase_n" reversible="false" fast="false" fbc:lowerFluxBound="FB2N0" fbc:upperFluxBound="FB3N1000">

<listOfReactants>

<speciesReference species="M_G6P__91__n__93____91__c__93__" stoichiometry="1" constant="true"/>

<speciesReference species="M_H2O__91__n__93__" stoichiometry="1" constant="true"/>

</listOfReactants>

<listOfProducts>

<speciesReference species="M_Pi__91__n__93____91__c__93__" stoichiometry="1" constant="true"/>

<speciesReference species="M_glucose__91__n__93____91__c__93__" stoichiometry="1" constant="true"/>

</listOfProducts>

</reaction>

<reaction metaid="GltS_n" id="GltS_n" name="GltS_n" reversible="true" fast="false" fbc:lowerFluxBound="FB1N1000" fbc:upperFluxBound="FB3N1000">

<listOfReactants>

<speciesReference species="M_Glutamine__91__n__93____91__c__93__" stoichiometry="1" constant="true"/>

<speciesReference species="M_Oxoglutarate__91__n__93____91__c__93__" stoichiometry="1" constant="true"/>

</listOfReactants>

<listOfProducts>

<speciesReference species="M_Glutamate__91__n__93____91__c__93__" stoichiometry="2" constant="true"/>

</listOfProducts>

</reaction>

<reaction metaid="GS_n" id="GS_n" name="GS_n" reversible="true" fast="false" fbc:lowerFluxBound="FB1N1000" fbc:upperFluxBound="FB3N1000">

<listOfReactants>

<speciesReference species="M_ATP__91__n__93____91__c__93__" stoichiometry="1" constant="true"/>

<speciesReference species="M_Glutamate__91__n__93____91__c__93__" stoichiometry="1" constant="true"/>

<speciesReference species="M_NH3__91__n__93____91__c__93__" stoichiometry="1" constant="true"/>

</listOfReactants>

<listOfProducts>

<speciesReference species="M_ADP__91__n__93____91__c__93__" stoichiometry="1" constant="true"/>

<speciesReference species="M_Glutamine__91__n__93____91__c__93__" stoichiometry="1" constant="true"/>

<speciesReference species="M_Pi__91__n__93____91__c__93__" stoichiometry="1" constant="true"/>

</listOfProducts>

</reaction>

<reaction metaid="PGP_n" id="PGP_n" name="PGP_n" reversible="false" fast="false" fbc:lowerFluxBound="FB2N0" fbc:upperFluxBound="FB3N1000">

<listOfReactants>

<speciesReference species="M_H2O__91__n__93__" stoichiometry="1" constant="true"/>

<speciesReference species="M_PGIA__91__n__93____91__c__93__" stoichiometry="1" constant="true"/>

</listOfReactants>

<listOfProducts>

<speciesReference species="M_Glycolate__91__n__93____91__c__93__" stoichiometry="1" constant="true"/>

<speciesReference species="M_Pi__91__n__93____91__c__93__" stoichiometry="1" constant="true"/>

</listOfProducts>

</reaction>

<reaction metaid="GOXper_n" id="GOXper_n" name="GOXper_n" reversible="false" fast="false" fbc:lowerFluxBound="FB2N0" fbc:upperFluxBound="FB3N1000">

<listOfReactants>

<speciesReference species="M_Glycolate__91__n__93____91__p__93__" stoichiometry="1" constant="true"/>

<speciesReference species="M_O2__91__n__93__" stoichiometry="1" constant="true"/>

</listOfReactants>

<listOfProducts>

<speciesReference species="M_Glyoxylate__91__n__93____91__p__93__" stoichiometry="1" constant="true"/>

<speciesReference species="M_H2O2__91__n__93__" stoichiometry="1" constant="true"/>

</listOfProducts>

</reaction>

<reaction metaid="Catalasec_n" id="Catalasec_n" name="Catalasec_n" reversible="false" fast="false" fbc:lowerFluxBound="FB2N0" fbc:upperFluxBound="FB3N1000">

<listOfReactants>

<speciesReference species="M_H2O2__91__n__93__" stoichiometry="1" constant="true"/>

</listOfReactants>

<listOfProducts>

<speciesReference species="M_H2O__91__n__93__" stoichiometry="1" constant="true"/>

<speciesReference species="M_O2__91__n__93__" stoichiometry="0.5" constant="true"/>

</listOfProducts>

</reaction>

<reaction metaid="SGTper_n" id="SGTper_n" name="SGTper_n" reversible="false" fast="false" fbc:lowerFluxBound="FB2N0" fbc:upperFluxBound="FB3N1000">

<listOfReactants>

<speciesReference species="M_Glyoxylate__91__n__93____91__p__93__" stoichiometry="1" constant="true"/>

<speciesReference species="M_Serine__91__n__93____91__p__93__" stoichiometry="1" constant="true"/>

</listOfReactants>

<listOfProducts>

<speciesReference species="M_Glycine__91__n__93____91__p__93__" stoichiometry="1" constant="true"/>

<speciesReference species="M_hydroxypyruvate__91__n__93____91__p__93__" stoichiometry="1" constant="true"/>

</listOfProducts>

</reaction>

<reaction metaid="GTper_n" id="GTper_n" name="GTper_n" reversible="false" fast="false" fbc:lowerFluxBound="FB2N0" fbc:upperFluxBound="FB3N1000">

<listOfReactants>

<speciesReference species="M_Glutamate__91__n__93____91__p__93__" stoichiometry="1" constant="true"/>

<speciesReference species="M_Glyoxylate__91__n__93____91__p__93__" stoichiometry="1" constant="true"/>

</listOfReactants>

<listOfProducts>

<speciesReference species="M_Glycine__91__n__93____91__p__93__" stoichiometry="1" constant="true"/>

<speciesReference species="M_Oxoglutarate__91__n__93____91__p__93__" stoichiometry="1" constant="true"/>

</listOfProducts>

</reaction>

<reaction metaid="HPRper_n" id="HPRper_n" name="HPRper_n" reversible="false" fast="false" fbc:lowerFluxBound="FB2N0" fbc:upperFluxBound="FB3N1000">

<listOfReactants>

<speciesReference species="M_NADH__91__n__93____91__p__93__" stoichiometry="1" constant="true"/>

<speciesReference species="M_hydroxypyruvate__91__n__93____91__p__93__" stoichiometry="1" constant="true"/>

</listOfReactants>

<listOfProducts>

<speciesReference species="M_Glycerate__91__n__93____91__p__93__" stoichiometry="1" constant="true"/>

<speciesReference species="M_NAD__91__n__93____91__p__93__" stoichiometry="1" constant="true"/>

</listOfProducts>

</reaction>

<reaction metaid="GK_n" id="GK_n" name="GK_n" reversible="false" fast="false" fbc:lowerFluxBound="FB2N0" fbc:upperFluxBound="FB3N1000">

<listOfReactants>

<speciesReference species="M_ATP__91__n__93____91__c__93__" stoichiometry="1" constant="true"/>

<speciesReference species="M_Glycerate__91__n__93____91__c__93__" stoichiometry="1" constant="true"/>

</listOfReactants>

<listOfProducts>

<speciesReference species="M_3PGA__91__n__93____91__c__93__" stoichiometry="1" constant="true"/>

<speciesReference species="M_ADP__91__n__93____91__c__93__" stoichiometry="1" constant="true"/>

</listOfProducts>

</reaction>

<reaction metaid="TALAc_n" id="TALAc_n" name="TALAc_n" reversible="true" fast="false" fbc:lowerFluxBound="FB1N1000" fbc:upperFluxBound="FB3N1000">

<listOfReactants>

<speciesReference species="M_G3P__91__n__93__" stoichiometry="1" constant="true"/>

<speciesReference species="M_S7P__91__n__93__" stoichiometry="1" constant="true"/>

</listOfReactants>

<listOfProducts>

<speciesReference species="M_E4P__91__n__93__" stoichiometry="1" constant="true"/>

<speciesReference species="M_F6P__91__n__93__" stoichiometry="1" constant="true"/>

</listOfProducts>

</reaction>

<reaction metaid="OPPPc_n" id="OPPPc_n" name="OPPPc_n" reversible="false" fast="false" fbc:lowerFluxBound="FB2N0" fbc:upperFluxBound="FB3N1000">

<listOfReactants>

<speciesReference species="M_G6P__91__n__93__" stoichiometry="1" constant="true"/>

<speciesReference species="M_H2O__91__n__93__" stoichiometry="1" constant="true"/>

<speciesReference species="M_NADP__91__n__93__" stoichiometry="2" constant="true"/>

</listOfReactants>

<listOfProducts>

<speciesReference species="M_CO2__91__n__93__" stoichiometry="1" constant="true"/>

<speciesReference species="M_NADPH__91__n__93__" stoichiometry="2" constant="true"/>

<speciesReference species="M_Ru5P__91__n__93__" stoichiometry="1" constant="true"/>

</listOfProducts>

</reaction>

<reaction metaid="TALA_n" id="TALA_n" name="TALA_n" reversible="true" fast="false" fbc:lowerFluxBound="FB1N1000" fbc:upperFluxBound="FB3N1000">

<listOfReactants>

<speciesReference species="M_G3P__91__n__93____91__c__93__" stoichiometry="1" constant="true"/>

<speciesReference species="M_S7P__91__n__93____91__c__93__" stoichiometry="1" constant="true"/>

</listOfReactants>

<listOfProducts>

<speciesReference species="M_E4P__91__n__93____91__c__93__" stoichiometry="1" constant="true"/>

<speciesReference species="M_F6P__91__n__93____91__c__93__" stoichiometry="1" constant="true"/>

</listOfProducts>

</reaction>

<reaction metaid="OPPP_n" id="OPPP_n" name="OPPP_n" reversible="false" fast="false" fbc:lowerFluxBound="FB2N0" fbc:upperFluxBound="FB3N1000">

<listOfReactants>

<speciesReference species="M_G6P__91__n__93____91__c__93__" stoichiometry="1" constant="true"/>

<speciesReference species="M_H2O__91__n__93__" stoichiometry="1" constant="true"/>

<speciesReference species="M_NADP__91__n__93____91__c__93__" stoichiometry="2" constant="true"/>

</listOfReactants>

<listOfProducts>

<speciesReference species="M_CO2__91__n__93__" stoichiometry="1" constant="true"/>

<speciesReference species="M_NADPH__91__n__93____91__c__93__" stoichiometry="2" constant="true"/>

<speciesReference species="M_Ru5P__91__n__93____91__c__93__" stoichiometry="1" constant="true"/>

</listOfProducts>

</reaction>

<reaction metaid="HEX1c_n" id="HEX1c_n" name="HEX1c_n" reversible="false" fast="false" fbc:lowerFluxBound="FB2N0" fbc:upperFluxBound="FB3N1000">

<listOfReactants>

<speciesReference species="M_ATP__91__n__93__" stoichiometry="1" constant="true"/>

<speciesReference species="M_glucose__91__n__93__" stoichiometry="1" constant="true"/>

</listOfReactants>

<listOfProducts>

<speciesReference species="M_ADP__91__n__93__" stoichiometry="1" constant="true"/>

<speciesReference species="M_G6P__91__n__93__" stoichiometry="1" constant="true"/>

</listOfProducts>

</reaction>

<reaction metaid="PGIc_n" id="PGIc_n" name="PGIc_n" reversible="true" fast="false" fbc:lowerFluxBound="FB1N1000" fbc:upperFluxBound="FB3N1000">

<listOfReactants>

<speciesReference species="M_F6P__91__n__93__" stoichiometry="1" constant="true"/>

</listOfReactants>

<listOfProducts>

<speciesReference species="M_G6P__91__n__93__" stoichiometry="1" constant="true"/>

</listOfProducts>

</reaction>

<reaction metaid="PFKc1_n" id="PFKc1_n" name="PFKc1_n" reversible="false" fast="false" fbc:lowerFluxBound="FB2N0" fbc:upperFluxBound="FB3N1000">

<listOfReactants>

<speciesReference species="M_ATP__91__n__93__" stoichiometry="1" constant="true"/>

<speciesReference species="M_F6P__91__n__93__" stoichiometry="1" constant="true"/>

</listOfReactants>

<listOfProducts>

<speciesReference species="M_ADP__91__n__93__" stoichiometry="1" constant="true"/>

<speciesReference species="M_FBP__91__n__93__" stoichiometry="1" constant="true"/>

</listOfProducts>

</reaction>

<reaction metaid="FBAc_n" id="FBAc_n" name="FBAc_n" reversible="true" fast="false" fbc:lowerFluxBound="FB1N1000" fbc:upperFluxBound="FB3N1000">

<listOfReactants>

<speciesReference species="M_DHAP__91__n__93__" stoichiometry="1" constant="true"/>

<speciesReference species="M_G3P__91__n__93__" stoichiometry="1" constant="true"/>

</listOfReactants>

<listOfProducts>

<speciesReference species="M_FBP__91__n__93__" stoichiometry="1" constant="true"/>

</listOfProducts>

</reaction>

<reaction metaid="TPIc_n" id="TPIc_n" name="TPIc_n" reversible="true" fast="false" fbc:lowerFluxBound="FB1N1000" fbc:upperFluxBound="FB3N1000">

<listOfReactants>

<speciesReference species="M_G3P__91__n__93__" stoichiometry="1" constant="true"/>

</listOfReactants>

<listOfProducts>

<speciesReference species="M_DHAP__91__n__93__" stoichiometry="1" constant="true"/>

</listOfProducts>

</reaction>

<reaction metaid="GAPDc_n" id="GAPDc_n" name="GAPDc_n" reversible="true" fast="false" fbc:lowerFluxBound="FB1N1000" fbc:upperFluxBound="FB3N1000">

<listOfReactants>

<speciesReference species="M_1_3BPGA__91__n__93__" stoichiometry="1" constant="true"/>

<speciesReference species="M_NADH__91__n__93__" stoichiometry="1" constant="true"/>

</listOfReactants>

<listOfProducts>

<speciesReference species="M_G3P__91__n__93__" stoichiometry="1" constant="true"/>

<speciesReference species="M_NAD__91__n__93__" stoichiometry="1" constant="true"/>

<speciesReference species="M_Pi__91__n__93__" stoichiometry="1" constant="true"/>

</listOfProducts>

</reaction>

<reaction metaid="NonP__45__GAPDHyc_n" id="NonP__45__GAPDHyc_n" name="NonP-GAPDHyc_n" reversible="false" fast="false" fbc:lowerFluxBound="FB2N0" fbc:upperFluxBound="FB3N1000">

<listOfReactants>

<speciesReference species="M_G3P__91__n__93__" stoichiometry="1" constant="true"/>

<speciesReference species="M_NADP__91__n__93__" stoichiometry="1" constant="true"/>

</listOfReactants>

<listOfProducts>

<speciesReference species="M_3PGA__91__n__93__" stoichiometry="1" constant="true"/>

<speciesReference species="M_NADPH__91__n__93__" stoichiometry="1" constant="true"/>

</listOfProducts>

</reaction>

<reaction metaid="PGK1_n" id="PGK1_n" name="PGK1_n" reversible="true" fast="false" fbc:lowerFluxBound="FB1N1000" fbc:upperFluxBound="FB3N1000">

<listOfReactants>

<speciesReference species="M_3PGA__91__n__93__" stoichiometry="1" constant="true"/>

<speciesReference species="M_ATP__91__n__93__" stoichiometry="1" constant="true"/>

</listOfReactants>

<listOfProducts>

<speciesReference species="M_1_3BPGA__91__n__93__" stoichiometry="1" constant="true"/>

<speciesReference species="M_ADP__91__n__93__" stoichiometry="1" constant="true"/>

</listOfProducts>

</reaction>

<reaction metaid="PGMc_n" id="PGMc_n" name="PGMc_n" reversible="true" fast="false" fbc:lowerFluxBound="FB1N1000" fbc:upperFluxBound="FB3N1000">

<listOfReactants>

<speciesReference species="M_3PGA__91__n__93__" stoichiometry="1" constant="true"/>

</listOfReactants>

<listOfProducts>

<speciesReference species="M_2PGA__91__n__93__" stoichiometry="1" constant="true"/>

</listOfProducts>

</reaction>

<reaction metaid="ENOc_n" id="ENOc_n" name="ENOc_n" reversible="true" fast="false" fbc:lowerFluxBound="FB1N1000" fbc:upperFluxBound="FB3N1000">

<listOfReactants>

<speciesReference species="M_2PGA__91__n__93__" stoichiometry="1" constant="true"/>

</listOfReactants>

<listOfProducts>

<speciesReference species="M_H2O__91__n__93__" stoichiometry="1" constant="true"/>

<speciesReference species="M_PEP__91__n__93__" stoichiometry="1" constant="true"/>

</listOfProducts>

</reaction>

<reaction metaid="PYKc_n" id="PYKc_n" name="PYKc_n" reversible="false" fast="false" fbc:lowerFluxBound="FB2N0" fbc:upperFluxBound="FB3N1000">

<listOfReactants>

<speciesReference species="M_ADP__91__n__93__" stoichiometry="1" constant="true"/>

<speciesReference species="M_PEP__91__n__93__" stoichiometry="1" constant="true"/>

</listOfReactants>

<listOfProducts>

<speciesReference species="M_ATP__91__n__93__" stoichiometry="1" constant="true"/>

<speciesReference species="M_Pyruvate__91__n__93__" stoichiometry="1" constant="true"/>

</listOfProducts>

</reaction>

<reaction metaid="EX1_n" id="EX1_n" name="EX1_n" reversible="false" fast="false" fbc:lowerFluxBound="FB2N0" fbc:upperFluxBound="FB3N1000">

<listOfReactants>

<speciesReference species="M_ATP__91__n__93____91__c__93__" stoichiometry="1" constant="true"/>

<speciesReference species="M_glucose__91__n__93____91__c__93__" stoichiometry="1" constant="true"/>

</listOfReactants>

<listOfProducts>

<speciesReference species="M_ADP__91__n__93____91__c__93__" stoichiometry="1" constant="true"/>

<speciesReference species="M_G6P__91__n__93____91__c__93__" stoichiometry="1" constant="true"/>

</listOfProducts>

</reaction>

<reaction metaid="PFK1c_n" id="PFK1c_n" name="PFK1c_n" reversible="false" fast="false" fbc:lowerFluxBound="FB2N0" fbc:upperFluxBound="FB3N1000">

<listOfReactants>

<speciesReference species="M_ATP__91__n__93____91__c__93__" stoichiometry="1" constant="true"/>

<speciesReference species="M_F6P__91__n__93____91__c__93__" stoichiometry="1" constant="true"/>

</listOfReactants>

<listOfProducts>

<speciesReference species="M_ADP__91__n__93____91__c__93__" stoichiometry="1" constant="true"/>

<speciesReference species="M_FBP__91__n__93____91__c__93__" stoichiometry="1" constant="true"/>

</listOfProducts>

</reaction>

<reaction metaid="PGM_n" id="PGM_n" name="PGM_n" reversible="true" fast="false" fbc:lowerFluxBound="FB1N1000" fbc:upperFluxBound="FB3N1000">

<listOfReactants>

<speciesReference species="M_3PGA__91__n__93____91__c__93__" stoichiometry="1" constant="true"/>

</listOfReactants>

<listOfProducts>

<speciesReference species="M_2PGA__91__n__93____91__c__93__" stoichiometry="1" constant="true"/>

</listOfProducts>

</reaction>

<reaction metaid="ENO_n" id="ENO_n" name="ENO_n" reversible="true" fast="false" fbc:lowerFluxBound="FB1N1000" fbc:upperFluxBound="FB3N1000">

<listOfReactants>

<speciesReference species="M_2PGA__91__n__93____91__c__93__" stoichiometry="1" constant="true"/>

</listOfReactants>

<listOfProducts>

<speciesReference species="M_H2O__91__n__93__" stoichiometry="1" constant="true"/>

<speciesReference species="M_PEP__91__n__93____91__c__93__" stoichiometry="1" constant="true"/>

</listOfProducts>

</reaction>

<reaction metaid="PYK_n" id="PYK_n" name="PYK_n" reversible="false" fast="false" fbc:lowerFluxBound="FB2N0" fbc:upperFluxBound="FB3N1000">

<listOfReactants>

<speciesReference species="M_ADP__91__n__93____91__c__93__" stoichiometry="1" constant="true"/>

<speciesReference species="M_PEP__91__n__93____91__c__93__" stoichiometry="1" constant="true"/>

</listOfReactants>

<listOfProducts>

<speciesReference species="M_ATP__91__n__93____91__c__93__" stoichiometry="1" constant="true"/>

<speciesReference species="M_Pyruvate__91__n__93____91__c__93__" stoichiometry="1" constant="true"/>

</listOfProducts>

</reaction>

<reaction metaid="PCm_n" id="PCm_n" name="PCm_n" reversible="false" fast="false" fbc:lowerFluxBound="FB2N0" fbc:upperFluxBound="FB3N1000">

<listOfReactants>

<speciesReference species="M_ATP__91__n__93____91__m__93__" stoichiometry="1" constant="true"/>

<speciesReference species="M_CO2__91__n__93__" stoichiometry="1" constant="true"/>

<speciesReference species="M_Pyruvate__91__n__93____91__m__93__" stoichiometry="1" constant="true"/>

</listOfReactants>

<listOfProducts>

<speciesReference species="M_ADP__91__n__93____91__m__93__" stoichiometry="1" constant="true"/>

<speciesReference species="M_Oxaloacetate__91__n__93____91__m__93__" stoichiometry="1" constant="true"/>

<speciesReference species="M_Pi__91__n__93____91__m__93__" stoichiometry="1" constant="true"/>

</listOfProducts>

</reaction>

<reaction metaid="PDHm_n" id="PDHm_n" name="PDHm_n" reversible="false" fast="false" fbc:lowerFluxBound="FB2N0" fbc:upperFluxBound="FB3N1000">

<listOfReactants>

<speciesReference species="M_CoenzymeA__91__n__93____91__m__93__" stoichiometry="1" constant="true"/>

<speciesReference species="M_NAD__91__n__93____91__m__93__" stoichiometry="1" constant="true"/>

<speciesReference species="M_Pyruvate__91__n__93____91__m__93__" stoichiometry="1" constant="true"/>

</listOfReactants>

<listOfProducts>

<speciesReference species="M_AcetylCoA__91__n__93____91__m__93__" stoichiometry="1" constant="true"/>

<speciesReference species="M_CO2__91__n__93__" stoichiometry="1" constant="true"/>

<speciesReference species="M_NADH__91__n__93____91__m__93__" stoichiometry="1" constant="true"/>

</listOfProducts>

</reaction>

<reaction metaid="PDH_n" id="PDH_n" name="PDH_n" reversible="false" fast="false" fbc:lowerFluxBound="FB2N0" fbc:upperFluxBound="FB3N1000">

<listOfReactants>

<speciesReference species="M_CoenzymeA__91__n__93____91__c__93__" stoichiometry="1" constant="true"/>

<speciesReference species="M_NAD__91__n__93____91__c__93__" stoichiometry="1" constant="true"/>

<speciesReference species="M_Pyruvate__91__n__93____91__c__93__" stoichiometry="1" constant="true"/>

</listOfReactants>

<listOfProducts>

<speciesReference species="M_AcetylCoA__91__n__93____91__c__93__" stoichiometry="1" constant="true"/>

<speciesReference species="M_CO2__91__n__93__" stoichiometry="1" constant="true"/>

<speciesReference species="M_NADH__91__n__93____91__c__93__" stoichiometry="1" constant="true"/>

</listOfProducts>

</reaction>

<reaction metaid="CSm_n" id="CSm_n" name="CSm_n" reversible="false" fast="false" fbc:lowerFluxBound="FB2N0" fbc:upperFluxBound="FB3N1000">

<listOfReactants>

<speciesReference species="M_AcetylCoA__91__n__93____91__m__93__" stoichiometry="1" constant="true"/>

<speciesReference species="M_H2O__91__n__93__" stoichiometry="1" constant="true"/>

<speciesReference species="M_Oxaloacetate__91__n__93____91__m__93__" stoichiometry="1" constant="true"/>

</listOfReactants>

<listOfProducts>

<speciesReference species="M_Citrate__91__n__93____91__m__93__" stoichiometry="1" constant="true"/>

<speciesReference species="M_CoenzymeA__91__n__93____91__m__93__" stoichiometry="1" constant="true"/>

</listOfProducts>

</reaction>

<reaction metaid="ACONTm_n" id="ACONTm_n" name="ACONTm_n" reversible="true" fast="false" fbc:lowerFluxBound="FB1N1000" fbc:upperFluxBound="FB3N1000">

<listOfReactants>

<speciesReference species="M_Citrate__91__n__93____91__m__93__" stoichiometry="1" constant="true"/>

</listOfReactants>

<listOfProducts>

<speciesReference species="M_Isocitrate__91__n__93____91__m__93__" stoichiometry="1" constant="true"/>

</listOfProducts>

</reaction>

<reaction metaid="ICDHym_n" id="ICDHym_n" name="ICDHym_n" reversible="true" fast="false" fbc:lowerFluxBound="FB1N1000" fbc:upperFluxBound="FB3N1000">

<listOfReactants>

<speciesReference species="M_Isocitrate__91__n__93____91__m__93__" stoichiometry="1" constant="true"/>

<speciesReference species="M_NADP__91__n__93____91__m__93__" stoichiometry="1" constant="true"/>

</listOfReactants>

<listOfProducts>

<speciesReference species="M_CO2__91__n__93__" stoichiometry="1" constant="true"/>

<speciesReference species="M_NADPH__91__n__93____91__m__93__" stoichiometry="1" constant="true"/>

<speciesReference species="M_Oxoglutarate__91__n__93____91__m__93__" stoichiometry="1" constant="true"/>

</listOfProducts>

</reaction>

<reaction metaid="ACONTc_n" id="ACONTc_n" name="ACONTc_n" reversible="true" fast="false" fbc:lowerFluxBound="FB1N1000" fbc:upperFluxBound="FB3N1000">

<listOfReactants>

<speciesReference species="M_Isocitrate__91__n__93____91__m__93__" stoichiometry="1" constant="true"/>

<speciesReference species="M_NAD__91__n__93____91__m__93__" stoichiometry="1" constant="true"/>

</listOfReactants>

<listOfProducts>

<speciesReference species="M_CO2__91__n__93__" stoichiometry="1" constant="true"/>

<speciesReference species="M_NADH__91__n__93____91__m__93__" stoichiometry="1" constant="true"/>

<speciesReference species="M_Oxoglutarate__91__n__93____91__m__93__" stoichiometry="1" constant="true"/>

</listOfProducts>

</reaction>

<reaction metaid="ICDHyc_n" id="ICDHyc_n" name="ICDHyc_n" reversible="true" fast="false" fbc:lowerFluxBound="FB1N1000" fbc:upperFluxBound="FB3N1000">

<listOfReactants>

<speciesReference species="M_Citrate__91__n__93__" stoichiometry="1" constant="true"/>

</listOfReactants>

<listOfProducts>

<speciesReference species="M_Isocitrate__91__n__93__" stoichiometry="1" constant="true"/>

</listOfProducts>

</reaction>

<reaction metaid="ICDHc_n" id="ICDHc_n" name="ICDHc_n" reversible="true" fast="false" fbc:lowerFluxBound="FB1N1000" fbc:upperFluxBound="FB3N1000">

<listOfReactants>

<speciesReference species="M_Isocitrate__91__n__93__" stoichiometry="1" constant="true"/>

<speciesReference species="M_NADP__91__n__93__" stoichiometry="1" constant="true"/>

</listOfReactants>

<listOfProducts>

<speciesReference species="M_CO2__91__n__93__" stoichiometry="1" constant="true"/>

<speciesReference species="M_NADPH__91__n__93__" stoichiometry="1" constant="true"/>

<speciesReference species="M_Oxoglutarate__91__n__93__" stoichiometry="1" constant="true"/>

</listOfProducts>

</reaction>

<reaction metaid="AKGDm_n" id="AKGDm_n" name="AKGDm_n" reversible="true" fast="false" fbc:lowerFluxBound="FB1N1000" fbc:upperFluxBound="FB3N1000">

<listOfReactants>

<speciesReference species="M_CoenzymeA__91__n__93____91__m__93__" stoichiometry="1" constant="true"/>

<speciesReference species="M_NAD__91__n__93____91__m__93__" stoichiometry="1" constant="true"/>

<speciesReference species="M_Oxoglutarate__91__n__93____91__m__93__" stoichiometry="1" constant="true"/>

</listOfReactants>

<listOfProducts>

<speciesReference species="M_CO2__91__n__93__" stoichiometry="1" constant="true"/>

<speciesReference species="M_NADH__91__n__93____91__m__93__" stoichiometry="1" constant="true"/>

<speciesReference species="M_SuccinylCoA__91__n__93____91__m__93__" stoichiometry="1" constant="true"/>

</listOfProducts>

</reaction>

<reaction metaid="SUCOASm_n" id="SUCOASm_n" name="SUCOASm_n" reversible="true" fast="false" fbc:lowerFluxBound="FB1N1000" fbc:upperFluxBound="FB3N1000">

<listOfReactants>

<speciesReference species="M_ADP__91__n__93____91__m__93__" stoichiometry="1" constant="true"/>

<speciesReference species="M_Pi__91__n__93____91__m__93__" stoichiometry="1" constant="true"/>

<speciesReference species="M_SuccinylCoA__91__n__93____91__m__93__" stoichiometry="1" constant="true"/>

</listOfReactants>

<listOfProducts>

<speciesReference species="M_ATP__91__n__93____91__m__93__" stoichiometry="1" constant="true"/>

<speciesReference species="M_CoenzymeA__91__n__93____91__m__93__" stoichiometry="1" constant="true"/>

<speciesReference species="M_Succinate__91__n__93____91__m__93__" stoichiometry="1" constant="true"/>

</listOfProducts>

</reaction>

<reaction metaid="FUMm_n" id="FUMm_n" name="FUMm_n" reversible="true" fast="false" fbc:lowerFluxBound="FB1N1000" fbc:upperFluxBound="FB3N1000">

<listOfReactants>

<speciesReference species="M_Fumarate__91__n__93____91__m__93__" stoichiometry="1" constant="true"/>

<speciesReference species="M_H2O__91__n__93__" stoichiometry="1" constant="true"/>

</listOfReactants>

<listOfProducts>

<speciesReference species="M_Malate__91__n__93____91__m__93__" stoichiometry="1" constant="true"/>

</listOfProducts>

</reaction>

<reaction metaid="FUMc_n" id="FUMc_n" name="FUMc_n" reversible="true" fast="false" fbc:lowerFluxBound="FB1N1000" fbc:upperFluxBound="FB3N1000">

<listOfReactants>

<speciesReference species="M_Fumarate__91__n__93__" stoichiometry="1" constant="true"/>

<speciesReference species="M_H2O__91__n__93__" stoichiometry="1" constant="true"/>

</listOfReactants>

<listOfProducts>

<speciesReference species="M_Malate__91__n__93__" stoichiometry="1" constant="true"/>

</listOfProducts>

</reaction>

<reaction metaid="MDHm_n" id="MDHm_n" name="MDHm_n" reversible="true" fast="false" fbc:lowerFluxBound="FB1N1000" fbc:upperFluxBound="FB3N1000">

<listOfReactants>

<speciesReference species="M_Malate__91__n__93____91__m__93__" stoichiometry="1" constant="true"/>

<speciesReference species="M_NAD__91__n__93____91__m__93__" stoichiometry="1" constant="true"/>

</listOfReactants>

<listOfProducts>
[truncated: 665,808 more chars]
