# Supplementary material for: Enhancing control systems of higher plant culture chambers via multilevel structural mechanistic modelling
Source: Front Plant Sci. 2022 Oct 20;13:970410. doi: 10.3389/fpls.2022.970410 (PMC9632494; doi:10.3389/fpls.2022.970410)
Supplement: Supplementary file 6 [file Table_6.docx]

**Data Availability Statement**

Scripts for the reproducibility of the results are accessible in the following Github repository:

https://github.com/HigherPlantsModelling/Frontiers-in-Plant-Science
